# Negishi-coupling-enabled synthesis of α-heteroaryl-α-amino acid building blocks for DNA-encoded chemical library applications

**DOI:** 10.3762/bjoc.20.168

**Published:** 2024-08-08

**Authors:** Matteo Gasparetto, Balázs Fődi, Gellért Sipos

**Affiliations:** 1 X-Chem Zrt., Záhony u. 7, DA Building, Graphisoft Park, Budapest, 1031, Hungary

**Keywords:** amino acids, DEL, flow chemistry, Negishi, on-DNA chemistry

## Abstract

Amino acids are vital motifs in the domain of biochemistry, serving as the foundational unit for peptides and proteins, while also holding a crucial function in many biological processes. Due to their bifunctional character, they have been also used for combinatorial chemistry purposes, such as the preparation of DNA-encoded chemical libraries. We developed a practical synthesis for α-heteroaryl-α-amino acids starting from an array of small heteroaromatic halides. The reaction sequence utilizes a photochemically enhanced Negishi cross-coupling as a key step, followed by oximation and reduction. The prepared amino esters were validated for on-DNA reactivity via a reverse amidation–hydrolysis–reverse amidation protocol.

## Introduction

DNA-encoded chemical library (DEL) technology is a powerful tool for hit identification [1–2]. DELs are chemically synthesized libraries in which every member is covalently attached to a unique DNA sequence serving as a molecular “barcode” [3]. The success of this technology ultimately relies on the quality and diversity of the libraries. DEL synthesis must employ DNA-compatible reactions; hence it operates under a limited set of conditions [4–5]. DELs are typically produced via split-and-pool combinatorial chemistry methods. Using bifunctional building blocks (BBs) can quickly increase the diversity of these molecular libraries [6]. Hence, DEL practitioners constantly seek access to novel building blocks [7].

Amino acids (AAs) are vital motifs in the domain of biochemistry, serving as the foundational unit for peptides and proteins, while also holding a crucial function in many biological processes [8]. Non-canonical amino acids (NCAs) are widely used in medicinal chemistry [9]. Not surprisingly, they also find broad use as bifunctional building blocks (BBs) for DELs. In an early example, an 800-million-members DEL utilized Fmoc-amino acids as primary diversity elements [10].

The pursuit of achieving the efficient synthesis of α-amino acids has been an ongoing challenge since 1850, marked by the initial report of the Strecker condensation [11]. The Strecker synthesis and the related Bucherer−Bergs hydantoin formation remains the most employed approach for producing this family of substrates [12]. Despite its effectiveness, this approach requires hazardous cyanides and harsh conditions for the subsequent hydrolysis of the nitrile or the hydantoin. Additionally, it carries significant limitations in its scope, reducing its overall applicability.

A different approach for the synthesis of α-amino acids involves the formation of dehydroamino acids and subsequent hydrogenation [13–14]. More recently, there have been reports of techniques that utilize phase transfer catalysts (PTCs) to alkylate glycine derivatives [15–16]. A range of less widely applicable strategies have been developed as well [17–22].

**Scheme 1:**
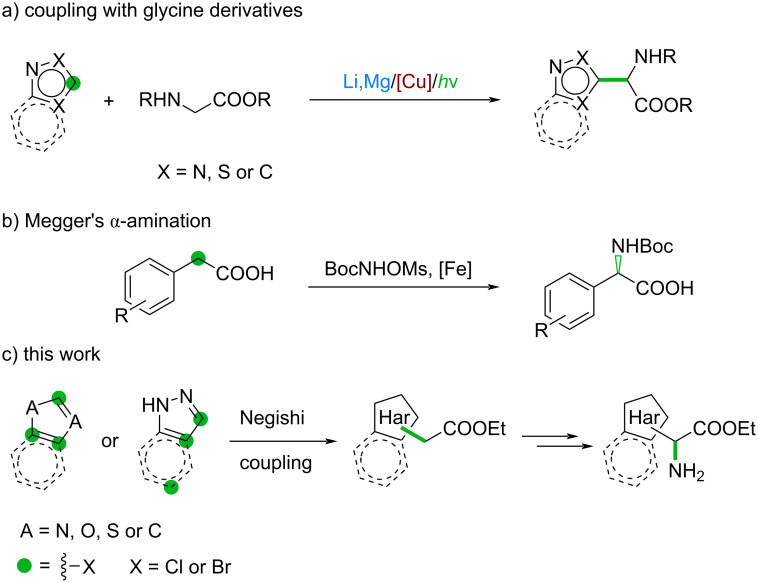
Known and improved synthetic strategies to access α-(hetero)aryl-amino acids.

The above-mentioned methods focus on the synthesis of α-alkyl-amino acids. Moving to α-aryl-amino acids, the Clayden group published an excellent asymmetric α-arylation method to access quaternary amino acids with high enantiomeric purity [23]. The synthesis of formally glycine-derived tertiary α-aryl-amino acids is much less developed. The most common strategy for obtaining these substrates is by lithiation of an aromatic ring followed by coupling with a glycine derivative (Scheme 1a). For example, this approach was applied to the synthesis of *N*-substituted pyrazoles and poly-substituted isothiazoles [24–25]. Glycine derivatives can be reacted with indoles using copper catalysis or metallophotoredox catalysis [26]. Le et al. reported the use of the same approach for imidazo[1,2-*a*]pyridines [27–28]. However, the selectivity of these photoredox reactions is driven by the structural properties of the heteroaromatic ring. During the preparation of this article, the Meggers group published an outstanding enantioselective iron-catalyzed α-amination pathway (Scheme 1b) [29]. The method is widely applicable to a broad range of substrates, however, it utilizes a catalyst that is not commercially available and small heteroaromatic rings are underrepresented in the scope.

Recognizing the importance of small heteroaromatic rings and the amino acid motif in medicinal chemistry [30–33], and aiming to expand our in-house DEL BB collection, we sought to develop a synthetic route capable of providing a broad range of α-heteroaryl-α-amino acids in a cost-effective manner (Scheme 1c).

Herein, we describe the synthesis and on-DNA validation of non-canonical α-heteroaryl-α-amino acids. We envisioned that α-heteroaryl acetates accessed through Negishi coupling can be used as key intermediates towards NCAs (Scheme 1c). Indeed, oximation of these motifs followed by reduction gave access to the desired NCAs.

## Results and Discussion

### Negishi cross-coupling step

The Negishi reaction provides convenient access to compounds featuring C(sp^2^)–C(sp^3^) bonds. However, the general view is that this transformation is less reliable than its orthogonal counterpart, the Suzuki reaction. Recent years have seen significant developments in Negishi reaction methodologies [34–39]. In particular, Alcazar et al. developed continuous flow protocols for both the generation of alkylzinc halides and for the subsequent Negishi cross-coupling reaction [40–44]. We successfully adapted Alcazar’s protocols for the synthesis of otherwise challenging heteroaryl–alkyl connections (see Table S1 in Supporting Information File 1). We decided to explore the potential of this methodology for the formation of α-heteroarylacetates. In particular, we were curious to see whether this methodology translates well for five-membered heteroarene substrates (e.g., thiazoles, pyrazoles, imidazoles) which are usually underrepresented in the peer-reviewed literature in comparison to phenyl groups or their six-membered counterparts (e.g., pyridines, pyrimidines) [42]. Furthermore, the increasing importance of small heteroaromatic rings containing nitrogen, sulfur and/or oxygen in medicinal chemistry is well depicted by the list of recently approved drugs by the FDA [31]. Fezolinetant (an NK3 receptor antagonist) and quizartinib (FLT3 inhibitor) are just a couple of examples among the drugs reaching the market in the last year.

**Scheme 2:**
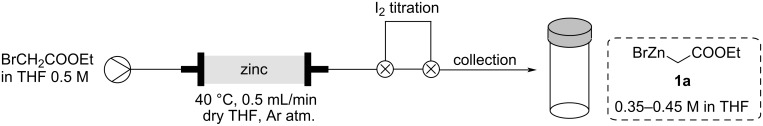
Reformatsky reagent production.

As shown in Scheme 2, ethyl (bromozinc)acetate (**1a**) was synthesized in flow by pumping a solution of ethyl 2-bromoacetate through a pre-activated zinc column (see page 11 in Supporting Information File 1) [44]. The Reformatsky reagent could be obtained in yields varying from 70 to 90% depending on the activation state of the column. The yield of the reaction was determined by titration with iodine (see page 11 Supporting Information File 1), affording final concentrations between 0.35 to 0.45 M in THF. The solution can be stored in the fridge under argon for one week before being used in the Negishi reaction. With concentrations above 0.4 M we observed crystallization of ethyl (bromozinc)acetate at the bottom of the vial after a few hours of storage in the fridge. The solid can be easily re-dissolved by gentle heating, and without affecting the product concentration and integrity.

After a brief screening, Pd(dba)_2_ and X-Phos (in a 1:2 ratio) were selected as the catalyst system for the Negishi reaction (Supporting Information File 1). Preliminary experiments were carried out with and without blue light irradiation in the PhotoCube^TM^ photoreactor [45]. These experiments revealed that while the conversion of imidazoles and pyrazoles benefits from irradiation, thiazoles seem to be largely unaffected by the presence of light (see pages 5 and 6 in Supporting Information File 1). In the case of indazoles, increased reaction rates were observed in the presence of light, but the overall yield was the same for the dark and irradiated experiments. Although these reactions are typically complete within 4 h in the dark, irradiation with blue light halves the reaction time for many compounds. Overall, these observations are in line with those of Alcazar et al. [43]. In their work, the authors demonstrated the formation of a complex between palladium and the organozinc reagent which is absorbing in the blue region. This complex then accelerates the oxidative addition of the aryl halide to the metal, which is usually the rate-limiting step for palladium-catalyzed cross-couplings. Based on these results we decided to perform all Negishi reactions under blue light irradiation.

**Scheme 3:**
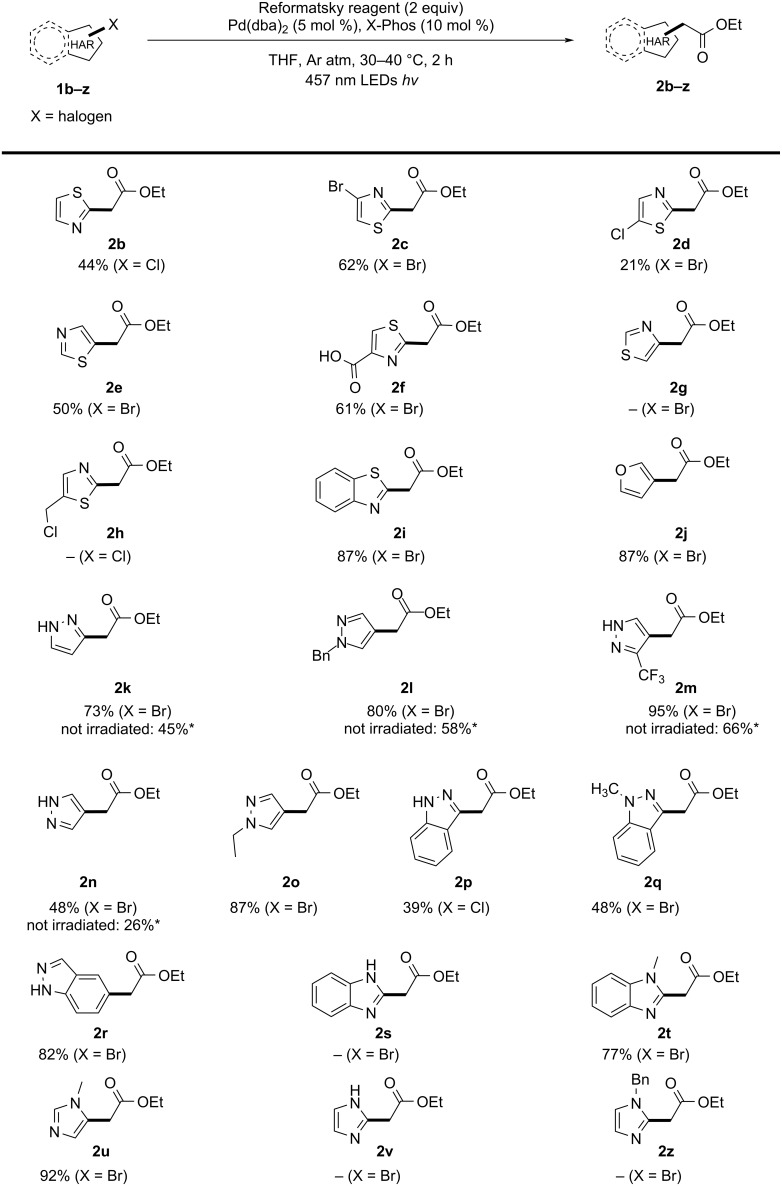
Scope of ethyl heteroarylacetates. Isolated yields are given. *Dark reactions were carried out for 4 h.

With the optimized conditions in hand, we proceeded with the investigation of the heteroaryl halide scope in batch (Scheme 3). Thiazoles proved to be challenging substrates typically affording the desired products in moderate yields (**2b**–**h**). While 2-chlorothiazole led to the production of **2b** in 44% yield, 2-bromo-5-chlorothiazole only afforded 21% yield (**2d**). Gratifyingly, the reaction selectively proceeded in position 2 of the ring. Position 4 seems to be inert to the Negishi coupling conditions as illustrated by substrates **2c** and **2g**. Somewhat surprisingly, LCMS analysis indicated that **1g** did not go through oxidative addition and remained unreacted. Formation of **2h** did not occur, however, we observed the formation of unidentified side products. Interestingly, the presence of a free carboxylic group is well tolerated (**2f**). Benzothiazole **1i** proved to be an excellent substrate for this reaction, leading to the desired acetate **2i** in high yield. A similar result was obtained for the furanyl derivative **2j**. Pyrazoles were the only substrate class which clearly benefited from light irradiation (**2k**–**o**), displaying not just a shorter reaction time but also higher yields. Even unprotected pyrazoles (**2k**, **2m**, **2n**) performed well, showing that *N*-protection is not mandatory for this transformation.

3-Bromo- and 3-chloroindazoles offered moderate yields (39% and 48% for **2p** and **2q**, respectively), while 5-substituted **2r** was isolated with 82% yield. In contrast to pyrazoles and indazoles, benzimidazoles and imidazoles required the protection of the aromatic NH group (**2s** vs **2t**; **2v** vs **2u**). Reactions with unprotected imidazoles **1s** and **1v** led to immediate formation of a precipitate upon addition of the Reformatsky reagent. Surprisingly, **1z** did not afford the expected product (**2z**).

The synthesis of the Reformatsky reagent can be combined with the Negishi cross-coupling step in a continuous flow manner [41–43]. Continuous flow chemistry offers superior control over reaction parameters compared to traditional batch methods. This approach leads to reproducible reactions, improved safety features, and it can facilitate high-throughput screening and rapid optimization [46–47]. Homogenous heating and mixing in flow reactors can lead to higher reaction rates and yields. In terms of photochemistry, continuous flow setups provide enhanced light irradiation as well [48–49]. These advantages make flow chemistry a powerful tool for chemical synthesis and industrial applications [50–51].

**Scheme 4:**
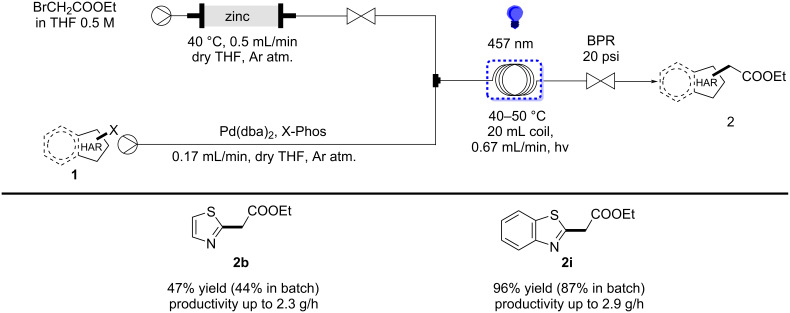
Telescoped flow synthesis of heteroarylacetates.

To assess the advantage of moving from batch to flow, the production of compounds **2b** and **2i** was carried out with the telescoped approach. Despite the difference in the yield being minimal, the rate of the transformation showed a significant improvement under continuous flow conditions, leading to reaction completion within 30 minutes (Scheme 4).

### Oxime formation

Once the ethyl heteroarylacetates scope was completed we turned our attention to the incorporation of the amino group. There are precedents for α-aminations, but we were not able to find a method suitable for our needs [52–53]. Benzylic bromination followed by nucleophilic substitution offers a general approach for the introduction of the nitrogen atom [54–56]. Consequently, the continuous flow Wohl–Ziegler bromination of **2b** was attempted [57]. Even though we could observe excellent LCMS-conversion for the mono-brominated compound, we encountered several problems related to the stability of the product (see Supporting Information File 1).

**Scheme 5:**
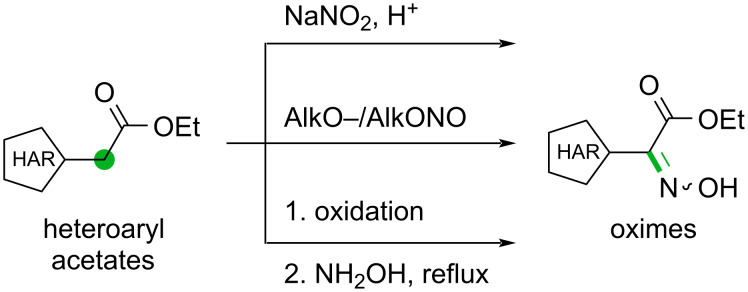
Potential routes for the preparation of oximes.

To circumvent these issues, we came across the possibility of inserting an oximino group into the benzylic position which can then be converted into an amino group by reduction. We reasoned that increasing the sp^2^ fraction and the rigidity of the whole structure will lead to increased stability of these derivatives. The first exploratory attempts demonstrated the easy preparation and the high bench stability of the oxime derivatives, therefore we opted to proceed using this route. In this study, we explored three distinct approaches commonly employed for the introduction of the oximino group into a molecule. The first approach is based on the generation of the nitrosonium ion from sodium nitrite under acidic conditions (Scheme 5, top) [58–59]. Additionally, another very common method involves the employment of a strong base, typically sodium ethoxide or methoxide, in combination with an alkyl nitrite to promote the incorporation of the oximino group (Scheme 5, middle) [60–61]. Furthermore, a widely adopted strategy involves the conversion of a carbonyl group to an oxime through condensation with hydroxylamine (Scheme 5, bottom) [62–64].

**Scheme 6:**
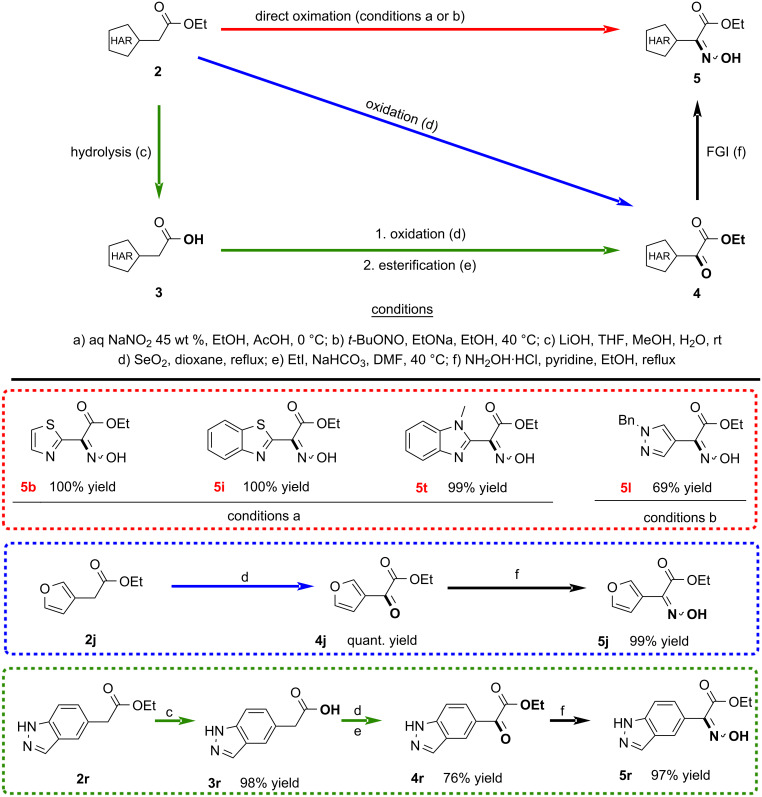
Oxime group insertion step.

In order to develop a synthetic approach applicable to several different substrates, we decided to screen the three methods on one example from each type of heteroaryl halides. According to our experimental results, thiazole **2b**, benzothiazole **2i** and benzimidazole **2t** react very well with sodium nitrite in an acidic environment (Scheme 6, red section). Among the various subclasses of compounds, pyrazole **2l** exhibited a high reactivity using *t*-BuONO and EtONa in ethanol (Scheme 6, red section). On the other hand, no reaction was observed with indazoles and furans using the first two conditions, requiring the formation of the ketoesters **4j** and **4r** first, followed by the functional group interconversion (FGI) with NH_2_OH·HCl (Scheme 6, blue and green sections).

The Riley oxidation of the furanyl derivative **2j** proceeded smoothly yielding **4j** in quantitative yield. However, obtaining compound **4r** presented some challenges due to the resistance of ester **2r** towards conventional oxidation methods (see Table S5 in Supporting Information File 1). Consequently, a multi-step process involving ester hydrolysis and subsequent re-esterification was necessary to achieve the desired ketoester.

### Reduction of the oximes

**Scheme 7:**
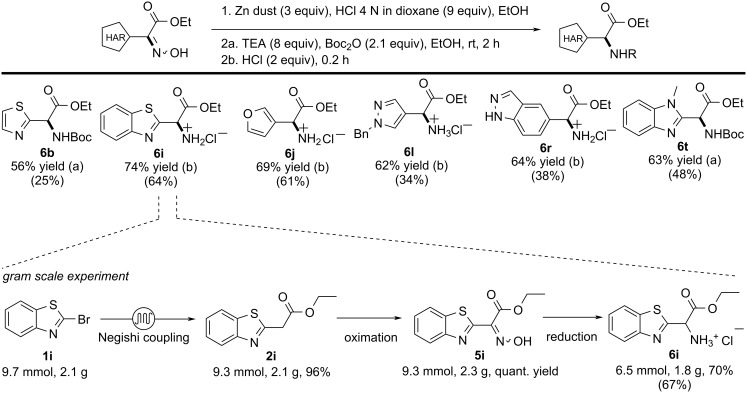
Amino ester production: general scheme, scope and gram scale experiment. The numbers in brackets represent the overall yield for the synthesis of the amino esters from the Negishi coupling till the reduction/protection step.

Oximes are commonly reduced to the corresponding amines using either palladium on activated carbon and hydrogen gas [65–68], or with zinc and a Brønsted acid as source of hydrogen [68–69]. Both methods were tested and after a brief optimization process, zinc dust and HCl in a mixture of EtOH/dioxane proved to be the best conditions in order to maximize the yield and limit the amino ester instability issues (see Table S6 in Supporting Information File 1). By slightly adjusting the reaction time and the temperature, all oxime derivatives underwent reduction to yield the corresponding amine. The amino esters were effectively safeguarded against degradation through the immediate formation of the HCl salt or by Boc-protection. This procedure allowed us to obtain all the protected amino acids in a yield that varies from 56 to 74% (Scheme 7).

### Gram scale experiment

With the optimized synthetic route, we were able to reach the final targets in good to excellent overall yields (from 25% to 64%, see Scheme 7). To test the robustness of our approach, the synthesis of compound **6i** was carried out on gram scale starting from 2.1 g of 2-bromobenzothiazole (**1i**). The gram scale production showed comparable results to those obtained in the small-scale procedure, leading to the formation of 1.8 g (67% overall yield) of the final product **6i** (Scheme 7, bottom).

### On-DNA validation

Due to the large complexity of DELs, there is only limited opportunity to track the efficiency of individual reactions during library synthesis. Therefore, BBs need to pass validation before being used in library synthesis settings. For these bifunctional amino esters, we performed a three-step validation where they were first attached to carboxylic acid functionalized DNA headpiece **7a** (first reverse amidation). Next, the ester was hydrolyzed to obtain acid **9**, and finally, a second reverse amidation with aniline afforded **10**.

**Scheme 8:**
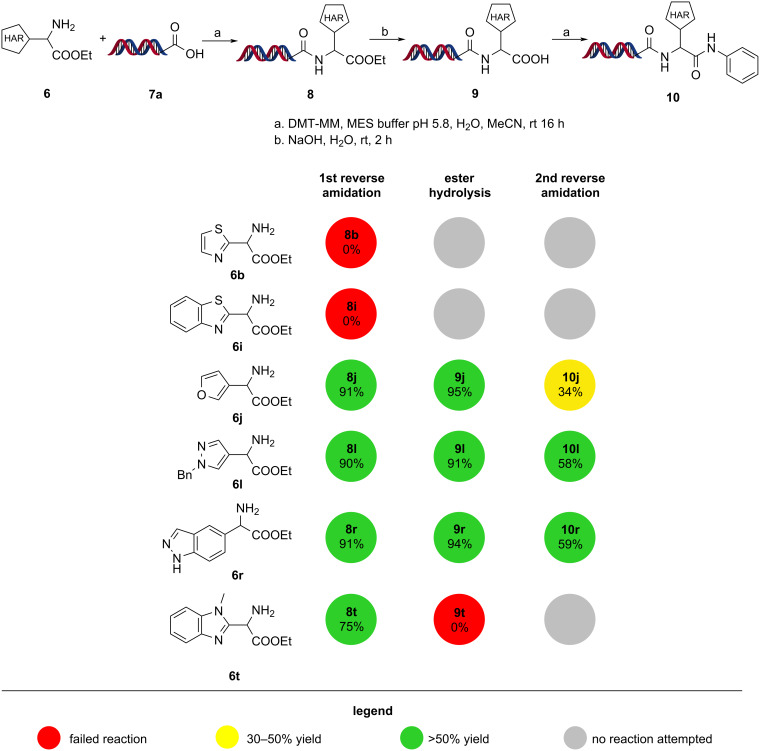
Reactions scheme and results for the on-DNA experiments. The reported values represent the normalized yield for each reaction (Supporting Information File 1).

Both the reverse amidation and the ester hydrolysis were performed following literature protocols [70–71]. In these experiments, compounds **6b** and **6i** proved to be unstable under on-DNA conditions as they failed to form esters **8b** and **8i**. Closely related structures, such as α-aminobenzothiazol-2-ylacetic acid is known to undergo decarboxylation at room temperature [72]. Compound **8t** underwent decarboxylation during the hydrolysis step. Compounds **6j**, **6l** and **6r** passed validation in moderate to good yields (Scheme 8).

## Conclusion

In conclusion, by taking advantage of the recent advances in the Negishi cross-coupling reaction we obtained a broad range of heteroarylacetates starting from heteroaromatic halides. One compound from each subclass of medicinal chemistry-relevant substrates (thiazoles, pyrazoles, etc.) was used for the preparation of α-heteroaryl-α-amino esters via the insertion of an oxime group and subsequent reduction step. The procedure relies solely on readily available and widely used reagents, rendering our approach well-suited for both industrial and academic settings. The synthesized amino esters were engaged in a three-step on-DNA validation protocol, demonstrating their possible application for DEL production.

## Supporting Information

File 1 Experimental part and NMR spectra.

## Data Availability

All data that supports the findings of this study is available in the published article and/or the supporting information to this article.
