# Supplementary material for: Negishi-coupling-enabled synthesis of α-heteroaryl-α-amino acid building blocks for DNA-encoded chemical library applications
Source: Beilstein J Org Chem. 2024 Aug 8;20:1922–32. doi: 10.3762/bjoc.20.168 (PMC11318629; doi:10.3762/bjoc.20.168)
Supplement: File 1 — Experimental part and NMR spectra. [file Beilstein_J_Org_Chem-20-1922-s001.pdf]

BEILSTEIN JOURNAL OF ORGANIC CHEMISTRY

Supporting Information

for

Negishi-coupling-enabled synthesis of α-heteroaryl-α-amino acid building blocks for DNA-encoded chemical library applications

Matteo Gasparetto, Balázs Fődi and Gellért Sipos

Beilstein J. Org. Chem. **2024,** *20,* 1922–1932. doi:10.3762/bjoc.20.168

Experimental part and NMR spectra

License and Terms: This is a supporting information file under the terms of the Creative Commons Attribution License (https://creativecommons.org/licenses/by/4.0). Please note that the reuse, redistribution and reproduction in particular requires that the author(s) and source are credited and that individual graphics may be subject to special legal provisions.
The license is subject to the *Beilstein Journal of Organic Chemistry* terms and conditions: (https://www.beilstein-journals.org/bjoc/terms)

Table of Contents

General information ... S3

Off-DNA experiments ... S3

On-DNA experiments ... S3

Figures ... S4

a) Headpiece ... S4

b) Headpiece-AOP ... S4

Optimization ... S5

a) Optimization of the Negishi conditions ... S5

Effect of blue light irradiation (457 nm) on pyrazoles ... S6

Effect of blue light irradiation (457 nm) on indazoles ... S6

Halogen effect study ... S7

b) Wohl-Ziegler bromination attempt ... S7

c) Optimization of the oxidation step ... S8

d) Optimization of the reduction conditions ... S9

Experimental procedures ... S10

a) Synthesis of *N*-methylated heteroaryl bromide starting materials ... S10

c) Characterization of ethyl heteroaryl acetates ... S12

d) Characterization of ketoesters **4j** and **4r** ... S21

e) Characterization of oximes **5b**, **5i**, **5t**, **5j**, **5r** and **5l** ... S23

f) Characterization of the amino esters ... S26

g) Validation on-DNA ... S29

Analytical data on-DNA reactions ... S34

NMR Spectra ... S45

NMR Spectra of the starting materials ... S45

NMR Spectra of the ethyl heteroaryl acetates ... S50

NMR spectra of the intermediates for the oxime formation ... S87

NMR spectra oximes ... S93

NMR spectra amino esters ... S109

References ... S121

General information

Off-DNA experiments

Unless otherwise stated, reagents were purchased from commercial suppliers and were used without further purification. TLC analyses were performed on pre-coated (0.20 mm) aluminum supported silica gel plates (ALLUGRAM® Xtra SIL G/UV_{254}). Compounds were visualized by exposure to UV light and/or by staining the plates with vanillin or permanganate stains. Flash column chromatography (FCC) purifications were carried out on silica gel (20–60 µm) on CombiFlash NextGen 300 instruments. Preparative HPLC purifications were carried out using C18 columns (5 µm). ^{1}H, ^{13}C NMR spectra and 2D experiments were recorded on a Bruker Avance 500 MHz or on a Varian 400 MHz instrument, using either [D_6]DMSO, [D_4]methanol or [D]chloroform as solvent. Chemical shifts are reported in parts per million (ppm) relative to the residual peak of the solvent. The following abbreviations are used to describe peak patterns where appropriate: s = singlet, d = doublet, dd = doublet of doublet, dt = doublet of triplet, t = triplet, q = quartet, m = multiplet and br = broad signal. Coupling constants (*J* value) are given in Hertz [Hz]. HPLC-MS data were obtained on Waters Acquity H-Class instruments. High resolution mass spectrometry (HRMS) analysis was performed with a Thermo Scientific™ Orbitrap Exploris™ mass spectrometer under ESI in positive ionization mode detection.

Flow reactions were carried out in a custom-made setup with an HPLC pump and PFA tube coil. Photochemical reactions were carried out in the PhotoCube under 457 nm LED irradiation (128 W – 1500 mA).

On-DNA experiments

All commercial reagents were used as received without further purification. The molecular structure of the DNA headpiece and the headpiece-AOP constructs are depicted on Figure S1 and S2. Reactions were performed in Eppendorf Safe-Lock microcentrifuge tubes without stirring. Reaction mixtures were vortexed with a Scientific Industries Vortex-Genie® 2 mixer. After the final ethanol precipitation, an Eppendorf® Centrifuge 5425 R or an Eppendorf® Centrifuge 5810 R was used for centrifugation. Amicon® Ultracel® - 3K – 0.5 mL were used for spin filtrations. UPLC-MS analyses were performed on a Thermo Scientific™ Vanquish Flex UPLC System coupled with a Thermo Scientific™ Orbitrap Exploris™ mass spectrometer under ESI in negative ionization mode detection. On-DNA reaction conversion rates were determined based on UV traces of UPLC-MS analysis at 260 nm. Low molecular weight impurities, M < 1000 g/mol, which were not DNA-derived, and impurities from previous reactions were not taken into account. According to this, the normalized yield was calculated by dividing the product UV conversion by the starting material purity. Optical density measurements were done with a Thermo Scientific™ Nanodrop One UV-vis spectrophotometer.

Figures

a) Headpiece

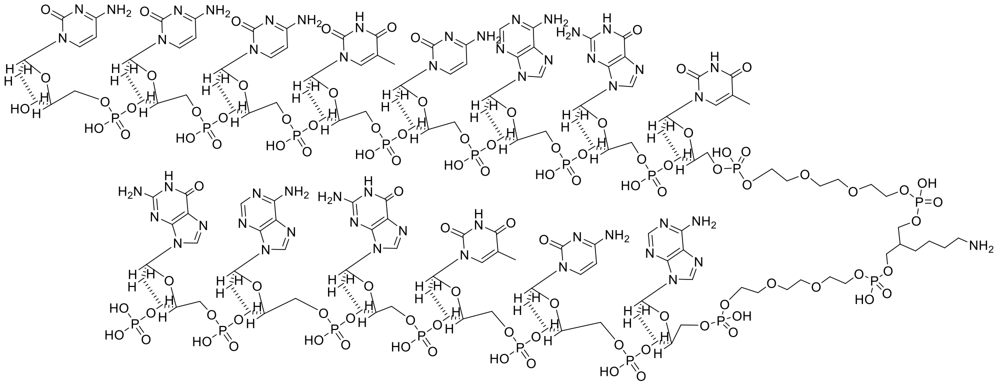

Figure S1. Sequence and structure of the "headpiece". MW = 4937 Da (Exact mass: 4934.88 Da)
Sequence: 5’-/5Phos/GAGTCA/iSp9/iUniAmM/iSp9/TGACTCCC-3’

b) Headpiece-AOP

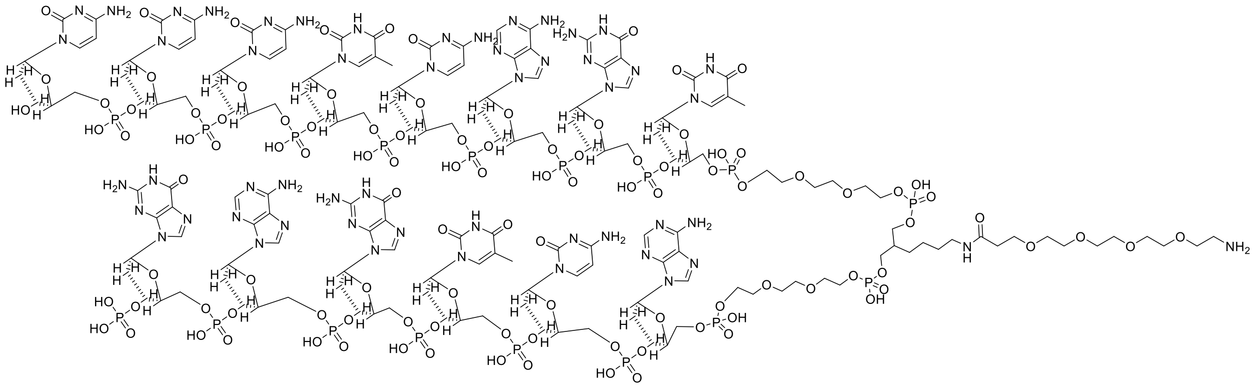

Figure S2. Structure of Headpiece-AOP. MW = 5184 Da (Exact mass: 5182.02 Da)[1]

Optimization

a) Optimization of the Negishi conditions

Table S1. Preliminary experiment for catalyst optimization

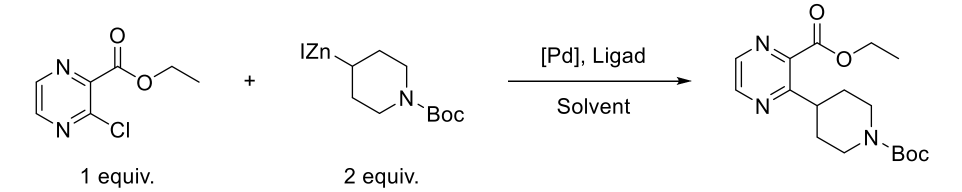

Entry	*Conditions*	*LCMS yield*
1	$NiCl_2$-glyme (5 mol %), dtbbpy (7.5 mol %), DMF	0%
2	$Pd(dba)_2$ (5 mol %), XantPhos (5 mol %), THF	31%
3	$PdCl_2$(dppf) CH_2Cl_2 (3.3 mol %), CuI (6.6 mol %), DMA	8%
4	$Pd(dba)_2$ (5 mol %), JohnPhos (10 mol %), THF	60%
5	**$Pd(dba)_2$ (5 mol %), X-Phos (10 mol %), THF**	**90%**

Table S2. Optimization of the reaction conditions

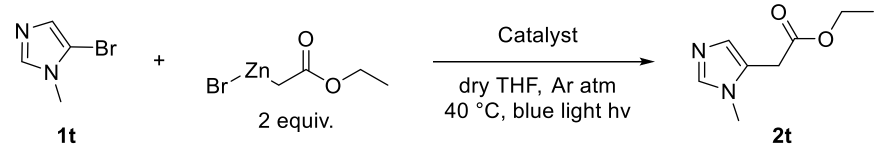

Entry	*t_r / h*	*Light*	*Catalyst*	*Conv.*[c]
1	**2**	**On**	**$Pd(dba)_2$/XPhos[a]**	**100%**
2	2	Off	$Pd(dba)_2$/XPhos[a]	88%
3	2	On	$PdCl_2$(dppf)[b]	0%
4	2	Off	$PdCl_2$(dppf)[b]	0%

[a] 5 mol% [Pd], 10 mol% ligand. [b] 5 mol% [Pd]. [c] Conversion data based on LCMS.

Table S3. Optimization Reformatsky reagent's equivalents

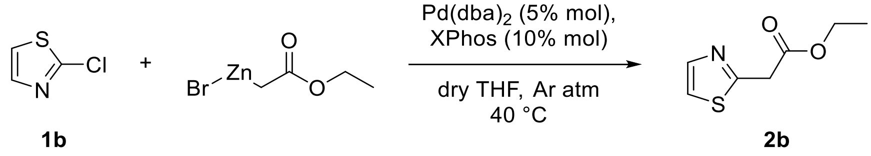

Entry	*t_r / h*	*Light*	*RZnBr Eq.*	*LCMS yield*	*Isolated yield*
1	2	On	1	73%	15%
2	2	Off	1	55%	12%
3	**2**	**On**	**2**	**100%**	**46%**
4	2	Off	2	100%	42%

Effect of blue light irradiation (457 nm) on pyrazoles

a)

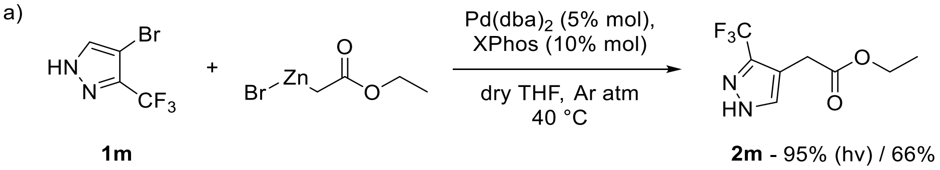

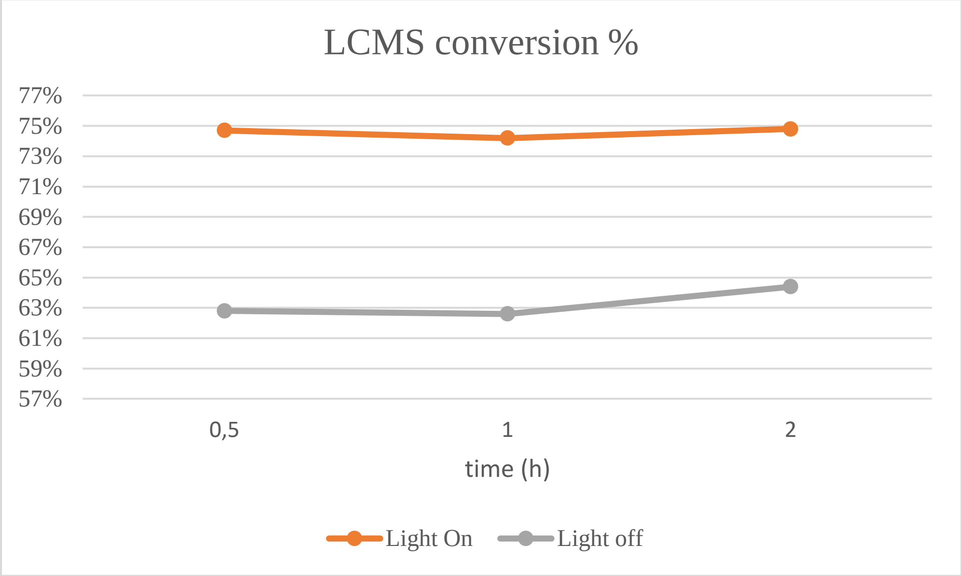

Effect of blue light irradiation (457 nm) on indazoles

b)

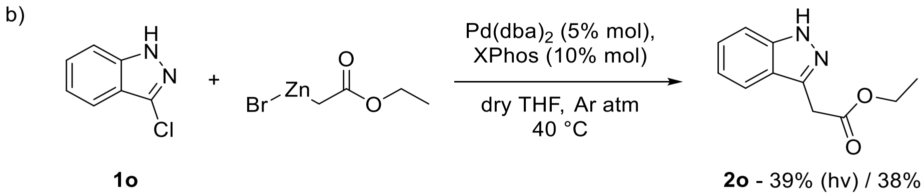

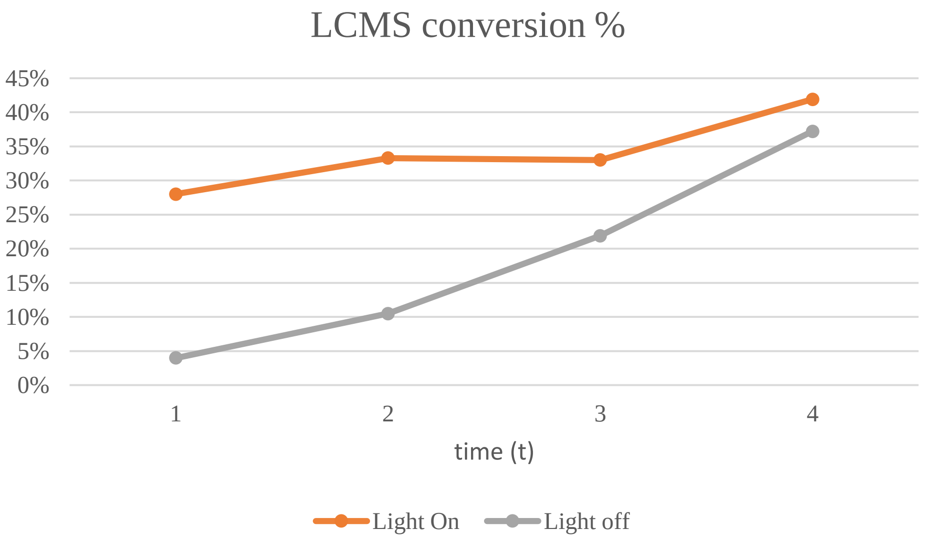

Halogen effect study

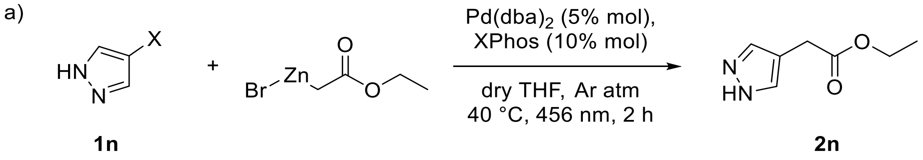

X	*LCMS yield*
Cl	0%
Br	52%
I	78%

b) Wohl-Ziegler bromination attempt

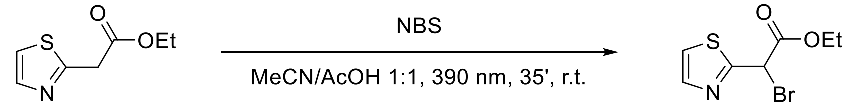

>80% by LCMS

Despite the good results measured by LCMS, the procedure was abandoned due to reproducibility issues originating from the low stability of the product. Product degradation occurred in multiple instances even after a simple work up (dilution with DCM, washing with water and brine followed by concentration).

c) Optimization of the oxidation step

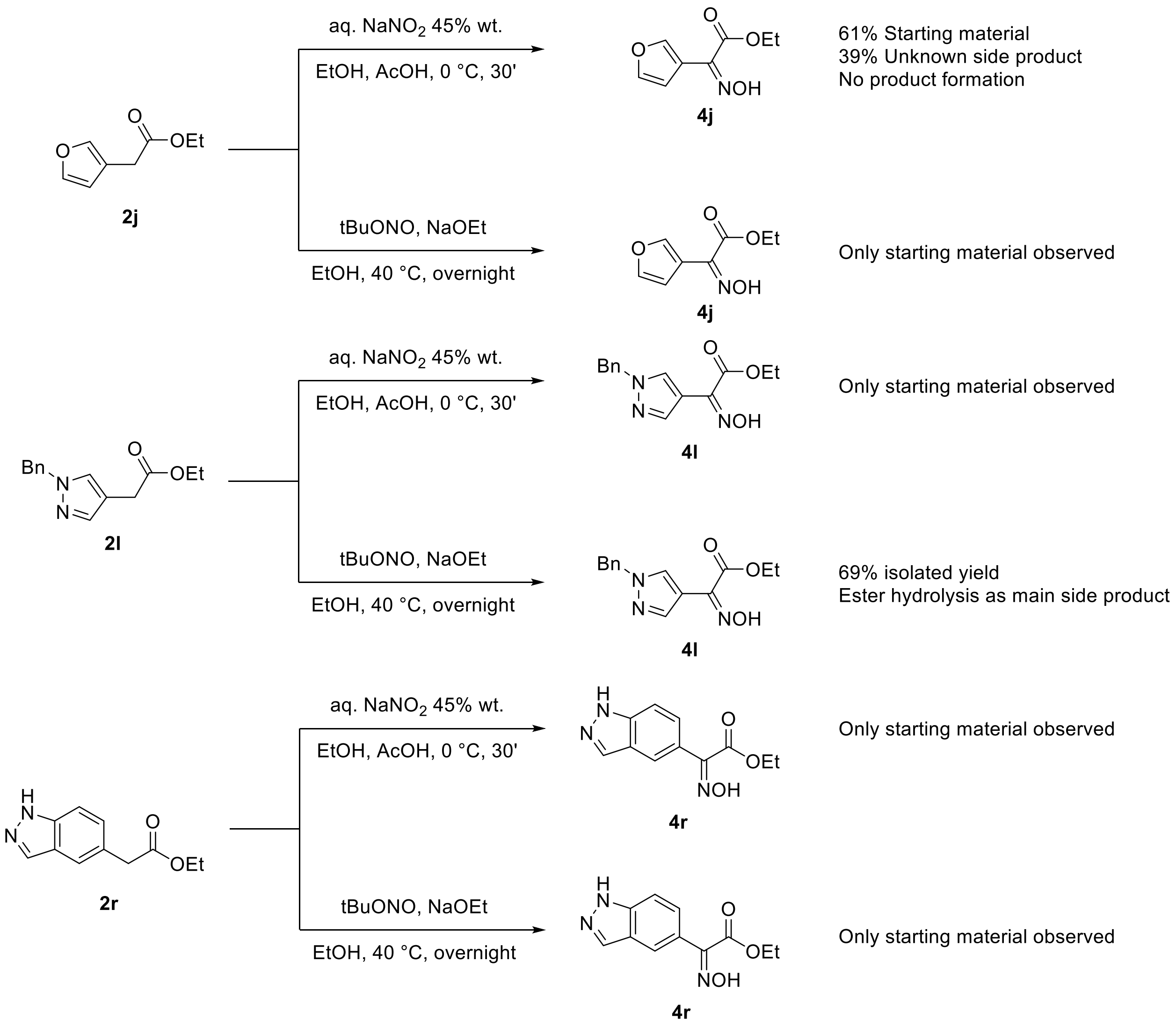

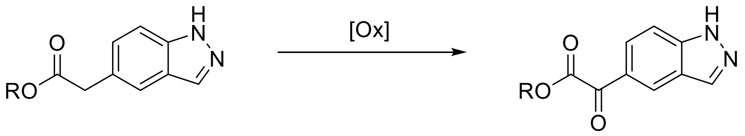

2r **4r**

Table S5. Condition tested for the oxidation of the indazolyl derivative

R	*[Ox]*	*LCMS yield*
Et	TBADT, O_2, HCl, 365 nm	0%
Et	Langlois reagent, O_2, 390 nm	0%
Et	NBS, O_2, 390 nm	0%
Et	$KMnO_2$/Alumina	0%
Et	SeO_2/Dioxane	0%
Et	SeO_2/Pyridine	0%
Et	PCC	0%
H	**SeO_2/Dioxane**	**45% (18 h), 92% (36 h)**
H	SeO_2/Pyridine	16% (18 h)

d) Optimization of the reduction conditions

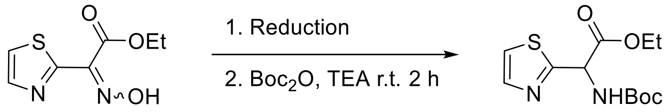

5b **6b**

Table S6. Optimized conditions for the oxime reduction

Entry	*Reduction conditions*	*Yield*
3a	Pd/C, H_2(balloon), $HCl_{(cat)}$, EtOH, 40°C, overnight	25%
3a	**Zn dust, HCl 4N in dioxane, EtOH, -15 °C 1 h then rt 3 h**	**56%**

Experimental procedures

a) Synthesis of *N*-methylated heteroaryl bromide starting materials

3-Bromo-1-methyl-1*H*-indazole (1q)

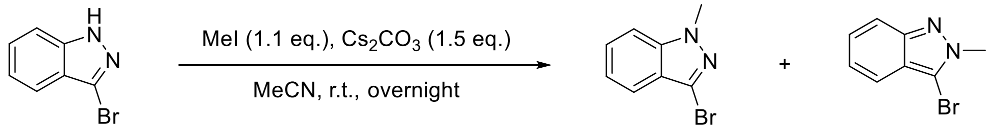

1q

3-Bromo-1*H*-indazole (985 mg, 5.0 mmol, 1.0 equiv) was dissolved in MeCN (35 mL), then cesium carbonate (2.4 g, 7.5 mmol, 1.5 equiv) was added. Methyl iodide (0.8 g, 0.3 mL, 5.6 mmol, 1.1 equiv) was added dropwise to the suspension and the reaction was stirred at room temperature for 18 hours. After completion, the mixture was diluted with water (15 mL) and EtOAc (15 mL) and stirred vigorously for 30 minutes. The volatiles were removed under reduced pressure and the aqueous phase was diluted with brine (30 mL) and extracted with EtOAc (2 times). The combined organic phases were washed with brine, dried on magnesium sulphate, and concentrated under reduced pressure. The crude was purified by flash chromatography to afford compound **1p** as a white solid (846 mg, 4.0 mmol, 80% yield). Formation of 3-bromo-2-methyl-2*H*-indazole was observed as a minor isomer but the compound was not isolated.

^{1}H NMR ($CDCl_3$, 400 MHz): *δ* 7.61 (dt, *J* = 8.2, 1.0 Hz, 1H), 7.44 (ddd, *J* = 8.5, 6.7, 1.1 Hz, 1H), 7.37 (dt, *J* = 8.5, 0.9 Hz, 1H), 7.21 (ddd, *J* = 8.2, 6.8, 0.9 Hz, 1H), 4.05 (s, 3H).

^{13}C NMR ($CDCl_3$, 100 MHz): *δ* 141.25, 127.61, 123.84, 121.38, 120.52, 120.05, 109.30, 36.04.

NMR spectra match the ones found in the literature [2].

MS–ESI (m/z): $[M+H]^+$ 210.9.

2-Bromo-1-methyl-1*H*-benzo[d]imidazole (1t)

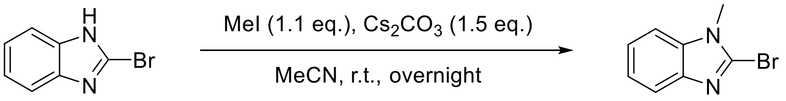

1t

2-Bromo-1*H*-benzimidazole (1.0 g, 5.1 mmol, 1.0 equiv) was dissolved in MeCN (35 mL), then cesium carbonate (2.5 g, 7.6 mmol, 1.5 equiv) was added. Methyl iodide (0.8 g, 0.4 mL, 5.6 mmol, 1.1 equiv) was added dropwise to the suspension and the reaction was stirred at room temperature for 18 hours. After completion, the mixture was diluted with water (15 mL) and EtOAc (15 mL) and stirred vigorously for 30 minutes. The volatiles were removed under reduced pressure and the aqueous phase was diluted with brine (30 mL) and extracted with EtOAc (2 times). The combined organic phases were washed with brine, dried on magnesium sulphate, and concentrated under reduced pressure. The crude was purified by flash chromatography to afford compound **1t** as a grey solid (995 mg, 4.9 mmol, 93% yield).

^{1}H NMR ($CDCl_3$, 400 MHz): *δ* 7.68 (dd, *J* = 6.7, 1.4 Hz, 1H), 7.31–7.19 (m, 3H), 3.76 (s, 3 H).

^{13}C NMR ($CDCl_3$, 100 MHz): *δ* 143.21, 136.23, 130.62, 123.25, 122.68, 119.50, 109.43, 31.80.

NMR spectra match the ones found in the literature [3].

MS–ESI (m/z): $[M+H]^+$ 210.9.

b) Procedure for the synthesis of the Reformatsky reagent

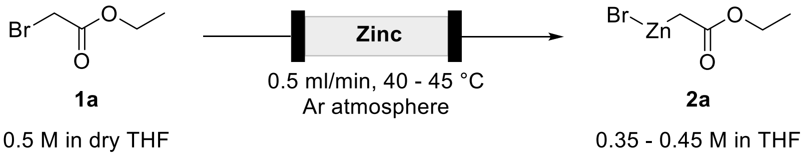

Ethyl bromozincacetate (**2a**) was prepared following literature procedure [4]. Ethyl 2-bromoacetate **1a** (0.5 M in dry THF) was pumped through a glass column filled with pre-activated zinc at a flow rate of 0.5 mL/min under argon atmosphere. The temperature was maintained between 40 and 45 °C during the whole procedure. The assay yield was determined by titration with iodine.

Titration procedure

Iodine (generally between 10 – 20 mg) was weighted into a 1.5 mL scaffold vial and an argon atmosphere was created inside the vial. The bromozinc reagent solution was added dropwise to the vial under argon till the brown color completely disappeared. The added volume of the Reformatsky reagent's solution was measured and used to calculate its concentration with the following equation:

$$[RZnBr](M) = \frac{W\ Iodine\ (mg)}{MW\ Iodine\ (\frac{mg}{mmol}) \times V_{solution}\ (ml)}$$

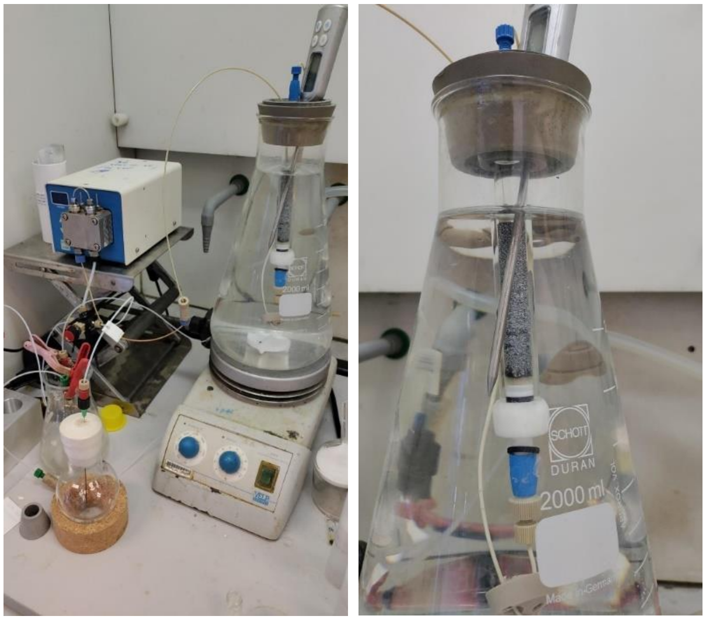

Figure S3. Flow setup for alkyl zinc halide formation

c) Characterization of ethyl heteroaryl acetates

General procedures for the synthesis of ethyl 2-heteroaryl acetates

Procedure A

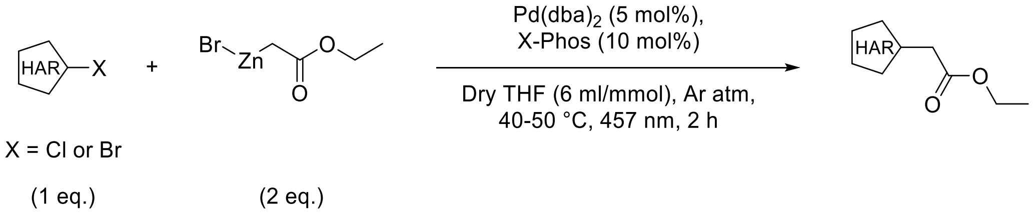

$Pd(dba)_2$ (5 mol%), X-Phos (10 mol%) and heteroaryl halide (1 equiv) were added into a flame-dried screw-cap vial equipped with a magnetic stirring bar. Argon atmosphere was created inside the vial and the reagents were dissolved in dry THF (1.5 mL/mmol). Finally, a solution of the Reformatsky reagent 0.4 M in THF (2 equiv) was added with a syringe and the reaction mixture was stirred under 457 nm light irradiation for 2 h at 40–50 °C in the PhotoCube. The reaction was monitored by LCMS. After completion, the reaction mixture was diluted with EtOAc and washed with saturated aq. NH_4Cl. The aqueous phase was extracted one more time with EtOAc, then the organic phases were merged, dried over Na_2SO_4 and concentrated under reduced pressure. The crude was purified by preparative HPLC or FCC.

Procedure B

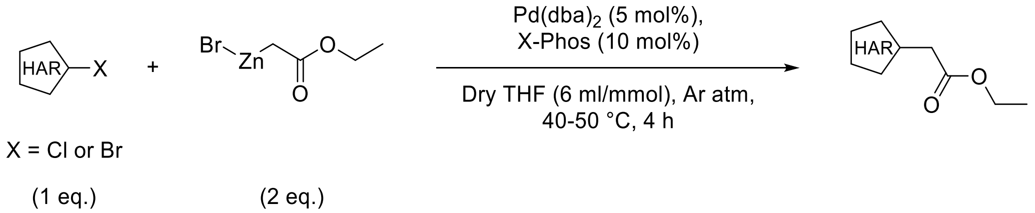

$Pd(dba)_2$ (5 mol%), X-Phos (10 mol%) and heteroaryl halide (1 equiv) were added into a flame-dried screw-cap vial equipped with a magnetic stirring bar and covered with aluminum foil. Argon atmosphere was created inside the vial and the reagents were dissolved in dry THF (1.5 mL/mmol). Finally, a solution of the Reformatsky reagent 0.4 M in THF (2 equiv) was added with a syringe and the reaction mixture was stirred for 4 h at 45 °C. The reaction was monitored by LCMS. After completion, the reaction mixture was diluted with EtOAc and washed with saturated aq. NH_4Cl. The aqueous phase was extracted one more time with EtOAc, then the organic phases were merged, dried over Na_2SO_4 and concentrated under reduced pressure. The crude was purified by preparative HPLC or FCC.

Procedure C (in-flow procedure)

1) Preparation of the flow setup

For the system set up an HPLC pump was used to pump the ethyl 2-bromoacetate solution through the glass column filled with zinc metal. A syringe pump was then used for the solution containing the aryl halide and the catalytic system in THF. The two outcome tubes were connected with a Y-mixer before entering the photoreactor (20 mL coil) at 40 - 50 °C with blue LEDs 457 nm.

a)

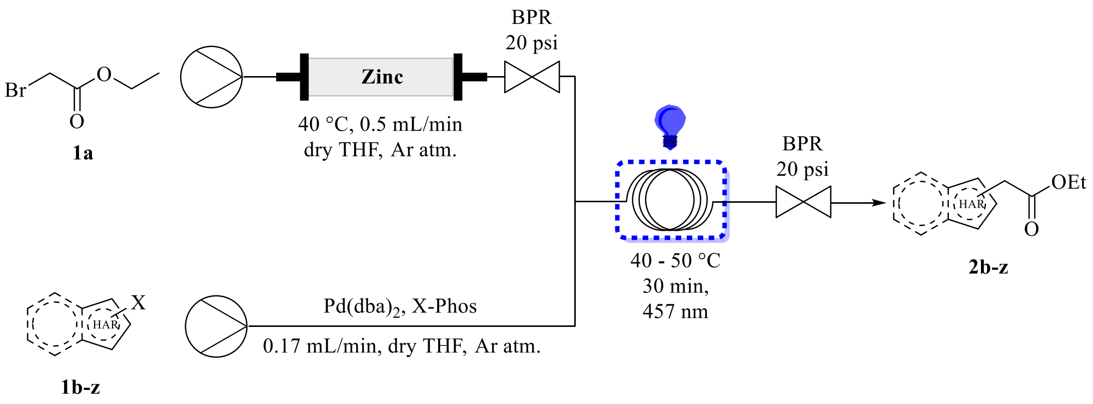

b)

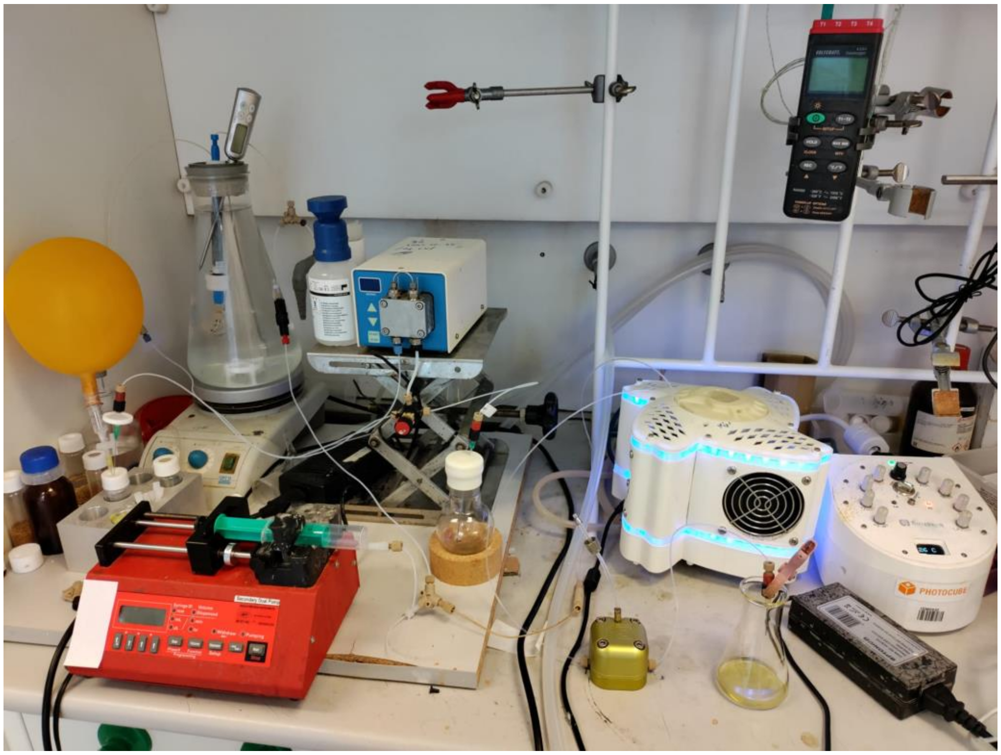

Figure S4. Flow setup: a) illustrative scheme; b) Picture of the flow system with the PhotoCube

2) Flow procedure:

Before running the flow experiment, the Zn column was activated following a literature procedure [4].

2 stock solutions were prepared:

Solution A: ethyl bromozinc acetate 0.5 M in dry THF

Solution B: heteroaryl halide 0.6 M in THF with $Pd(dba)_2$ (5 mol %) and XPhos (10 mol %) in a flame-dried screw-cap vial under argon atmosphere.

50 mL of Solution A were injected through the zinc column with a flow rate of 0.5 mL/min where the organozinc was formed with a final concentration monitored with iodine titration. The outcome was mixed with an appropriate volume of Solution B (0.17 mL/min) through a Y-mixer before entering the photoreactor in a 20 mL loop with a flow rate of 0.67 mL/min where the mixture was heated at 40–50 °C and irradiated with 457 nm LEDs for 30 minutes. The volume of Solution B is calculated according to the concentration of the Reformatsky reagent coming from the column. The outcoming solution was collected in a scaffold vial under argon atmosphere. The reaction mixture was then diluted with EtOAc and washed with saturated aq. NH_4Cl. The aqueous phase was extracted one more time with EtOAc and the organic phases were merged, dried over Na_2SO_4 and concentrated under reduced pressure. The crude was purified by FCC.

Ethyl 2-(1,3-thiazol-2-yl)acetate (2b)

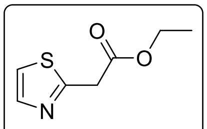

2b

Compound **2b** was prepared from 2-chlorothiazole following the general procedure. The crude was purified by preparative HPLC to afford the product as a pale-yellow oil.

Procedure A: Starting material: 1000 mg, 8.4 mmol
Product: 632 mg, 3.7 mmol, 44% yield.

Procedure C: Reformatsky reagent concentration: 0.44 M
Solution B: 440 mg, 3.7 mmol, 5.8 mL (0.64 M in dry THF)
Product: 295 mg, 1.7 mmol, 47% yield

^{1}H NMR (400 MHz, $CDCl_3$): δ 7.73 (d, J = 3.3 Hz, 1H), 7.31 (d, J = 3.3 Hz, 1H), 4.22 (q, J = 7.1 Hz, 2H), 4.07 (s, 2H), 1.28 (t, J = 7.1 Hz, 3H).

^{1}H NMR data matches the one reported in the literature [5].

^{13}C NMR (126 MHz, $CDCl_3$): δ 169.11, 161.83, 142.46, 120.09, 61.70, 38.86, 14.22.

MS–ESI (m/z): $[M+H]^+$ 172.2

Ethyl 2-(4-bromo-1,3-thiazol-2-yl)acetate (2c)

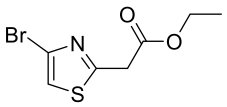

2c

Compound **2c** was prepared from 2,4-dibromothiazole (680 mg, 2.8 mmol) following Procedure A. The crude was purified by preparative HPLC using a C18 5 µm column to afford the product as a brown oil (433 mg, 1.7 mmol, 62% yield).

^{1}H NMR (400 MHz, $CDCl_3$) δ 7.20 (s, 1H), 4.21 (q, J = 7.1 Hz, 2H), 4.04 (s, 2H), 1.27 (t, J = 7.1 Hz, 3H).

^{13}C NMR (101 MHz, $CDCl_3$): δ 168.61, 163.08, 124.52, 118.10, 61.89, 38.66, 14.17.

MS–ESI (m/z): $[M+H]^+$ 250.1

HRMS: m/z calcd. for $C_7H_9{}^{79}BrNO_2S$ $[M+H]^+$: 249.9532; found 249.9530.

Ethyl 2-(5-chloro-1,3-thiazol-2-yl)acetate (2d)

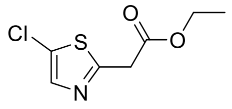

2d

Compound **2d** was prepared from 2-bromo-5-chlorothiazole (595 mg, 3.0 mmol) following Procedure A. The crude was purified by preparative HPLC using a C18 5 µm column to afford compound the product as a pale-yellow oil (131 mg, 0.6 mmol, 21% yield).

^{1}H NMR (500 MHz, $CDCl_3$) *δ* 7.51 (s, 1H), 4.23 (q, *J* = 7.2 Hz, 2H), 3.98 (s, 2H), 1.29 (t, *J* = 7.2 Hz, 3H).

^{13}C NMR (126 MHz, $CDCl_3$) *δ* 168.72, 160.68, 140.08, 127.52, 61.92, 39.50, 14.22.

MS–ESI (m/z): $[M+H]^+$ 206.2

Ethyl 2-(1,3-thiazol-5-yl)acetate (2e)

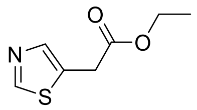

2e

Compound **2e** was prepared from 5-bromothiazole (306 mg, 1.8 mmol) following Procedure A. The crude was purified by FCC (eluent: chloroform/THF) to afford the product as a brown oil (157 mg, 0.9 mmol, 50% yield).

^{1}H NMR (400 MHz, $CDCl_3$): *δ* 8.75 (s, 1H), 7.73 (s, 1H), 4.20 (q, *J* = 7.2 Hz, 2H), 3.88 (s, 2H), 1.28 (t, *J* = 7.1 Hz, 3H).

^{13}C NMR (101 MHz, $CDCl_3$) *δ* 169.90, 153.45, 142.60, 129.96, 61.69, 32.62, 14.25.

MS–ESI (m/z): $[M+H]^+$ 172.1

2-(2-ethoxy-2-oxoethyl)thiazole-4-carboxylic acid (2f)

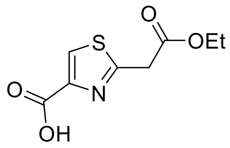

2f

Compound **2f** was prepared from 2-bromo-1,3-thiazole-4-carboxylic acid (208 mg, 1.0 mmol) following Procedure A. The crude was purified by preparative HPLC using a C18 5 µm column to afford the product as a light yellow solid (132 mg, 0.6 mmol, 61% yield).

^{1}H NMR (500 MHz, DMSO) *δ* 13.00 (br s, 1H), 8.40 (s, 1H), 4.21 (s, 2H), 4.15 (q, *J* = 7.1 Hz, 2H), 1.21 (t, *J* = 7.1 Hz, 3H).

^{1}H NMR data match the ones found in the literature [6].

^{13}C NMR (126 MHz, DMSO) *δ* 168.89, 162.54, 162.02, 146.52, 129.60, 61.02, 38.01, 13.99.

MS–ESI (m/z): $[M + H]^+$ 216.3

Ethyl 2-(1,3-benzothiazol-2-yl)acetate (2i)

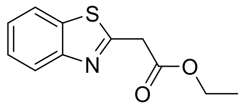

2i

Compound **2i** was prepared from 2-bromo-1,3-benzothiazole (1284 mg, 6.0 mmol) following the general procedure. The crude was purified by flash chromatography using a mixture of heptane and ethyl acetate as eluent to afford the product as a yellow liquid ().

Procedure A: Starting material: 1284 mg, 6.0 mmol
Product: 1152 mg, 5.2 mmol, 87% yield

Procedure C: Reformatsky reagent concentration: 0.39 M
Solution B: 2071 mg, 9.7 mmol, 17 mL (0.57 M in dry THF)
Product: 2060 mg, 9.3 mmol, 96% yield

^{1}H NMR (400 MHz, $CDCl_3$): δ 8.01 (d, J = 8.1 Hz, 1H), 7.88 (d, J = 8.1 Hz, 1H), 7.47 (ddd, J = 8.3, 7.2, 1.3 Hz, 1H), 7.39 (ddd, J = 8.3, 7.2, 1.3 Hz, 1H), 4.25 (q, J = 7.1 Hz, 2H), 4.17 (s, 2H), 1.30 (t, J = 7.2 Hz, 3H).

^{13}C NMR (126 MHz, $CDCl_3$): δ 168.59, 162.82, 152.89, 135.96, 126.22, 125.33, 123.15, 121.66, 61.90, 39.99, 14.25.

1H NMR data match the ones found in the literature [7].

MS–ESI (m/z): $[M+H]^+$ 222.2

Ethyl 2-(furan-3-yl)acetate (2j)

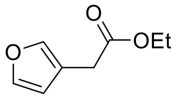

2j

Compound **2j** was prepared from 3-bromofuran (300 mg, 2 mmol) following general procedure A. The crude was purified by flash chromatography (eluent: cyclohexane/ethyl acetate) to afford the product as a yellow oil (275 mg, 1,78 mmol, 87% yield).

^{1}H NMR (400 MHz, $CDCl_3$): δ 7.39-7.37 (m, 2H), 6.38 (t, J = 1.4 Hz 1H), 4.17 (q, J = 7.1 Hz, 2H), 3.45 (s, 2H), 1.27 (t, J = 7.1 Hz, 3H).

^{1}H NMR data match the ones found in the literature [8].

^{13}C NMR data not reported

^{13}C NMR (101 MHz, $CDCl_3$): δ 171.32, 143.09, 140.48, 117.53, 111.48, 61.01, 31.05, 14.30.

GCMS m/z 154.0 (24%), 126.0 (12%), 109.0 (4%), 81.1 (50%), 53.1 (10%)

Ethyl 2-(1*H*-pyrazol-3-yl)acetate (2k)

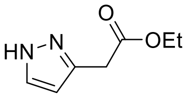

2k

Compound **2k** was prepared from 3-bromopyrazole (265 mg, 1.8 mmol) following the general procedure. The crude was purified by FCC and preparative HPLC to afford the product as a colorless oil.

Procedure A: (203 mg, 1.3 mmol, 73% yield)

Procedure B: (125 mg, 0.8 mmol, 45% yield)

^{1}**H NMR** (400 MHz, $CDCl_3$) δ 9.13 (br s, 1H), 7.54 (d, *J* = 2.0 Hz, 1H), 6.25 (d, *J* = 2.0 Hz, 1H), 4.18 (q, *J* = 7.1 Hz, 2H), 3.74 (s, 2H), 1.26 (t, *J* = 7.1 Hz, 3H).

^{13}**C NMR** (101 MHz, $CDCl_3$) δ 170.71, 142.08, 133.05, 105.19, 61.30, 33.51, 14.27.

MS–ESI (m/z): $[M+H]^+$ 155.2

^{1}H-NMR data match the ones in literature [9].

Ethyl 2-(1-benzyl-1*H*-pyrazol-4-yl)acetate (2l)

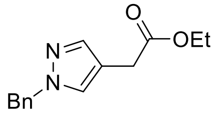

2l

Compound **2l** was prepared from 1-benzyl-4-bromo-1*H*-pyrazole (1.2 g, 5.3 mmol) following the general procedure. The crude was purified by flash chromatography (eluent: cyclohexane/ethyl acetate) to afford the product as a yellow oil.

Procedure A: (1.0 g, 4.2 mmol, 80% yield)

Procedure B: (725 mg, 3.1 mmol, 58% yield)

^{1}**H NMR** (400 MHz, $CDCl_3$): δ 7.46 (s, 1H), 7.37-7.27 (m, 4H), 7.21 (d, *J* = 6.9 Hz, 2H), 5.25 (s, 2H), 4.14 (q, *J* = 7.1 Hz, 2H), 3.46 (s, 2H), 1.24 (t, *J* = 7.1 Hz, 3H).

^{13}**C NMR** (101 MHz, $CDCl_3$): δ 171.60, 139.59, 136.60, 128.85, 128.78 128.08, 127.78, 113.75, 60.90, 56.11, 30.45, 14.22.

MS–ESI (m/z): $[M+H]^+$ 245.3

HRMS: m/z calcd. for $C_{14}H_{17}N_2O_2$ $[M-H]^+$, 245.1285; found 245.1277.

Ethyl 2-(3-(trifluoromethyl)-1*H*-pyrazol-4-yl)acetate (2m)

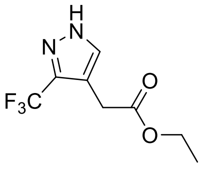

2m

Compound **2m** was prepared from 4-bromo-3-(trifluoromethyl)-1*H*-pyrazole (215 mg, 1.0 mmol) following the general procedure. The crude was purified by flash chromatography (eluent: heptane/ethyl acetate) to afford the product as a yellow oil.

Procedure A: (210 mg, 0.9 mmol, 95% yield)

Procedure B: (154 mg, 0.7 mmol, 66% yield)

^{1}**H NMR** (500 MHz, $CDCl_3$): δ 12.25 (br s, 1H), 7.72 (s, 1H), 4.19 (q, *J* = 7.1 Hz, 2H), 3.65 (s, 2H), 1.28 (t, *J* = 7.1 Hz, 3H).

^{13}**C NMR** (126 MHz, $CDCl_3$): δ 170.69 (s), 140.56 (q, *J* = 36.3 Hz), 130.73 (s), 121.92 (q, *J* = 269.0 Hz), 112.15 (s), 61.41 (s), 29.13 (s), 14.22 (s).

MS–ESI (m/z): $[M-H]^-$ 221.1

HRMS: m/z calcd. for $C_8H_8F_3N_2O_2^-$ 221.0543, found 221.0541

Ethyl 2-(1*H*-pyrazol-4-yl)acetate (2n)

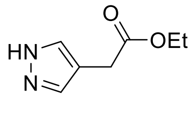

2n

Compound **2n** was prepared from 4-bromopyrazole (220 mg, 1.5 mmol) following the general procedure. The crude was purified by preparative FCC (eluent: CH_2Cl_2/MeCN) to afford the product as a white solid.

Procedure A: (110 mg, 0.7 mmol, 48% yield

Procedure B: (41 mg, 0.4 mmol, 26% yield)

^{1}H NMR (400 MHz, $CDCl_3$) δ 7.55 (s, 2H), 4.16 (q, *J* = 7.1 Hz, 2H), 3.53 (s, 2H), 1.26 (t, *J* = 7.1 Hz, 3H).

^{13}C NMR (101 MHz, $CDCl_3$) δ 171.78, 133.79, 112.98, 61.07, 30.38, 14.30.

MS–ESI (m/z): $[M+H]^+$ 155.2

Ethyl 2-(1-ethyl-1*H*-pyrazol-4-yl)acetate (2o)

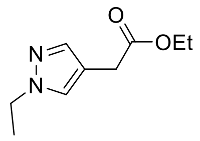

2o

Compound **2o** was prepared from 4-bromo-1-ethyl-*1H*-pyrazole (1600 mg, 9.1 mmol) following general procedure A. The crude was purified by FCC using a mixture of heptane and ethyl acetate as eluent to afford the product as a pale-yellow oil (1450 mg, 8.0 mmol, 87% yield).

^{1}H NMR (500 MHz, DMSO) δ 7.60 (s, 1H), 7.30 (s, 1H), 4.07 (q, *J* = 7.3 Hz, 2H), 4.07 (q, *J* = 7.1 Hz, 2H), 3.46 (s, 2H), 1.33 (t, *J* = 7.3 Hz, 3H), 1.18 (t, *J* = 7.1 Hz, 3H).

^{13}C NMR (126 MHz, DMSO) δ 171.25, 138.37, 128.30, 112.54, 60.14, 46.01, 29.65, 15.50, 14.06.

MS–ESI (m/z): $[M+H]^+$ 183.3

Ethyl 2-(1*H*-indazol-3-yl)acetate (2p)

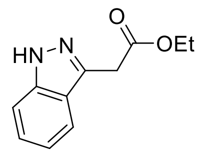

2p

Compound **2p** was prepared from 3-chloro-1*H*-indazole (568 mg, 3.7 mmol) following general procedure A. The crude was purified by preparative HPLC using a C18 5 µm column to afford the product as a pale-yellow oil (294 mg, 1.4 mmol, 39% yield).

^{1}H NMR (400 MHz, $CDCl_3$): δ 10.13 (s, 1H), 7.74 (dt, J = 8.2, 1.0 Hz, 1H), 7.45 (dt, J = 8.5, 1.0 Hz, 1H), 7.39 (ddd, J = 8.3, 6.8, 1.1 Hz, 1H), 7.17 (ddd, *J* = 8.0, 6.8, 1.0 Hz, 1H), 4.19 (q, J = 7.1 Hz, 2H), 4.04 (s, 2H), 1.25 (t, J = 7.1 Hz, 3H).

^{13}C NMR (101 MHz, $CDCl_3$): δ 170.40, 141.31, 140.26, 127.09, 122.42, 121.02, 120.48, 109.96, 61.32, 33.87, 14.32.

MS–ESI (m/z): $[M+H]^+$ 205.2

^{1}H-NMR data does not match the literature [10].

Ethyl 2-(1-methyl-1*H*-indazol-3-yl)acetate (2q)

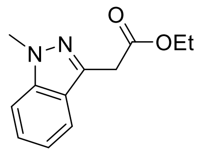

2q

Compound **2q** was prepared from 3-bromo-1-methyl-1*H*-indazole (500 mg – 2.4 mmol) following general procedure A. The crude was purified by preparative HPLC using a C18 5 µm column to afford the product as an orange liquid (250 mg, 1.2 mmol, 48%).

^{1}H NMR (400 MHz, $CDCl_3$): δ 7.69 (dt, *J* = 8.2, 1.0 Hz, 1H), 7.45 – 7.29 (m, 2H), 7.13 (ddd, *J* = 7.9, 6.3, 1.4 Hz, 1H), 4.17 (q, *J* = 7.1 Hz, 2H), 4.02 (s, 3H), 3.98 (s, 2H), 1.24 (t, *J* = 7.1 Hz, 3H).

^{13}C NMR (101 MHz, $CDCl_3$): δ 170.51, 141.12, 137.91, 126.51, 123.02, 120.66, 120.43, 109.14, 61.25, 35.49, 33.78, 14.33.

NMR data match the ones found in the literature [11].

MS–ESI (m/z): $[M+H]^+$ 218.9

Ethyl 2-(1*H*-indazol-5-yl)acetate (2r)

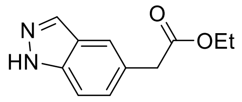

2r

Compound **2r** was prepared from 5-bromo-*1H*-indazole (750 mg, 3.8 mmol) following general procedure A. The crude was purified by flash chromatography (eluent: heptane/ethyl acetate) to afford the product as light brown crystals (634 mg, 3.1 mmol, 82% yield).

^{1}H NMR (400 MHz, $CDCl_3$): δ 10.82 (br s, 1H), 8.04 (s, 1H), 7.65 (s, 1H), 7.42 (d, *J* = 8.6 Hz, 1H), 7.31 (dd, *J* = 8.6, 1.1 Hz, 1H), 4.17 (q, *J* = 7.1 Hz, 2H), 3.72 (s, 2H), 1.26 (t, *J* = 7.1 Hz, 3H).

^{13}C NMR (101 MHz, $CDCl_3$): δ 172.25, 139.48, 134.66, 128.57, 126.90, 123.56, 121.18, 110.05, 61.08, 41.35, 14.30.

^{13}C NMR data match the ones found in the literature [12].

^{1}H NMR has not been reported before.

MS–ESI (m/z): $[M+H]^+$ 205.1

Ethyl 2-(1-methyl-1*H*-benzo[*d*]imidazol-2-yl)acetate (2t)

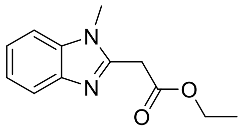

2t

Compound **2t** was prepared from 2-bromo-1-methyl-*1H*-benzo[*d*]imidazole (422 mg, 2.0 mmol) following general procedure A. The crude was purified by preparative FCC using a mixture of heptane and isopropanol as eluent to afford the product as an orange oil (336 mg, 1.5 mmol, 77% yield).

^{1}H NMR (400 MHz, $CDCl_3$): δ 7.73 (d, *J* = 7.4 Hz, 1H), 7.37 – 7.21 (m, 3H), 4.21 (q, *J* = 7.2 Hz, 2H), 4.03 (s, 2H), 3.78 (s, 3H), 1.27 (t, *J* = 7.1 Hz, 3H).

^{13}C NMR (101 MHz, $CDCl_3$): δ 168.37, 147.98, 142.54, 136.12, 122.79, 122.26, 119.77, 109.40, 61.84, 34.69, 30.39, 14.26.

MS–ESI (m/z): $[M+H]^+$ 219.2

NMR data partially match the one found in the literature [13].

Ethyl 2-(1-methyl-1*H*-imidazol-5-yl)acetate (2u)

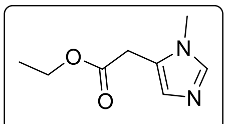

2u

Compound **2u** was prepared from 5-bromo-1-methyl-*1H*-imidazole (150 mg, 0.9 mmol) following general procedure A. The crude was purified by FCC (eluent: CH_2Cl_2/MeOH) to afford the product as a yellow oil (145 mg, 0.8 mmol, 92% yield).

^{1}H NMR (400 MHz, $CDCl_3$) δ 7.95 (s, 1H), 7.07 (s, 1H), 4.16 (q, J = 7.1 Hz, 2H), 3.67 (s, 3H), 3.62 (s, 2H), 1.26 (t, J = 7.1 Hz, 3H).

^{13}C NMR (100 MHz, $CDCl_3$) δ 168.90, 139.46, 126.74, 126.10, 61.84, 32.69, 29.89, 14.23.

MS–ESI (m/z): $[M+H]^+$ 169.2

d) Characterization of ketoesters 4j and 4r

Production of ketoester 4j

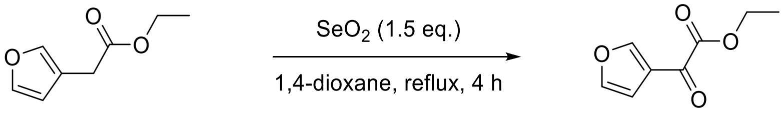

2j **4j**

Ethyl 2-(furan-3-yl)acetate (100 mg, 0.7 mmol) was dissolved in dioxane (5 mL) and the solution was heated to 60 °C. Next, SeO_2 (108 mg, 1.0 mmol, 1.5 equiv) was added and the reaction was refluxed for 4 h under vigorous stirring. After completion, the selenium powder was removed by filtration with a PTFE syringe filter. The solution was concentrated under reduced pressure to afford the desired ketoester **4j** in quantitative yield (109 mg, 0.7 mmol).

The product was analyzed by GCMS and NMR before being used for the following step without further purification.

^{1}H NMR ($CDCl_3$, 400 MHz): δ 8.53 (dd, J = 1.4, 0.8 Hz, 1H), 7.47 (t, J = 1.6 Hz, 1H), 6.90 (dd, J = 2.0, 0.8 Hz, 1H), 4.40 (q, J = 7.1 Hz, 2H), 1.41 (t, J = 7.2 Hz, 3H).

^{13}C NMR ($CDCl_3$, 100 MHz): δ 178.08, 161.28, 152.15, 144.31, 124.02, 109.34, 62.79, 14.16.

NMR spectra match the ones found in literature [14].

GCMS: m/z 168.0 (8%); 140.0 (8%); 95.0 (80%); 67.0 (4%)

Production of ketoester 4r

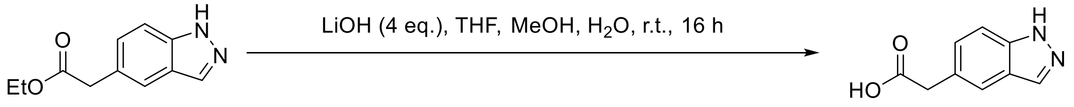

2r **3r**

Ethyl 2-(1*H*-indazol-5-yl)acetate (1.25 g, 6.1 mmol, 1.0 equiv) was dissolved in a mixture of THF (30 mL), MeOH (9 mL) and water (8 mL) and cooled down to 0 °C in an ice bath. LiOH monohydrate (1.03 g, 24.5 mmol, 4 equiv) was added to the reaction that was allowed to warm up to rt and stirred overnight. After completion, water (20 mL) was added to dissolve all the precipitate appeared during the night. The organic solvents were removed under reduced pressure and the aqueous phase was acidified to pH 2 with cc. HCl. The precipitate was collected and dried in a vacuum chamber to afford 2-(1*H*-indazol-5-yl)acetic acid (**3r**, 1.1 g, 6.0 mmol, 98% yield).

^{1}H NMR (CD_3OD, 400 MHz): δ 7.99 (d, J = 1.1 Hz, 1H), 7.66 (dd, J = 1.6, 0.8 Hz, 1H), 7.49 (dt, J = 8.7, 1.0 Hz, 1H), 7.33 (dd, J = 8.6, 1.6 Hz, 1H), 3.71 (s, 2H).

^{13}C NMR (CD_3OD_3, 100 MHz): δ 176.00, 140.86, 134.57, 129.75, 128.62, 124.52, 121.98, 111.08, 41.75.

MS–ESI (m/z): $[M+H]^+$ 177.1

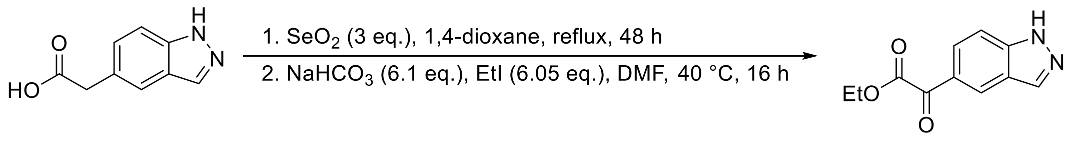

3r **4r**

Step 1. 2-(*1H*-Indazol-5-yl)acetic acid (400.0 mg, 2.27 mmol, 1.0 equiv) was dissolved in dioxane (15 mL) and the solution was heated to 60 °C. Next, SeO_2 (503.9 mg, 4.54 mmol, 2.0 equiv) was added and the reaction was refluxed for 24 h under vigorous stirring. After 24 h the reaction mixture was allowed to cool down to 60 °C and fresh SeO_2 was added to the reaction. The reaction mixture was then refluxed for another 24 h under vigorous stirring. After completion, the selenium powder was removed by filtration with an PTFE syringe filter. The dioxane solution was concentrated under reduced pressure to afford crude 2-(1*H*-indazol-5-yl)-2-oxoacetic acid.

Step 2. Crude 2-(1*H*-indazol-5-yl)-2-oxoacetic acid was dissolved in DMF (12 mL) and $NaHCO_3$ (1.14 g, 13.6 mmol, 6 equiv) was added, followed by EtI (1.1 mL, 13.7 mmol, 6.05 equiv) and the reaction mixture was stirred at 40 °C overnight. The reaction mixture was poured into cold water (100 mL) and extracted four times with EtOAc (25 mL). The combined organic phases were washed with water (100 mL) and brine (100 mL), then dried over sodium sulphate and concentrated under reduced pressure. The crude was purified with FCC using a mixture of heptane and ethyl acetate as eluent. The desired ketoester **4r** was isolated in a good overall yield of 76% over the three steps.

^{1}H NMR (400 MHz, DMSO-*d_6*): δ 13.62 (s, 1H), 8.51 (s, 1H), 8.36 (s, 1H), 7.91 (dd, *J* = 8.8, 1.6 Hz, 1H), 7.72 (d, *J* = 8.8 Hz, 1H), 4.45 (q, *J* = 7.1 Hz, 2H), 1.35 (t, *J* = 7.1 Hz, 3H).

^{13}C NMR (100 MHz, DMSO-*d_6*): δ 186.44, 164.50, 142.28, 136.55, 126.94, 125.50, 124.60, 122.64, 111.36, 62.22, 13.91.

MS–ESI (m/z): $[M\text{-}H]^-$ 217.1

e) Characterization of oximes 5b, 5i, 5t, 5j, 5r and 5l

General procedure for the oximation of compounds 2b, 2i and 2t

Ethyl heteroarylacetate (1 equiv) was dissolved in a mixture of ethanol (1 mL/mmol) and AcOH (0.7 mL/mmol) and the solution was cooled to 0 °C. A 45% wt % aq. solution of $NaNO_2$ (1.16 equiv) was added dropwise and the reaction mixture was stirred at 0 °C for 30 min. The reaction mixture was diluted three times with water and stirred at rt for 2 h. The precipitate was collected, washed with water and dried in a vacuum chamber overnight. The product was analyzed by LCMS and NMR before being used in the next step without further purification.

Ethyl 2-(hydroxyimino)-2-(thiazol-2-yl)acetate (5b)

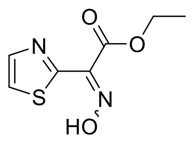

5b

Compound **5b** was prepared from compound **2b** (400 mg, 2.3 mmol) following general procedure. The compound was isolated as a brown solid without any purification (467 mg, 2.3 mmol, 100% yield). The product was isolated as a mixture of *cis* and *trans* isomers. Isomers could be separated by FCC using Hept/EtOAc as eluent.

^{1}H NMR (400 MHz, $CDCl_3$): δ 7.97 (d, J = 3.3 Hz, 1H),[a] 7.90 (d, J = 3.2 Hz, 1H),[b] 7.65 (d, J = 3.3 Hz, 1H),[a] 7.38 (d, J = 3.2 Hz, 1H),[b] 4.51 (q, J = 7.2 Hz, 2H),[b] 4.46 (q, J = 7.2 Hz, 2H),[a] 1.44 (t, J = 7.2 Hz, 3H),[a] 1.40 (t, J = 7.2 Hz, 3H).[b]

^{13}C NMR (101 MHz, $CDCl_3$): δ 163.30,[a] 161.52,[b] 159.85,[b] 156.74,[a] 147.69, [b] 144.01,[b] 140.49,[a] 138.27,[a] 123.55,[a] 120.84,[b] 62.87,[a] 62.74,[b] 14.28,[a] 14.21.[b]

[a]Major isomer, [b]Minor isomer

MS–ESI (m/z): $[M+H]^+$ 201.2

Ethyl 2-(benzo[*d*]thiazol-2-yl)-2-(hydroxyimino)acetate (5i)

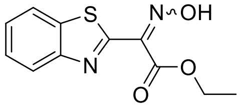

5i

Compound **5i** was prepared from compound **2i** (400 mg, 1.8 mmol) following the general procedure. The desired oxime was isolated as a yellow powder (448 mg, 1.8 mmol. 100% yield). The product was isolated as a mixture of *cis* and *trans* isomers.

^{1}H NMR (400 MHz, $CDCl_3$): δ 8.08 (d, J = 7.2 Hz, 1H),[b] 8.07 (d, J = 8.0 Hz, 1H),[a] 8.03 (d, J = 7.9 Hz, 1H),[a] 7.88 (d, J = 7.8 Hz, 1H),[b] 7.59 (dtd, J = 20.1, 7.2, 1.3 Hz, 2H),[a] 7.52 – 7.42 (m, 2H),[b] 4.54 (q, J = 7.1 Hz, 2H),[b] 4.50 (q, J = 7.2 Hz, 2H),[a] 1.47 (t, J = 7.1 Hz, 3H),[a] 1.44 (t, J = 7.2 Hz, 3H).[b]

^{13}C NMR (100 MHz, $CDCl_3$): δ 163.27,[a] 157.40,[a] 149.16,[a] 138.73,[a] 134.60,[a] 127.58,[a] 127.58,[a] 126.85,[b] 126.59,[b] 124.45,[b] 123.02,[a] 121.89,[a] 121.73,[b] 62.97,[a] 62.87,[b] 14.33,[a] 14.26.[b]

[a]Main isomer, [b]Minor isomer

MS–ESI (m/z): $[M+H]^+$ 251.2

Ethyl 2-(hydroxyimino)-2-(1-methyl-1*H*-benzo[*d*]imidazol-2-yl)acetate (5t)

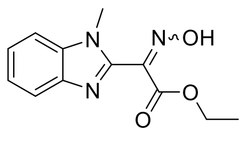

5t

Compound **5t** was prepared from compound **2t** (327 mg,1.5 mmol) following general procedure. The desired oxime was isolated as a yellow powder (367 mg, 1.5 mmol, 99% yield). The product was obtained as a mixture of *cis* and *trans* isomers and then the two isomers were separated by FCC using a mixture DCM/MeOH as eluent. The main isomer was analyzed by NMR.

^{1}H NMR (400 MHz, CD_3OD): δ 7.68 (d, *J* = 8.3 Hz, 1H), 7.56 (d, *J* = 7.7 Hz, 1H), 7.34 (dddd, *J* = 22.2, 8.3, 7.2, 1.2 Hz, 3H), 4.33 (q, *J* = 7.1 Hz, 2H), 3.72 (s, 3H), 1.30 (t, *J* = 7.0 Hz, 3H).

^{13}C NMR (100 MHz, CD_3OD): δ 163.56, 145.35, 143.12, 142.72, 136.21, 124.83, 123.96, 120.12, 111.40, 63.36, 49.00, 31.08, 14.36.

MS–ESI (m/z): $[M+H]^+$ 248.0

General procedure for the synthesis of compounds 5j and 5r

$NH_2OH{\cdot}HCl$ (2 equiv) was added to a solution of the correspondent ketoester (1 equiv) in a mixture of EtOH (1 mL/mmol) and pyridine (1 mL/mmol) and the reaction was refluxed for 55 min. After completion, the reaction was cooled to room temperature and concentrated under reduced pressure. The crude was dissolved in DCM and washed with 10 wt % aq citric acid and brine. The organic phase was then concentrated under reduced pressure to afford the desired oxime. The compound was used in the next step without further purification.

Ethyl 2-(furan-3-yl)-2-(hydroxyimino)acetate (5j)

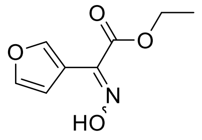

5j

Compound **5j** was prepared from **4j** (240 mg, 1.4 mmol) following general procedure. The desired oxime was isolated without any purification as a pale orange solid (258 mg, 1.4 mmol, 99% yield)

^{1}H NMR (400 MHz, $CDCl_3$): δ 10.58 (s, 1H), 8.48 (d, *J* = 0.9 Hz, 1kH), 7.46 (t, *J* = 1.7 Hz, 1H), 7.16 (d, *J* = 1.2 Hz, 1H), 4.39 (q, *J* = 7.1 Hz, 2H), 1.42 (t, *J* = 7.1 Hz, 3H).

^{13}C NMR (100 MHz, $CDCl_3$): δ 163.14, 148.13, 142.19, 141.52, 114.05, 110.99, 62.25, 14.19.

MS–ESI (m/z): $[M+H]^+$ 184.2

Ethyl 2-(hydroxyimino)-2-(1*H*-indazol-5-yl)acetate (5r)

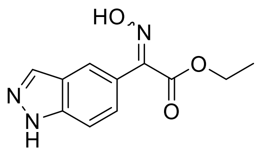

5r

Compound **5r** was prepared from compound **4r** (280 mg, 1.3 mmol) following general procedure. The desired oxime was isolated without any purification as a white solid (290 mg, 1.3 mmol, 97% yield). The product was isolated as a mixture of *cis* and *trans* isomers.

^{1}H NMR (400 MHz, CD_3OD): δ 8.10[b] (d, *J* = 1.4 Hz, 1H), 8.09[a] (d, *J* = 1.1 Hz, 1H), 7.95[b] (t, *J* = 1.2 Hz, 1H), 7.81[a] (t, *J* = 1.2 Hz, 1H), 7.76[a] (dd, *J* = 8.9, 1.6 Hz, 1H), 7.57[b] (d, *J* = 1.0 Hz, 1H), 7.55[a] (d, *J* = 1.0 Hz, 1H), 7.48[b] (dd, *J* = 8.8, 1.5 Hz, 1H), 4.44[a] (q, *J* = 7.2 Hz, 2H), 4.31[b] (q, *J* = 7.1 Hz, 2H), 1.39[a] (t, *J* = 7.2 Hz, 4H), 1.31[b] (t, *J* = 7.1 Hz, 3H).

^{13}C NMR (100 MHz, CD_3OD): δ 166.27[a], 166.13[b], 152.38[a], 150.77[b], 135.72[a], 129.03[a], 125.42[b], 125.15[a], 124.13[b], 123.79[b], 123.67[a], 120.51[a], 111.86[a], 110.42[b], 62.83k[b], 62.73[a], 14.50[a], 14.40[b].

[a]Main isomer, [b]Minor isomer

MS–ESI (m/z): $[M+H]^+$ 233.9

Procedure for the production of ethyl 2-(1-benzyl-1*H*-pyrazol-4-yl)-2-(hydroxyimino)acetate (5l)

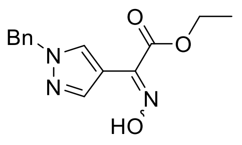

5l

A solution of NaOEt 21% wt in EtOH (2.7 mL, 6.6 mmol) was added to a solution of ethyl 2-(1-benzyl-1*H*-pyrazol-4-yl)acetate **2l** (1.0 g, 4.1 mmol) in EtOH (5 mL). t-BuONO (0.6 mL, 4.5 mmol) was added tot he mixture and the reaction was stirred at 40 °C for 20 h. After reaction time completion, the reaction mixture was concentrated under reduced pressure and diluted with water (20 mL). The pH was ad*J*usted to 5 with HCl(aq) 1M and the aqueous suspension was extracted with EtOAc (5 × 15 mL). The merged organic phase was dried over $MgSO_4$ and concentrated under reduced pressure. The crude was purified by FCC (eluent: Heptane/EtOAc) to afford the desired oxime **5l**.

^{1}H NMR (400 MHz, $CDCl_3$): δ 10.55 (s, 1H), 8.37 (d, *J* = 5.9 Hz, 2H), 7.39 – 7.27 (m, 3H), 7.27 – 7.23 (m, 2H), 5.35 (s, 2H), 4.36 (q, *J* = 7.2 Hz, 2H), 1.37 (t, *J* = 7.1 Hz, 3H).

^{13}C NMR (100 MHz, $CDCl_3$): δ 163.47, 142.02, 141.32, 135.85, 133.18, 129.05, 128.45, 127.99, 110.65, 62.13, 56.37, 14.20.

MS–ESI (m/z): $[M+H]^+$ 274.3

f) Characterization of the amino esters

Synthesis of the amino esters

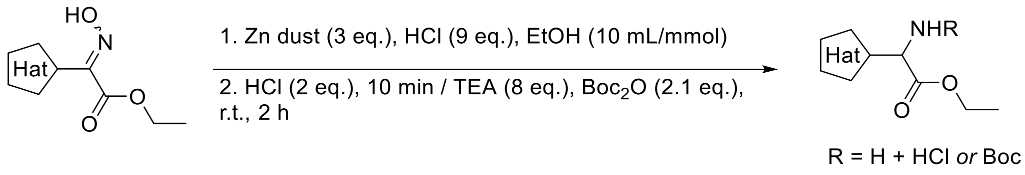

Scheme S1. General scheme for the reduction/protection step.

Ethyl 2-((*tert*-butoxycarbonyl)amino)-2-(thiazol-2-yl)acetate (6b)

Compound **6b** was obtained by reduction and then protection of compound **5b** according to the general scheme.

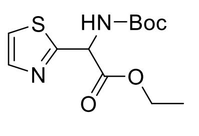

6b

A solution of oxime **5b** (200 mg, 1 mmol, 1 equiv) in EtOH (10 mL) in a 20 mL scaffold vial was cooled at −15 °C with a cooling bath. Then, HCl (9 mmol, 2.3 mL, 4 M in dioxane, 9 equiv) and zinc dust (196 mg, 3 mmol, 3 equiv) were added. The reaction mixture was stirred for 1 h at −15 °C under argon atmosphere and then at rt for 3 h. The mixture was then filtered into another scaffold vial equipped with a magnetic stir bar containing TEA (1.1 mL, 8.0 mmol, 8 equiv) and Boc_2O (689 mg, 3 mmol, 3 equiv) under argon atmosphere using a syringe FPTE filter. The reaction was stirred at rt for 2 h before being concentrated under reduced pressure. The crude was then purified by FCC using a mixture of heptane and ethyl acetate as eluent to afford the title compound as a pale-yellow oil (160 mg, 0.6 mmol, 56% yield)

^{1}H NMR ($CDCl_3$, 400 MHz): δ 7.73 (d, J = 3.2 Hz, 1H), 7.32 (d, J = 3.2 Hz, 1H), 5.97 (d, J = 8.0 Hz, 1H), 5.68 (d, J = 7.9 Hz, 1H), 4.25 (qq, J = 7.1, 3.7 Hz, 2H), 1.44 (s, 9H), 1.25 (t, J = 7.1 Hz, 3H).

^{13}C NMR ($CDCl_3$, 100 MHz): δ 168.57, 164.96, 154.92, 142.79, 120.34, 80.72, 62.58, 55.62, 28.35, 14.11.

HRMS: m/z calcd. for $C_{12}H_{18}N_2O_4S$ 287.1060; found 287.1060

Ethyl 2-amino-2-(benzo[d]thiazol-2-yl)acetate hydrochloride (6i)

Compound **6i** was obtained by reduction of compound **5i** according to the general scheme.

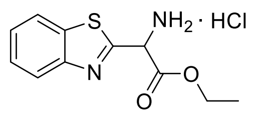

6i

A solution of oxime **5i** (200 mg, 0.8 mmol, 1 equiv) in EtOH (8 mL) in a 20 mL scaffold vial was cooled at −20 °C with a cooling bath. Then, HCl (7.2 mmol, 1.8 mL, 4 M in dioxane, 9 equiv) and zinc dust (157 mg, 2.4 mmol, 3 equiv) were added. The reaction was stirred for 1 h at −20 °C under argon atmosphere and then at rt for 4 h. The reaction mixture was filtered under argon by a syringe using a FPTE filter in order to remove the zinc and acidified with 1 equiv of conc. HCl. The mixture was stirred at rt for 10 minutes and concentrated under vacuum to afford the title compound in a good yield (140 mg, 0.6 mmol, 74% yield)

^{1}H NMR (DMSO-d_6, 400 MHz): δ 9.33 (s, 3H), 8.23 (ddd, J = 7.9, 1.5, 0.8 Hz, 1H), 8.09 (ddd, J = 8.1, 1.4, 0.7 Hz, 1H), 7.59 (dddd, J = 21.6, 8.4, 7.3, 1.3 Hz, 1H), 6.13 (s, 1H), 4.30 (qq, J = 7.0, 3.7 Hz, 1H), 1.23 (t, J = 7.1 Hz, 1H).

^{13}C NMR (DMSO-d_6, 100 MHz): δ 165.57, 161.07, 151.43, 135.29, 127.01, 126.40, 123.05, 122.79, 63.19, 53.56, 13.84.

MS–ESI (m/z): [M+H$^+$] 237.0

Ethyl 2-amino-2-(furan-3-yl)acetate hydrochloride (6j)

Compound **6j** was obtained by reduction of compound **5j** according to the general scheme.

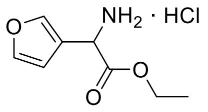

6j

A solution of oxime **5j** (220 mg, 1.2 mmol, 1 equiv) in EtOH (12 mL) in a 20 mL scaffold vial was cooled at 0 °C with an ice bath. Then, HCl (10.8 mmol, 2.7 mL, 4 M in dioxane, 9 equiv) and zinc dust (235 mg, 3.6 mmol, 3 equiv) were added. The reaction mixture was stirred for 1 h at 0 °C under argon atmosphere and then at rt for 4 h. The reaction mixture was filtered by a syringe using a FPTE filter in order to remove the zinc and it was concentrated under reduced pressure. The crude was purified by preparative HPLC using and then acidified with 1 equiv of conc. HCl to form the salt. The collected fractions after the salt formation were concentrated overnight using a lyophilizer to afford the title compound in a good yield (170 mg, 0.8 mmol, 69%)

^{1}H NMR (CD_3OD, 500 MHz): δ 7.79 (s, 1H), 7.62 (t, J = 1.8 Hz, 1H), 6.57 (d, J = 1.9 Hz, 1H), 5.22 (s, 1H), 4.31 (qd, J = 7.0, 0.7 Hz, 2H), 1.27 (t, J = 7.1 Hz, 3H).

^{13}C NMR (CD_3OD, 126 MHz): δ 169.21, 145.94, 144.00, 118.58, 109.79, 64.02, 49.82, 14.27.

MS–ESI (m/z): [M+H^{+}] 169.8

Ethyl 2-amino-2-(1-benzyl-1*H*-pyrazol-3-yl)acetate hydrochloride (6l)

Compound **6l** was obtained by reduction of compound **5l** according to the general scheme.

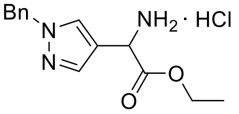

6l

A solution of oxime **5l** (340 mg, 1.2 mmol, 1 equiv) in EtOH (12 mL) in a 20 mL scaffold vial was cooled at 0 °C with an ice bath. Then, HCl (10.8 mmol, 2.7 mL, 4 M in dioxane, 9 equiv) and zinc dust (235 mg, 3.6 mmol, 3 equiv) were added. The reaction mixture was stirred for 1 h at 0 °C under argon atmosphere and then at rt for 2 h. The reaction mixture was filtered by a syringe using a FPTE filter in order to remove the zinc and it was concentrated under reduced pressure. The crude was purified by preparative HPLC using and then acidified with 1 equiv of conc. HCl to form the salt. The collected fractions after the salt formation were concentrated overnight using a lyophilizer to afford the title compound in a good yield (228 mg, 0.8 mmol, 62% yield)

^{1}H NMR (CD_3OD, 400 MHz): δ 7.92 (t, J = 0.5 Hz, 1H), 7.63 (d, J = 0.8 Hz, 1H), 7.39 – 7.23 (m, 5H), 5.37 (s, 2H), 5.22 (s, 1H), 4.30 (q, J = 7.1 Hz, 2H), 1.25 (t, J = 7.1 Hz, 3H).

^{13}C NMR (CD_3OD, 100 MHz): δ 169.42, 139.58, 137.73, 131.71, 129.84, 129.26, 128.91, 114.45, 63.98, 56.94, 14.26.

HRMS: m/z calcd. for $C_{14}H_{18}N_3O_2$ 260.1394; found 260.1395

Ethyl 2-amino-2-(1*H*-indazol-5-yl)acetate hydrochloride (6r)

Compound **6r** was obtained by reduction of compound **5r** according to the general scheme.

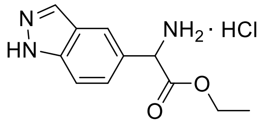

6r

A solution of oxime **5r** (327 mg, 1.4 mmol, 1 equiv) in EtOH (14 mL) in a 20 mL scaffold vial was cooled at 0 °C with an ice bath. Then, HCl (12.6 mmol, 3.2 mL, 4 M in dioxane, 9 equiv) and zinc dust (275 mg, 4.2 mmol, 3 equiv) were added. The reaction mixture was stirred for 1 h at 0 °C under argon atmosphere and then at rt for 24 h. The reaction mixture was filtered by a syringe using a FPTE filter in order to remove the zinc and it was concentrated under reduced pressure. The crude was purified by preparative HPLC using and then acidified with 1 equiv of conc. HCl to form the salt. The collected fractions after the salt formation were concentrated overnight using a lyophilizer to afford the title compound in a good yield (234 mg, 0.9 mmol, 64% yield)

^{1}H NMR (CD_3OD, 400 MHz): δ 8.14 (s, 1H), 7.95 (t, J = 1.2 Hz, 1H), 7.68 (d, J = 8.7 Hz, 1H), 7.47 (dd, J = 8.8, 1.8 Hz, 1H), 5.30 (s, 1H), 4.37 – 4.20 (m, 2H), 1.21 (t, J = 7.1 Hz, 3H).

^{13}C NMR (CD_3OD, 100 MHz): δ 169.84, 141.76, 135.16, 127.45, 126.13, 124.28, 123.00, 112.75, 63.95, 57.77, 14.24.

HRMS: m/z calcd. for $C_{11}H_{14}N_3O_2$ 220.1081; found 220.1079

Ethyl 2-((*tert*-butoxycarbonyl)amino)-2-(1-methyl-1*H*-benzo[*d*]imidazol-2-yl)acetate (6t)

Compound **6t** was obtained by reduction and subsequent protection of compound **5t** according to the general scheme.

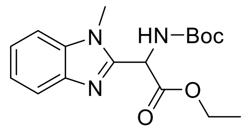

6t

A solution of oxime **5t** (200 mg, 0.8 mmol, 1 equiv) in EtOH (8 mL) in a 20 mL scaffold vial was cooled at 0 °C with an ice bath. Then, HCl (7.2 mmol, 1.8 mL, 4 M in dioxane, 9 equiv) and zinc dust (157 mg, 2.4 mmol, 3 equiv) were added. The reaction mixture was stirred for 1 h at 0 °C under argon atmosphere and then at rt for 3 h. The mixture was then filtered into another scaffold vial equipped with a magnetic stir bar containing TEA (0.9 mL, 6.5 mmol, 8 equiv) and Boc_2O (530 mg, 2.4 mmol, 3 equiv) under argon atmosphere using a syringe FPTE filter. The reaction was stirred at rt for 2 h before being concentrated under reduced pressure. The crude was then purified by FCC using a mixture heptane/ethyl acetate as eluent to afford the title compound as a white oil (170 mg, 0.5 mmol, 63% yield)

^{1}H NMR ($CDCl_3$, 400 MHz): δ 7.74 (dd, J = 7.4, 1.5 Hz, 1H), 7.41 – 7.27 (m, 3H), 6.13 (d, J = 8.4 Hz, 1H), 5.70 (d, J = 8.4 Hz, 1H), 4.31 – 4.14 (m, 2H), 3.93 (s, 3H), 1.45 (s, 9H), 1.24 (t, J = 7.1 Hz, 3H).

^{13}C NMR ($CDCl_3$, 100 MHz): δ 168.29, 155.38, 149.46, 142.27, 135.94, 123.29, 122.64, 120.10, 109.78, 80.70, 62.60, 50.95, 30.47, 28.40, 14.16.

HRMS: m/z calcd. for $C_{17}H_{24}N_3O_4$ 334.1761; Found 334.1758

g) Validation on-DNA

DNA-COOH (7a) preparation.

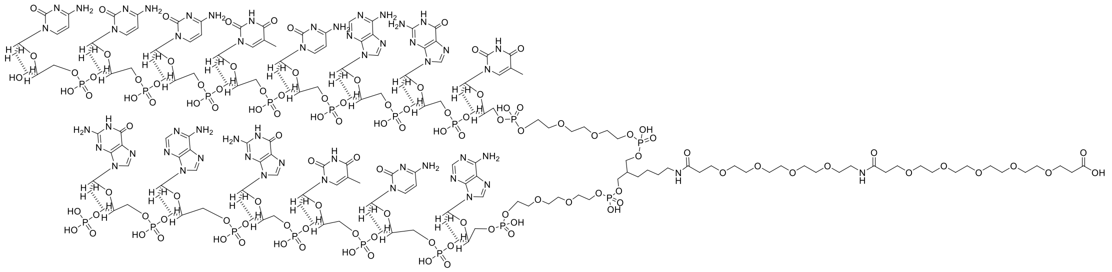

The synthesis of **7a** was performed by adapting a literature protocol [1]

Headpiece-AOP (500 nmol, 50 µL, 10 mM in water, 1 equiv) was diluted with pH 9.5 borate buffer (450 µL, 500 mM in water), then 4,7,10,13,16-pentaoxanonadecanedioic acid (20 000 nmol, 200 µL, 200 mM in DMF, 40 equiv) and DMTMM (20 000 nmol, 200 µL, 200 mM in water, 40 equiv) were added. The reaction was allowed to proceed at room temperature for 16 h. Following completion, as monitored by UPLC-MS, the reaction was precipitated by the addition of 100 µL of 5 M NaCl aqueous solution and 4 mL of cold ethanol. The sample was placed in dry ice for 60 minutes and centrifugated for 30 minutes. The supernatant was removed and the resulting DNA pellet was dried on a lyophilizer for 1 h. The dried pellet was then redissolved in 300 µL of water and three cycles of spin-filtration-dilution with water were done using a centrifugal filter. The remaining solution was then diluted to 0.5 mL with water and the optical density of the sample was measured to assess the final concentration to 1 mM. The intended product **7a** was formed in 91% yield (based on OD and HPLC-MS).

General procedure for the synthesis of the DNA-tagged amino esters

The synthesis of the DNA-tagged amino esters was performed by adapting a literature protocol [15].

Carboxylic acid-terminated DNA **7a** (20 nmol, 20 µL, 1 mM water, 1 equiv) was diluted with pH 5.8 MES buffer (10 000 nmol, 20 µL, 500 mM in water, 500 equiv). Water (17 µL) and MeCN (25 µL) were added, followed by the amino ester building block (2 000 nmol, 10 µL, 200 mM in MeCN, 100 equiv) and DMTMM (2 000 nmol, 10 µL, 200 mM in water, 100 equiv). The solution was vortexed and allowed to incubate overnight at room temperature. After completion, as monitored by UPLC-MS, the reaction was precipitated by the addition of 10 µL of 5 M NaCl aqueous solution and 400 µL of cold ethanol. The sample was placed in dry ice for 60 minutes and centrifugated for 30 minutes. The supernatant was removed and the DNA pellet was re-dissolved in H_2O (100 µL). Three cycles of spin-filtration-dilution with water were done using a centrifugal filter. The final solution was dried in the lyophilizer overnight. Finally, the dried DNA pellet was redissolved in water (20 µL). An aliquot (1 µL) was taken, diluted with H_2O (100 µL) and analyzed via UPLC-MS. The intended compounds were used for the following reactions without further purification.

Synthesis of DNA-tagged amino ester 8j

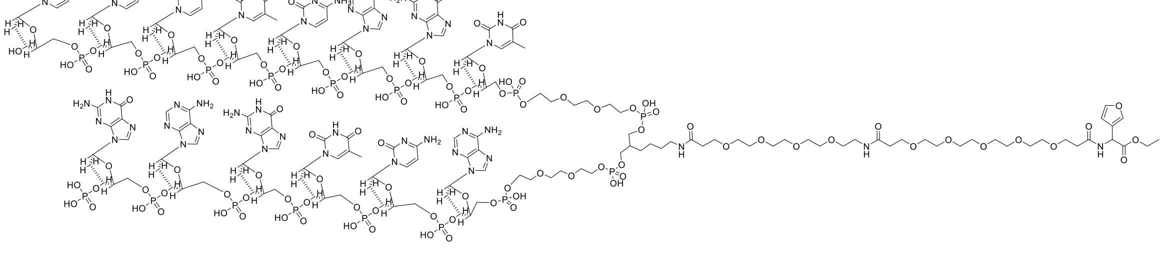

The title compound was synthesized according to the general procedure from ethyl 2-amino-2-(furan-3-yl)acetate hydrochloride (0.4 mg, 2.0 µmol). The intended product was formed in 91% yield.

m/z = 5655.16

Synthesis of DNA-tagged amino ester 8l

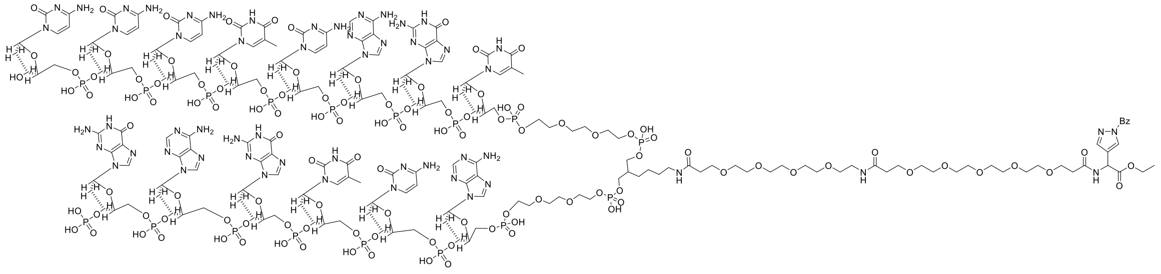

The title compound was synthesized according to the general procedure from ethyl 2-amino-2-(1-benzyl-1*H*-pyrrol-3-yl)acetate hydrochloride (0.6 mg, 2.0 µmol). The intended product was formed in 90% yield.

m/z = 5745.22

Synthesis of DNA-tagged amino ester 8r

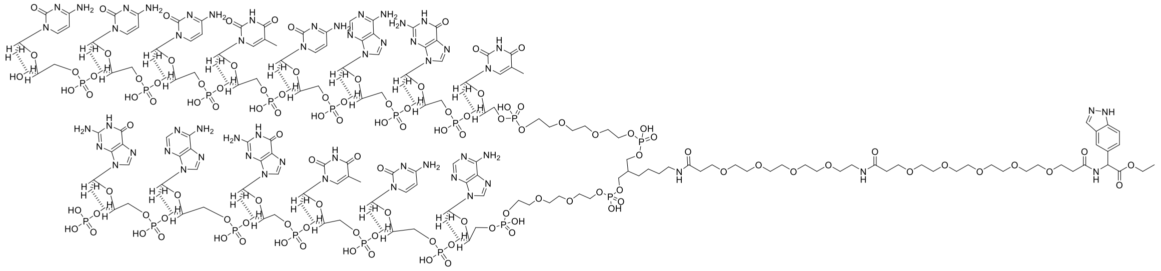

The title compound was synthesized according to the general procedure from ethyl 2-amino-2-(1*H*-indazol-5-yl)acetate hydrochloride (0.6 mg, 2.0 µmol). The intended product was formed in 91% yield.

m/z = 5705.19

Synthesis of DNA-tagged amino ester 8t

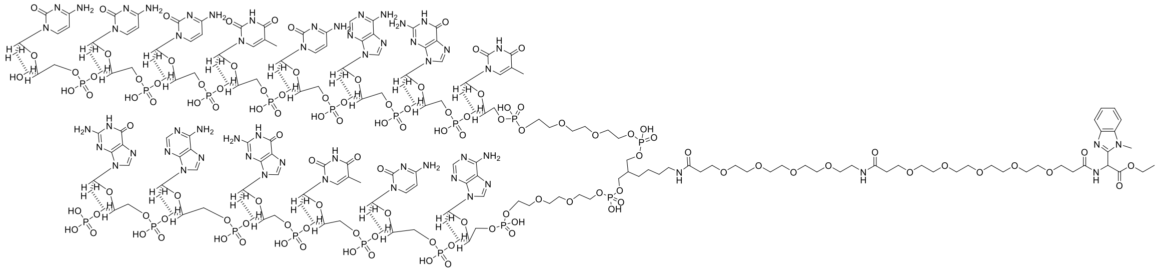

Boc deprotection of **6t**: Ethyl 2-((*tert*-butoxycarbonyl)amino)-2-(1-methyl-1*H*-benzo[*d*]imidazol-2-yl)acetate (35 mg, 0.1 mmol, 1 equiv) was dissolved in 4N HCl in dioxane (263 µL, 1.0 mmol, 10 equiv) and stirred at rt The reaction was monitored by UPLC-MS and after completion the solution was concentrated under reduced pressure to complete dryness. The crude material was used without any further purification for the on-DNA step.

The title compound was synthesized according to the general procedure from ethyl 2-amino-2-(1-methyl-*1H*-1,3-benzodiazol-2-yl)acetate hydrochloride (0.6 mg, 2.0 µmol). The intended product was formed in 75% yield.

m/z = 5719.205

General procedure for the hydrolysis of the DNA-tagged amino esters

The hydrolysis of the DNA-tagged amino esters was performed by adapting a literature protocol [16].

NaOH (2000 nmol, 20 µL, 100 mM in water, 100 equiv) was added to the DNA-tagged amino esters (20 nmol, 20 µL, 1 mM in water, 1 equiv). The solution was shaken at 400 rpm and heated at 60 °C for 2 h. Following completion, as monitored by UPLC-MS, the reaction was precipitated by the addition of 4 µL of 5 M NaCl aqueous solution and 160 µL of cold ethanol. The sample was placed in dry ice for 60 minutes and centrifugated for 30 minutes. The supernatant was removed and the DNA pellet was re-dissolved in H_2O (100 µL). Three cycles of spin-filtration-dilution with water were done using a centrifugal filter. The final solution was dried in the lyophilizer overnight. Finally, the dried DNA pellet was redissolved in water (20 µL). An aliquot (1 µL) was taken, diluted with H_2O (100 µL) and analyzed via UPLC. The intended compounds were used for the following reactions without further purification

Synthesis of DNA-tagged amino acid (9j)

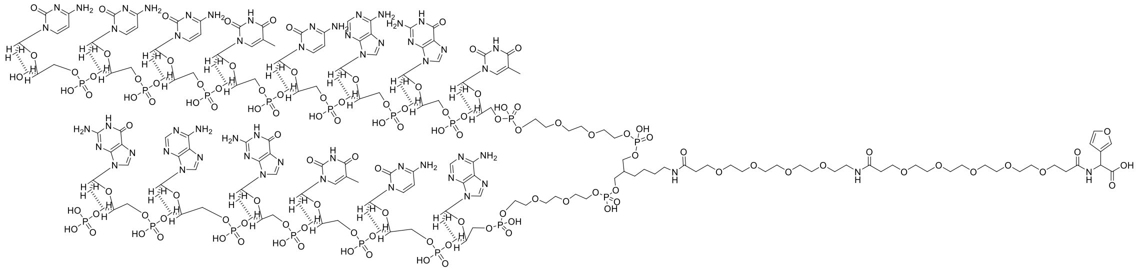

The title compound was synthesized according to the general procedure from compound **8j** (20 nmol, 20 µL, 1 mM in H_2O). The intended product was formed in 95% yield.

m/z = 5627.13

Synthesis of DNA-tagged amino acid (9l)

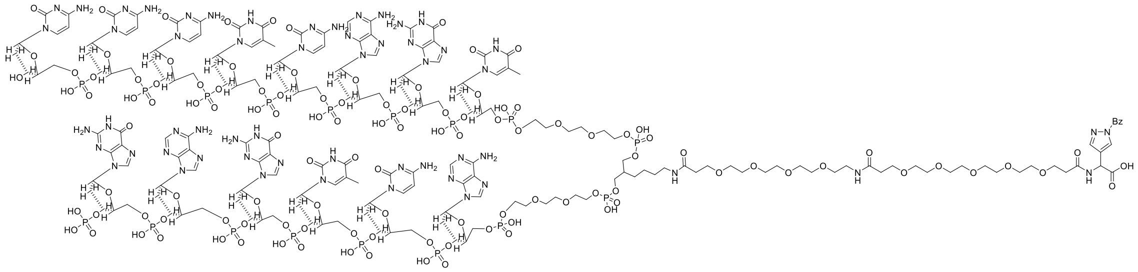

The title compound was synthesized according to the general procedure from compound **8l** (20 nmol, 20 µL, 1 mM in H_2O). The intended product was formed in 91% yield.

m/z = 5717.19

Synthesis of DNA-tagged amino acid (9r)

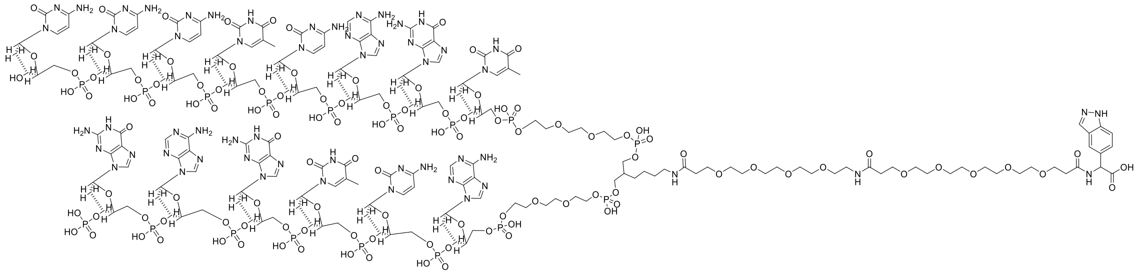

The title compound was synthesized according to the general procedure from compound **8r** (20 nmol, 20 µL, 1 mM in H_2O). The intended product was formed in 94% yield.

m/z = 5677.16

General procedure for the reverse amidation of the DNA-tagged amino acids

The second reverse amidation was performed by adapting a literature protocol [15].

DNA-tagged amino acid (20 nmol, 20 µL, 1 mM in water, 1 equiv) was diluted with pH 5.8 MES buffer (10 000 nmol, 20 µL, 500 mM in water, 500 equiv). Additional water (17 µL) and MeCN (25 µL) were added, followed by aniline (2 000 nmol, 10 µL, 200 mM in MeCN, 100 equiv) and DMTMM (2 000 nmol, 10 µL, 200 mM in water, 100 equiv). The solution was vortexed and allowed to incubate overnight at room temperature. Following completion, as monitored by UPLC-MS, the reaction was precipitated by the addition of 10 µL of 5 M NaCl aqueous solution and 400 µL of cold ethanol. The sample was placed in dry ice for 60 minutes and centrifugated for 30 minutes. The supernatant was removed and the DNA pellet was re-dissolved in H_2O (100 µL). Three cycles of spin-filtration-dilution with water were done using a centrifugal filter. The final solution was dried in the lyophilizer overnight. Finally, the dried DNA pellet was redissolved in water (20 µL). An aliquot (1 µL) was taken, diluted with H_2O (100 µL) and analyzed via UPLC.

Reverse amidation with DNA-tagged amino acid 9j (10j)

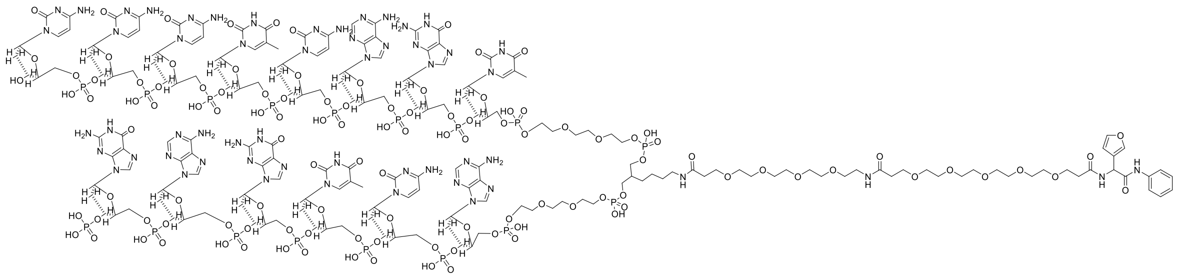

The title compound was synthesized according to the general procedure from compound **9j** (20 nmol, 20 µL, 1 mM in H_2O). The intended product was formed in 34% yield.

m/z = 5702.26

Reverse amidation with DNA-tagged amino acid 9l (10l)

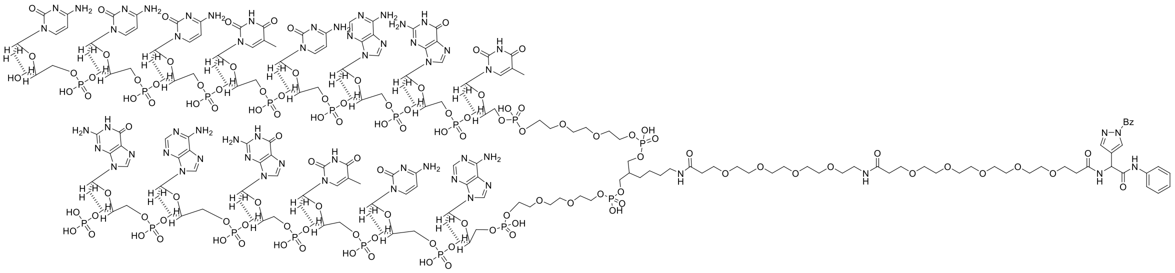

The title compound was synthesized according to the general procedure from compound **9l** (20 nmol, 20 µL, 1 mM in H_2O). The intended product was formed in 58% yield.

m/z = 5792.32

Reverse amidation with DNA-tagged amino acid 9r (10r)

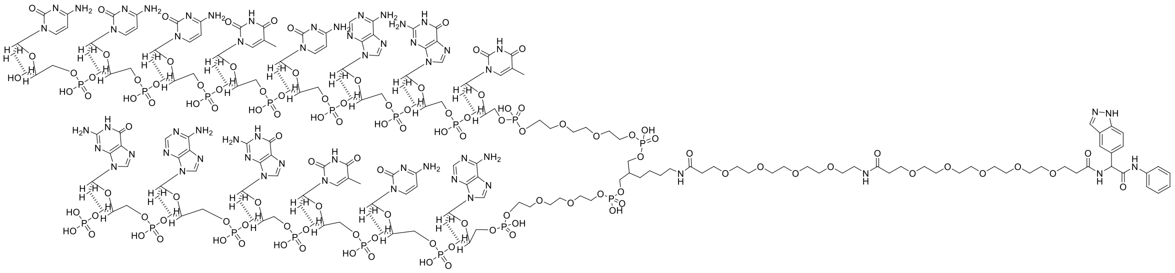

The title compound was synthesized according to the general procedure from compound **9q** (20 nmol, 20 µL, 1 mM in H_2O). The intended product was formed in 59% yield.

m/z = 5752.29

Analytical data on-DNA reactions

DNA-COOH (7a)

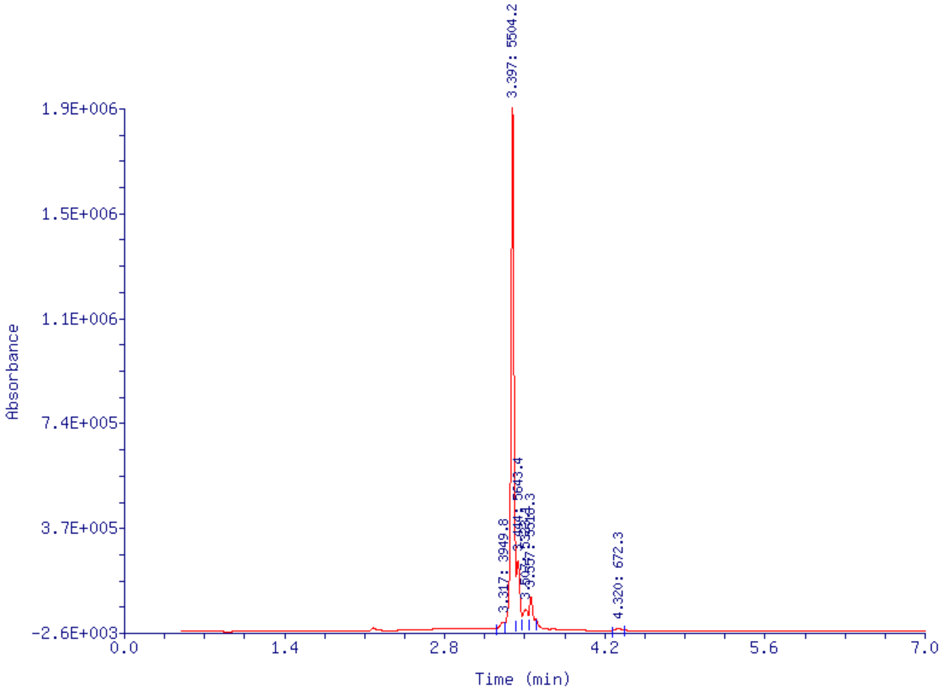

RT (min)	Base Peak Mass (Da)	Intensity	Spectral Quality	LC/UV Peak Area	LC/UV Area Percent	
3.317	3949.8	77900000	ok	3.89E+04	0.93	
3.397	5504.2	1610000000	ok	3.40E+06	81.07	**Product**
3.444	5643.4	349000000	ok	4.18E+05	9.97	**DMT adduct**
3.507	5323.1	91800000	ok	1.40E+05	3.35	
3.557	5518.3	113000000	ok	1.76E+05	4.2	
4.32	672.3	3000000	low score	2.02E+04	0.48	

Total purity: 91% (Product)

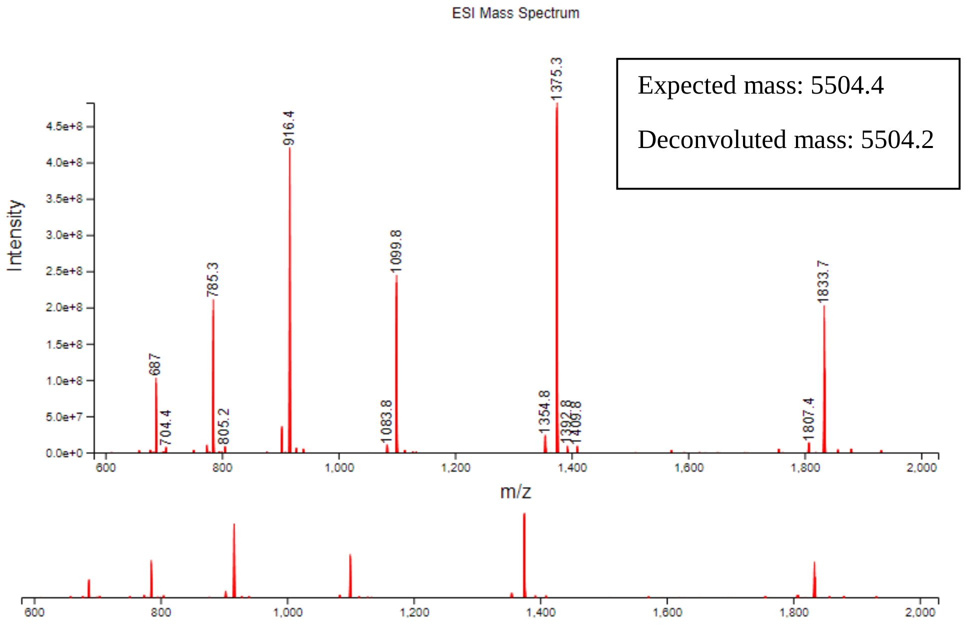

DNA-tagged amino ester (8j)

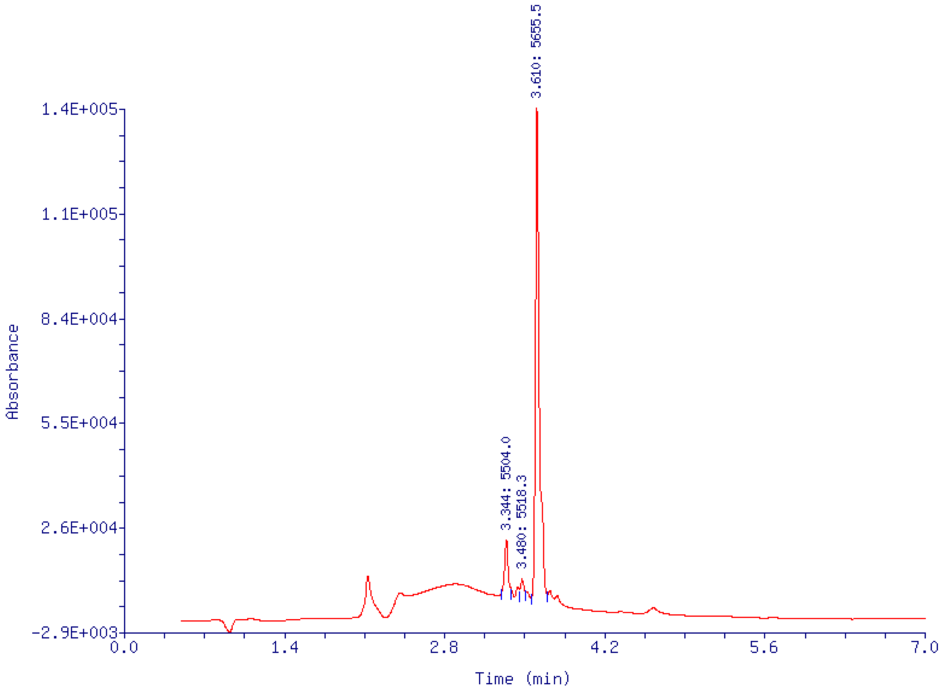

RT (min)	Base Peak Mass (Da)	Intensity	Spectral Quality	LC/UV Peak Area	LC/UV Area Percent	
3.344	5504	1.71E+07	ok	3.02E+04	8.43	
3.48	5518.3	7.85E+06	ok	8.67E+03	2.42	
3.61	5655.5	1.03E+08	ok	3.20E+05	89.16	**Product**

Purity: 89% - Normalized yield: 91%

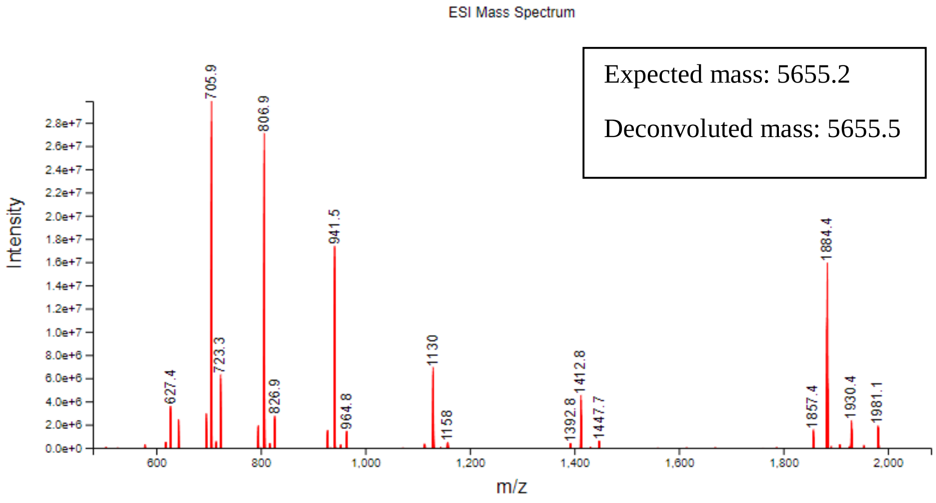

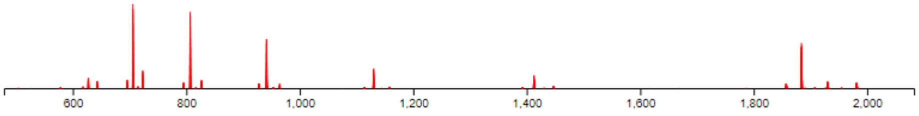

DNA-tagged amino ester (8l)

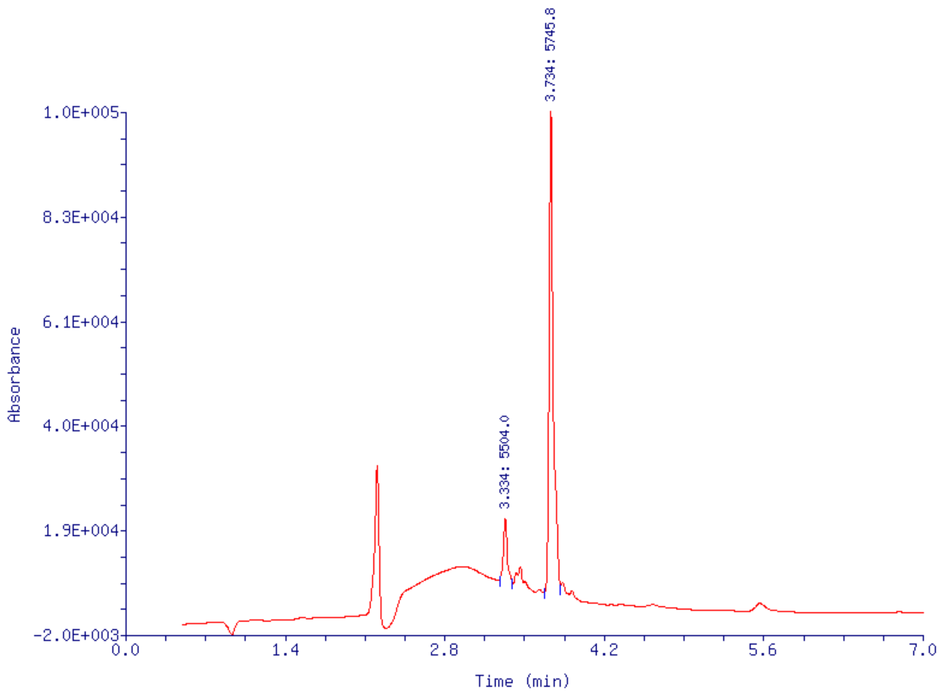

RT (min)	Base Peak Mass (Da)	Intensity	Spectral Quality	LC/UV Peak Area	LC/UV Area Percent	
3.334	5504	12200000	ok	2.89E+04	10.5	
3.734	5745.8	69600000	ok	2.47E+05	89.5	**Product**

Purity: 90% - Normalized yield: 90%

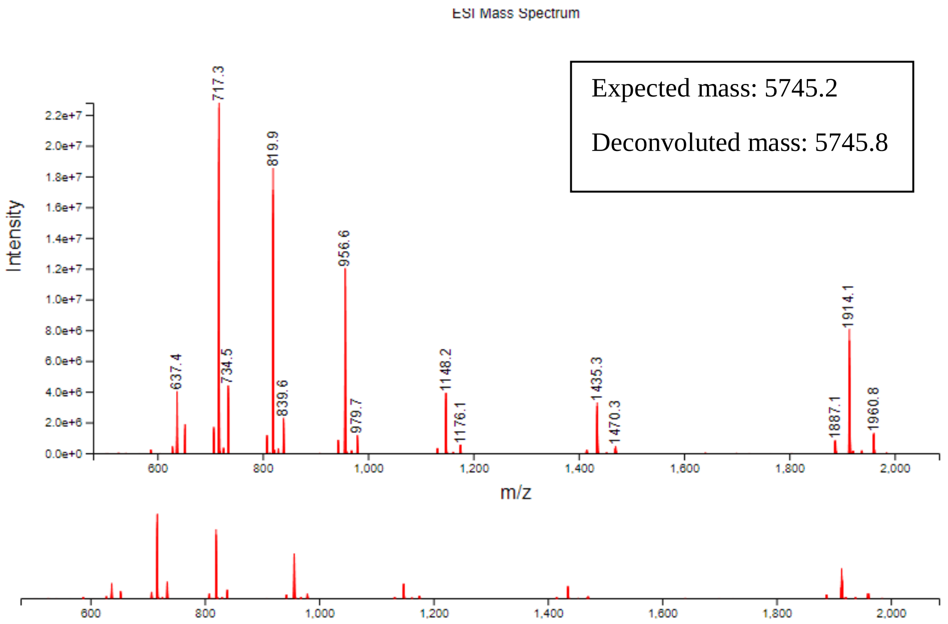

DNA-tagged amino ester (8r)

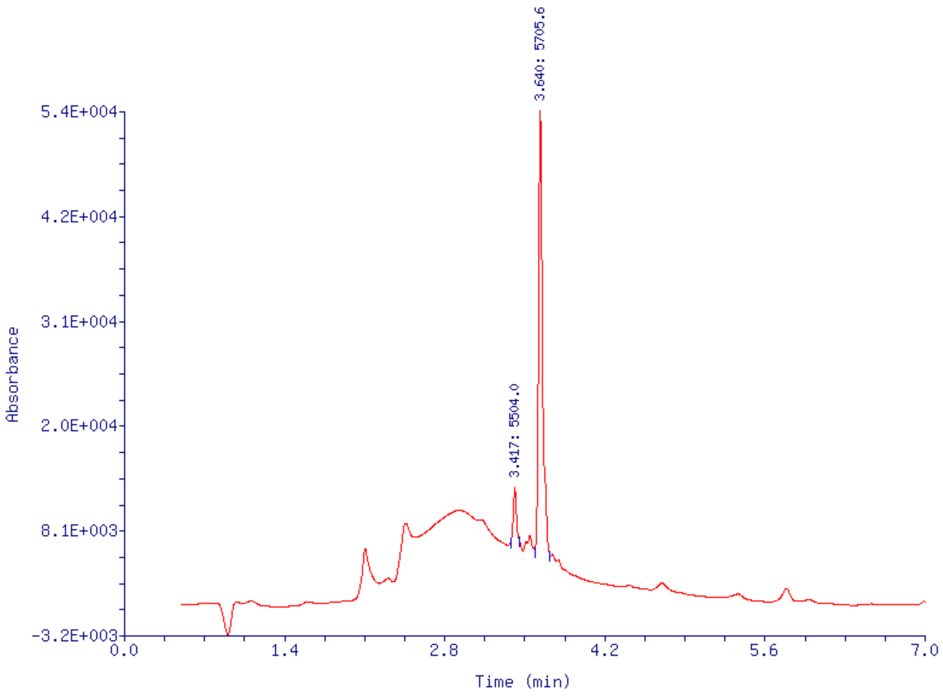

RT (min)	Base Peak Mass (Da)	Intensity	Spectral Quality	LC/UV Peak Area	LC/UV Area Percent	
3.417	5504	7.33E+06	ok	1.09E+04	8.94	
3.64	5705.6	3.26E+07	ok	1.11E+05	91.06	**Product**

Purity 91% - Yield 91%

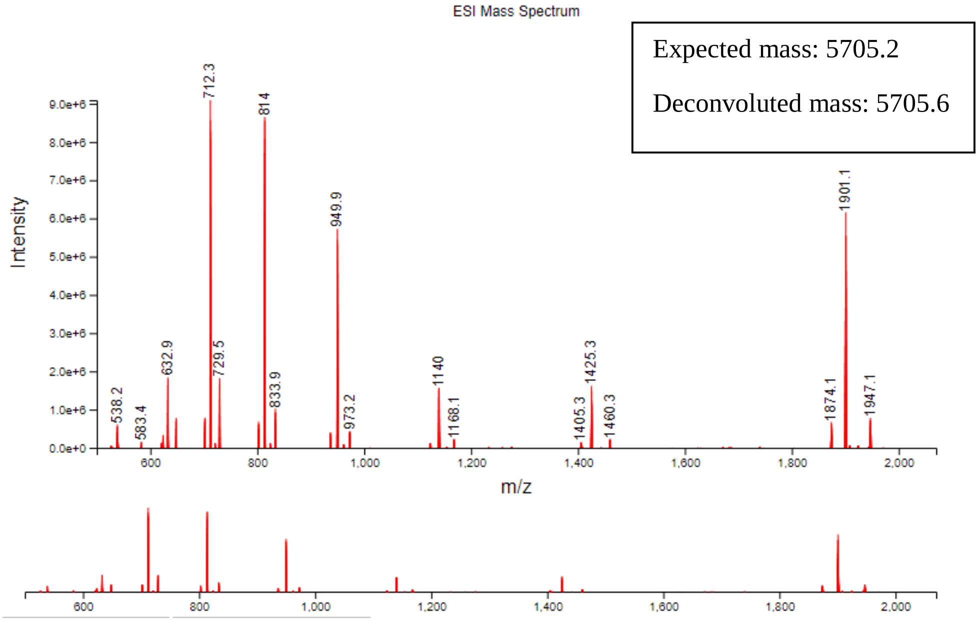

DNA-tagged amino ester (8t)

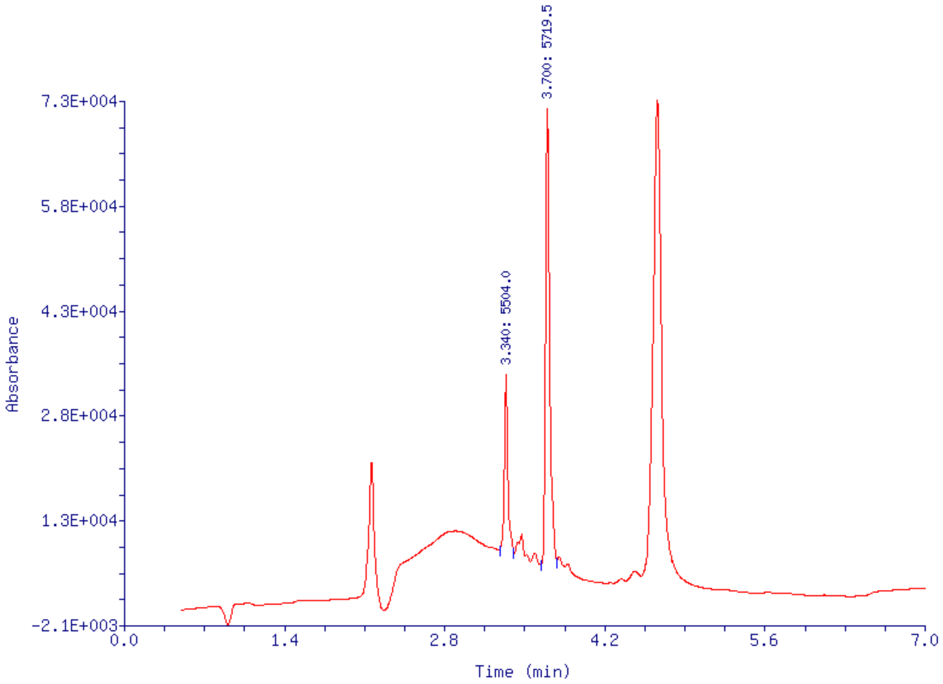

RT (min)	Base Peak Mass (Da)	Intensity	Spectral Quality	LC/UV Peak Area	LC/UV Area Percent	
3.34	5504	2.12E+07	ok	5.33E+04	25.45	
3.7	5719.5	4.99E+07	ok	1.56E+05	74.55	**Product**

Purity: 75% - Normalized yield: 75%

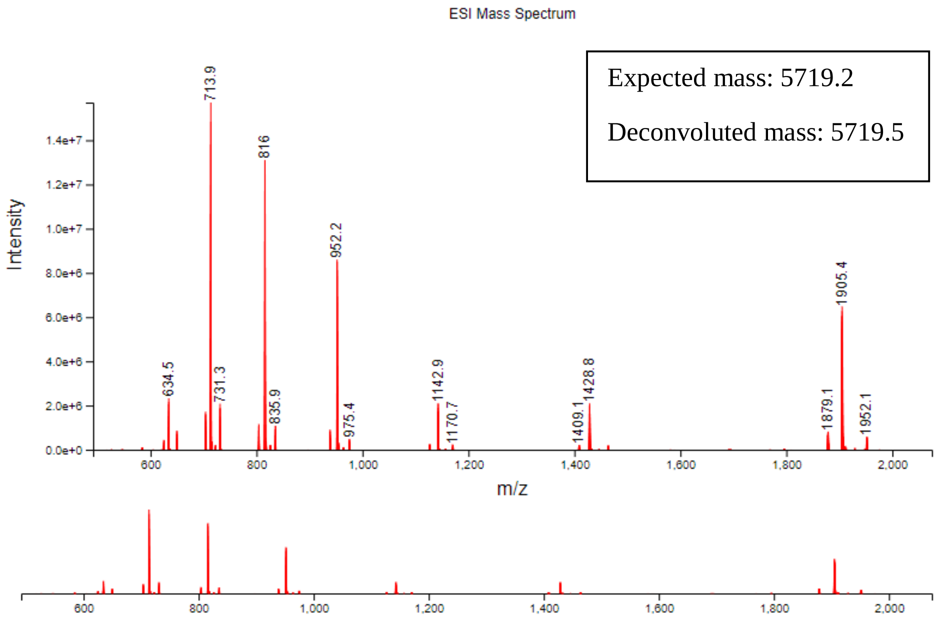

DNA-tagged amino acid (9j)

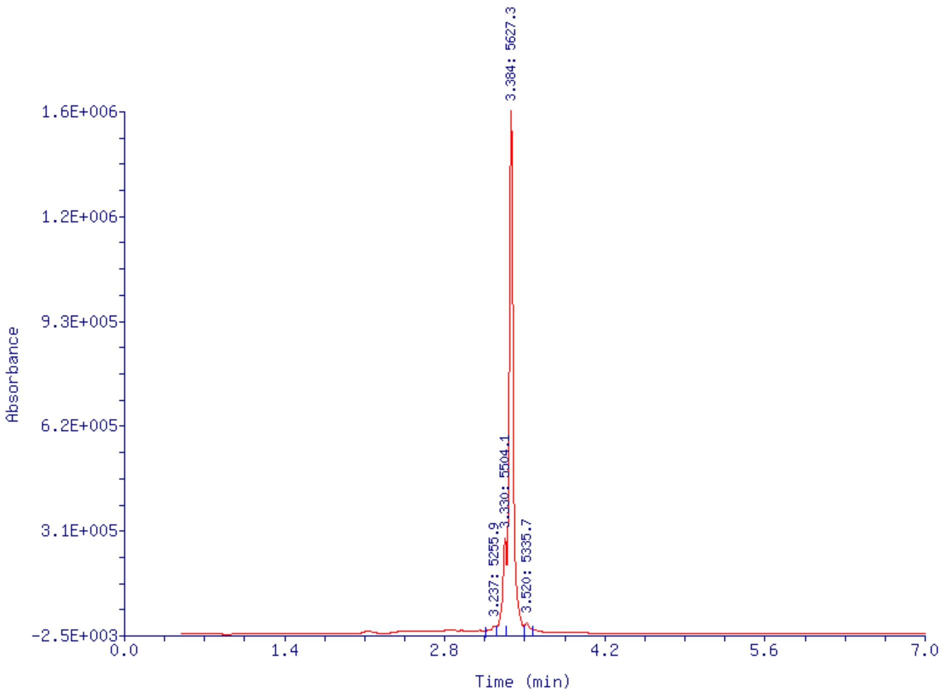

RT (min)	Base Peak Mass (Da)	Intensity	Spectral Quality	LC/UV Peak Area	LC/UV Area Percent	
3.237	5255.9	1.40E+07	ok	3.26E+04	0.74	
3.33	5504.1	1.85E+08	ok	6.09E+05	13.8	
3.384	5627.3	5.96E+08	ok	3.72E+06	84.32	**Product**
3.52	5335.7	7.61E+06	ok	5.01E+04	1.14	

Purity: 84% - Normalized yield: 95%

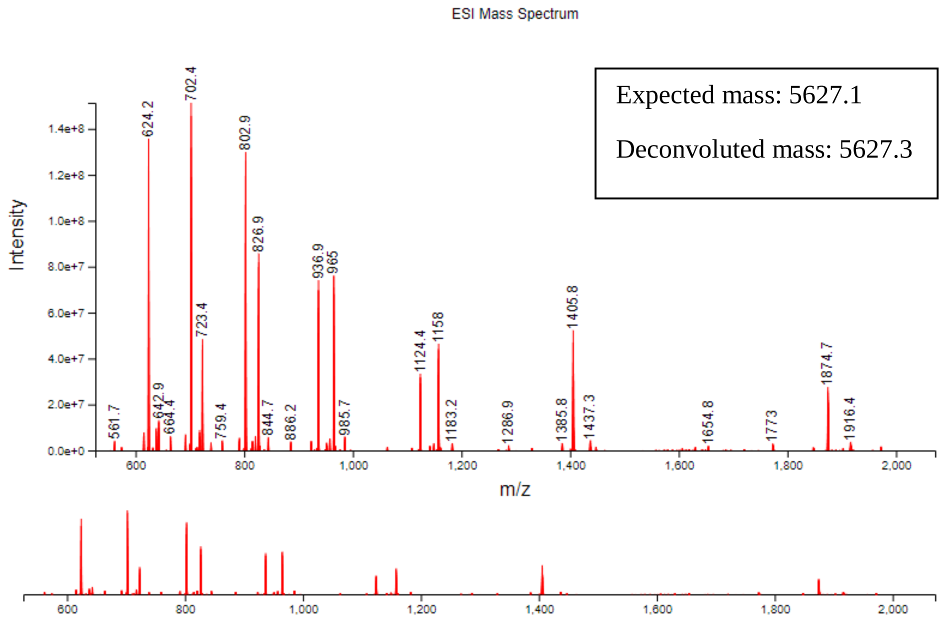

DNA-tagged amino acid 9l

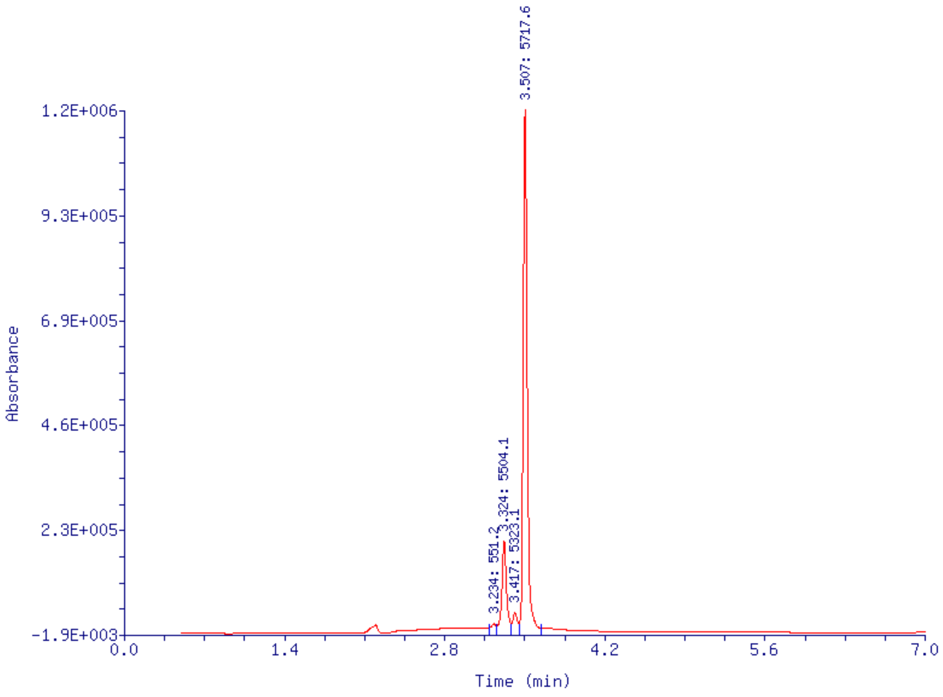

RT (min)	Base Peak Mass (Da)	Intensity	Spectral Quality	LC/UV Peak Area	LC/UV Area Percent	
3.234	551.2	2.28E+07	low score	1.77E+04	0.55	
3.324	5504.1	1.40E+08	ok	4.86E+05	14.98	
3.417	5323.1	4.07E+07	ok	8.59E+04	2.65	
3.507	5717.6	3.90E+08	ok	2.65E+06	81.83	**Product**

Purity: 81% - Normalized yield: 91%

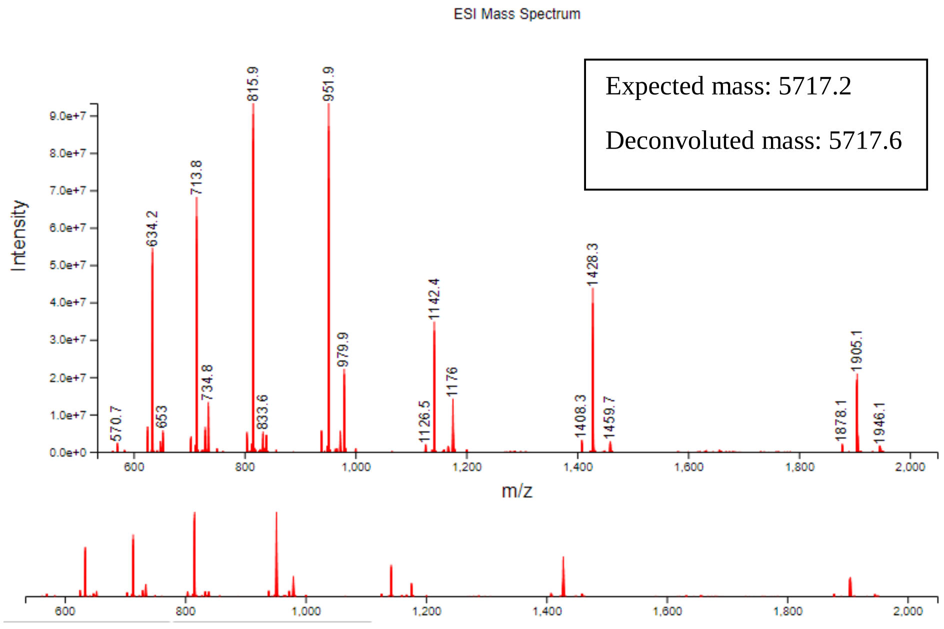

DNA-tagged amino acid (9r)

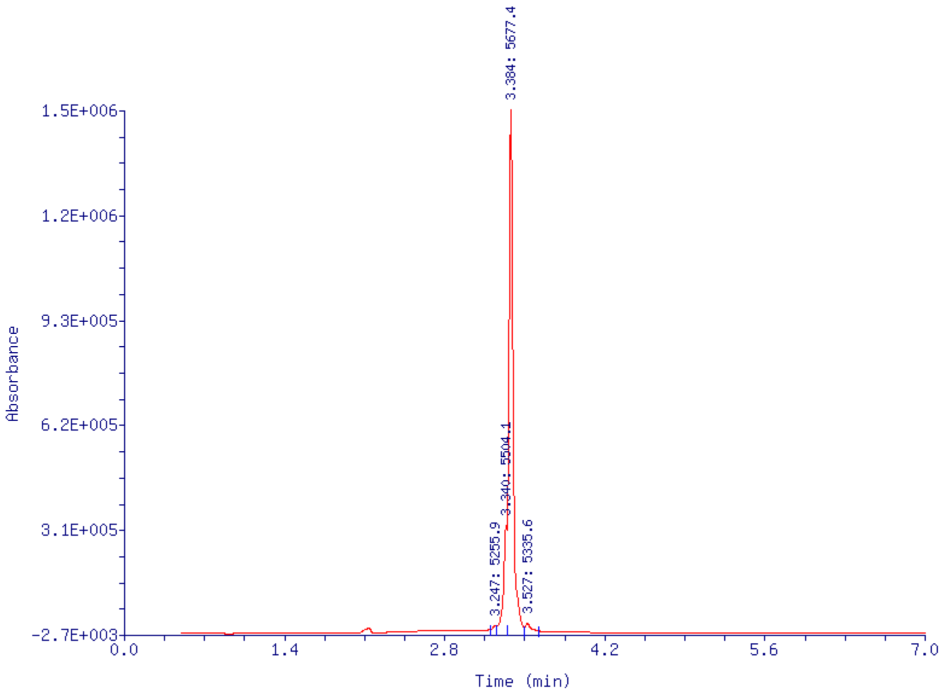

RT (min)	Base Peak Mass (Da)	Intensity	Spectral Quality	LC/UV Peak Area	LC/UV Area Percent	
3.247	5255.9	2.01E+07	ok	2.36E+04	0.54	
3.34	5504.1	1.83E+08	ok	5.69E+05	12.93	
3.384	5677.4	6.58E+08	ok	3.74E+06	85.11	**Product**
3.527	5335.6	5.80E+06	ok	6.25E+04	1.42	

Purity: 85% - Normalized yield: 94%

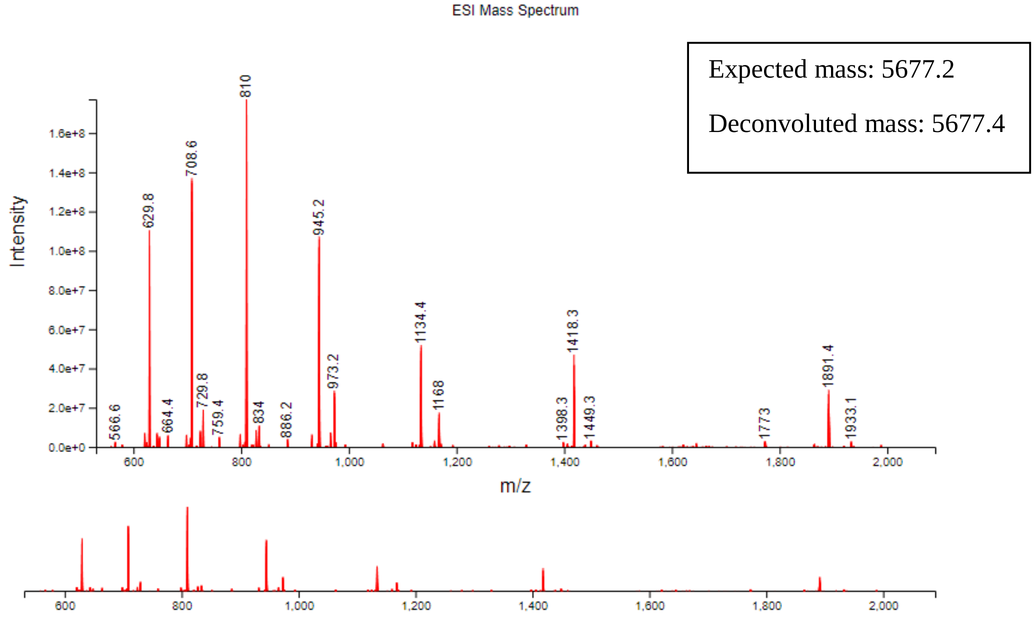

Expected mass: 5677.2

Deconvoluted mass: 5677.4

DNA-tagged compound 10j

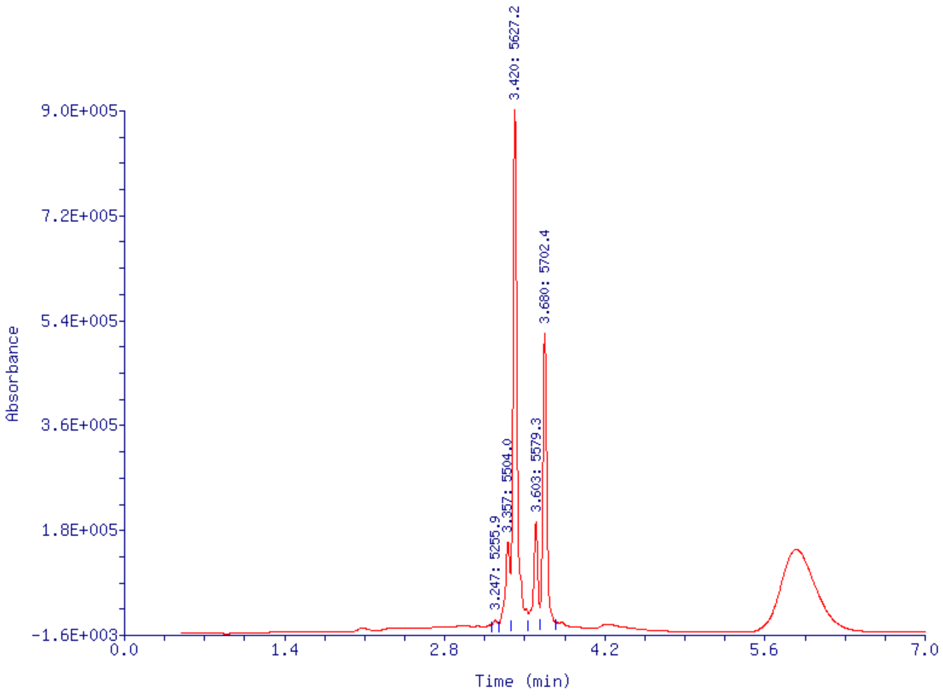

RT (min)	Base Peak Mass (Da)	Intensity	Spectral Quality	LC/UV Peak Area	LC/UV Area Percent
3.247	5255.9	1.67E+07	ok	1.82E+04	0.43
3.357	5504	8.36E+07	ok	3.86E+05	8.96
3.42	5627.2	3.49E+08	ok	2.23E+06	51.72
3.603	5579.3	8.70E+07	ok	4.52E+05	10.49
3.68	5702.4	2.54E+08	ok	1.22E+06	28.4

Purity: 28% - Normalized yield: 34%

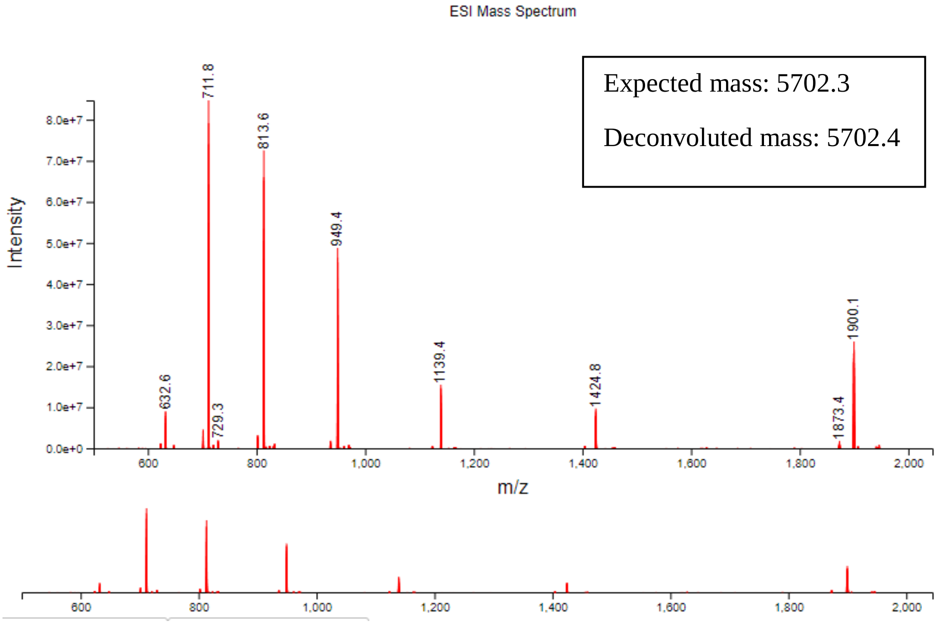

DNA-tagged compound 10l

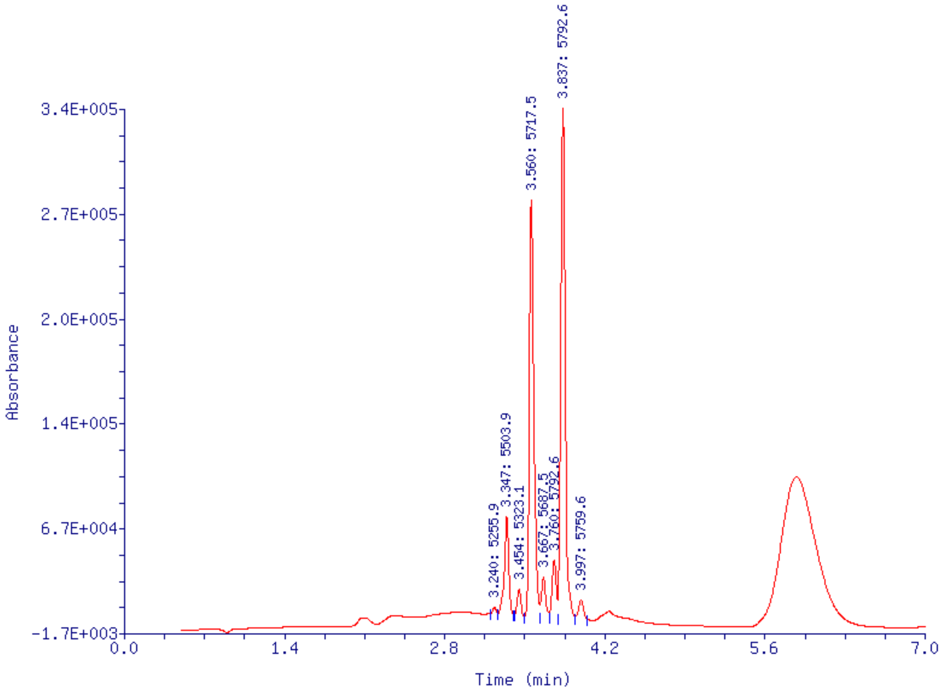

RT (min)	Base Peak Mass (Da)	Intensity	Spectral Quality	LC/UV Peak Area	LC/UV Area Percent	
3.24	5255.9	6.84E+06	ok	8.18E+03	0.4	
3.347	5503.9	3.89E+07	ok	1.70E+05	8.34	
3.454	5323.1	1.69E+07	ok	3.64E+04	1.78	
3.56	5717.5	1.27E+08	ok	7.48E+05	36.7	
3.667	5687.5	2.64E+07	ok	6.73E+04	3.3	
3.76	5792.6	4.12E+07	ok	9.05E+04	4.44	**Product**
3.837	5792.6	1.57E+08	ok	8.81E+05	43.26	
3.997	5759.6	1.32E+07	ok	3.58E+04	1.76	

Purity: 48% - Normalized yield: 59%

ESI Mass Spectrum

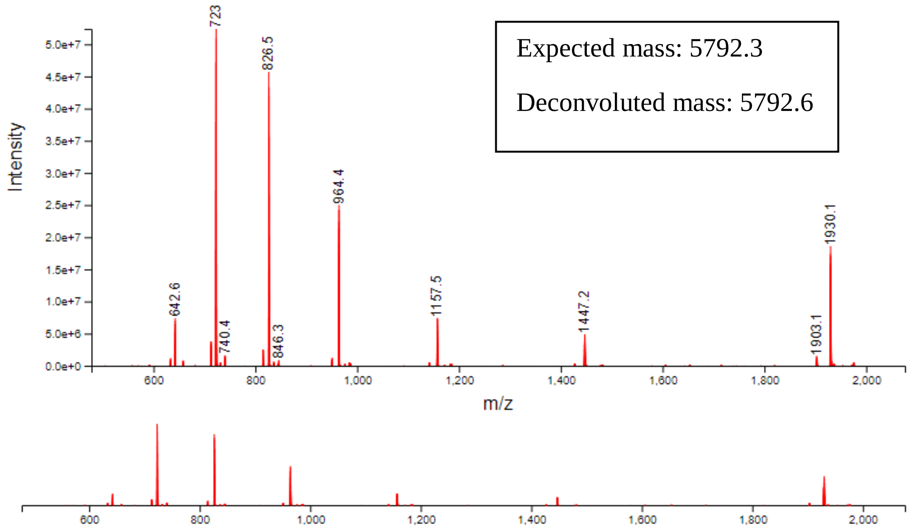

DNA-tagged compound 10r

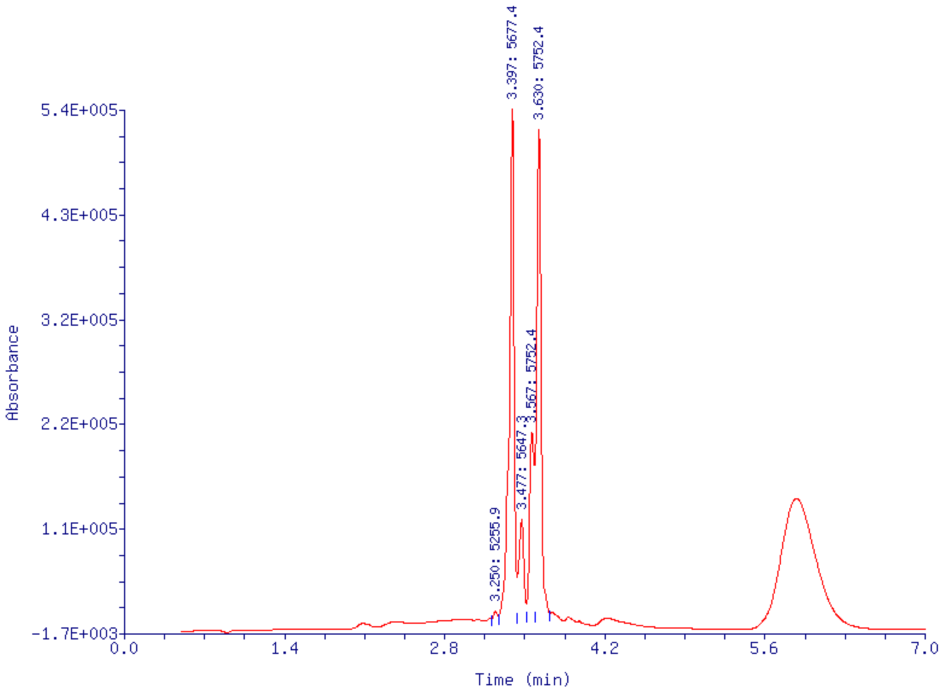

RT (min)	Base Peak Mass (Da)	Intensity	Spectral Quality	LC/UV Peak Area	LC/UV Area Percent	
3.25	5255.9	1.37E+07	ok	1.61E+04	0.43	
3.397	5677.4	2.02E+08	ok	1.53E+06	41.17	
3.477	5647.3	9.22E+07	ok	3.02E+05	8.13	
3.567	5752.4	1.34E+08	ok	5.16E+05	13.88	Product
3.63	5752.4	6.90E+07	ok	1.35E+06	36.38	

Purity: 50% - Normalized yield: 59%

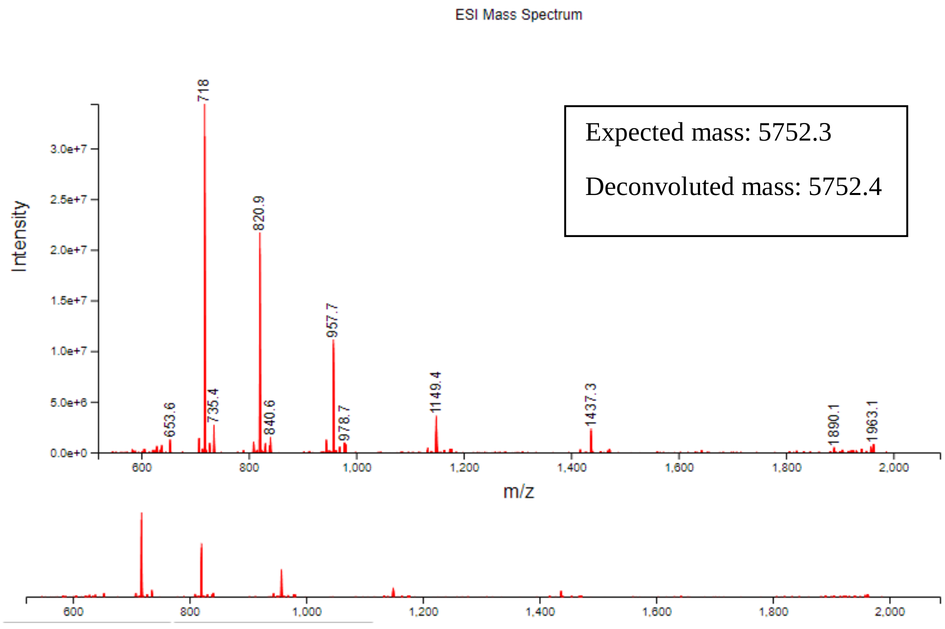

NMR Spectra

NMR Spectra of the starting materials

3-Bromo-1-methyl-1H-indazole **(1q)** *($CDCl_3$, ^{1}H-NMR: 400 MHz, ^{13}C-NMR: 101 MHz, HMBC)*

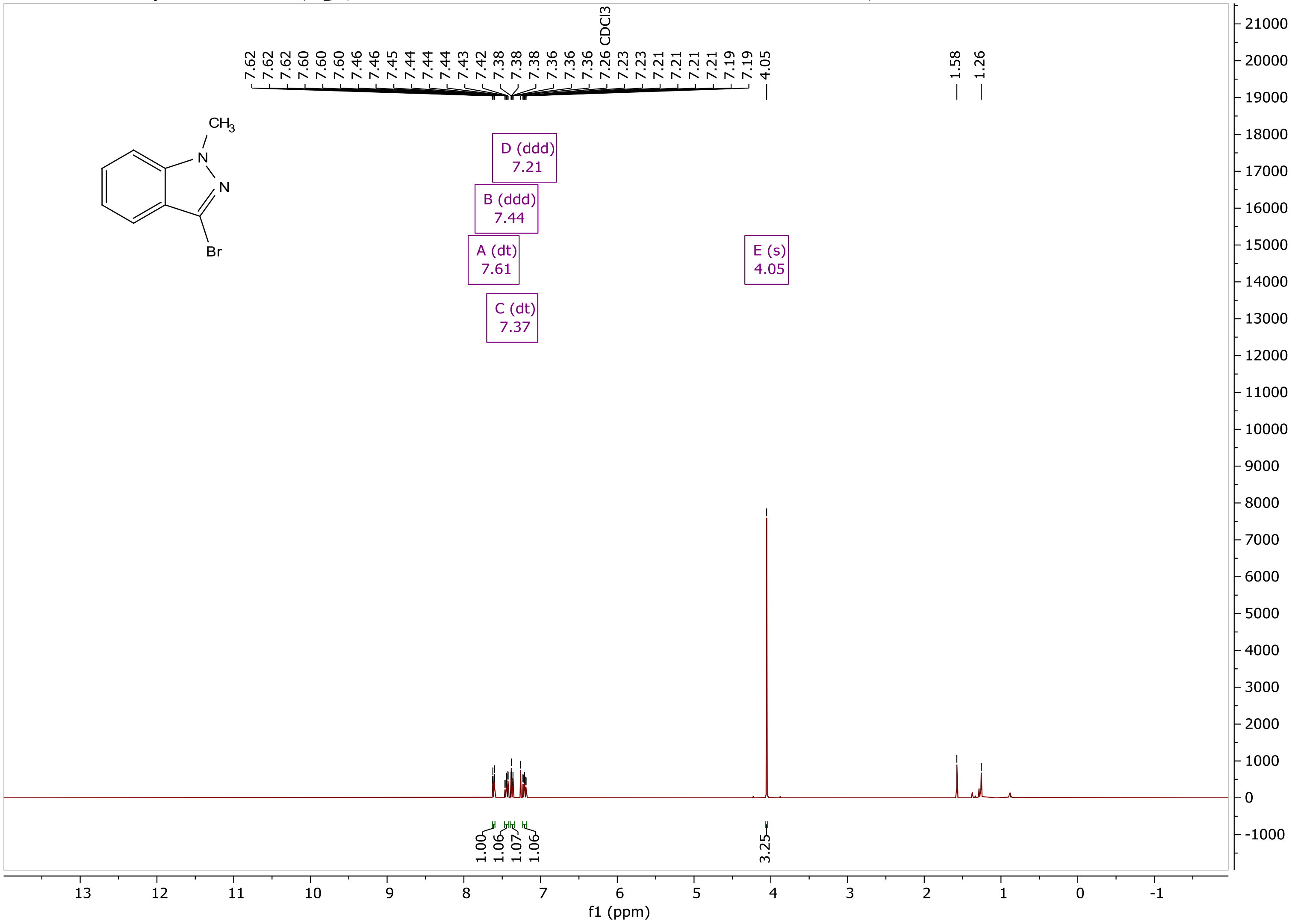

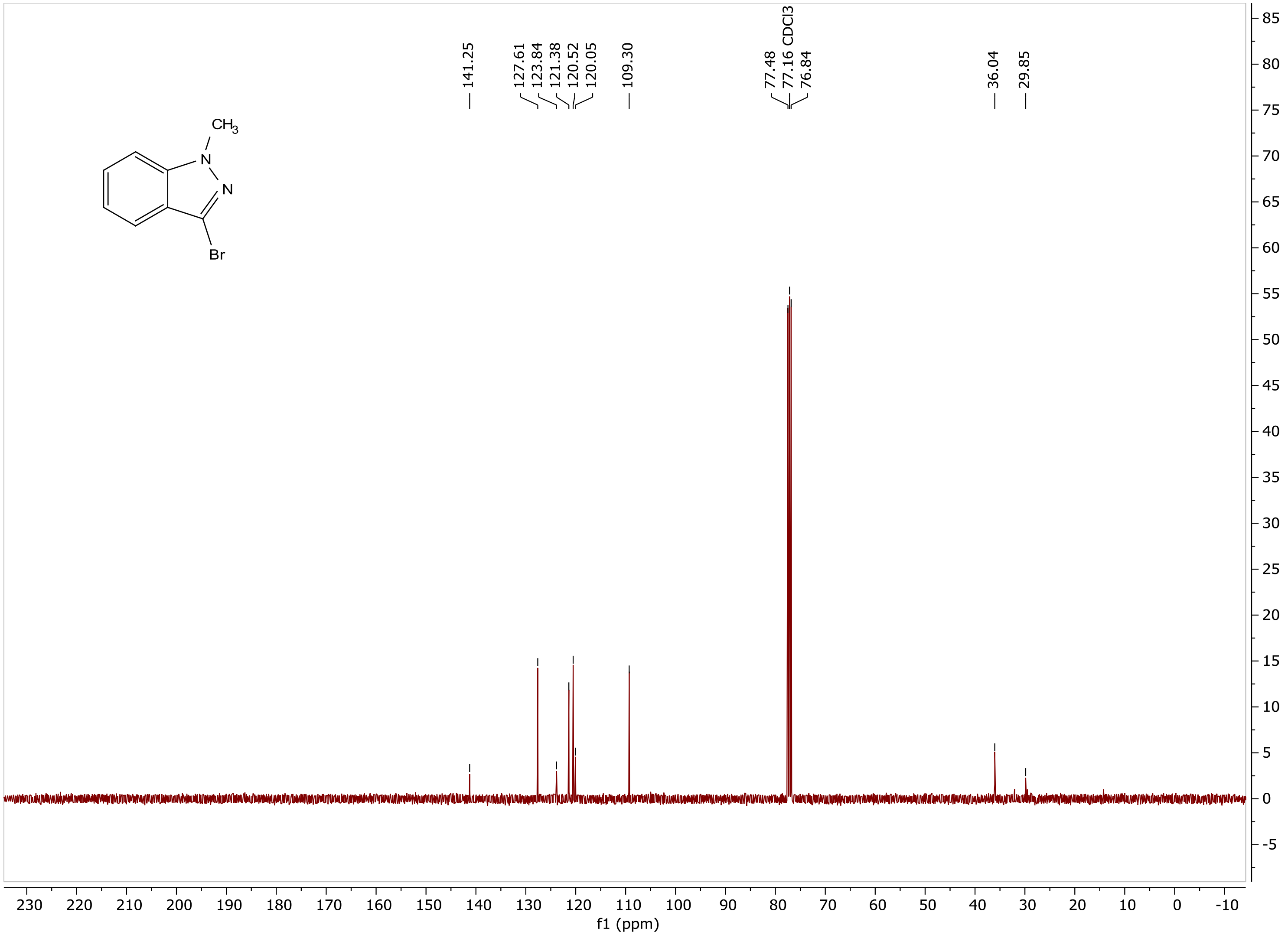

141.25
127.61
123.84
121.38
120.52
120.05
109.30
77.48
77.16 CDCl3
76.84
36.04
29.85
CH3
N
N
Br
85
80
75
70
65
60
55
50
45
40
35
30
25
20
15
10
5
0
-5
230
220
210
200
190
180
170
160
150
140
130
120
110
100
90
80
70
60
50
40
30
20
10
0
-10
f1 (ppm)

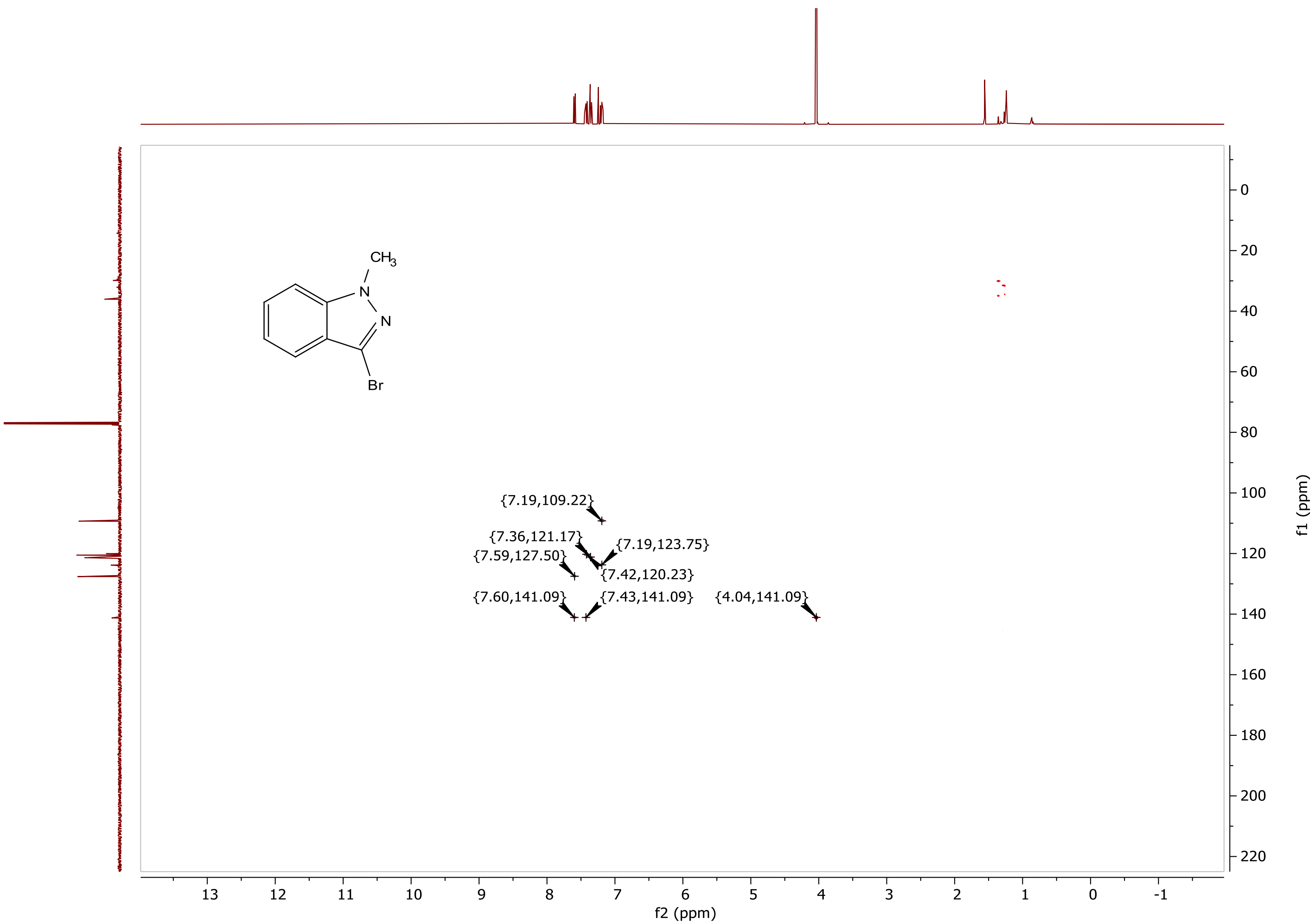

CH3
N
N
Br
{7.19,109.22}
{7.36,121.17}
{7.19,123.75}
{7.59,127.50}
{7.42,120.23}
{7.60,141.09}
{7.43,141.09}
{4.04,141.09}
f1 (ppm)
f2 (ppm)

2-Bromo-1-methyl-1H-benzo[d]imidazole **(1t)** ($CDCl_3$, ^1H-NMR: 400 MHz, ^{13}C-NMR: 101 MHz)

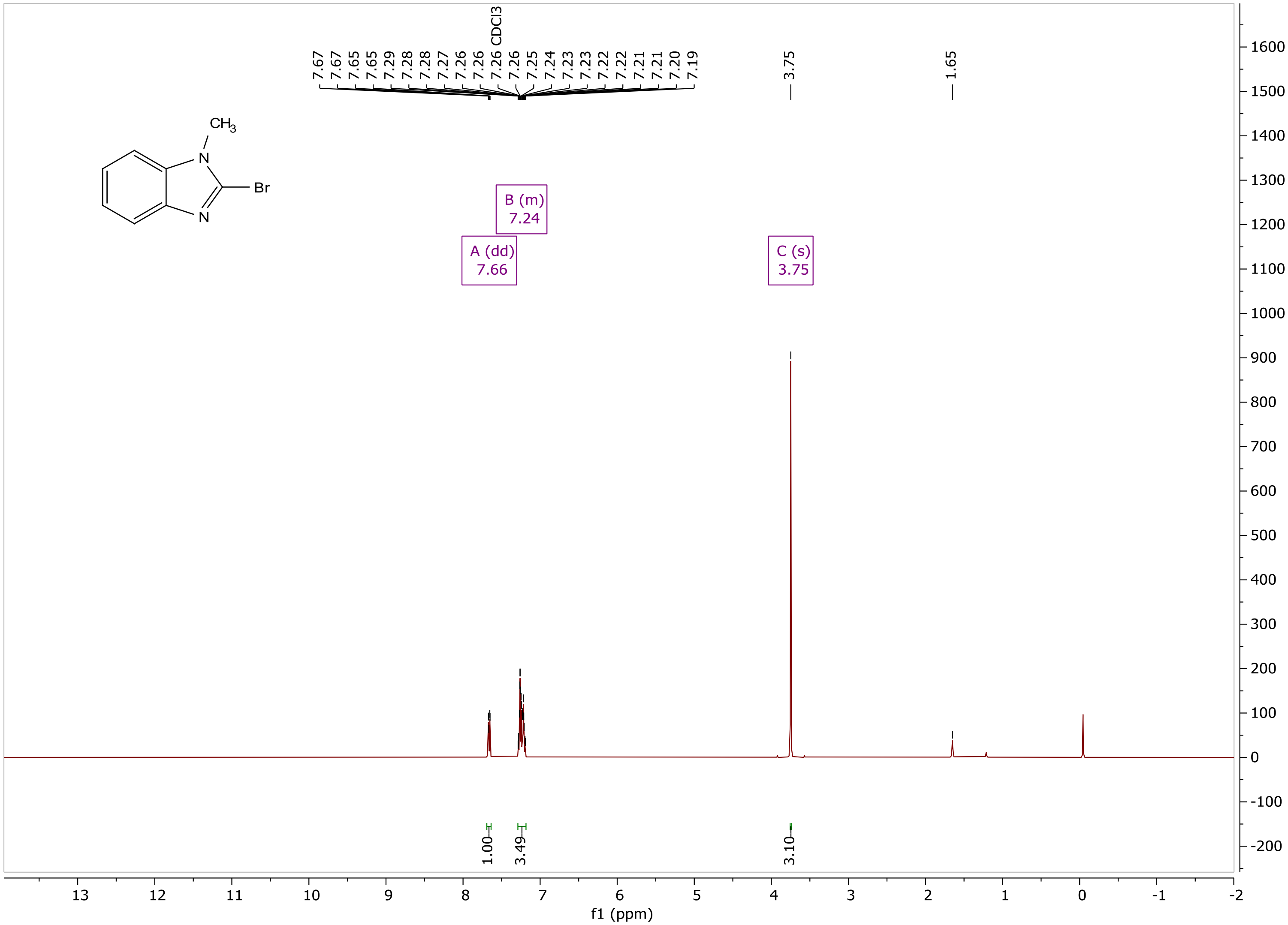

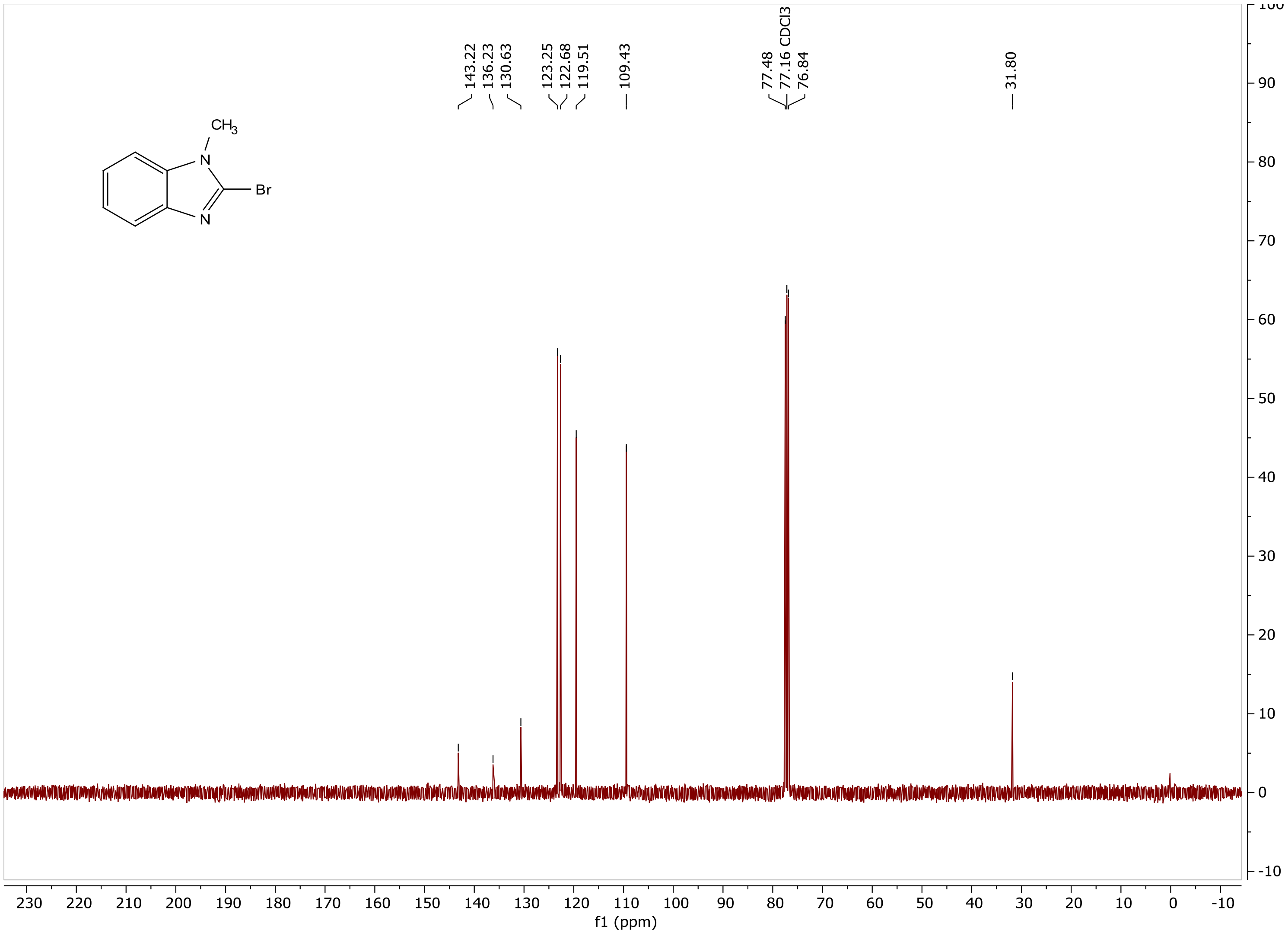

CH3
N
Br
N
143.22
136.23
130.63
123.25
122.68
119.51
109.43
77.48
77.16 CDCl3
76.84
31.80
100
90
80
70
60
50
40
30
20
10
0
-10
230
220
210
200
190
180
170
160
150
140
130
120
110
100
90
80
70
60
50
40
30
20
10
0
-10
f1 (ppm)

NMR Spectra of the ethyl heteroaryl acetates

Ethyl 2-(1,3-thiazol-2-yl)acetate **(2b)** *($CDCl_3$, ¹H-NMR: 400 MHz, ¹³C-NMR: 101 MHz)*

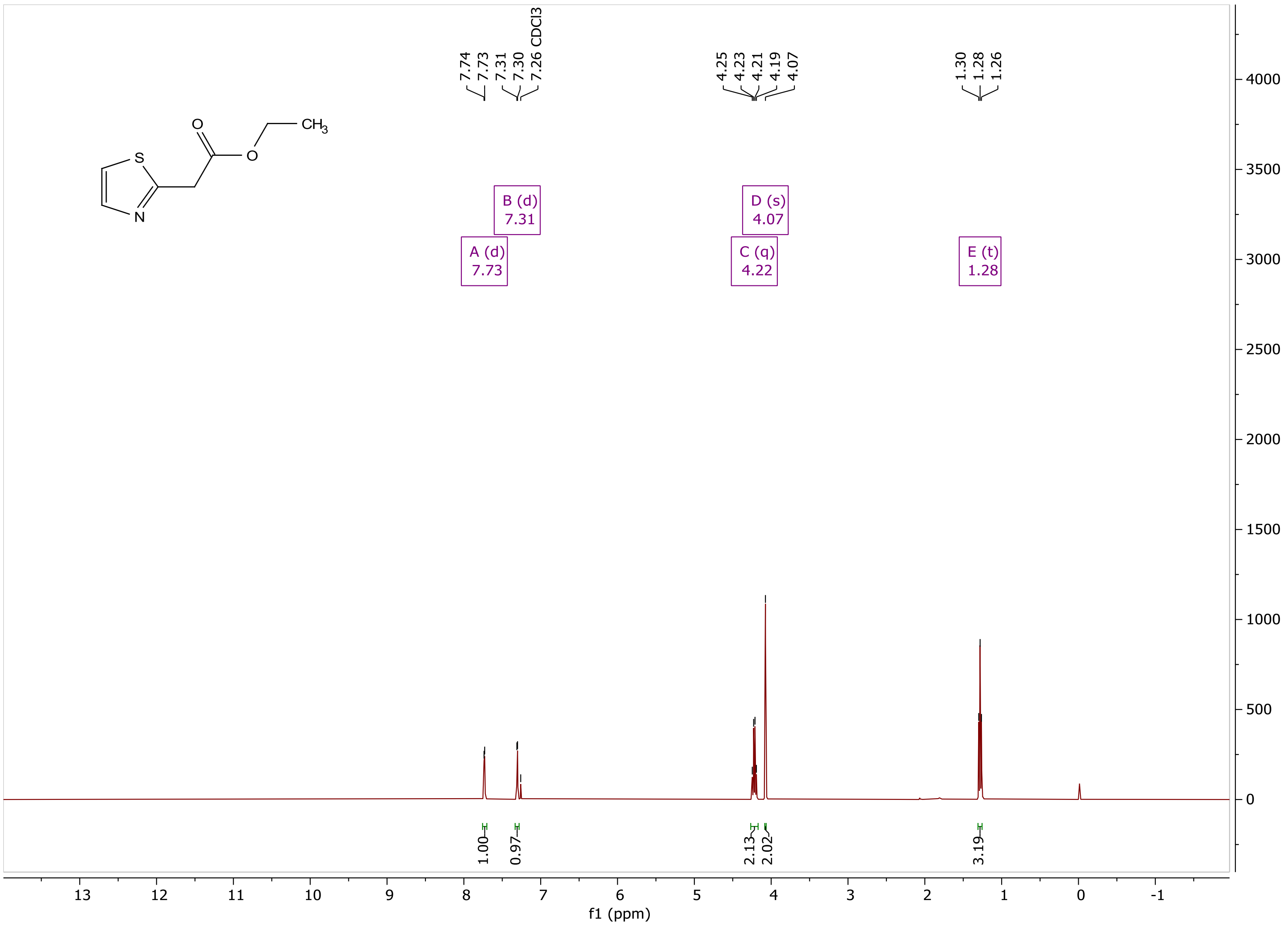

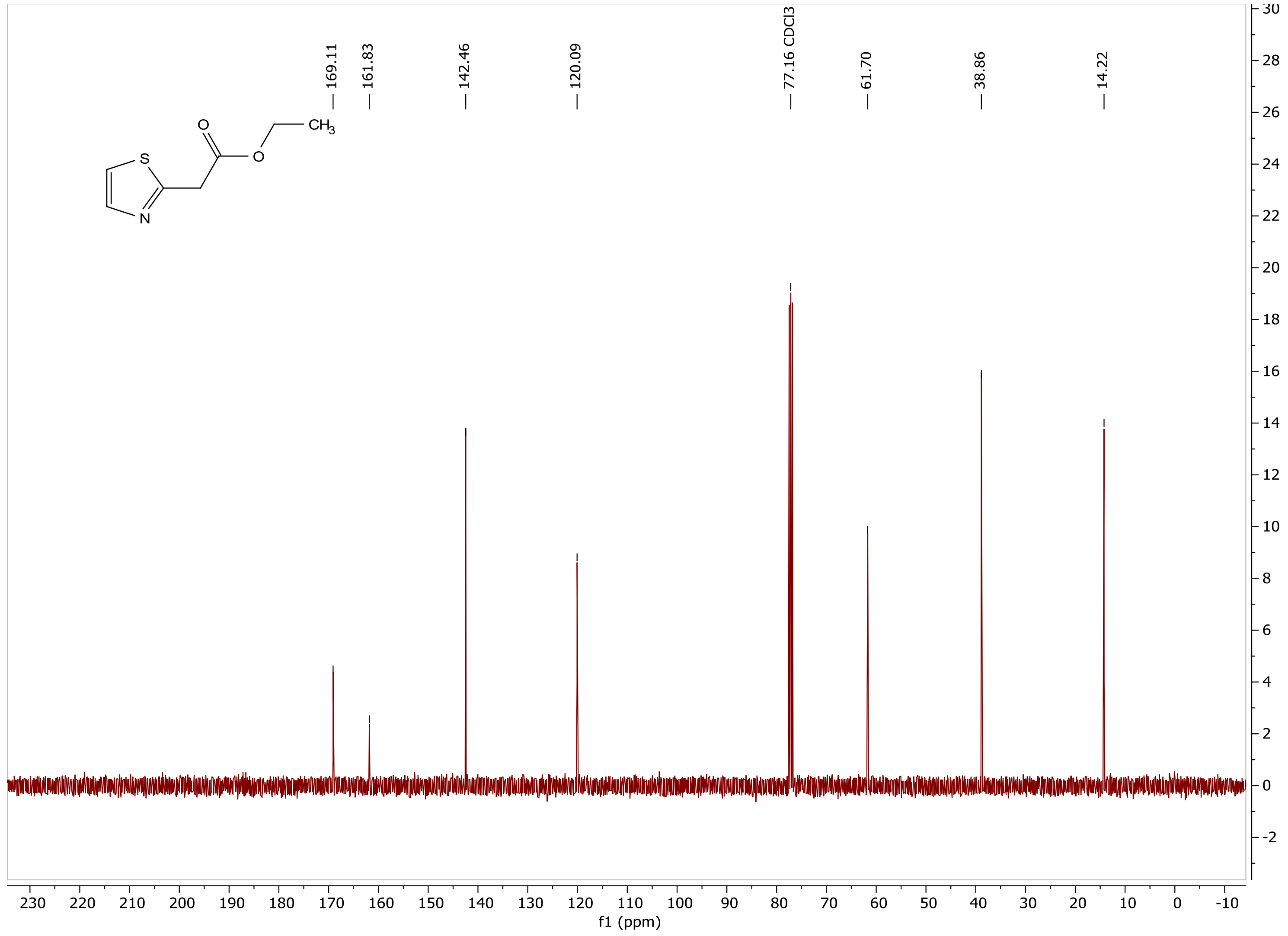
169.11
161.83
142.46
120.09
77.16 CDCl3
61.70
38.86
14.22
O
O
CH3
S
N
230 220 210 200 190 180 170 160 150 140 130 120 110 100 90 80 70 60 50 40 30 20 10 0 -10
f1 (ppm)
30 28 26 24 22 20 18 16 14 12 10 8 6 4 2 0 -2

Ethyl 2-(4-bromo-1,3-thiazol-2-yl)acetate ***(2c)*** *($CDCl_3$, ^{1}H-NMR: 400 MHz, ^{13}C-NMR: 101 MHz, HSQC, HMBC, NOESY)*

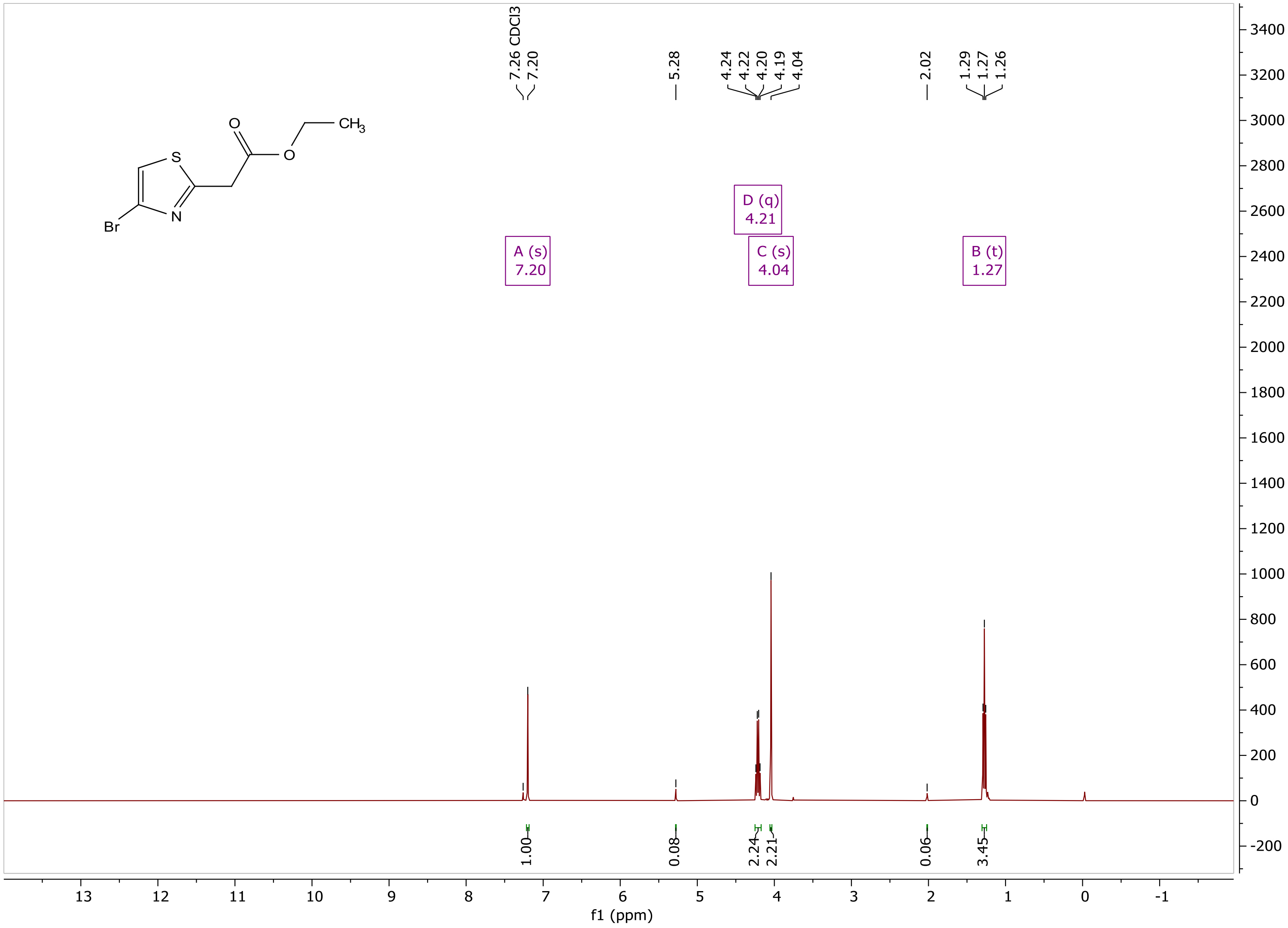

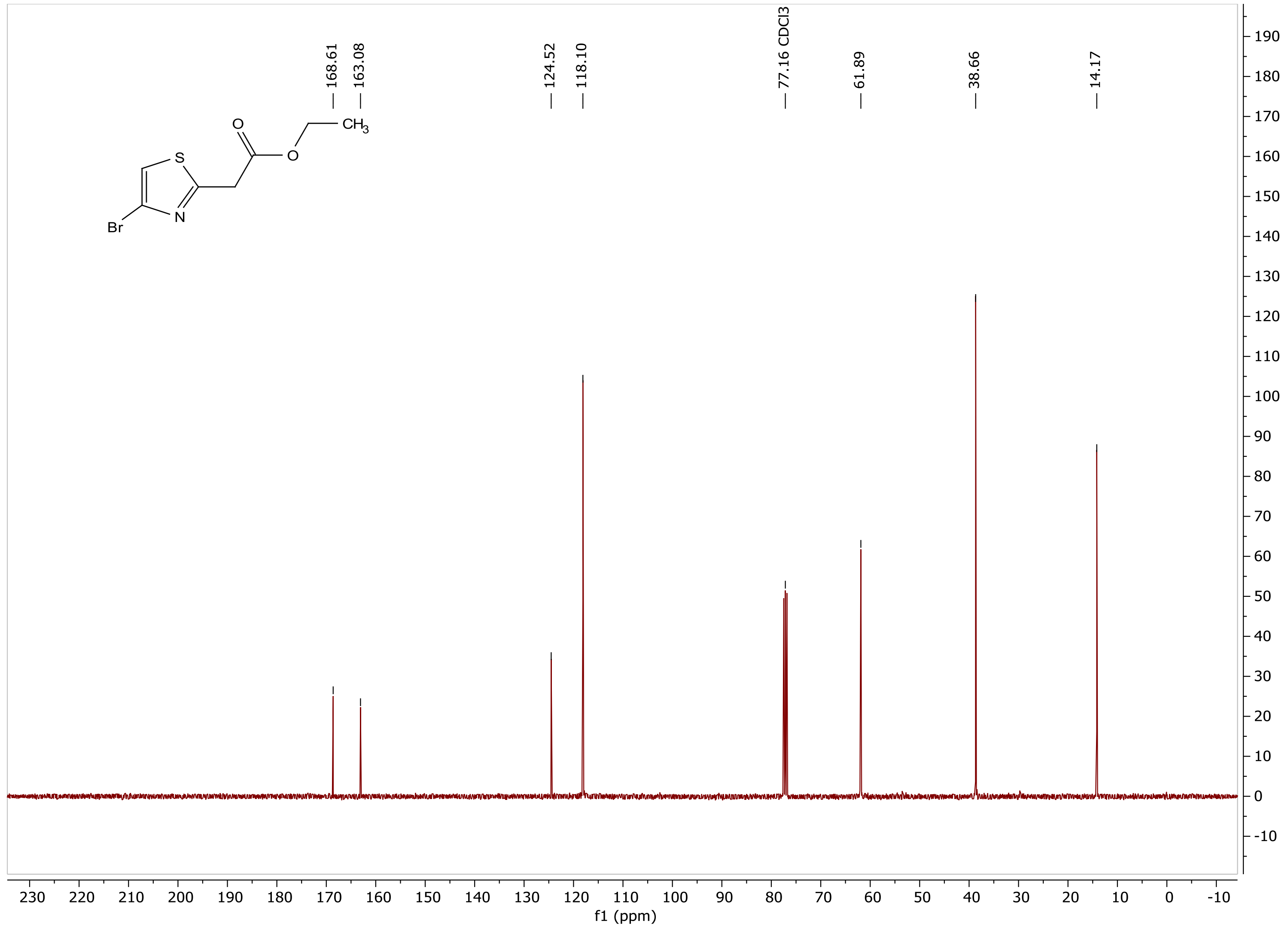

168.61
163.08
124.52
118.10
77.16 CDCl3
61.89
38.66
14.17
S
N
Br
O
O
CH3
f1 (ppm)

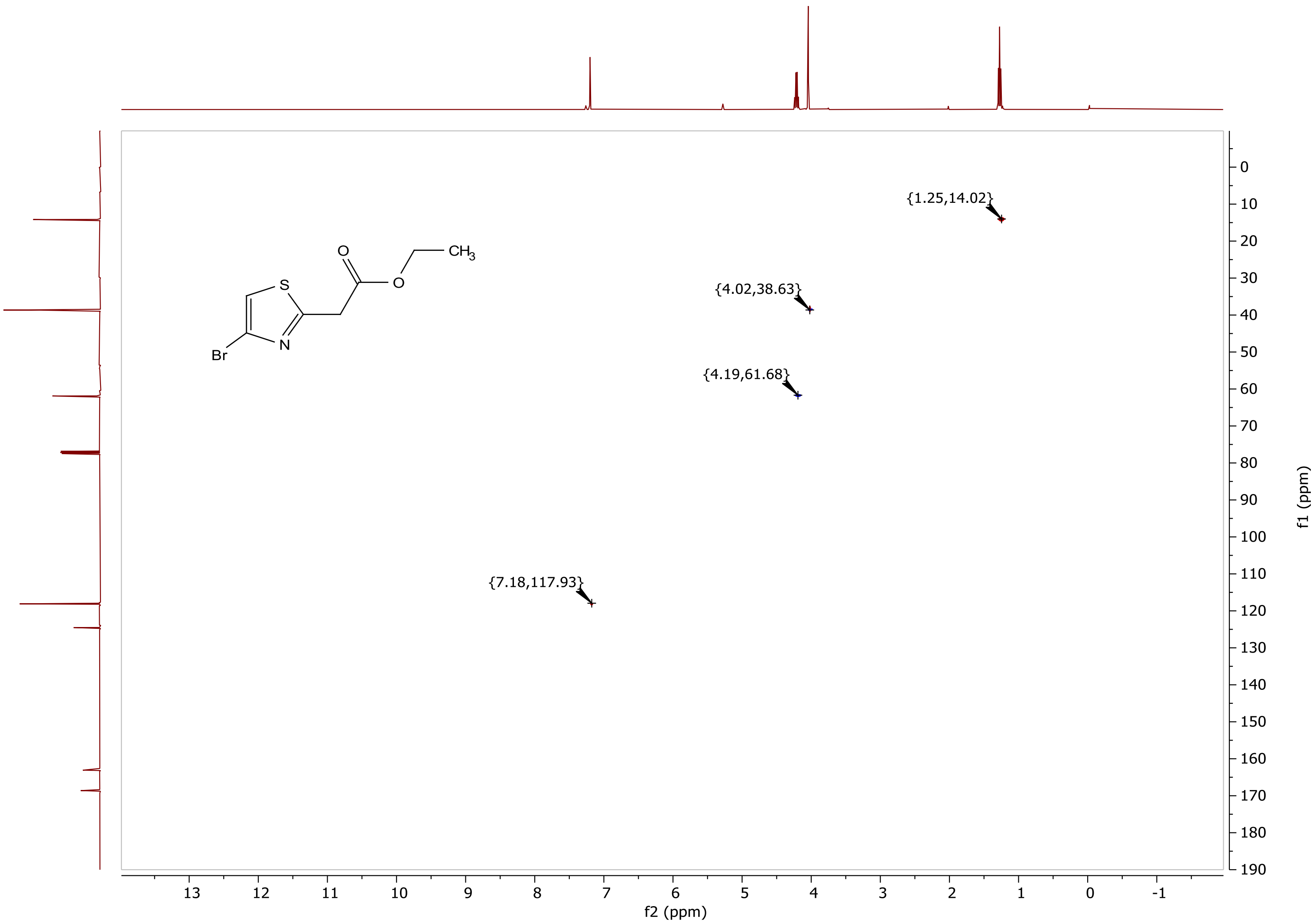
{1.25,14.02}
{4.02,38.63}
{4.19,61.68}
{7.18,117.93}
S
N
Br
O
O
CH3
f2 (ppm)
f1 (ppm)

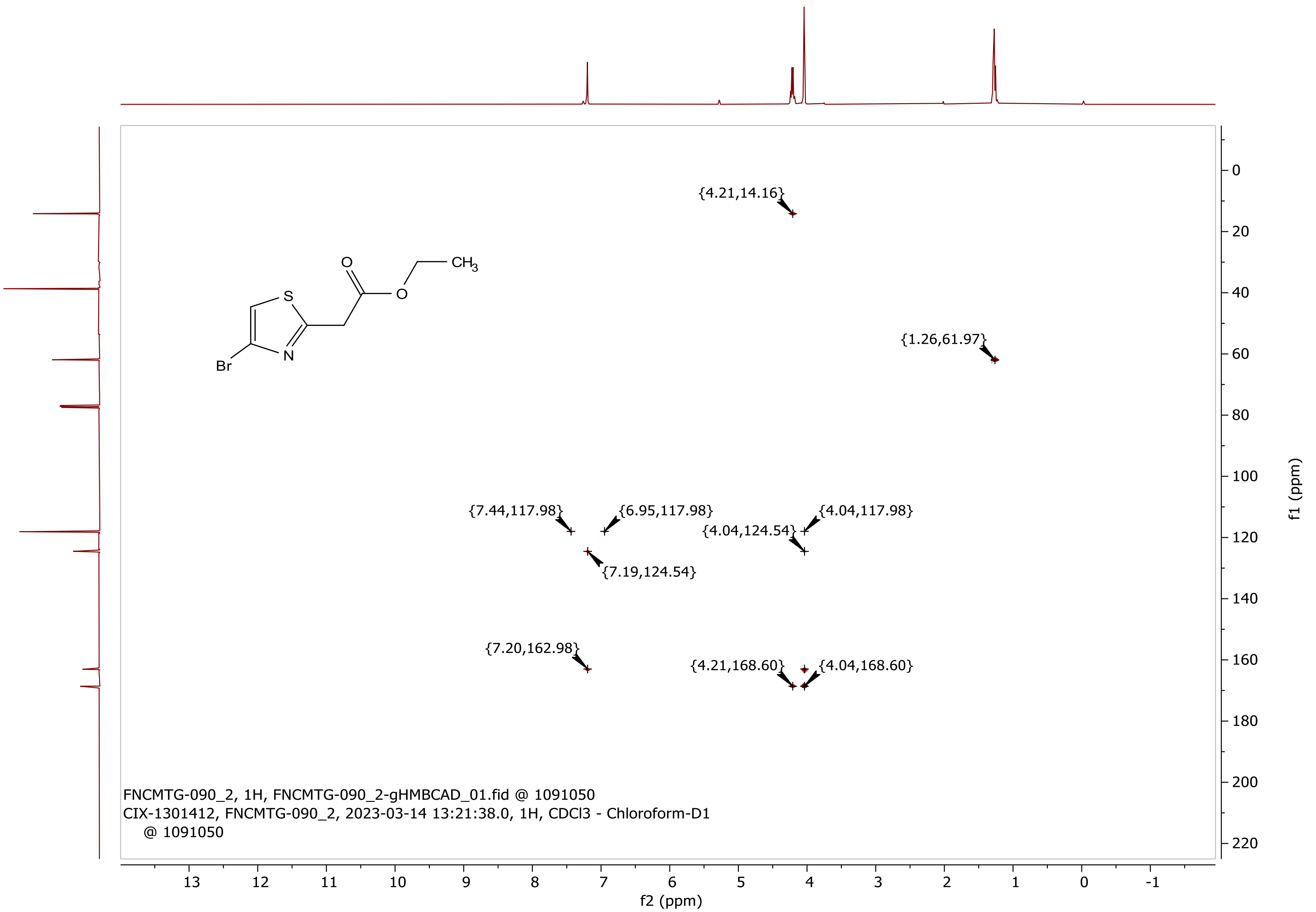
{4.21,14.16}
{1.26,61.97}
{7.44,117.98}
{6.95,117.98}
{4.04,117.98}
{4.04,124.54}
{7.19,124.54}
{7.20,162.98}
{4.21,168.60}
{4.04,168.60}
FNCMTG-090_2, 1H, FNCMTG-090_2-gHMBCAD_01.fid @ 1091050
CIX-1301412, FNCMTG-090_2, 2023-03-14 13:21:38.0, 1H, CDCl3 - Chloroform-D1
@ 1091050
f2 (ppm)
f1 (ppm)

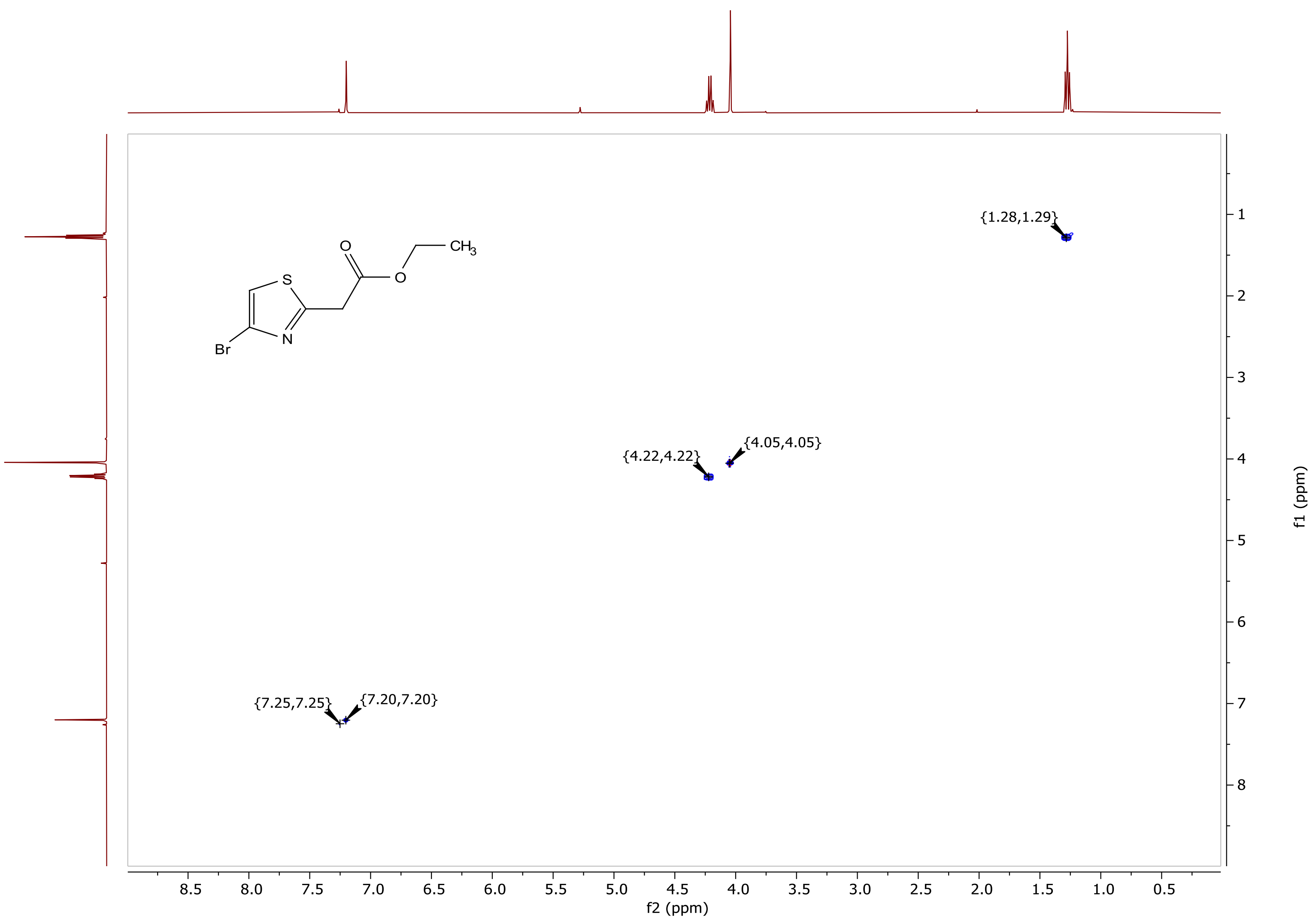

Br
S
N
O
O
CH3
{1.28,1.29}
{4.22,4.22}
{4.05,4.05}
{7.25,7.25}
{7.20,7.20}
f2 (ppm)
f1 (ppm)

Ethyl 2-(5-chloro-1,3-thiazol-2-yl)acetate ***(2d)*** *($CDCl_3$, ^{1}H-NMR: 500 MHz, ^{13}C-NMR: 126 MHz)*

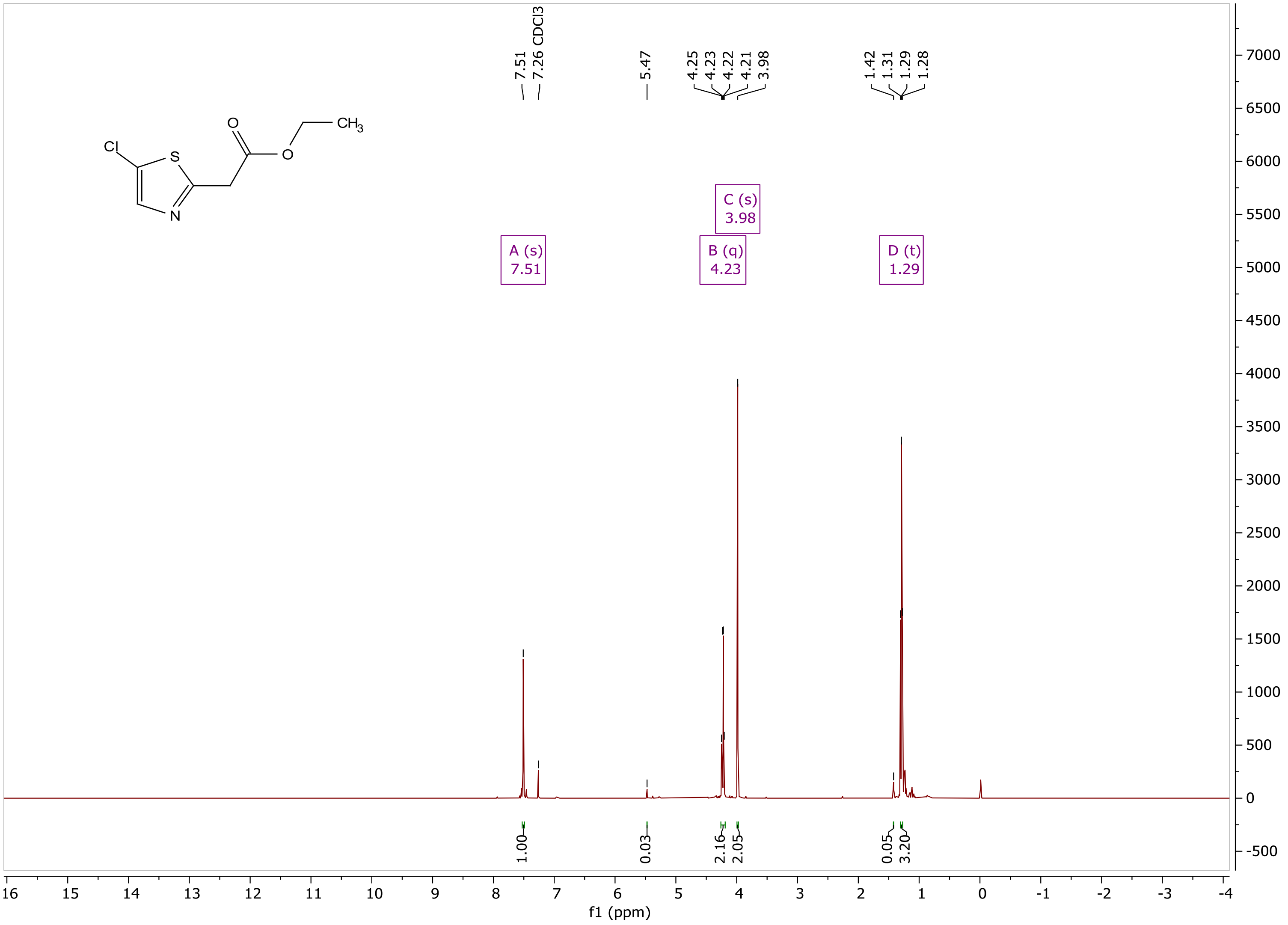

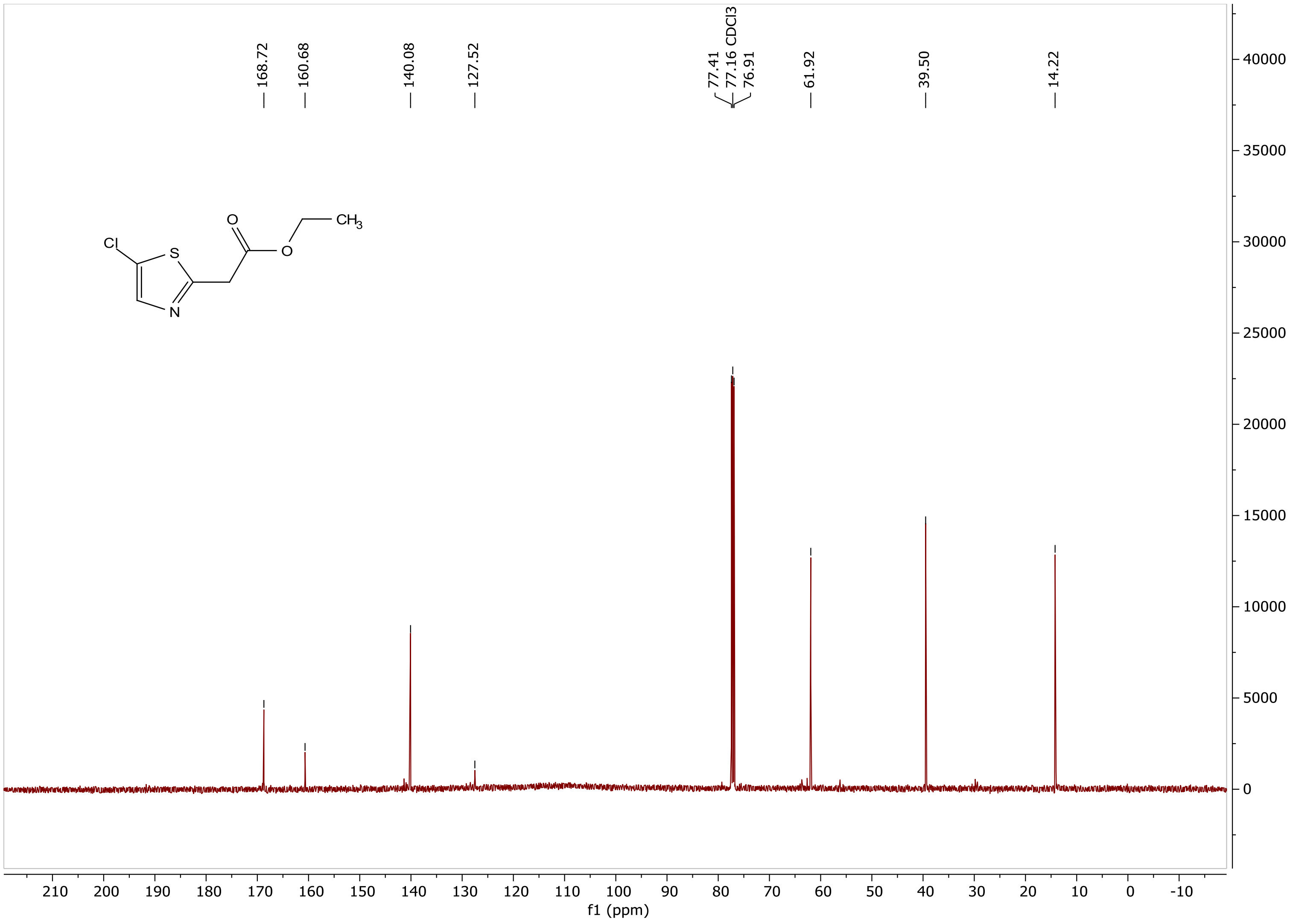
168.72
160.68
140.08
127.52
77.41
77.16 CDCl3
76.91
61.92
39.50
14.22
Cl
S
N
O
O
CH3
40000
35000
30000
25000
20000
15000
10000
5000
0
210
200
190
180
170
160
150
140
130
120
110
100
90
80
70
60
50
40
30
20
10
0
-10
f1 (ppm)

Ethyl 2-(1,3-thiazol-5-yl)acetate ***(2e)*** *($CDCl_3$, ^{1}H-NMR: 400 MHz, ^{13}C-NMR: 101 MHz)*

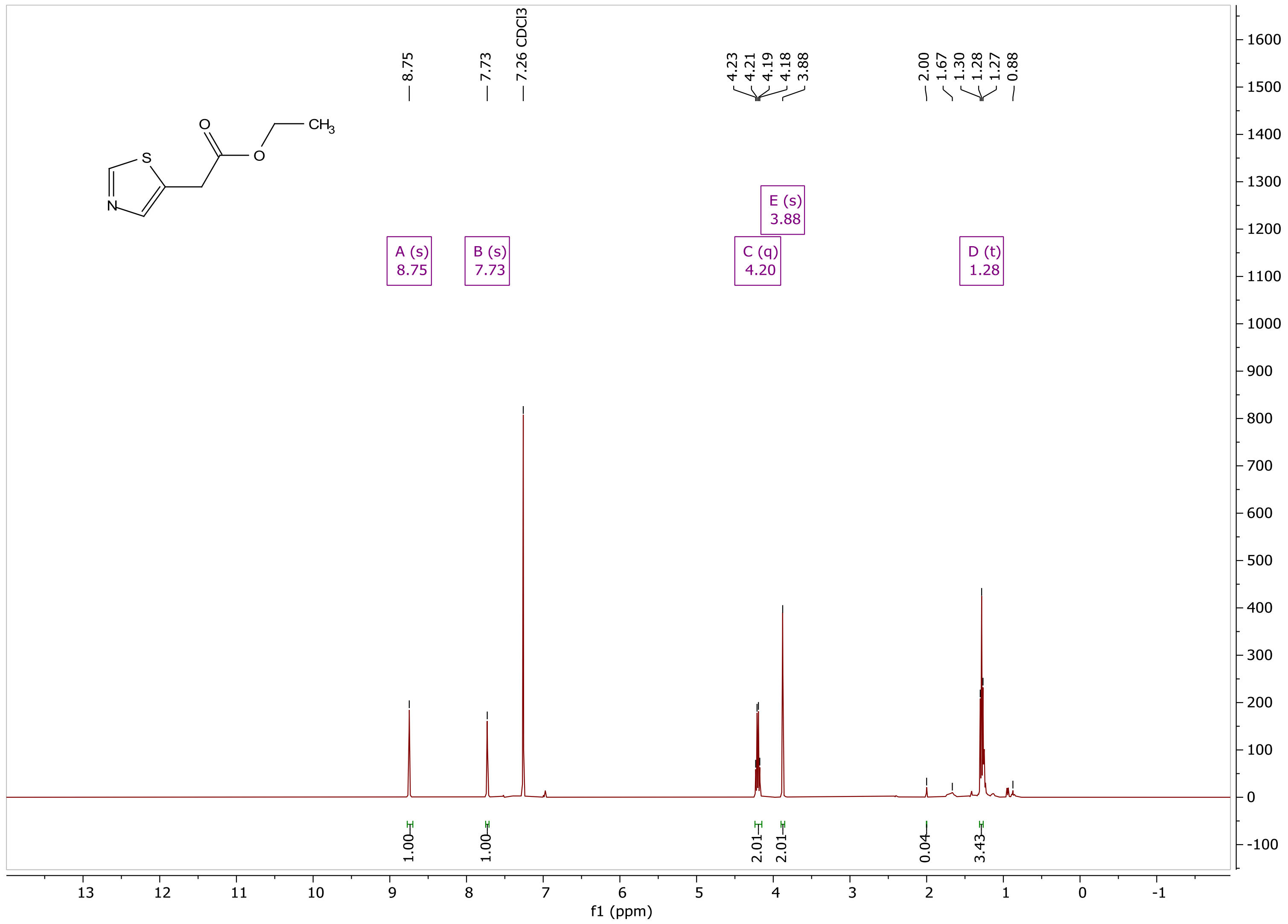

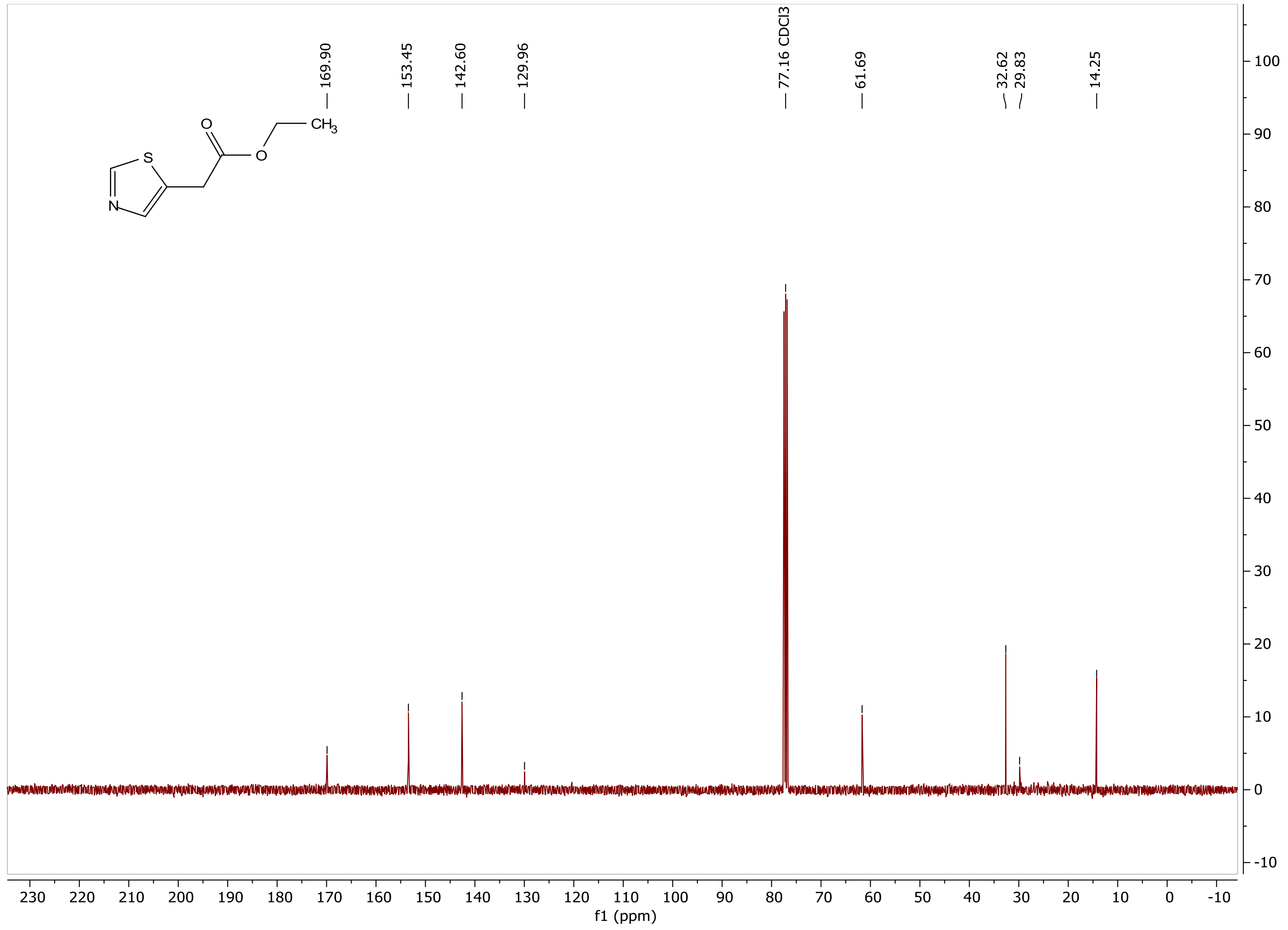

169.90
153.45
142.60
129.96
77.16 CDCl3
61.69
32.62
29.83
14.25
O
O
CH3
S
N
230 220 210 200 190 180 170 160 150 140 130 120 110 100 90 80 70 60 50 40 30 20 10 0 -10
f1 (ppm)
100 90 80 70 60 50 40 30 20 10 0 -10

2-(2-Ethoxy-2-oxoethyl)thiazole-4-carboxylic acid **(2f)** (CDCl$_3$, ^{1}H-NMR: 500 MHz, ^{13}C-NMR: 126 MHz)

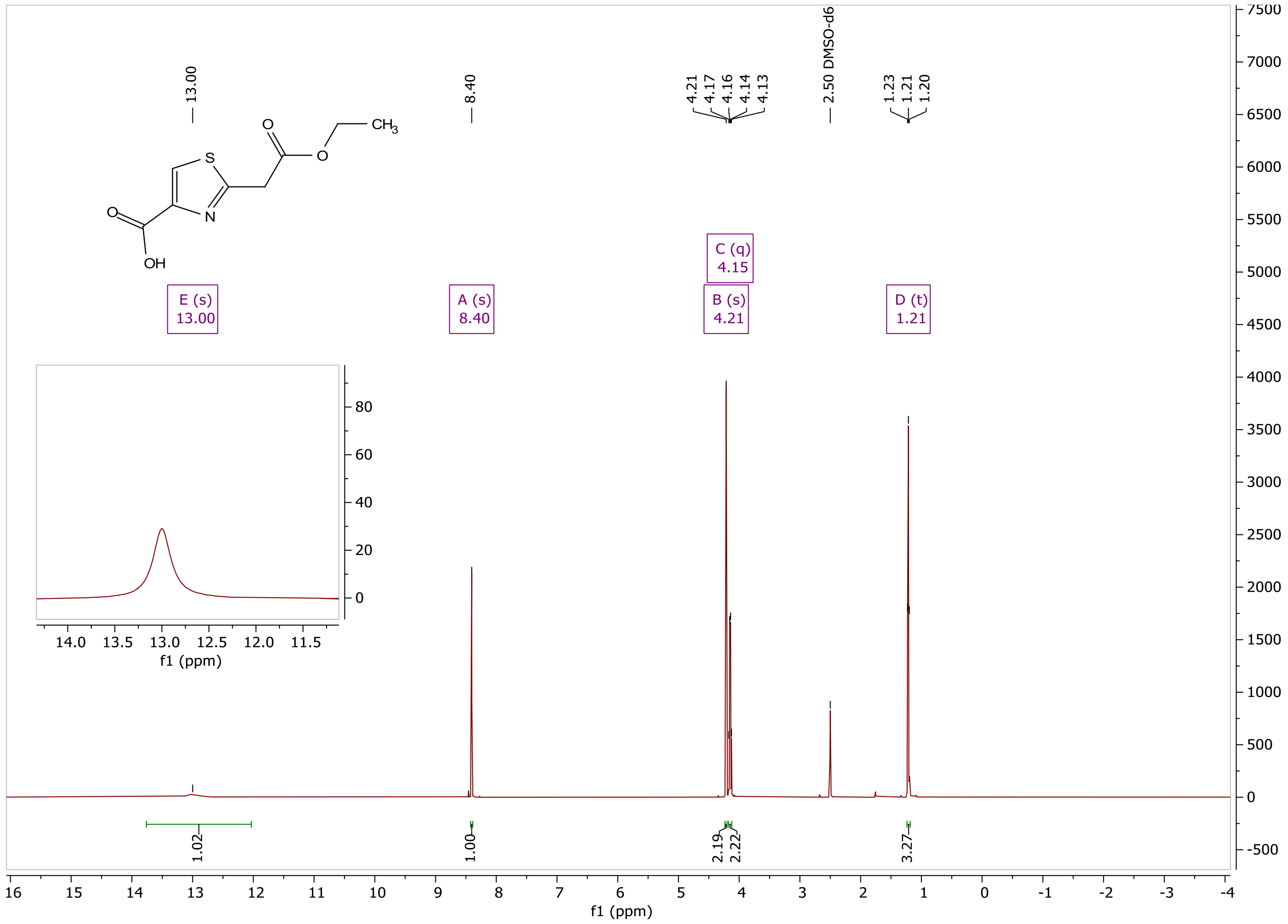

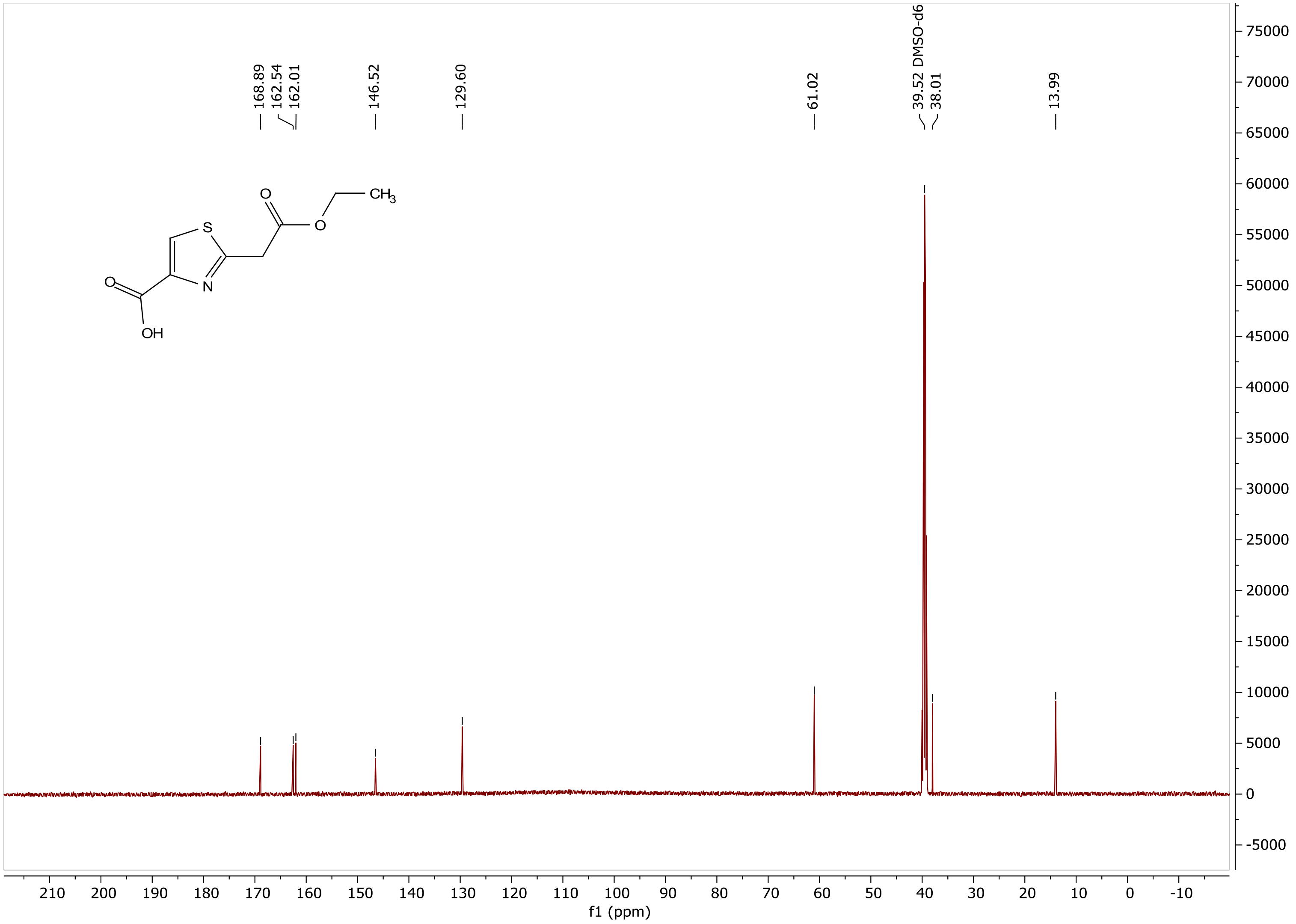

168.89
162.54
162.01
146.52
129.60
61.02
39.52 DMSO-d6
38.01
13.99
O
CH3
S
O
O
N
OH
75000
70000
65000
60000
55000
50000
45000
40000
35000
30000
25000
20000
15000
10000
5000
0
-5000
210
200
190
180
170
160
150
140
130
120
110
100
90
80
70
60
50
40
30
20
10
0
-10
f1 (ppm)

Ethyl 2-(1,3-benzothiazol-2-yl)acetate **(2i)** *(CDCl$_3$, ^{1}H-NMR: 400 MHz, ^{13}C-NMR: 101 MHz)*

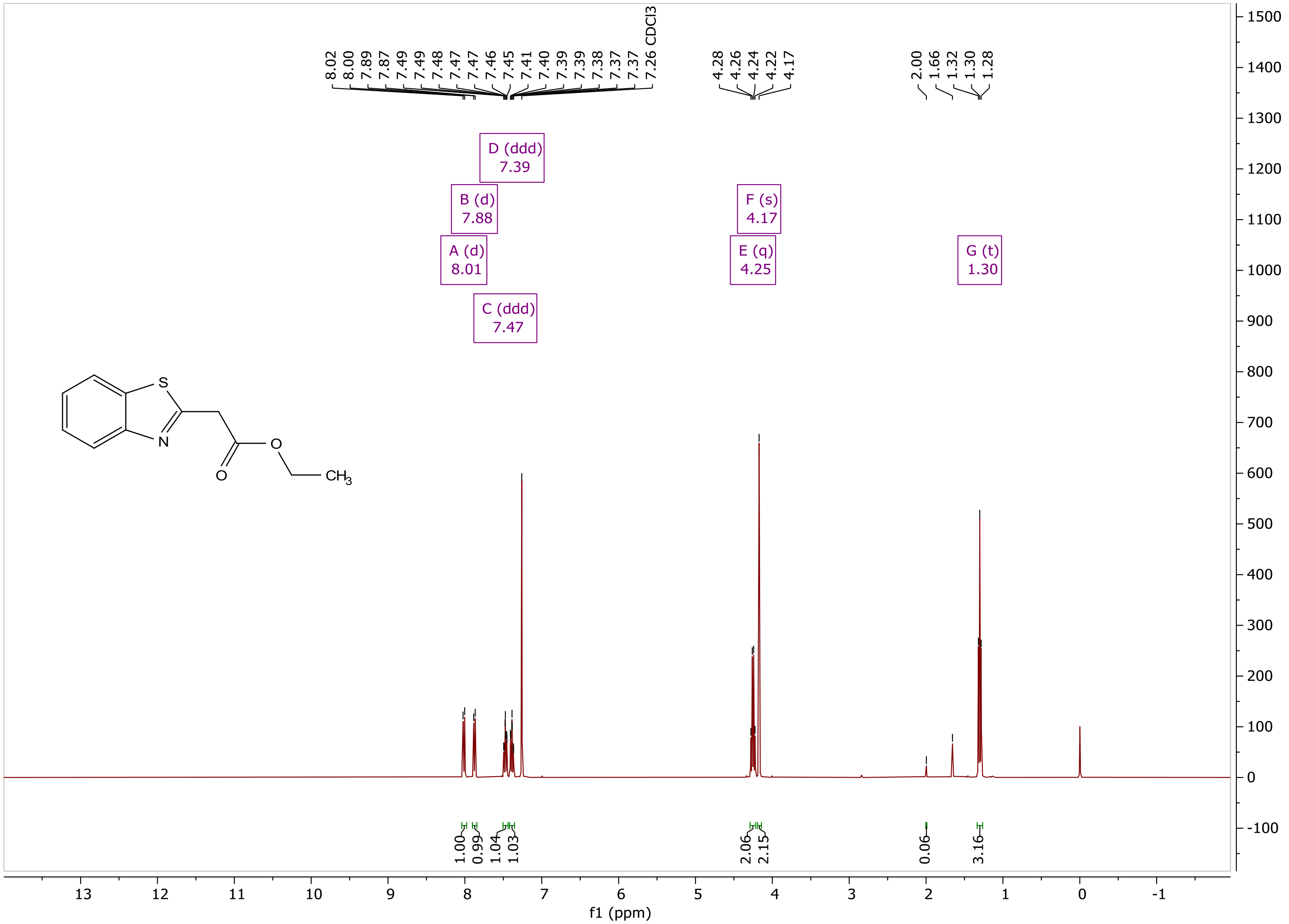

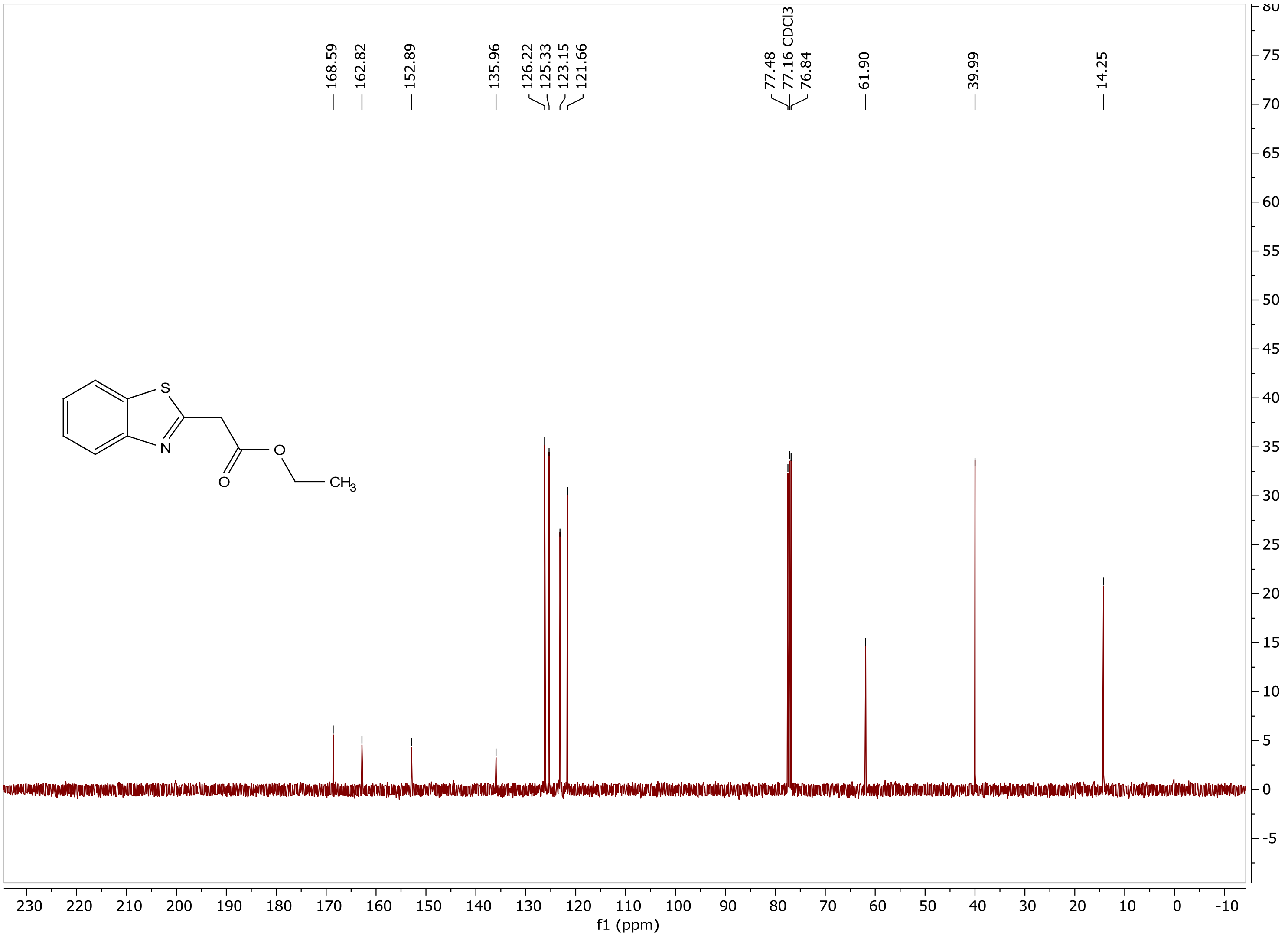

168.59
162.82
152.89
135.96
126.22
125.33
123.15
121.66
77.48
77.16 CDCl3
76.84
61.90
39.99
14.25
S
N
O
O
CH3
f1 (ppm)

Ethyl 2-(furan-3-yl)acetate **(2l)** *(CDCl*$_3$*,* ^{1}*H-NMR: 400 MHz,* ^{13}*C-NMR: 101 MHz)*

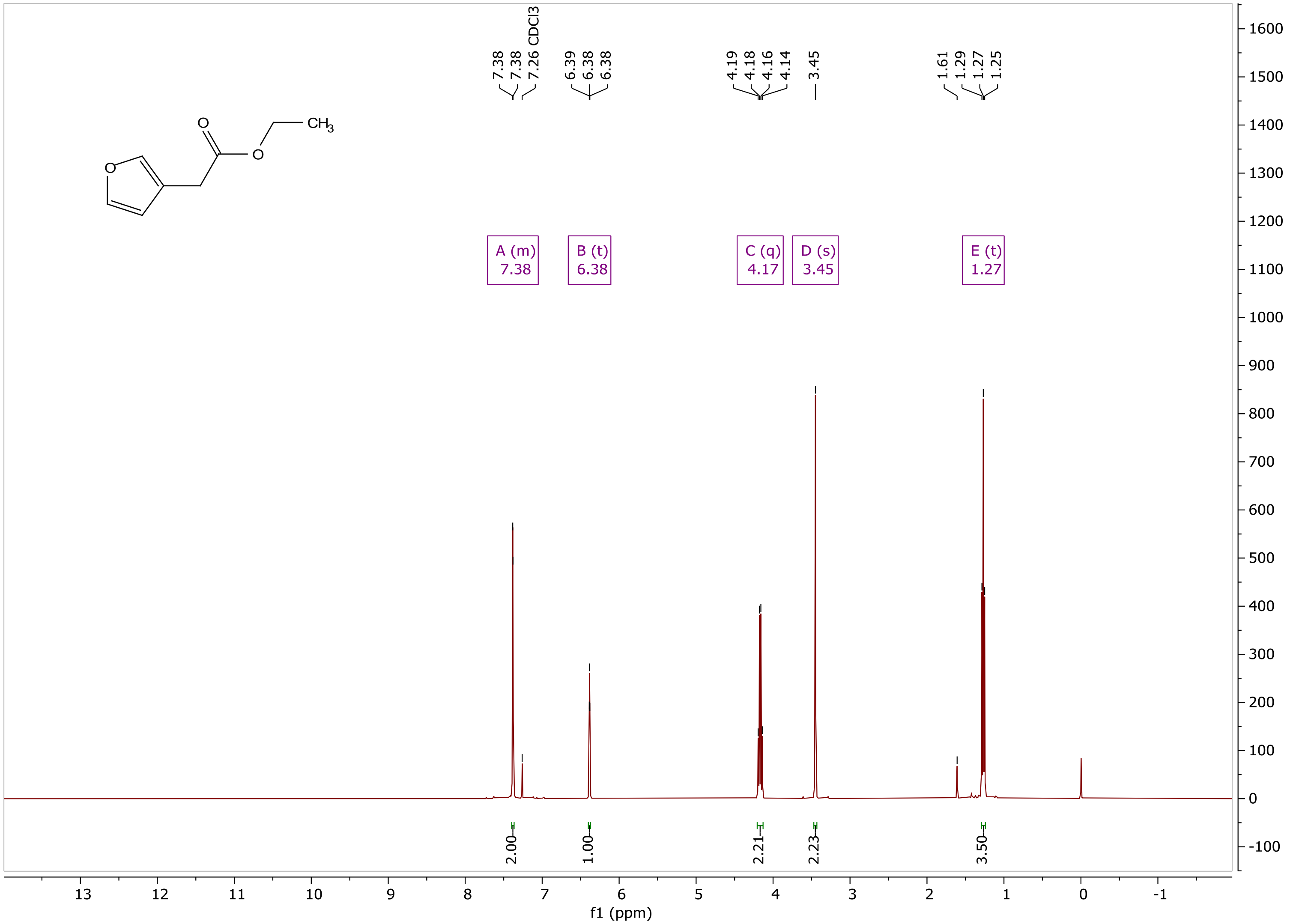

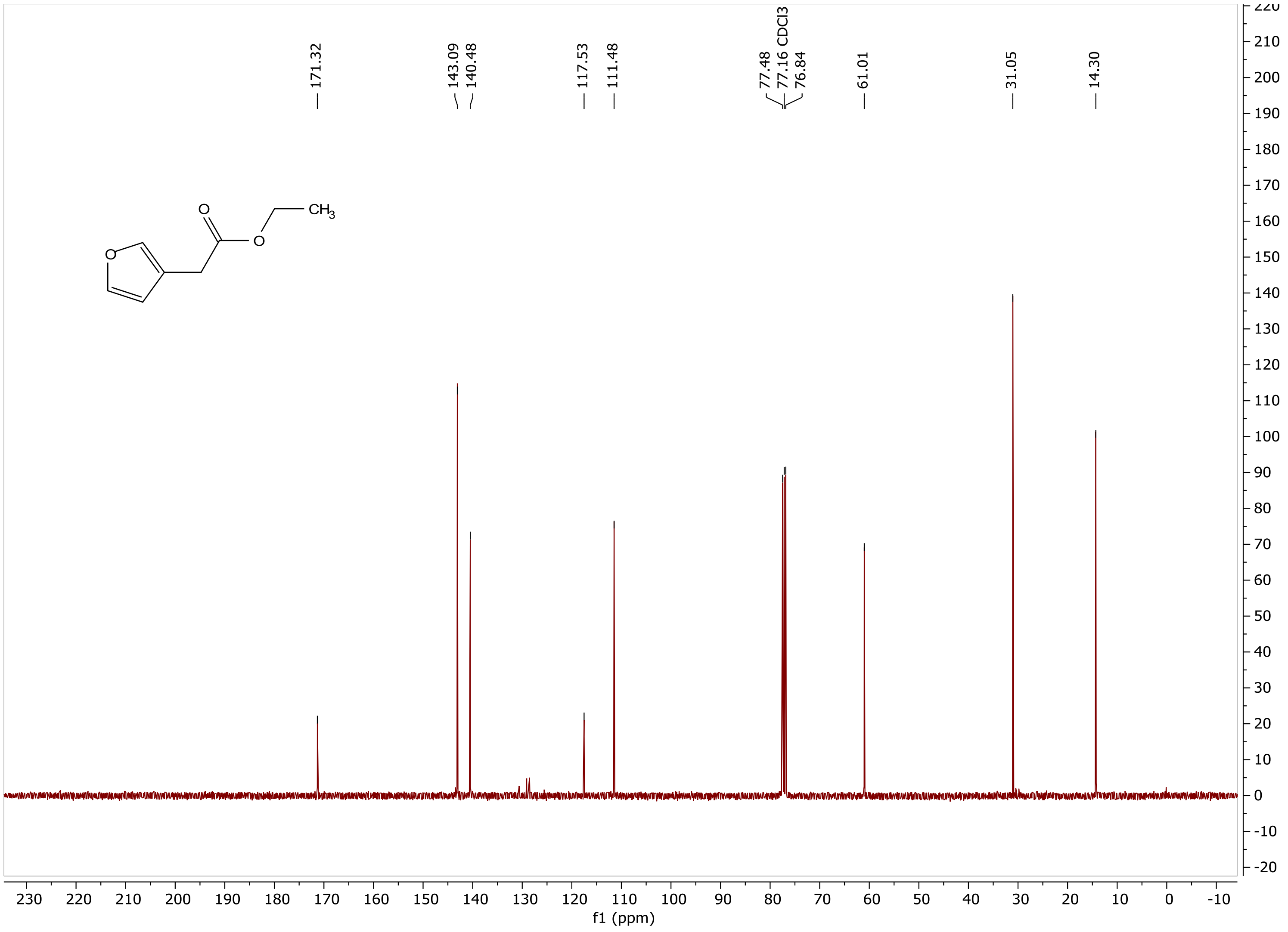

171.32
143.09
140.48
117.53
111.48
77.48
77.16 CDCl3
76.84
61.01
31.05
14.30
O
O
O
CH3
f1 (ppm)

Ethyl 2-(1H-pyrazol-3-yl)acetate **(2k)** *(CDCl$_3$, ^{1}H-NMR: 400 MHz, ^{13}C-NMR: 101 MHz)*

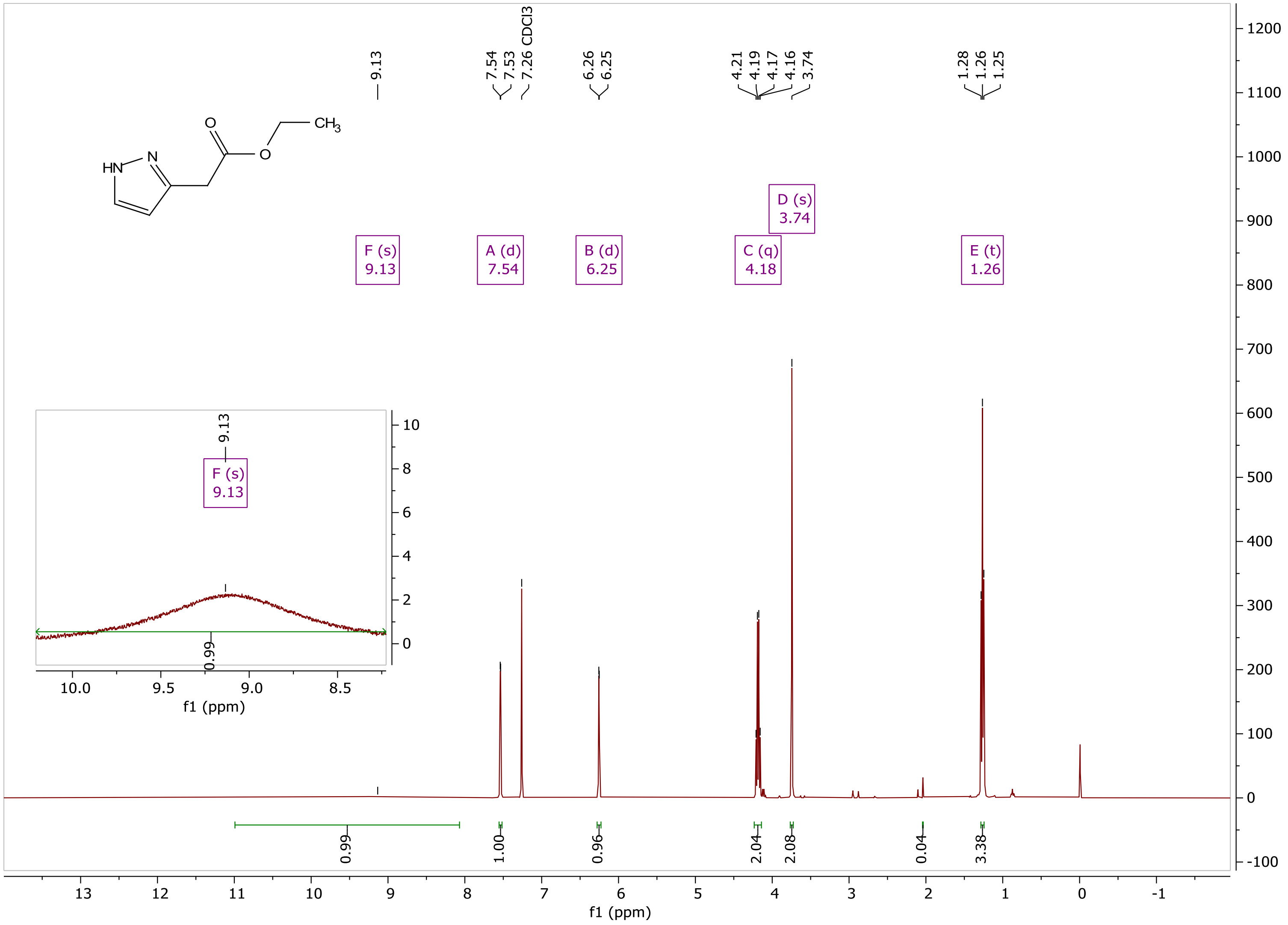

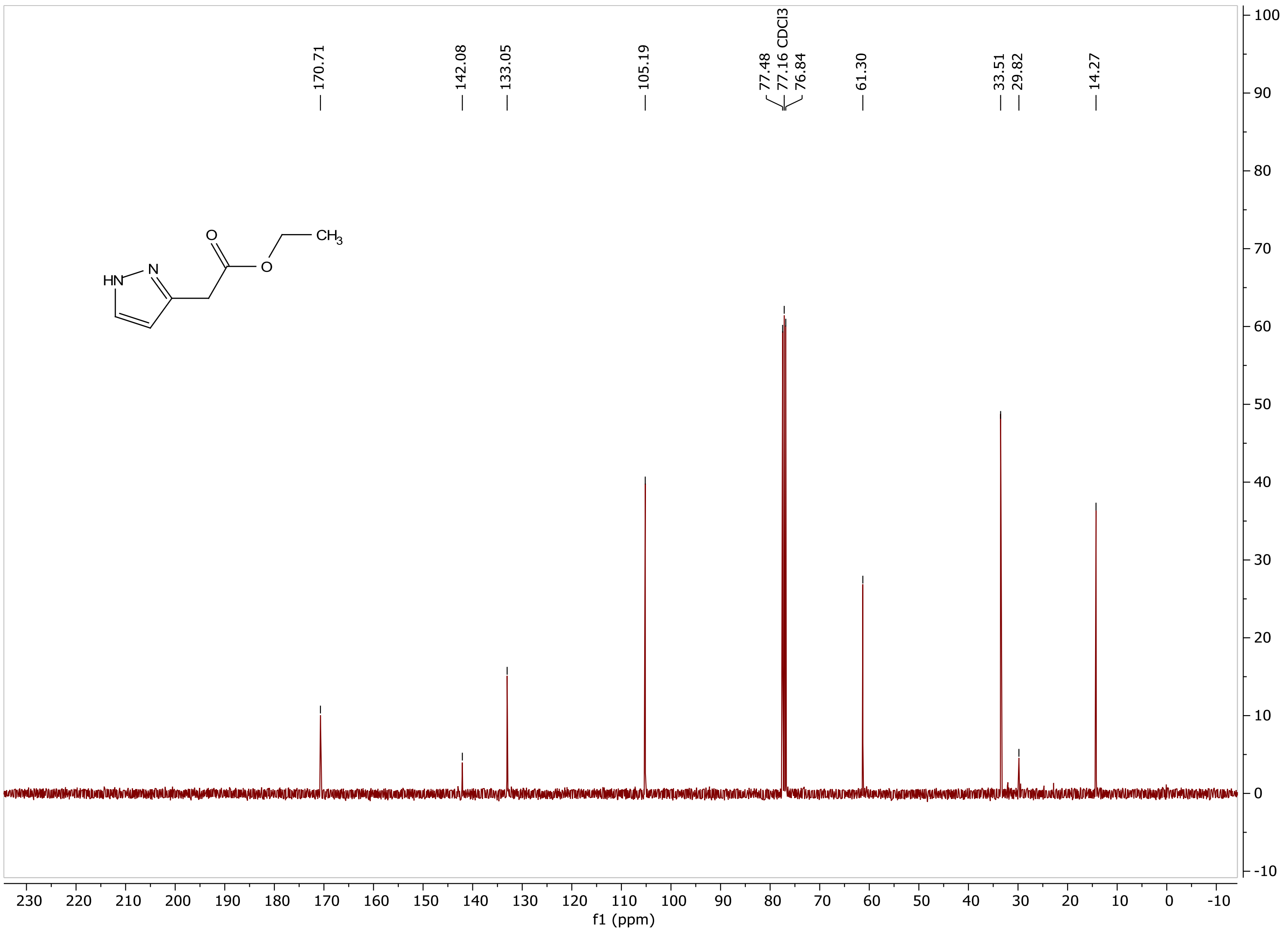
170.71
142.08
133.05
105.19
77.48
77.16 CDCl3
76.84
61.30
33.51
29.82
14.27
HN
N
O
O
CH3
f1 (ppm)

Ethyl 2-(1-benzyl-1H-pyrazol-4-yl)acetate ***(2l)*** *($CDCl_3$, ^{1}H-NMR: 400 MHz, ^{13}C-NMR: 101 MHz)*

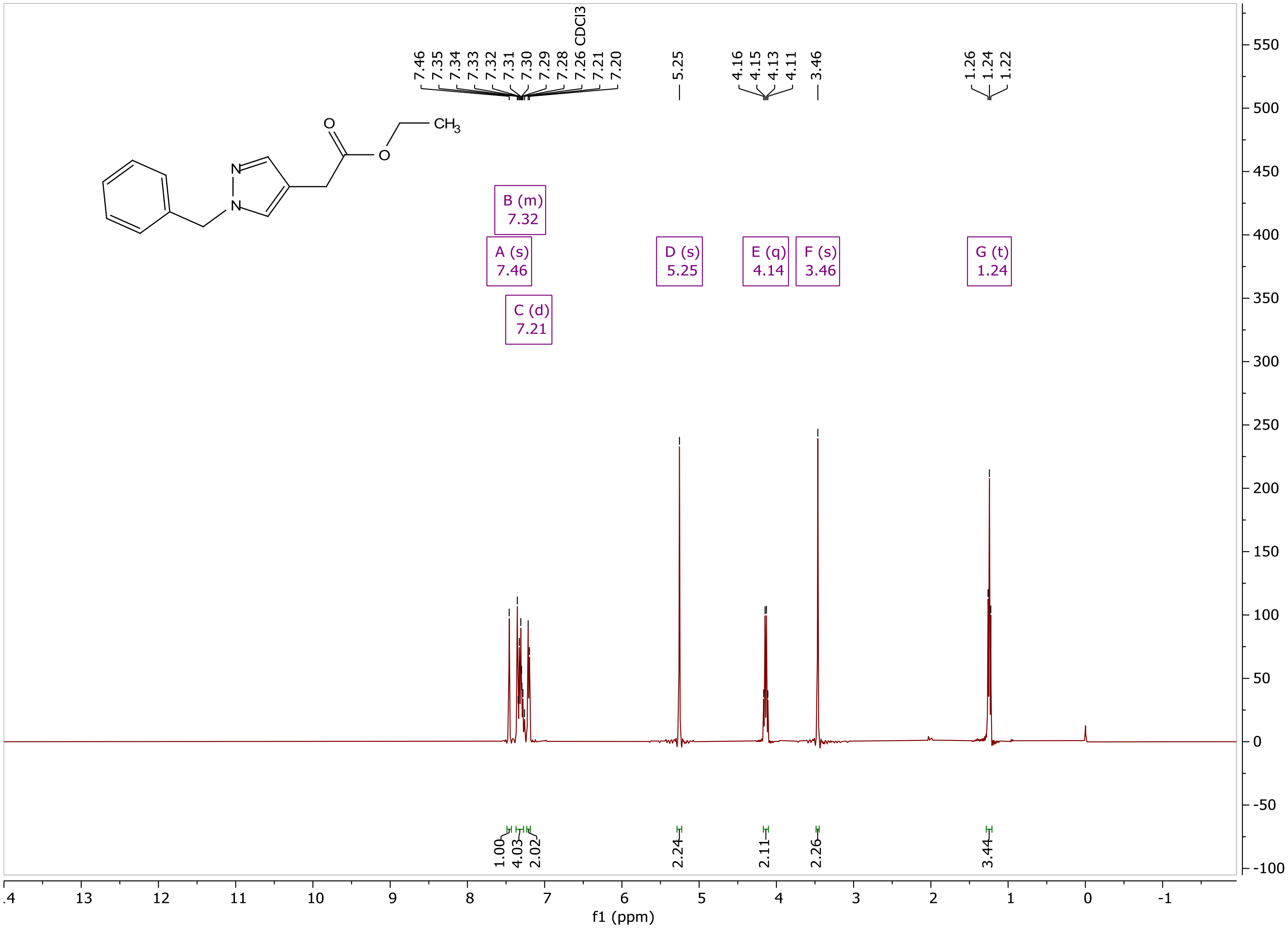

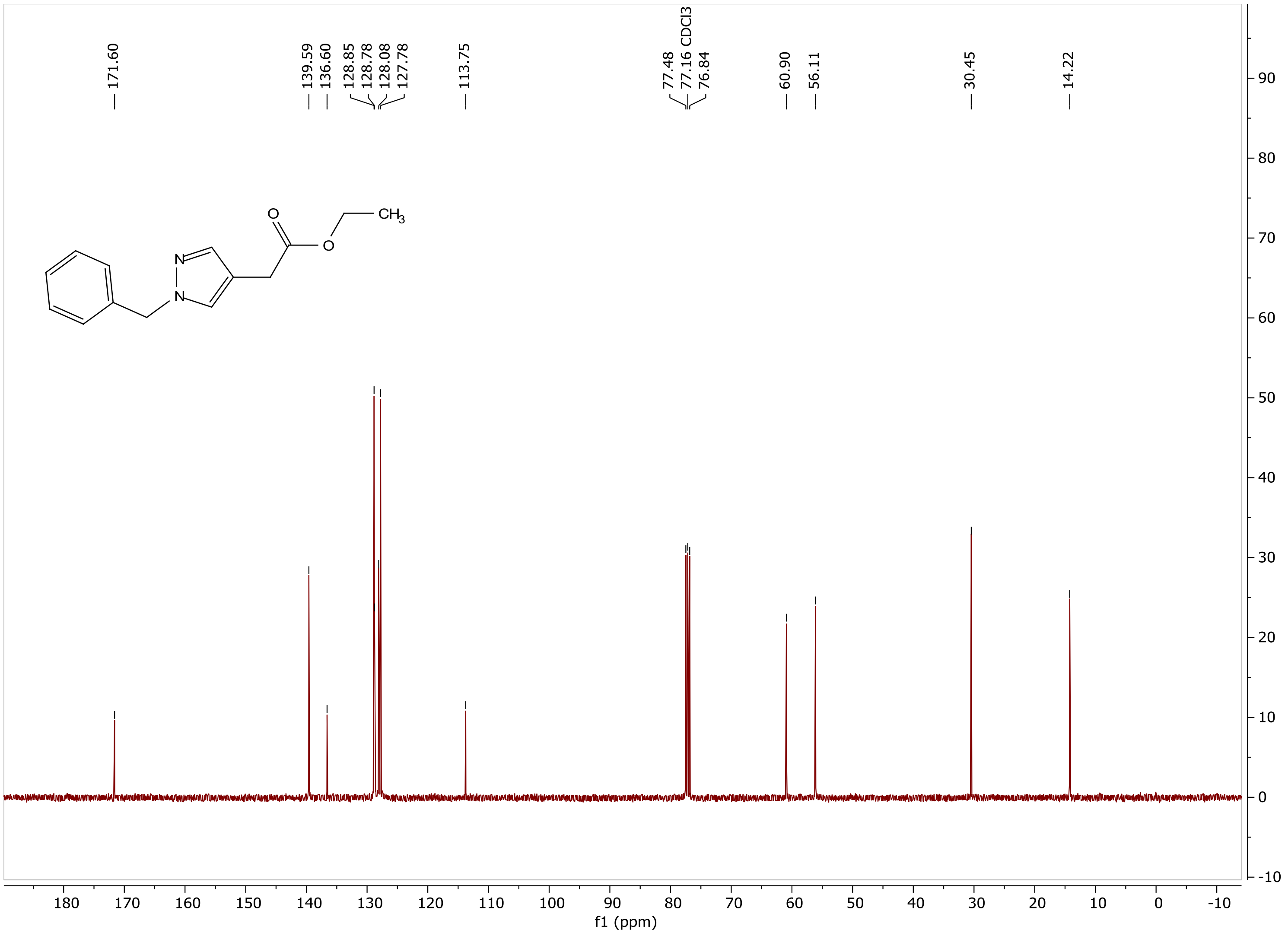
171.60
139.59
136.60
128.85
128.78
128.08
127.78
113.75
77.48
77.16 CDCl3
76.84
60.90
56.11
30.45
14.22
f1 (ppm)

Ethyl 2-(3-(trifluoromethyl)-1H-pyrazol-4-yl)acetate **(2m)** *($CDCl_3$, ^{1}H-NMR: 500 MHz, ^{13}C-NMR: 126 MHz)*

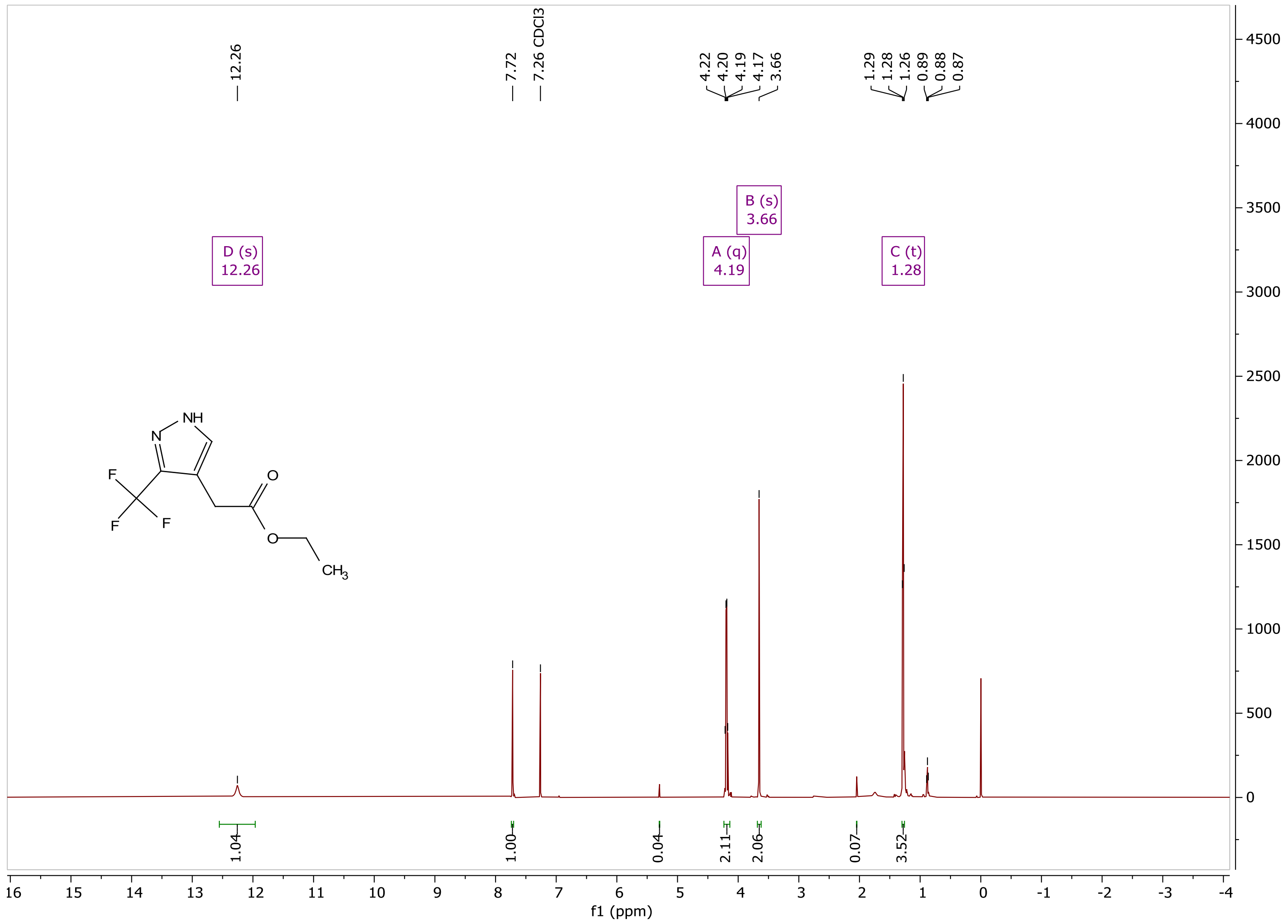

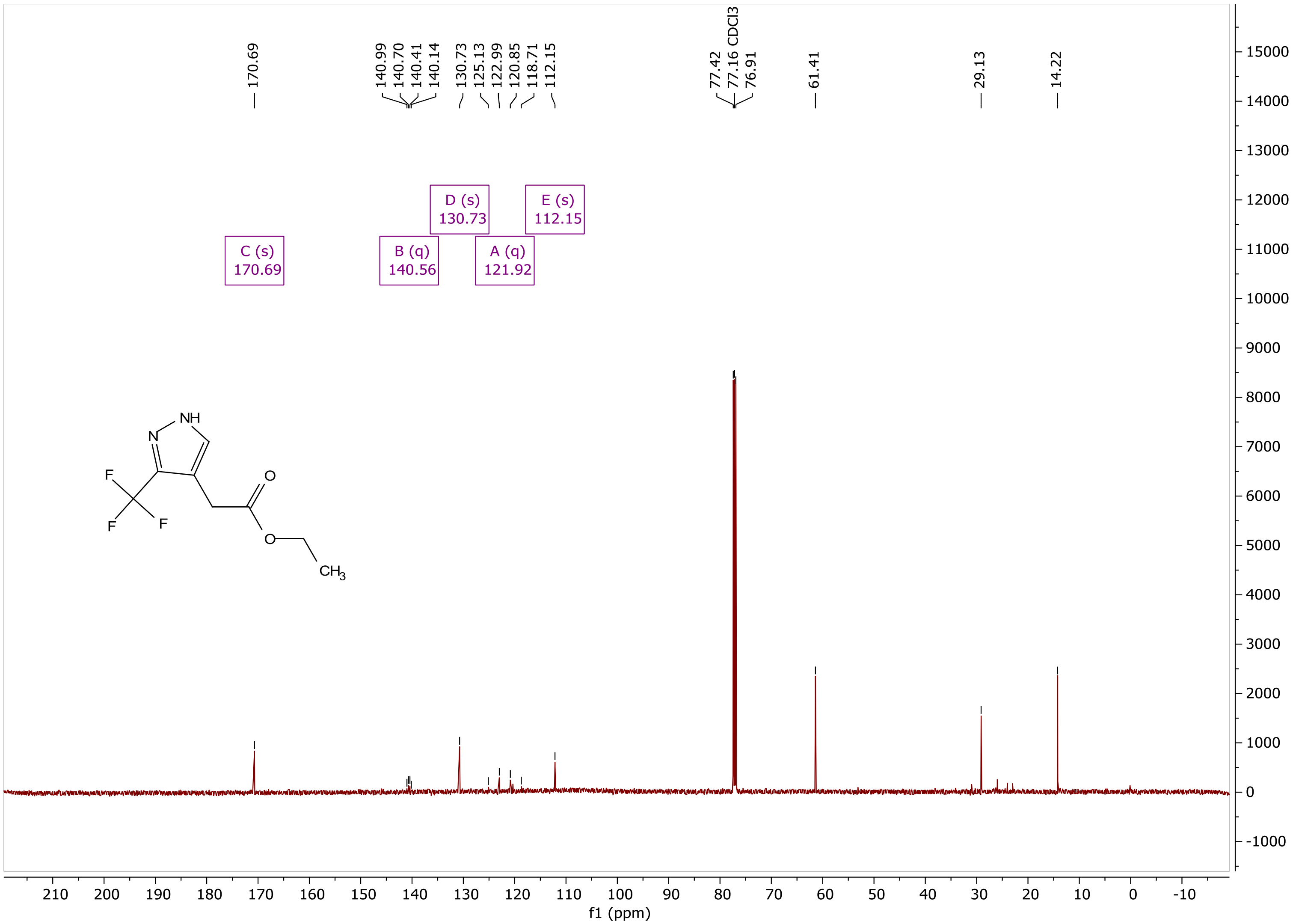
170.69
140.99
140.70
140.41
140.14
130.73
125.13
122.99
120.85
118.71
112.15
77.42
77.16 CDCl3
76.91
61.41
29.13
14.22
D (s) 130.73
E (s) 112.15
C (s) 170.69
B (q) 140.56
A (q) 121.92
NH
N
F
F
F
O
O
CH3
f1 (ppm)

Ethyl 2-(1H-pyrazol-4-yl)acetate **(2n)** *($CDCl_3$, ^{1}H-NMR: 400 MHz, ^{13}C-NMR: 101 MHz)*

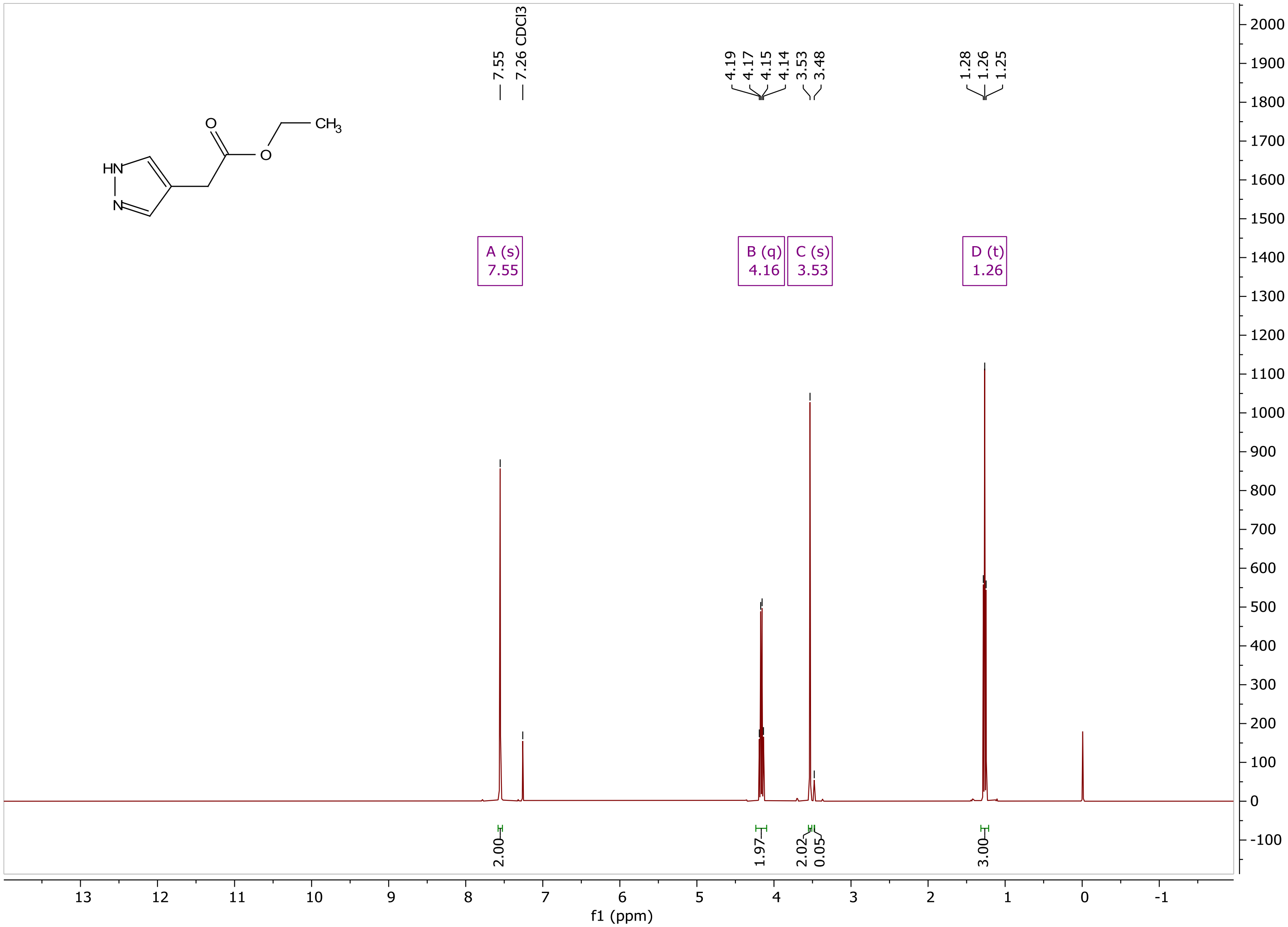

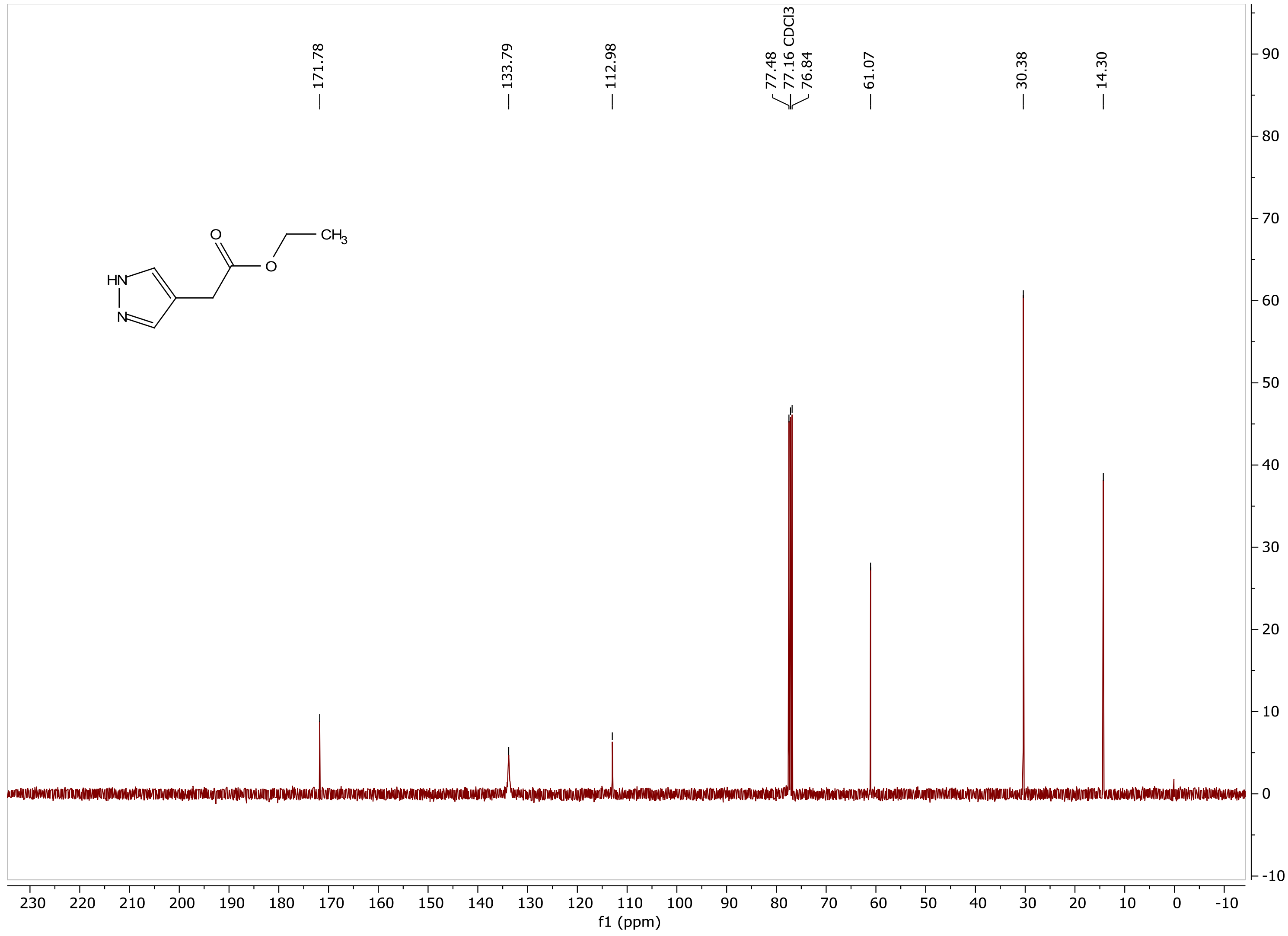
171.78
133.79
112.98
77.48
77.16 CDCl3
76.84
61.07
30.38
14.30
HN
N
O
O
CH3
f1 (ppm)

Ethyl 2-(1-ethyl-1H-pyrazol-4-yl)acetate **(2o)** *($CDCl_3$, ^{1}H-NMR: 500 MHz, ^{13}C-NMR: 126 MHz)*

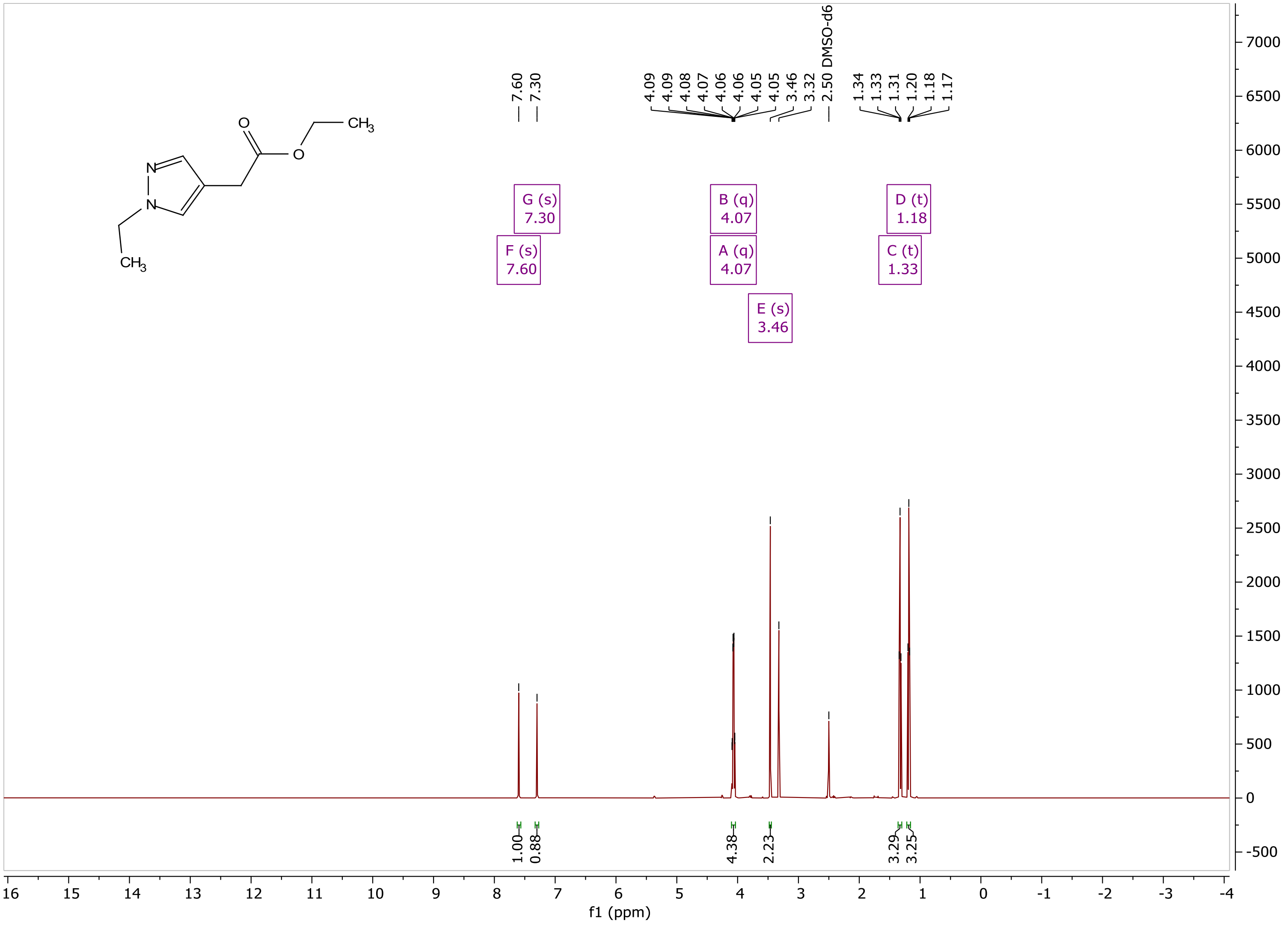

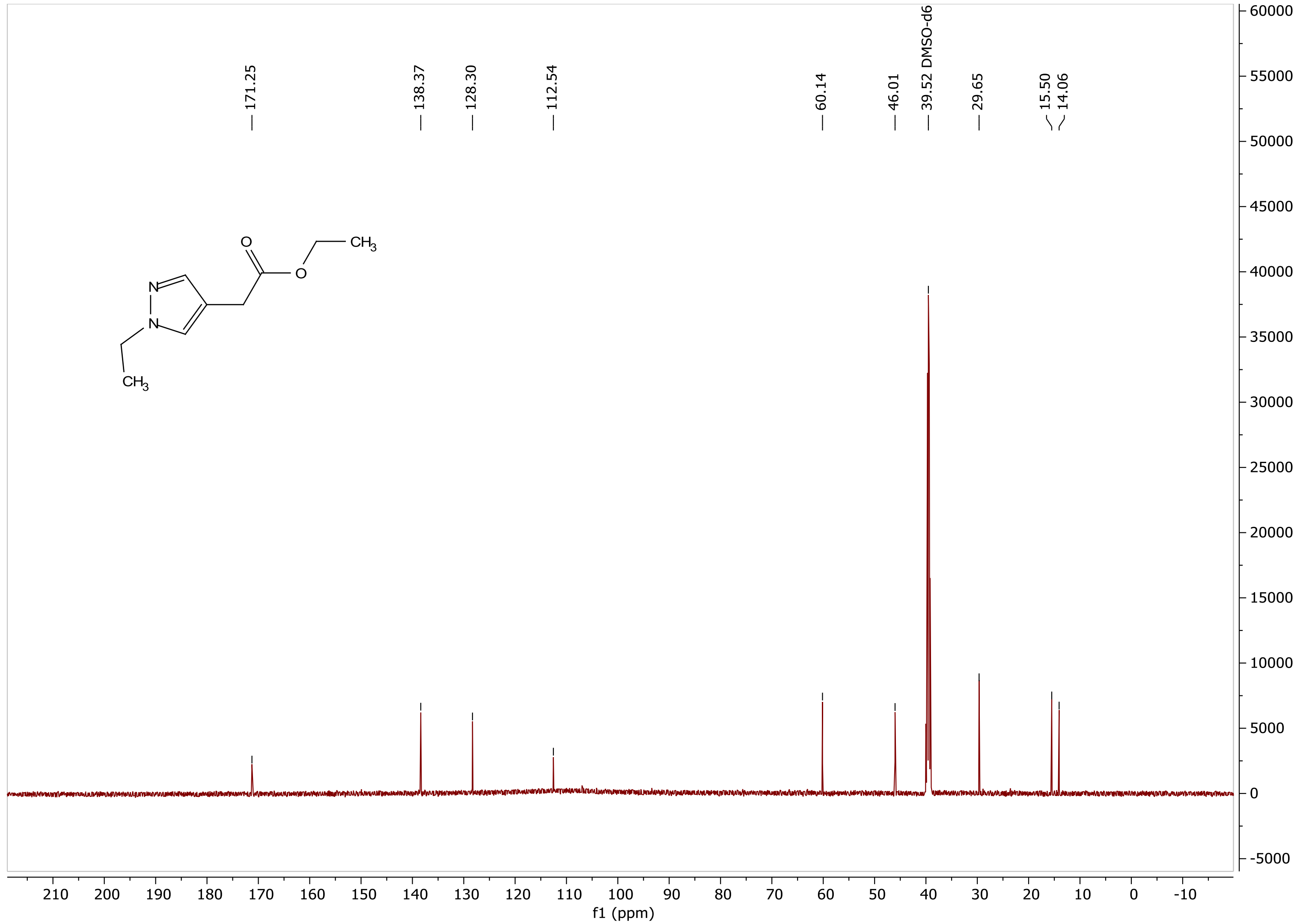

171.25
138.37
128.30
112.54
60.14
46.01
39.52 DMSO-d6
29.65
15.50
14.06
60000
55000
50000
45000
40000
35000
30000
25000
20000
15000
10000
5000
0
-5000
210 200 190 180 170 160 150 140 130 120 110 100 90 80 70 60 50 40 30 20 10 0 -10
f1 (ppm)
O
O
CH3
N
N
CH3

Ethyl 2-(1H-indazol-3-yl)acetate **(2p)** *($CDCl_3$, ^{1}H-NMR: 400 MHz, ^{13}C-NMR: 101 MHz)*

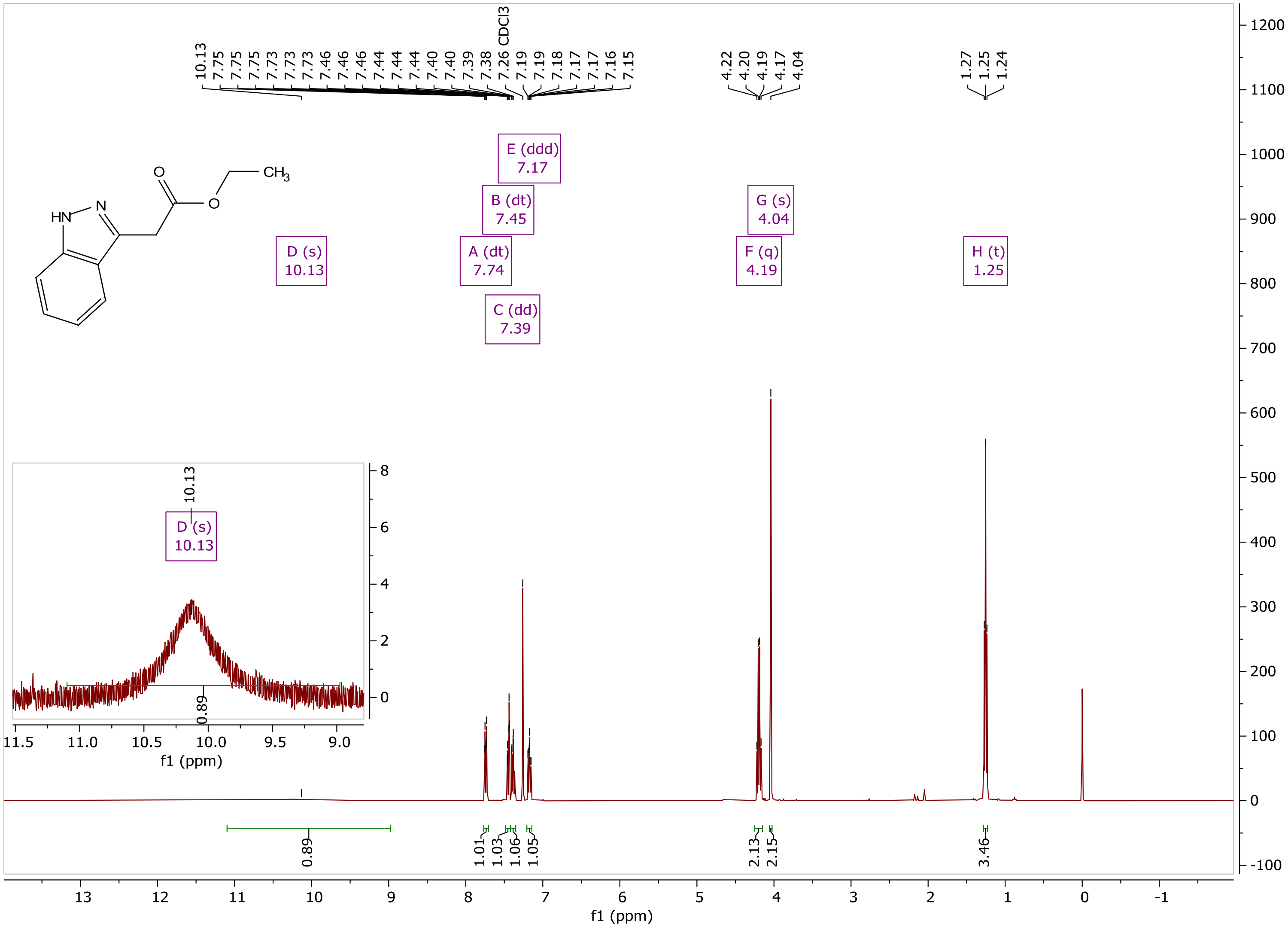

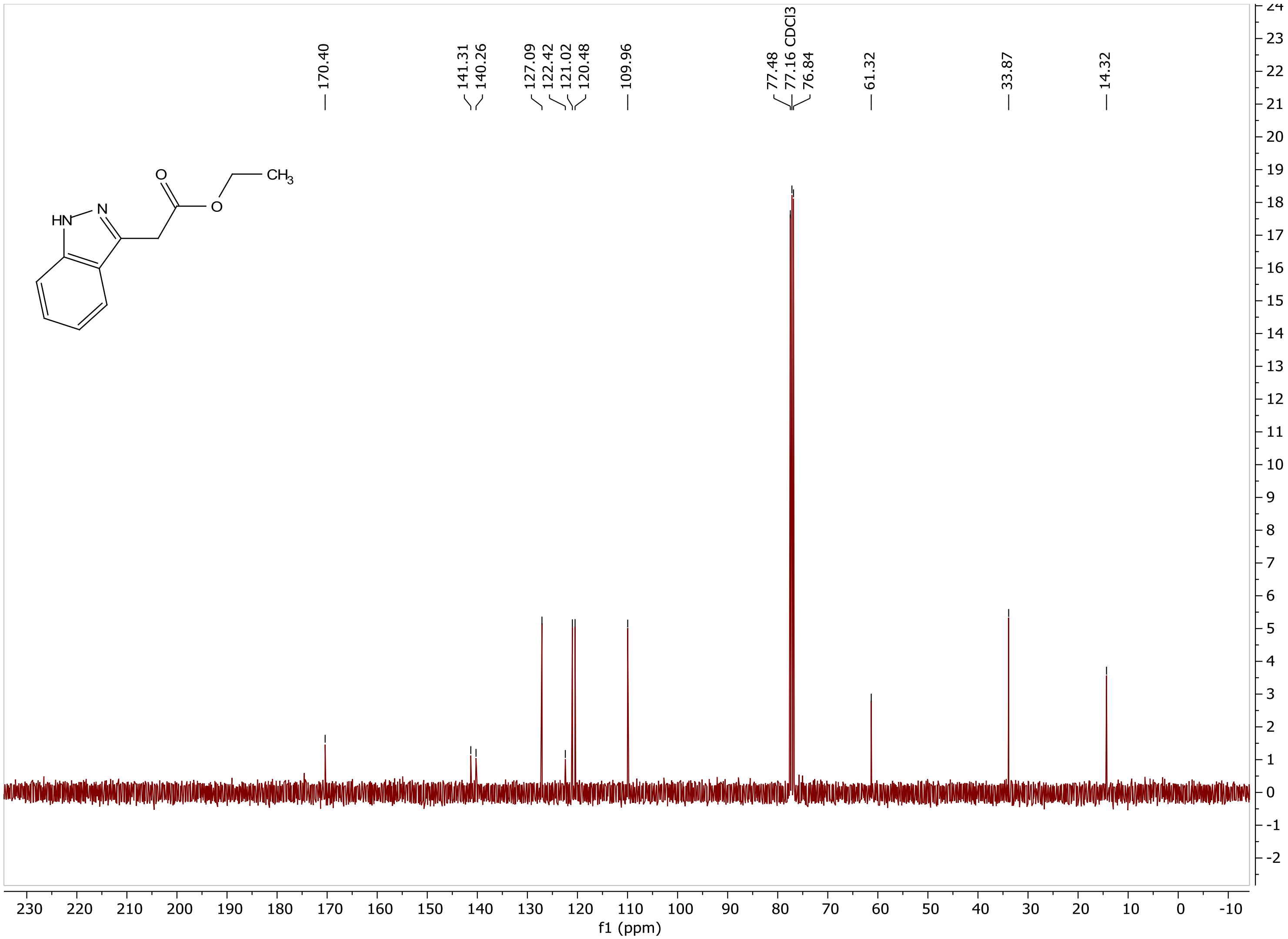

170.40
141.31
140.26
127.09
122.42
121.02
120.48
109.96
77.48
77.16 CDCl3
76.84
61.32
33.87
14.32
O
CH3
HN
N
O
f1 (ppm)

Ethyl 2-(1-methyl-1H-indazol-3-yl)acetate ***(2q)*** *($CDCl_3$, ^{1}H-NMR: 400 MHz, ^{13}C-NMR: 101 MHz)*

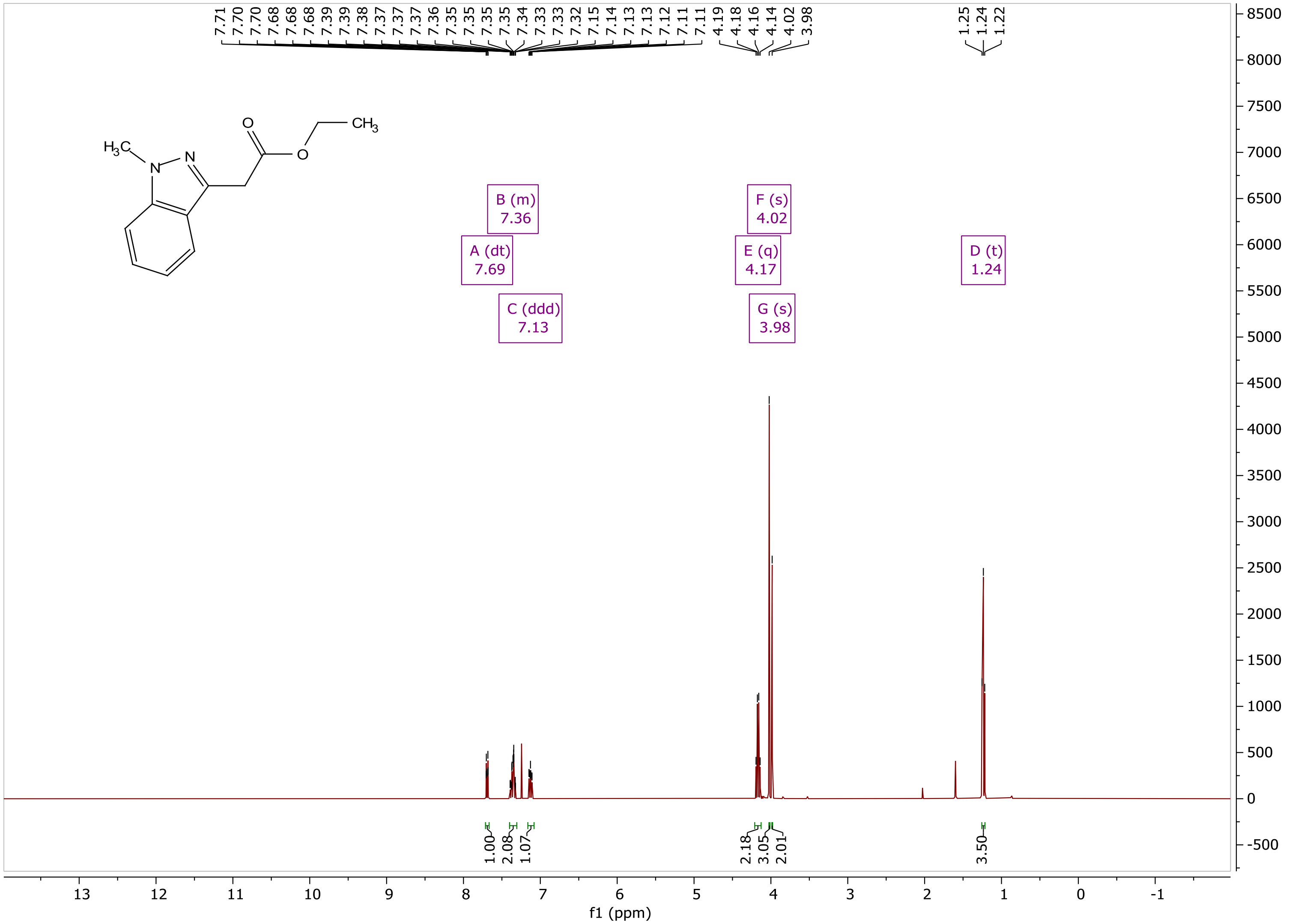

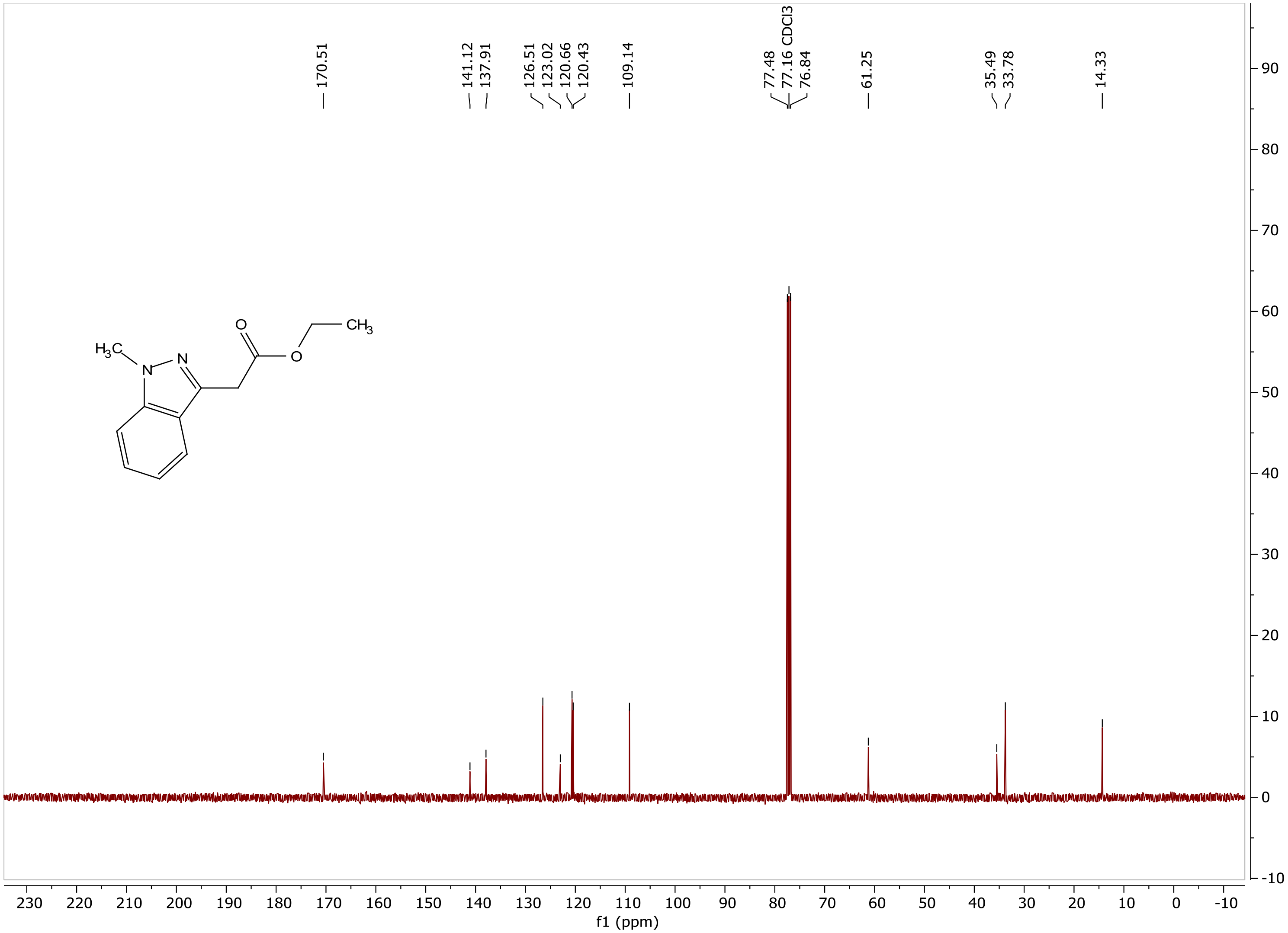
170.51
141.12
137.91
126.51
123.02
120.66
120.43
109.14
77.48
77.16 CDCl3
76.84
61.25
35.49
33.78
14.33
f1 (ppm)

Ethyl 2-(1H-indazol-5-yl)acetate ***(2r)*** *($CDCl_3$, ^{1}H-NMR: 400 MHz, ^{13}C-NMR: 101 MHz)*

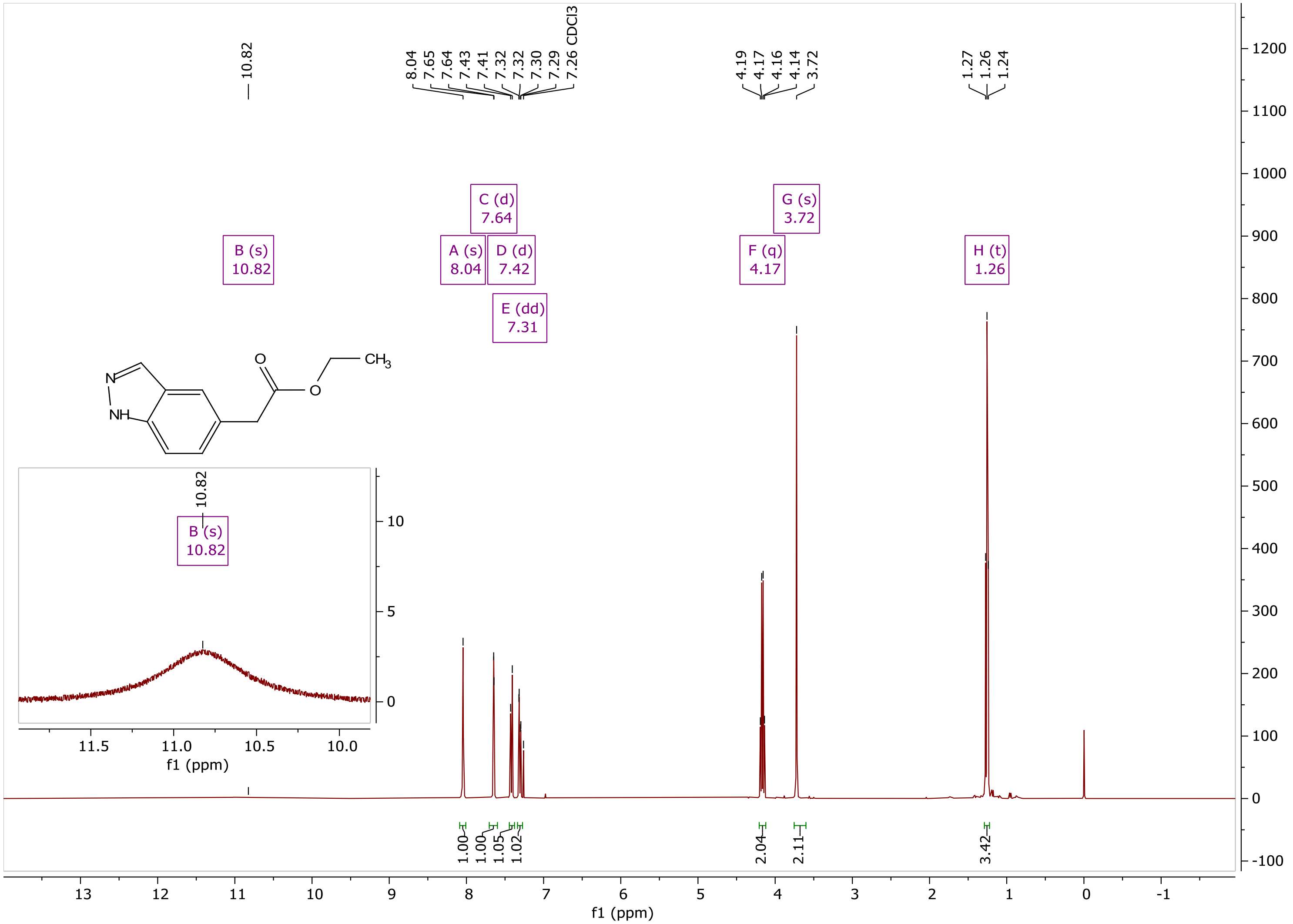

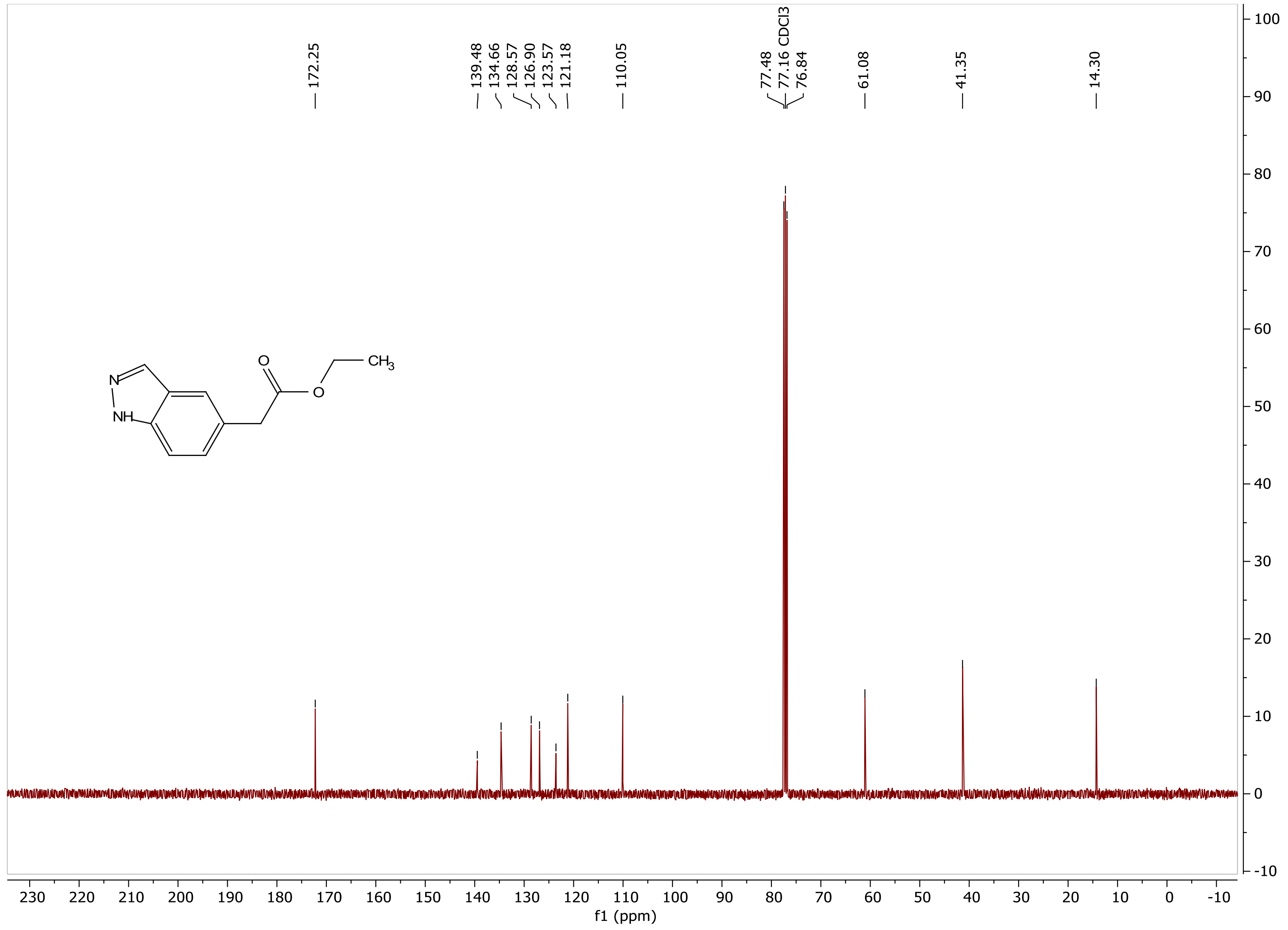

172.25
139.48
134.66
128.57
126.90
123.57
121.18
110.05
77.48
77.16 CDCl3
76.84
61.08
41.35
14.30
N
NH
O
O
CH3
f1 (ppm)

Ethyl 2-(1-methyl-1H-benzo[d]imidazol-2-yl)acetate **(2t)** ($CDCl_3$, ¹H-NMR: 400 MHz, ¹³C-NMR: 101 MHz)

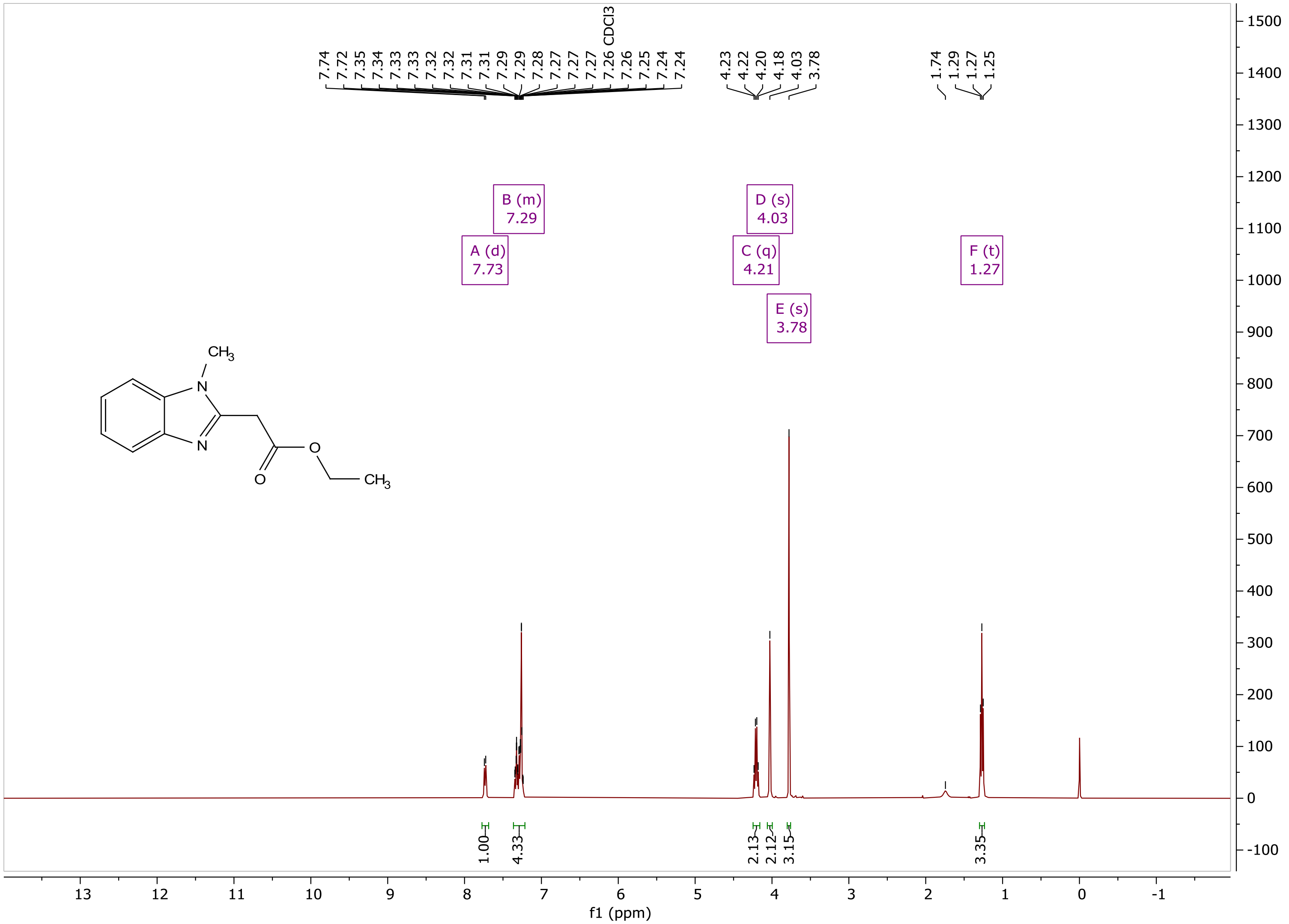

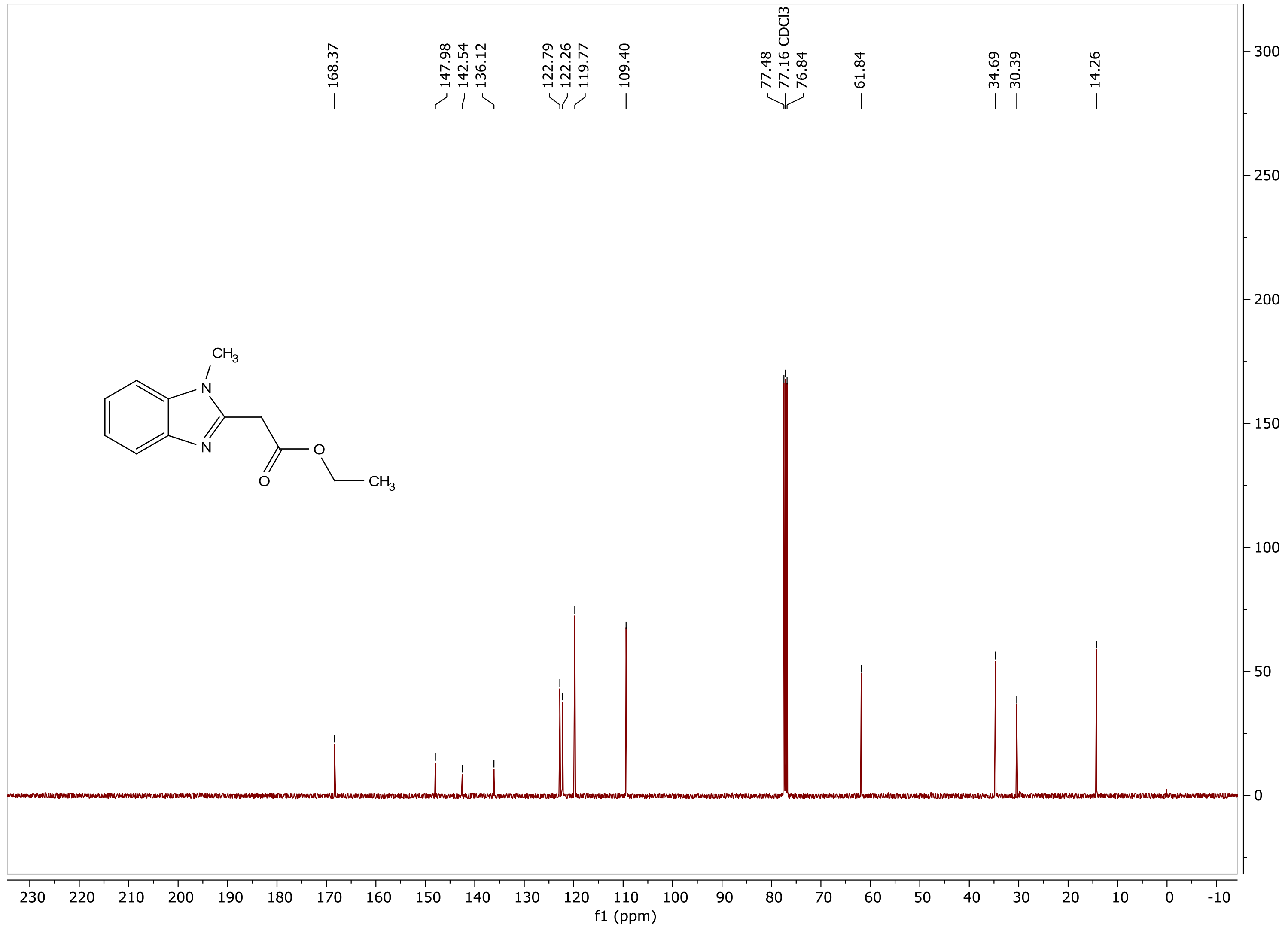
168.37
147.98
142.54
136.12
122.79
122.26
119.77
109.40
77.48
77.16 CDCl3
76.84
61.84
34.69
30.39
14.26
f1 (ppm)

Ethyl 2-(1-methyl-1H-imidazol-5-yl)acetate ***(2u)*** *($CDCl_3$, ^{1}H-NMR: 400 MHz, ^{13}C-NMR: 101 MHz)*

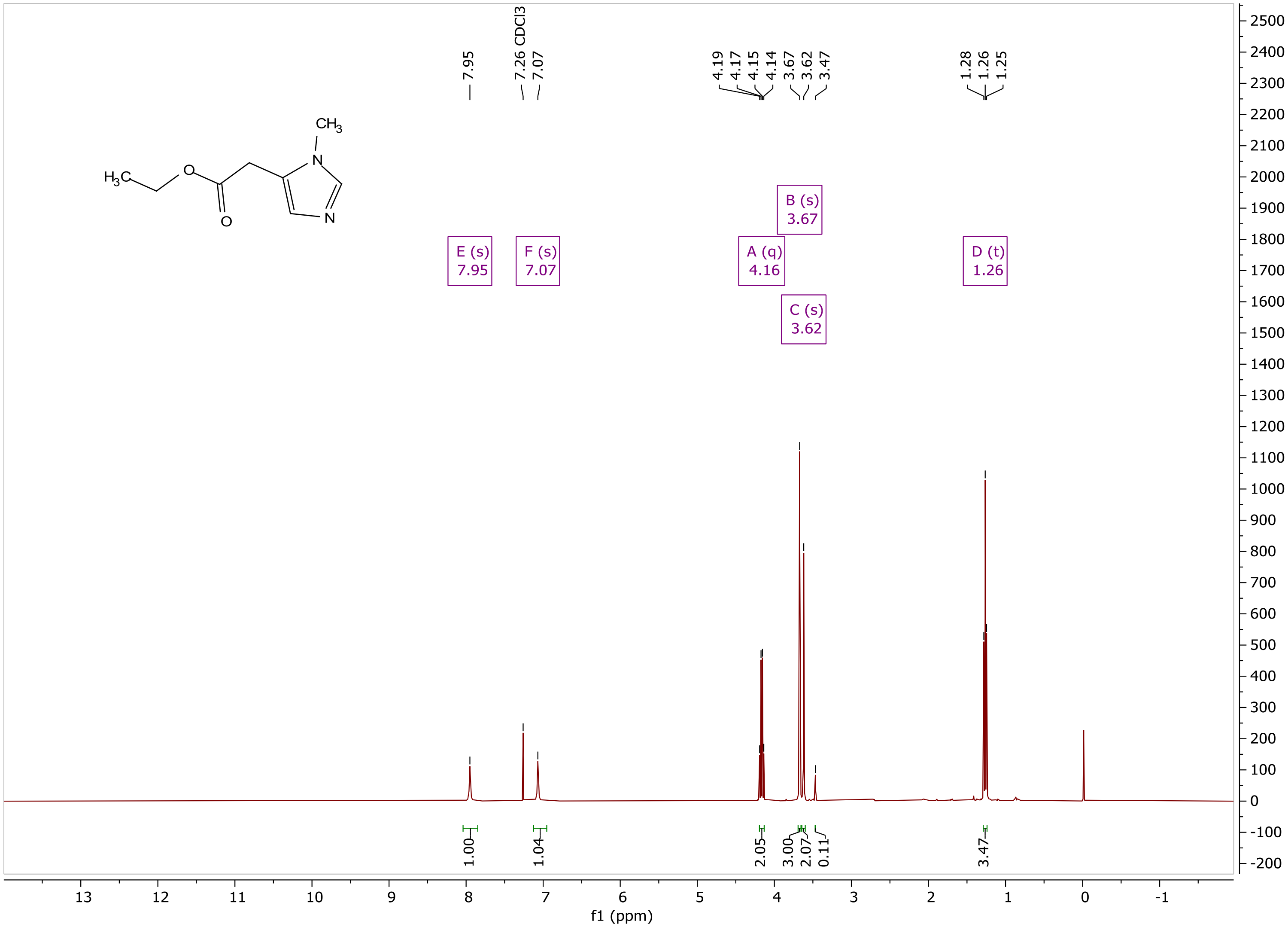

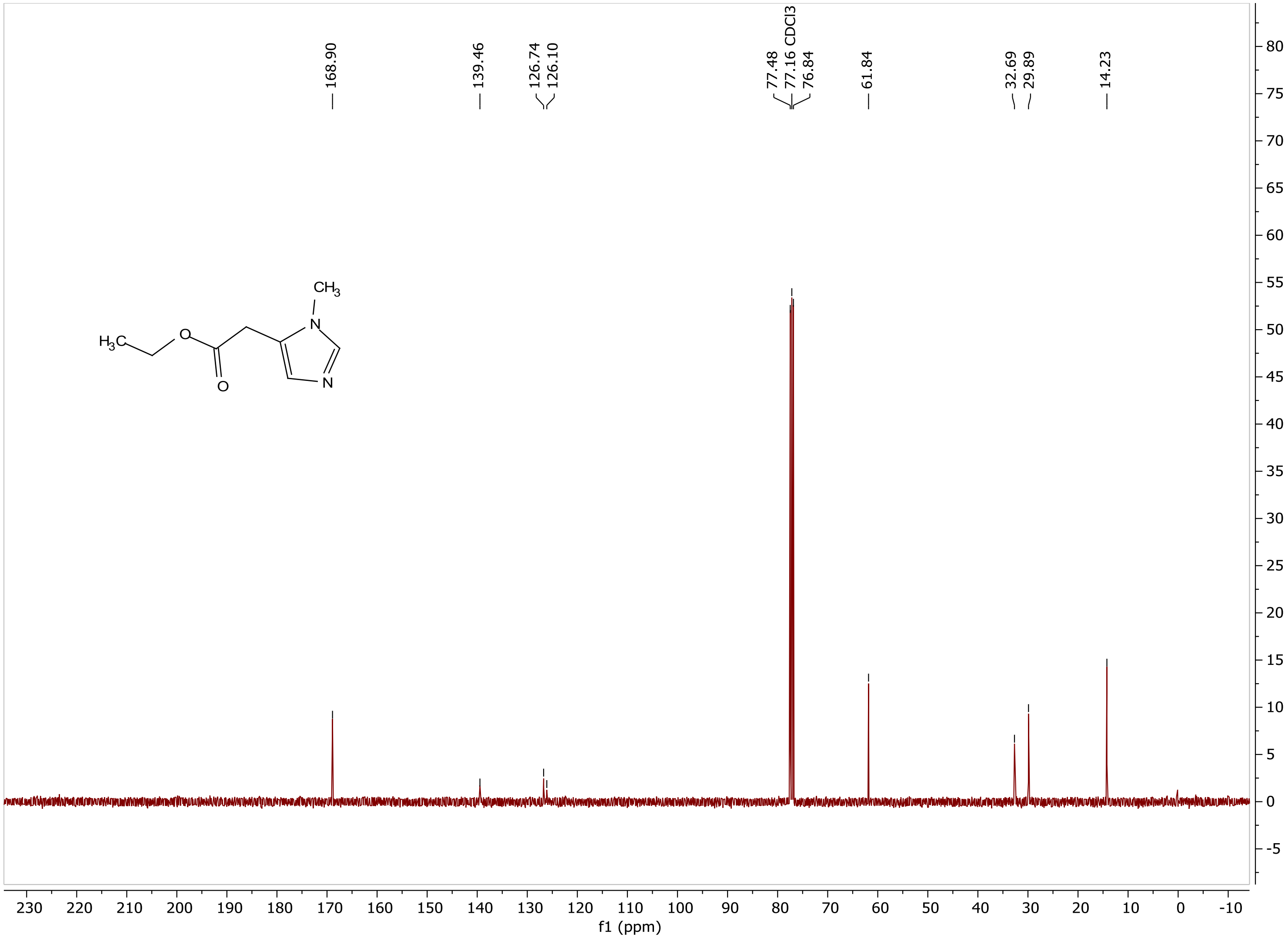

168.90
139.46
126.74
126.10
77.48
77.16 CDCl3
76.84
61.84
32.69
29.89
14.23
CH3
N
N
O
O
H3C
230 220 210 200 190 180 170 160 150 140 130 120 110 100 90 80 70 60 50 40 30 20 10 0 -10
f1 (ppm)
80 75 70 65 60 55 50 45 40 35 30 25 20 15 10 5 0 -5

NMR spectra of the intermediates for the oxime formation

Ethyl 2-(furan-3-yl)-2-oxoacetate ***(4j)*** *($CDCl_3$, ^{1}H-NMR: 400 MHz, ^{13}C-NMR: 101 MHz)*

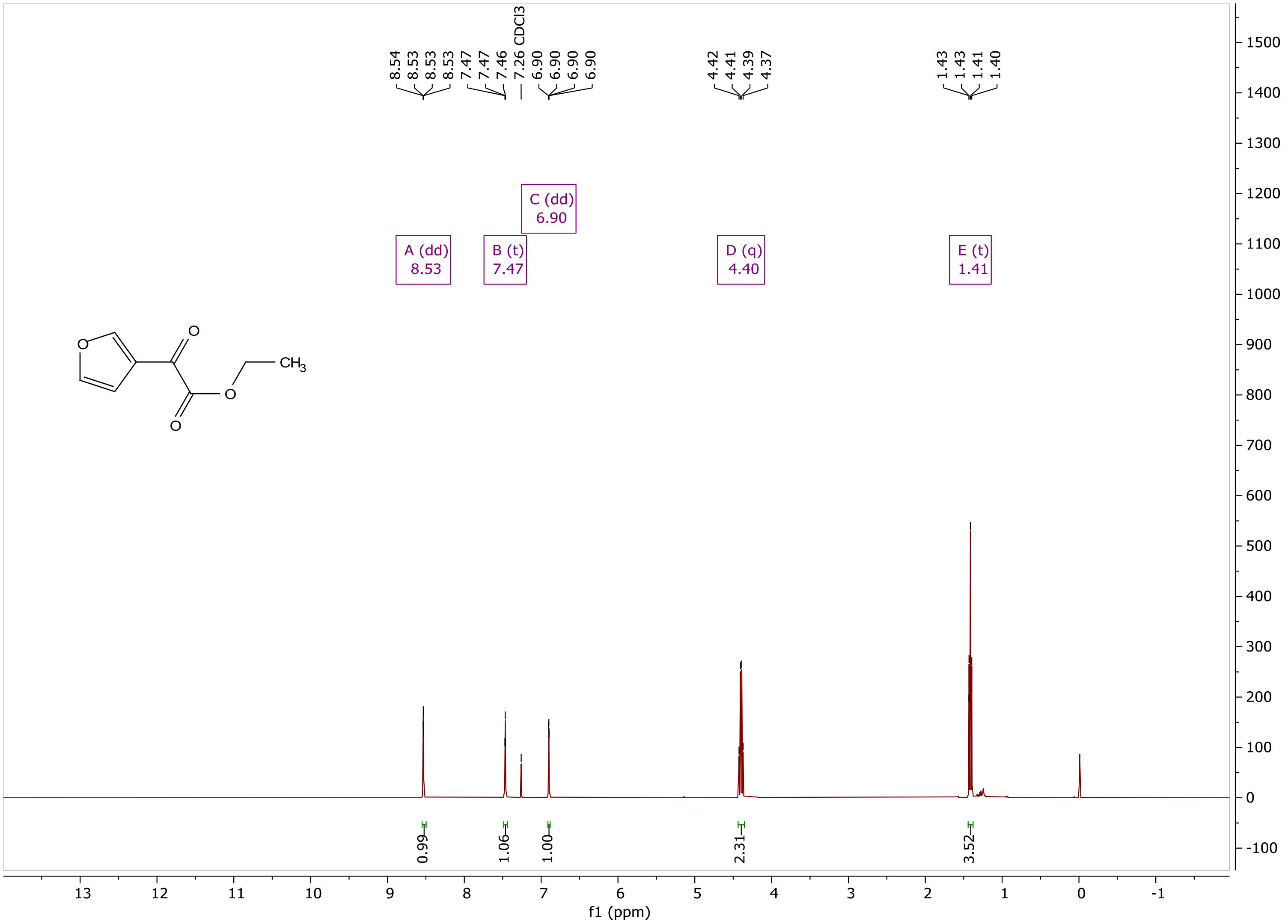

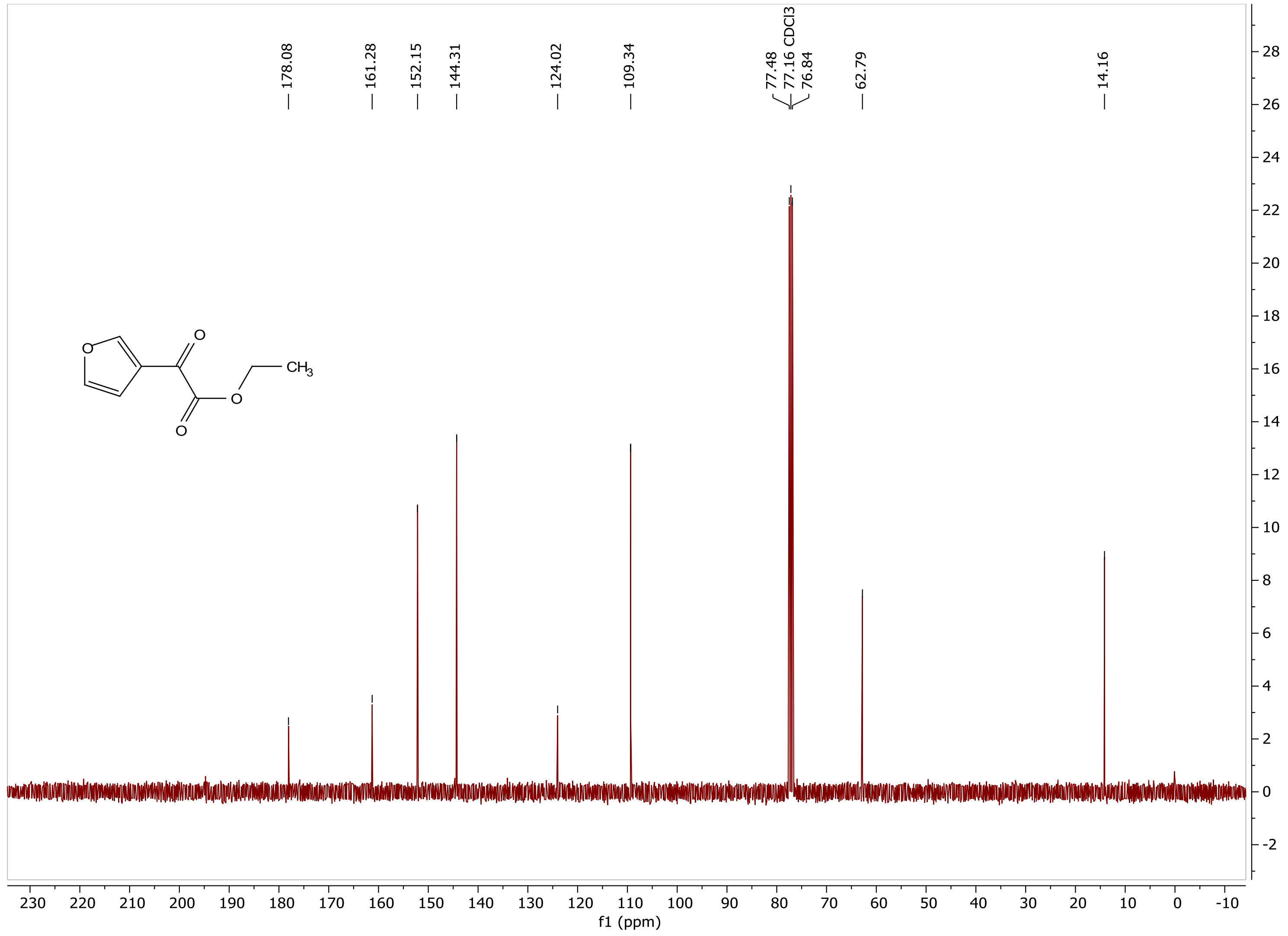
178.08
161.28
152.15
144.31
124.02
109.34
77.48
77.16 CDCl3
76.84
62.79
14.16
O
O
O
O
CH3
f1 (ppm)

2-(1H-Indazol-5-yl)acetic acid ***(3r)*** *- (CD_3OD, ^{1}H-NMR: 400 MHz, ^{13}C-NMR: 101 MHz)*

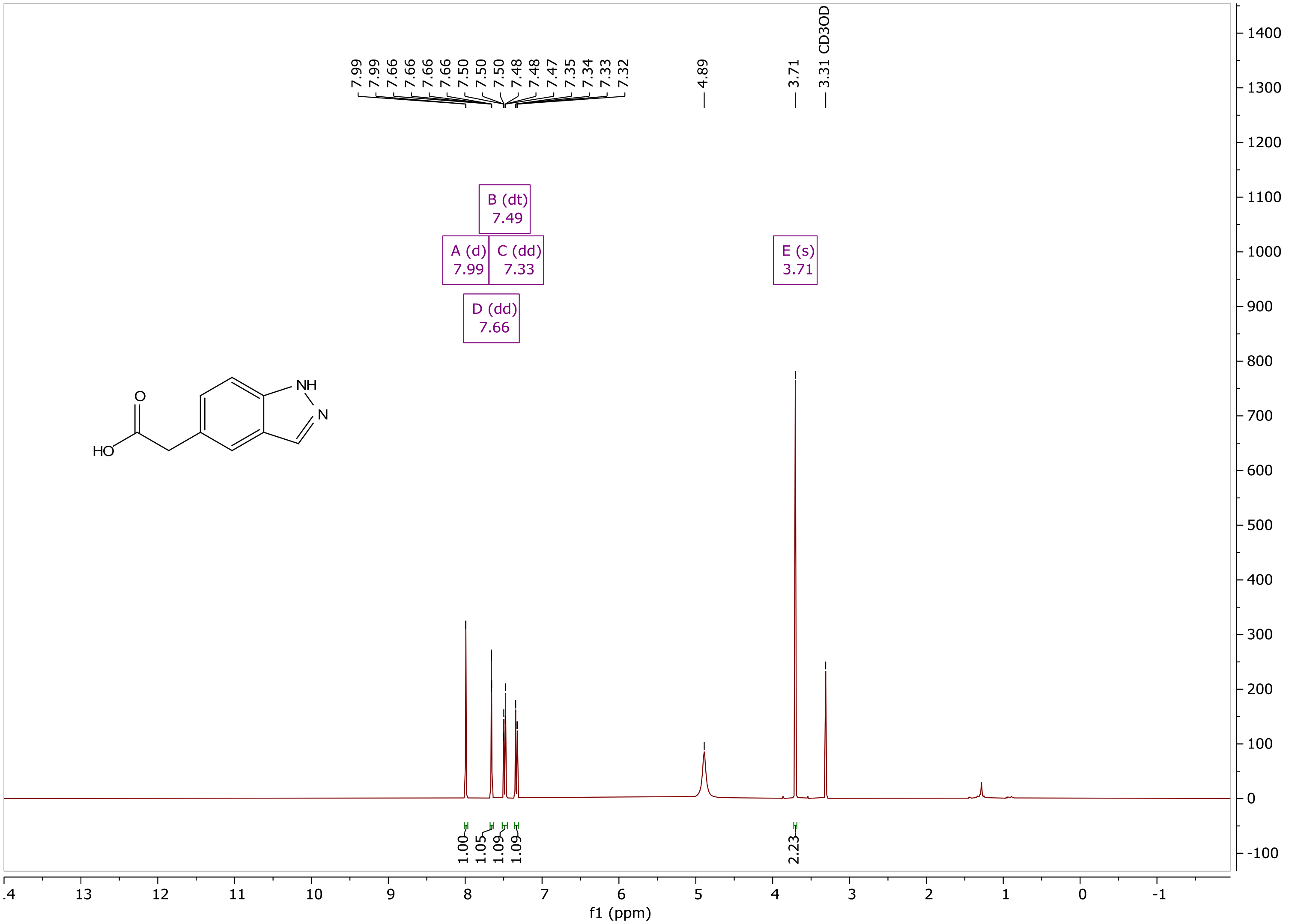

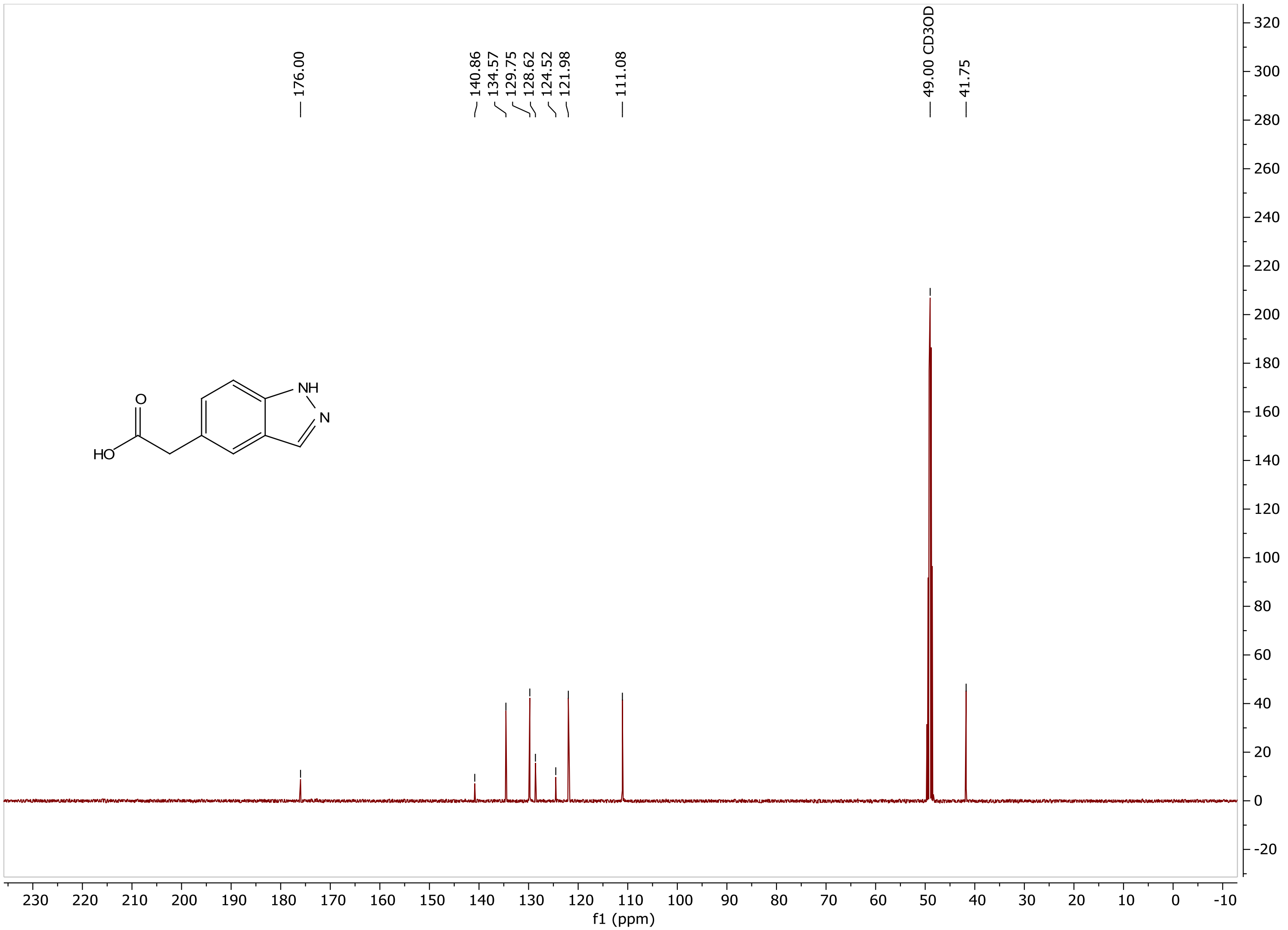
176.00
140.86
134.57
129.75
128.62
124.52
121.98
111.08
49.00 CD3OD
41.75
NH
N
O
HO
f1 (ppm)

Ethyl 2-(1H-indazol-5-yl)-2-oxoacetate ***(4r)*** *(DMSO-d^6, ^{1}H-NMR: 400 MHz, ^{13}C-NMR: 101 MHz)*

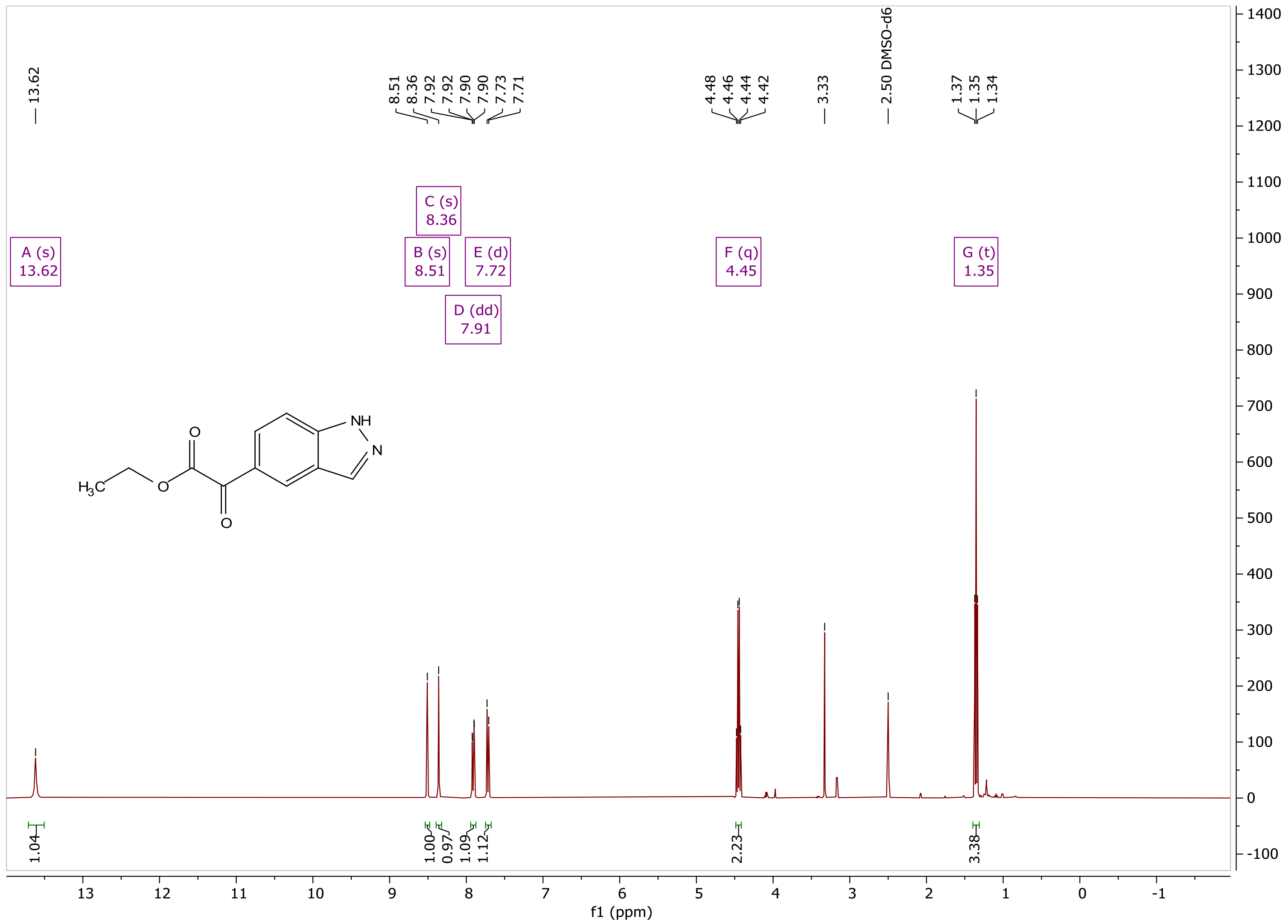

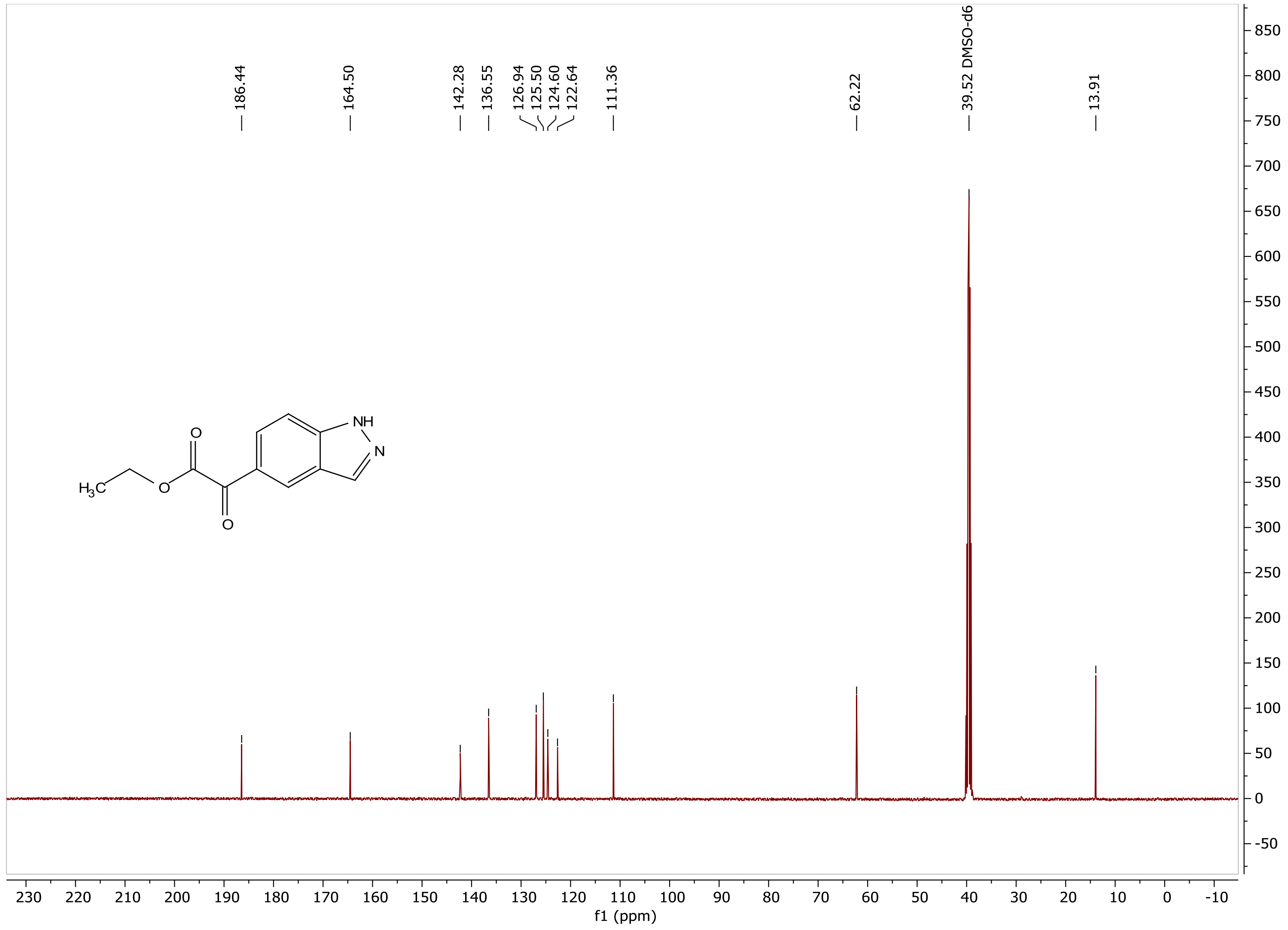
186.44
164.50
142.28
136.55
126.94
125.50
124.60
122.64
111.36
62.22
39.52 DMSO-d6
13.91
NH
N
O
O
O
H3C
f1 (ppm)

NMR spectra oximes

Oximes were synthesized as a mixture of E/Z isomers and double peaks in the spectra are due to E/Z isomerism.

Ethyl 2-(hydroxyimino)-2-(thiazol-2-yl)acetate - mixture ***(5b)*** *- ($CDCl_3$, ^{1}H-NMR: 400 MHz, ^{13}C-NMR: 101 MHz)*

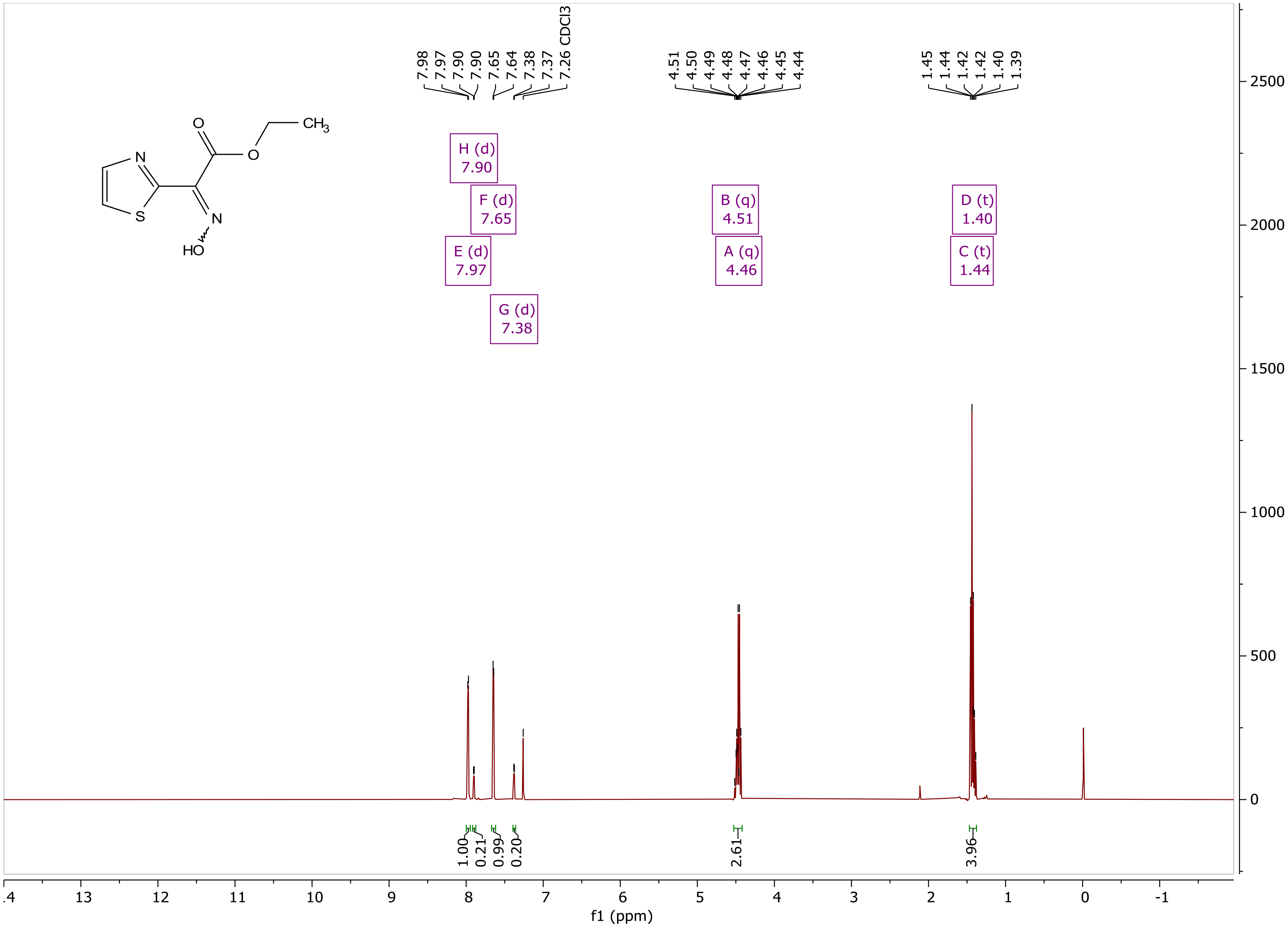

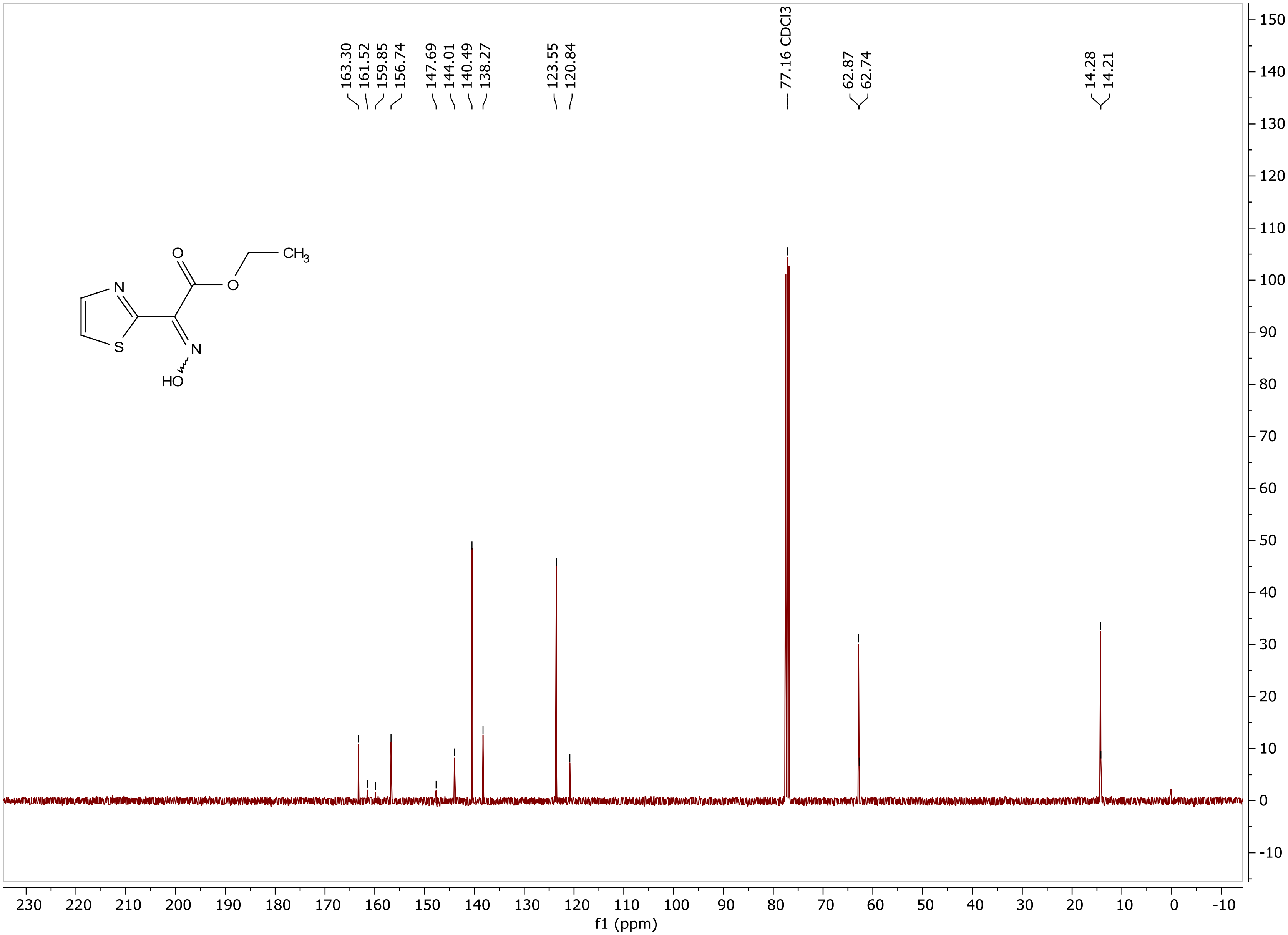

163.30
161.52
159.85
156.74
147.69
144.01
140.49
138.27
123.55
120.84
77.16 CDCl3
62.87
62.74
14.28
14.21
O
CH3
N
O
S
N
HO
f1 (ppm)

Ethyl 2-(hydroxyimino)-2-(thiazol-2-yl)acetate – main isomer - ($CDCl_3$, ^{1}H-NMR: 400 MHz, ^{13}C-NMR: 101 MHz)

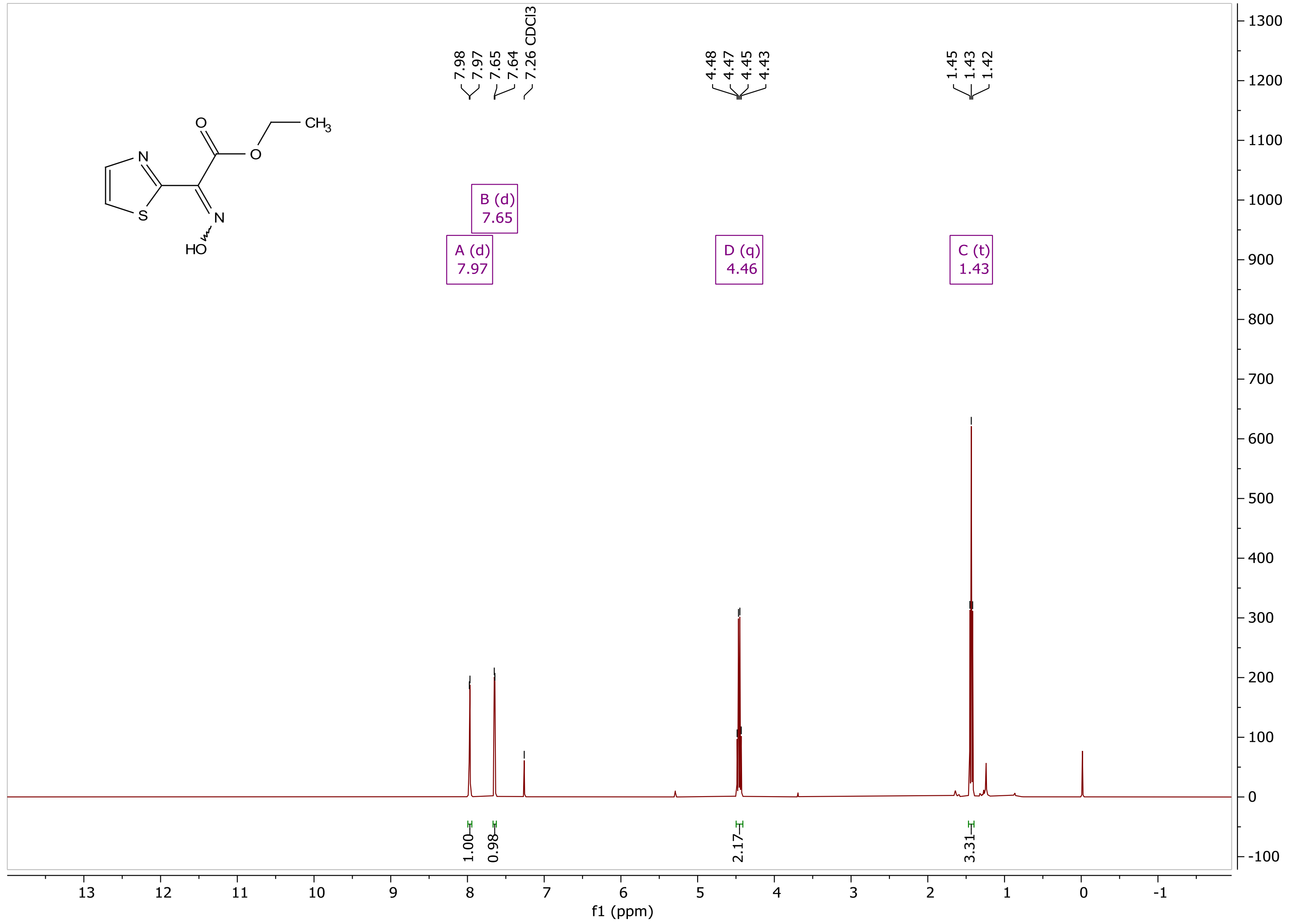

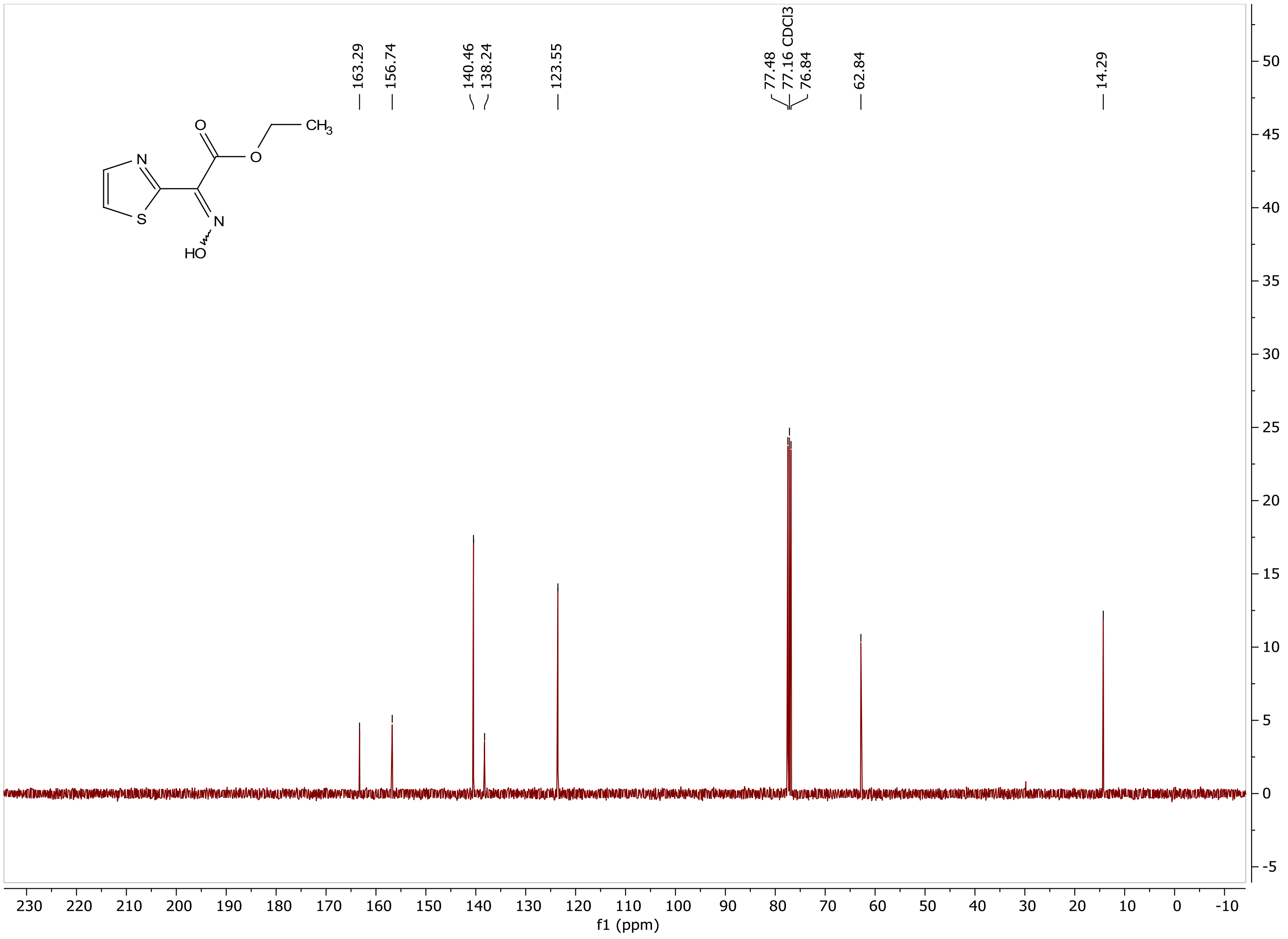

163.29
156.74
140.46
138.24
123.55
77.48
77.16 CDCl3
76.84
62.84
14.29
O
O
CH3
N
S
N
HO
f1 (ppm)

Ethyl 2-(benzo[d]thiazol-2-yl)-2-(hydroxyimino)acetate **(5i)** *($CDCl_3$, ^{1}H-NMR: 400 MHz, ^{13}C-NMR: 101 MHz)*

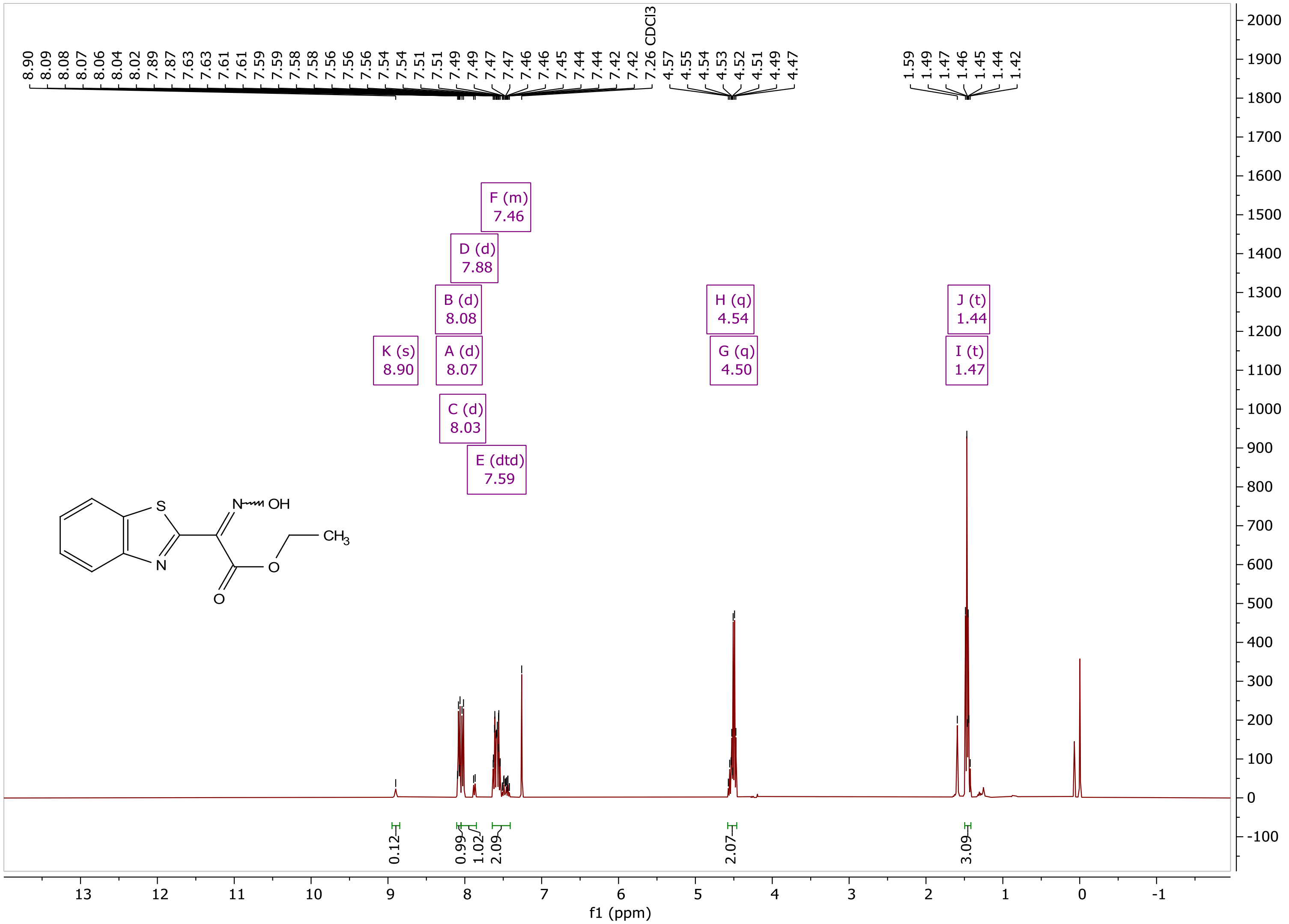

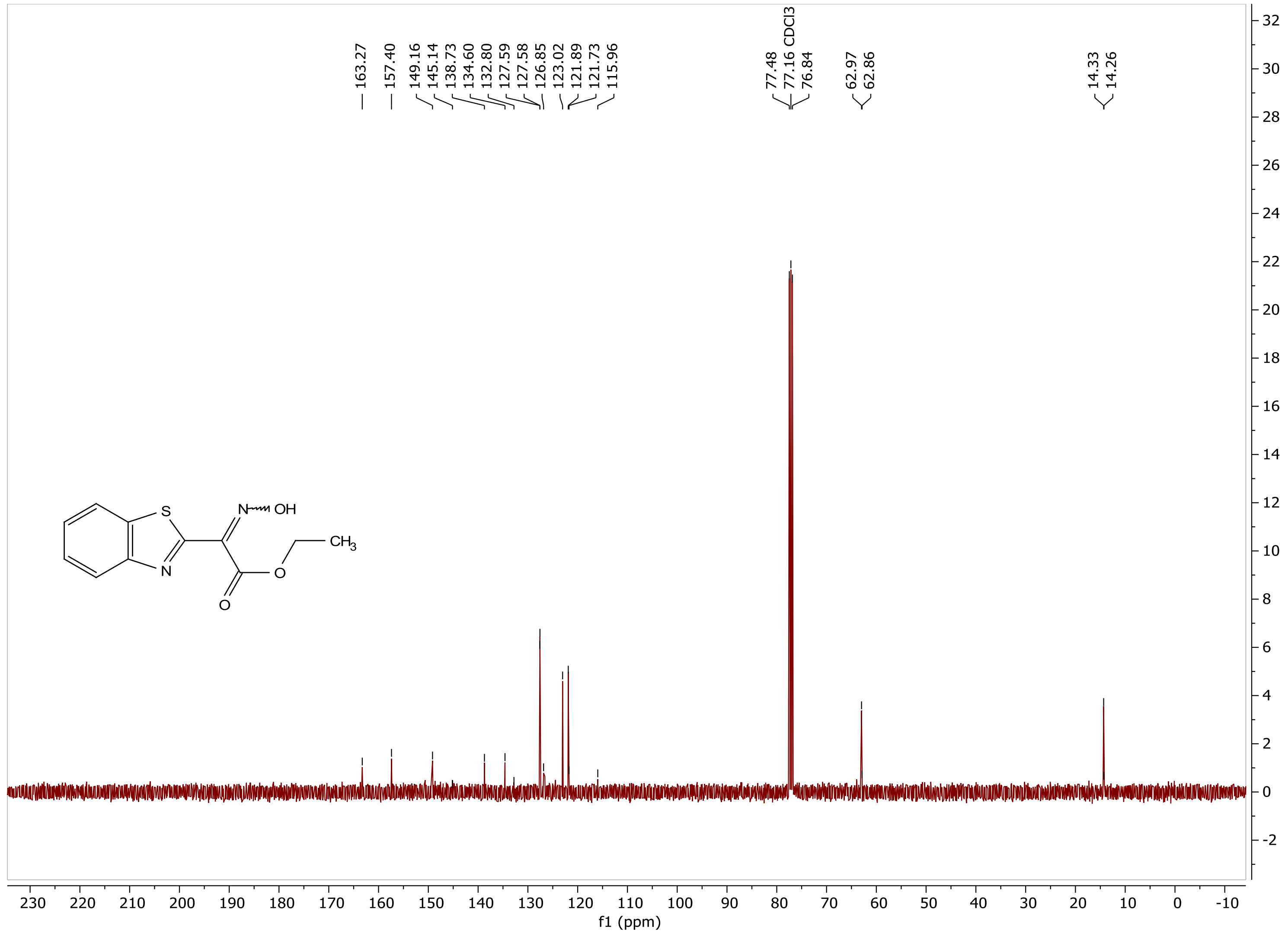
163.27
157.40
149.16
145.14
138.73
134.60
132.80
127.59
127.58
126.85
123.02
121.89
121.73
115.96
77.48
77.16 CDCl3
76.84
62.97
62.86
14.33
14.26
N OH
S
N
CH3
O
O
f1 (ppm)

Ethyl 2-(furan-3-yl)-2-(hydroxyimino)acetate **(5j)** ($CDCl_3$, ^{1}H-NMR: 400 MHz, ^{13}C-NMR: 101 MHz)

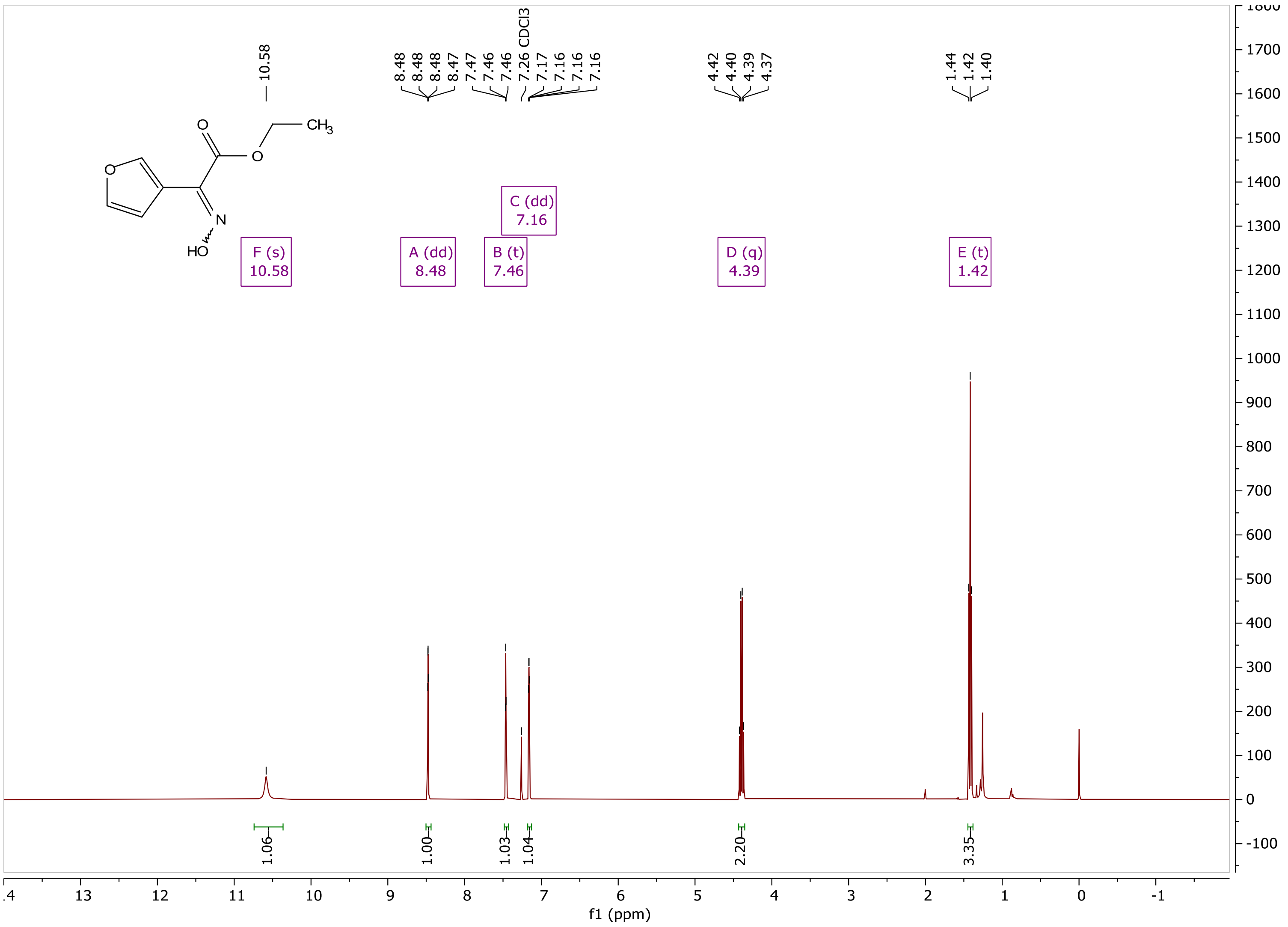

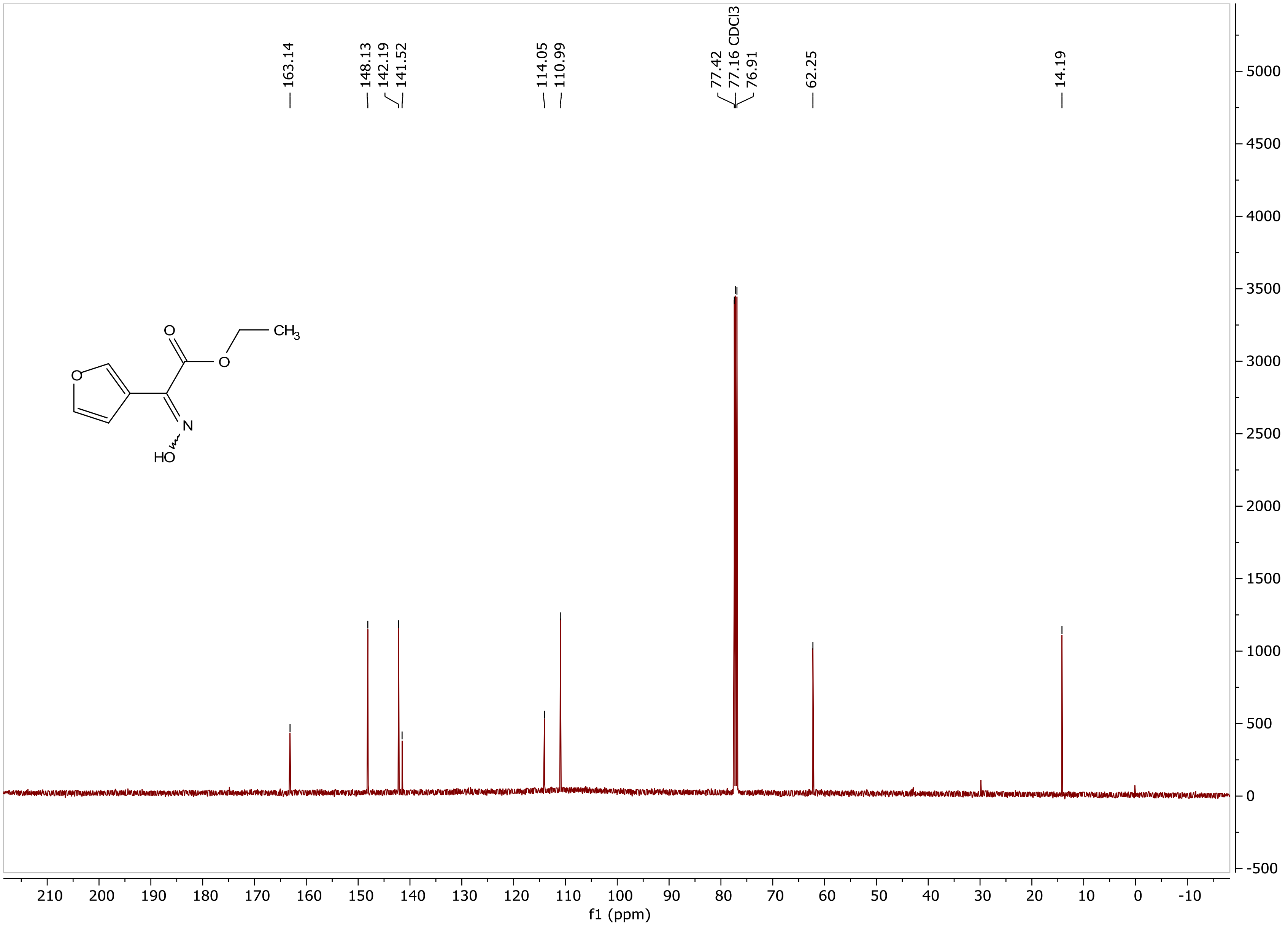
163.14
148.13
142.19
141.52
114.05
110.99
77.42
77.16 CDCl3
76.91
62.25
14.19
f1 (ppm)
O
CH3
O
O
N
HO

Ethyl 2-(1-benzyl-1H-pyrazol-4-yl)-2-(hydroxyimino)acetate ***(5l)*** *($CDCl_3$, ^{1}H-NMR: 400 MHz, ^{13}C-NMR: 101 MHz)*

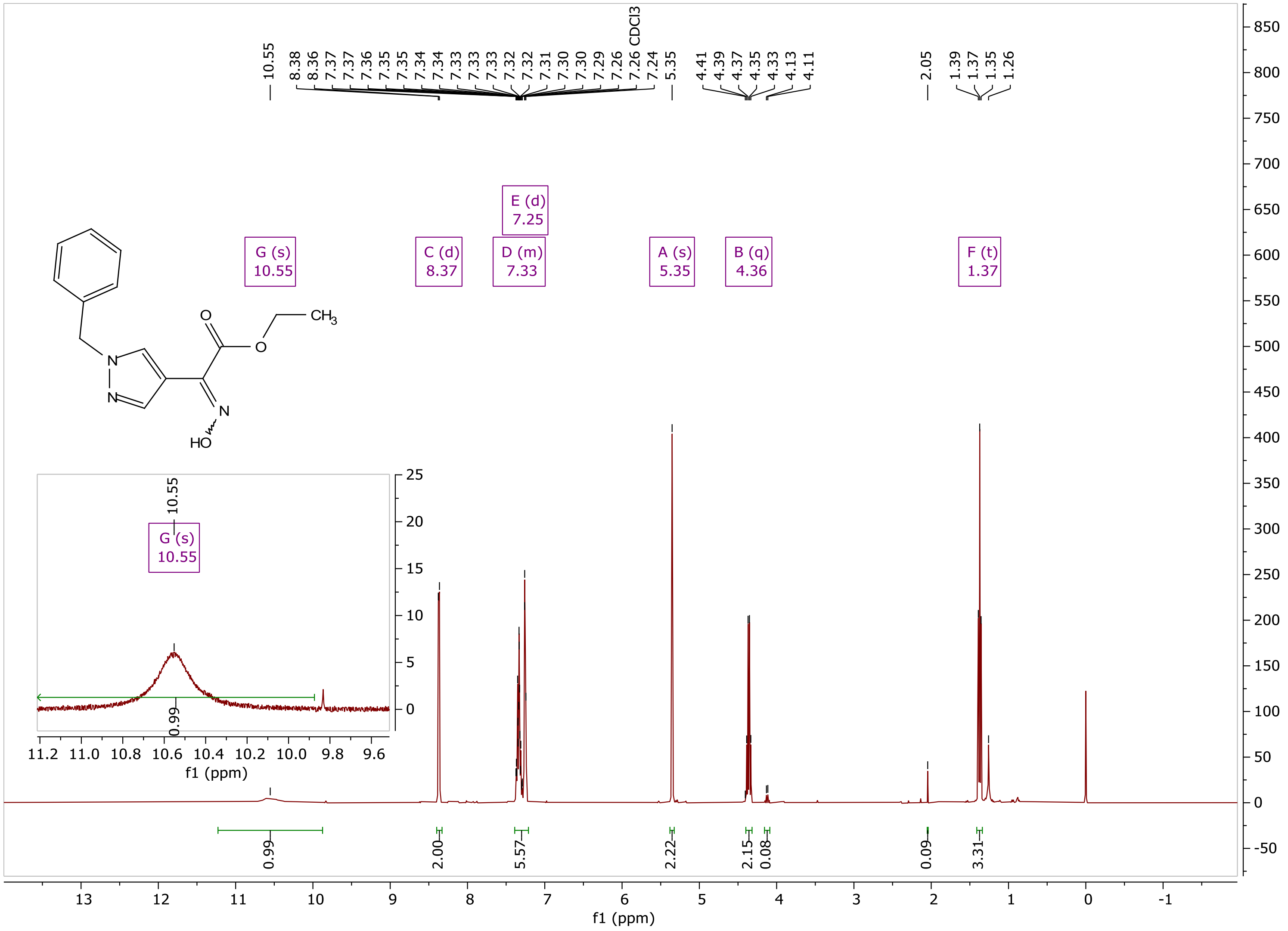

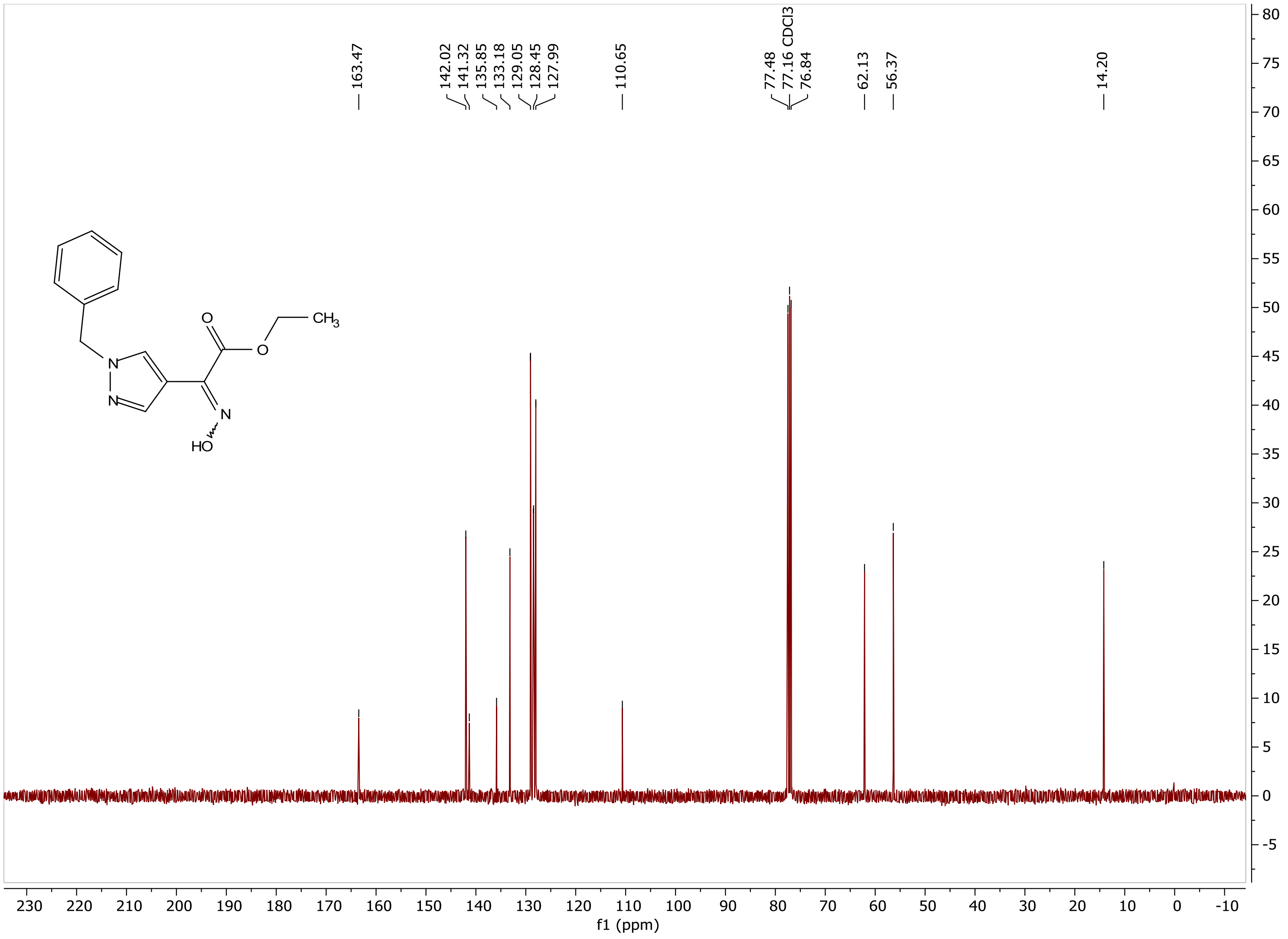

163.47
142.02
141.32
135.85
133.18
129.05
128.45
127.99
110.65
77.48
77.16 CDCl3
76.84
62.13
56.37
14.20
f1 (ppm)
O
O
CH3
N
N
N
HO

Ethyl 2-(hydroxyimino)-2-(1H-indazol-5-yl)acetate **(5r)** *(CD_3OD, ^1H-NMR: 400 MHz, ^{13}C-NMR: 101 MHz, HMBC, HSQC)*

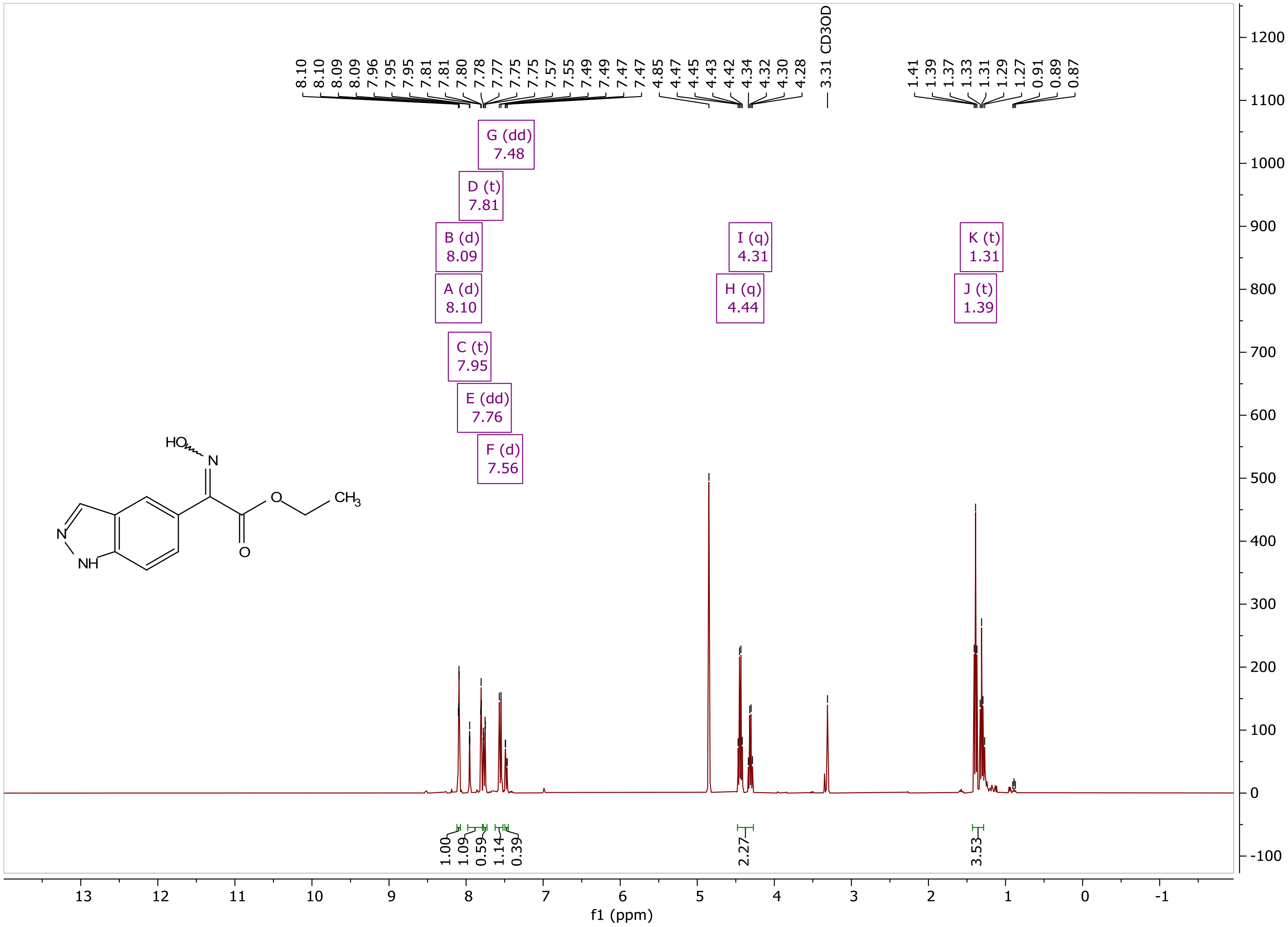

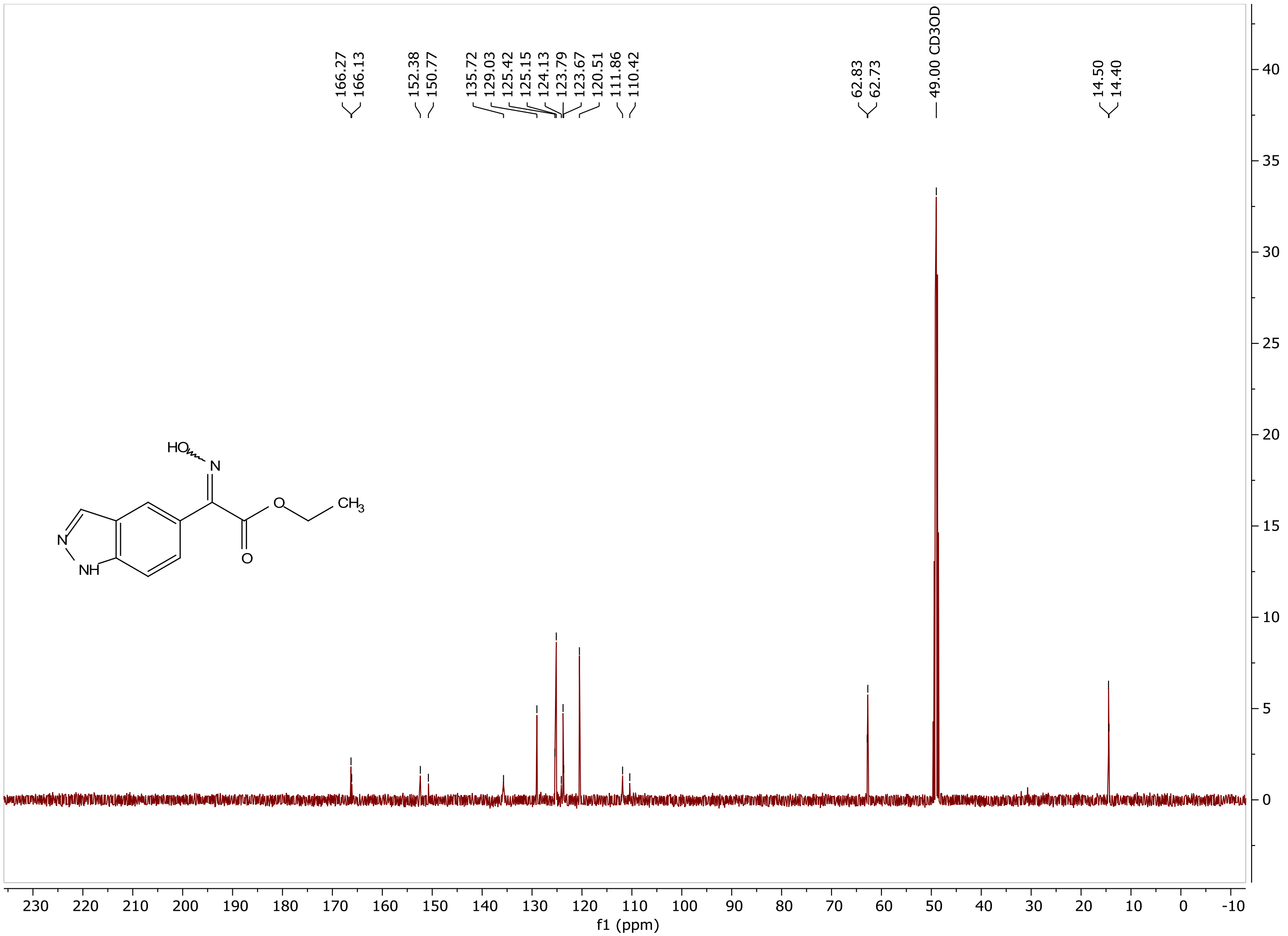
166.27
166.13
152.38
150.77
135.72
129.03
125.42
125.15
124.13
123.79
123.67
120.51
111.86
110.42
62.83
62.73
49.00 CD3OD
14.50
14.40
HO
N
N
NH
O
O
CH3
f1 (ppm)

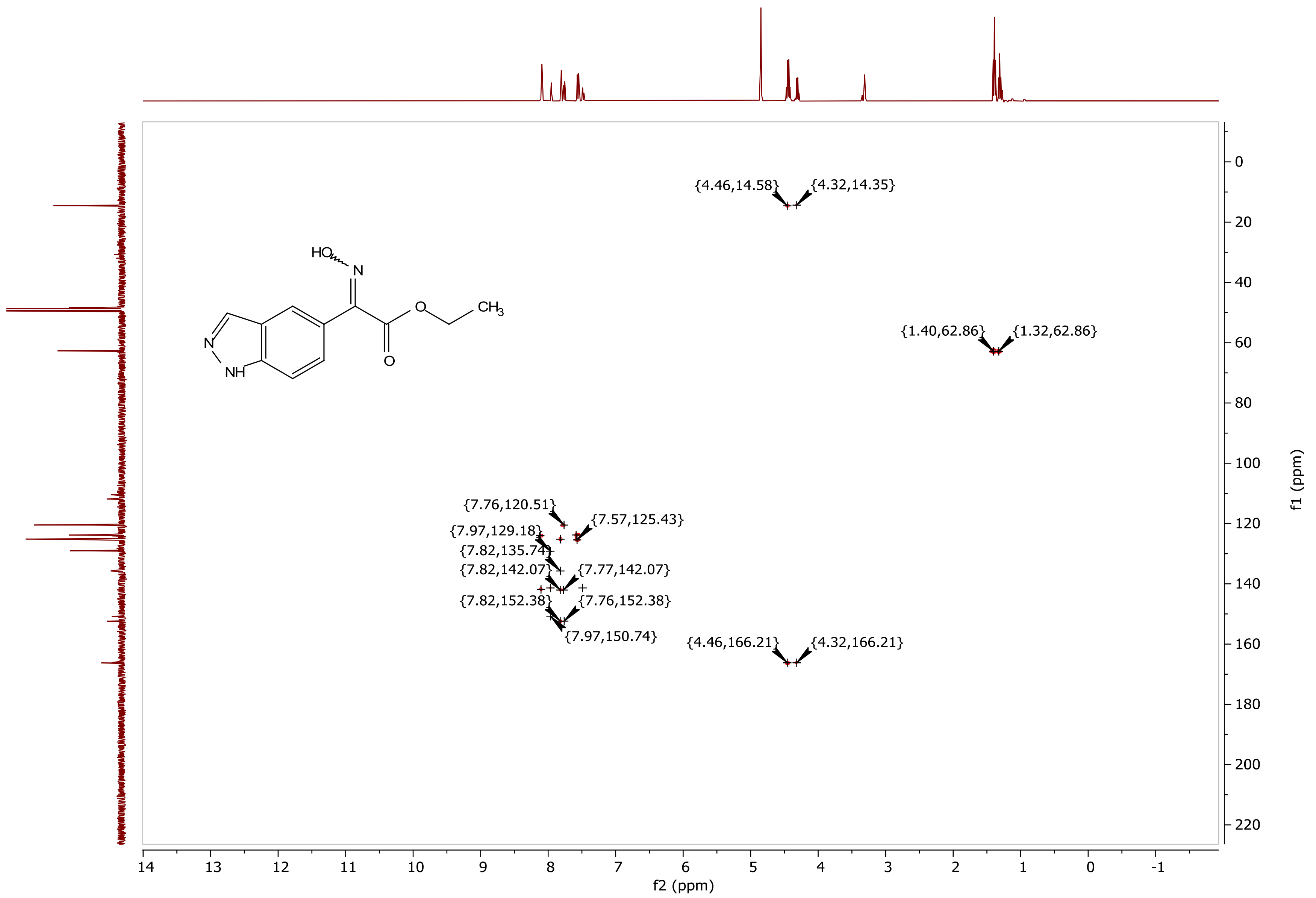

HO
N
O
CH3
N
NH
O
{4.46,14.58}
{4.32,14.35}
{1.40,62.86}
{1.32,62.86}
{7.76,120.51}
{7.57,125.43}
{7.97,129.18}
{7.82,135.74}
{7.82,142.07}
{7.77,142.07}
{7.82,152.38}
{7.76,152.38}
{7.97,150.74}
{4.46,166.21}
{4.32,166.21}
f2 (ppm)
f1 (ppm)

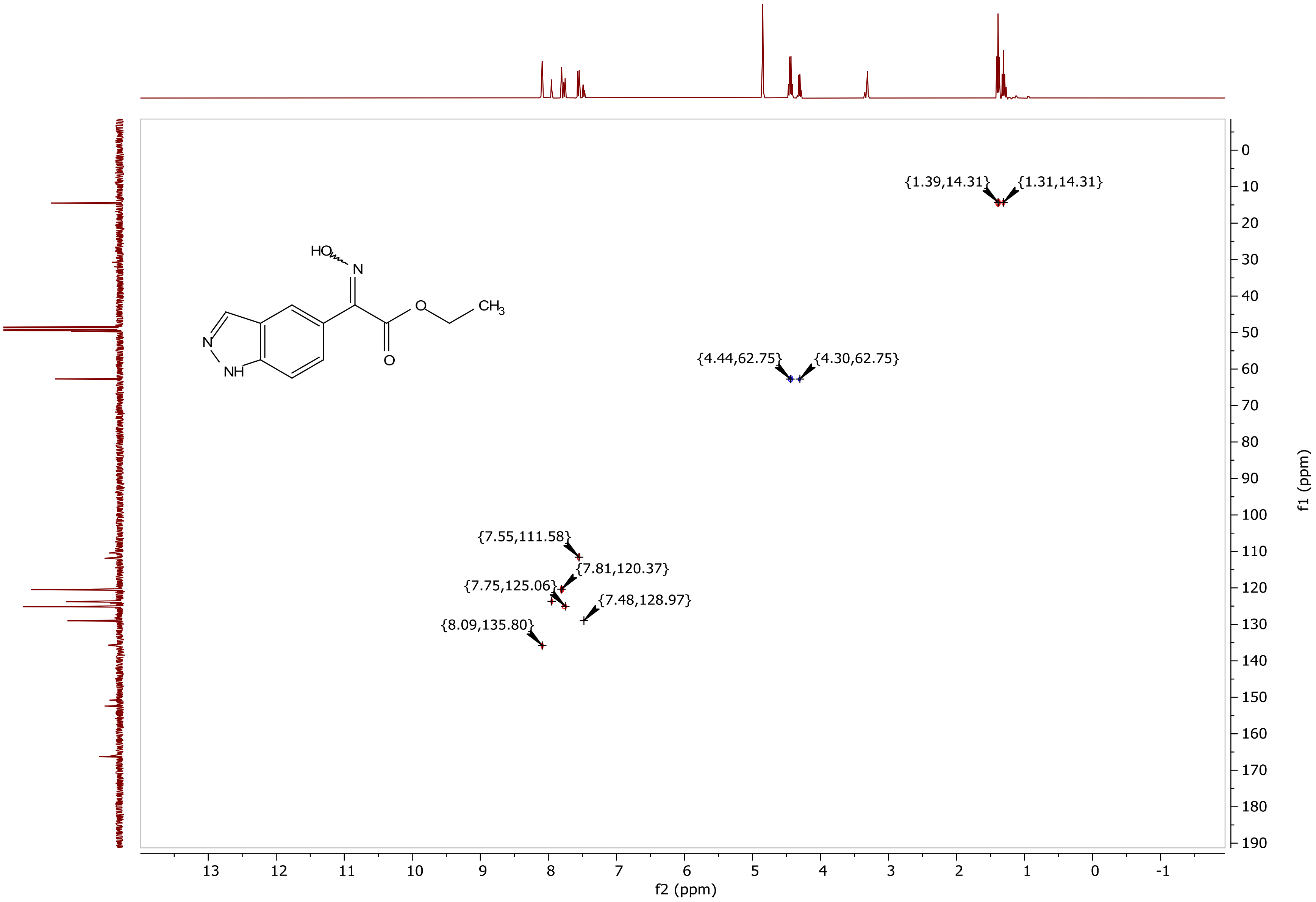
HO
N
O
CH3
N
NH
O
{1.39,14.31}
{1.31,14.31}
{4.44,62.75}
{4.30,62.75}
{7.55,111.58}
{7.81,120.37}
{7.75,125.06}
{7.48,128.97}
{8.09,135.80}
f1 (ppm)
f2 (ppm)

Ethyl 2-(hydroxyimino)-2-(1-methyl-1H-benzo[d]imidazol-2-yl)acetate **(5t)** *(CD_3OD, ^{1}H-NMR: 400 MHz, ^{13}C-NMR: 101 MHz)*

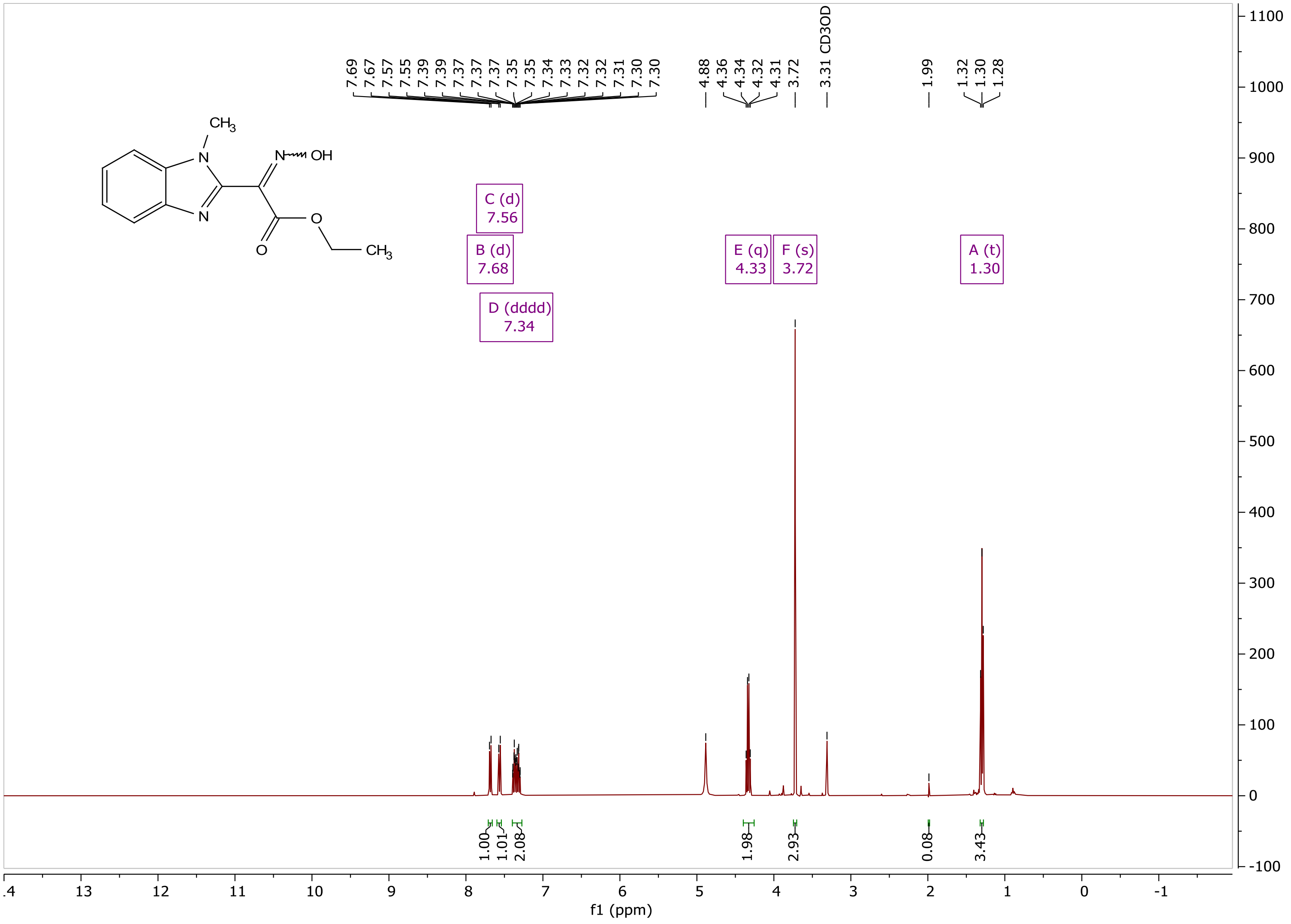

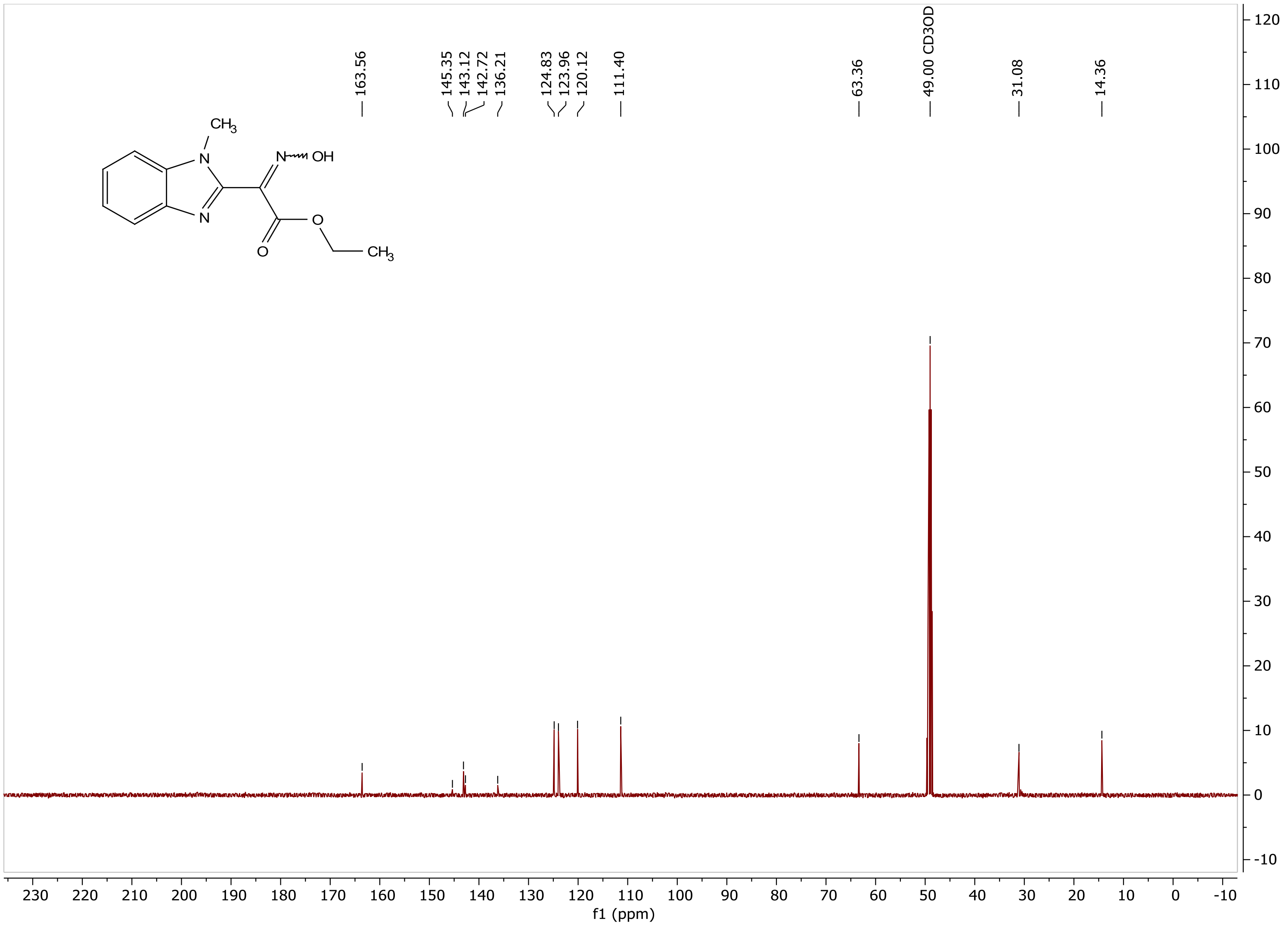
163.56
145.35
143.12
142.72
136.21
124.83
123.96
120.12
111.40
63.36
49.00 CD3OD
31.08
14.36
CH3
N
N
N
OH
O
O
CH3
f1 (ppm)

NMR spectra amino esters

Ethyl 2-((tert-butoxycarbonyl)amino)-2-(thiazol-2-yl)acetate ***(6b)*** *($CDCl_3$, ^{1}H-NMR: 400 MHz, ^{13}C-NMR: 101 MHz)*

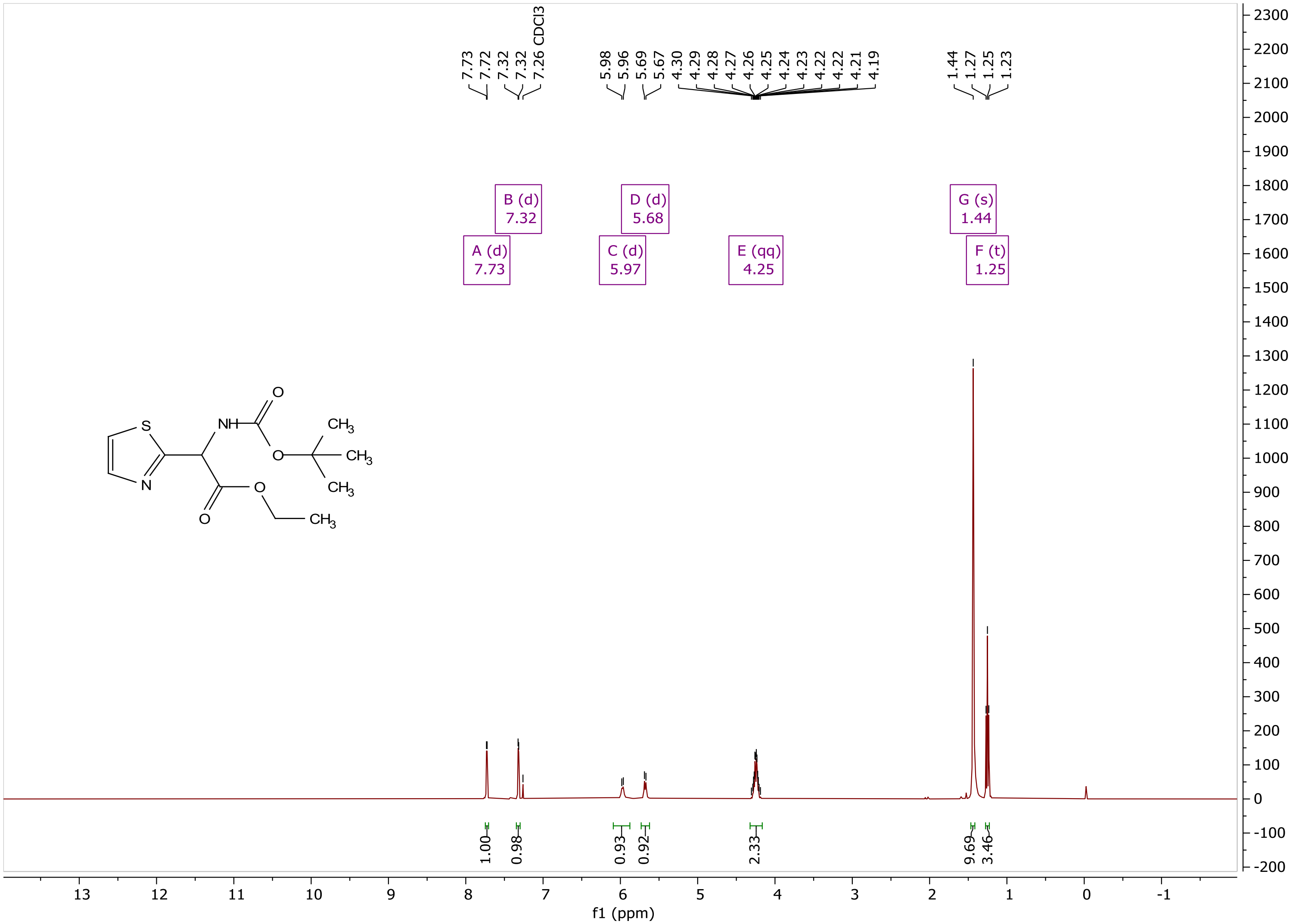

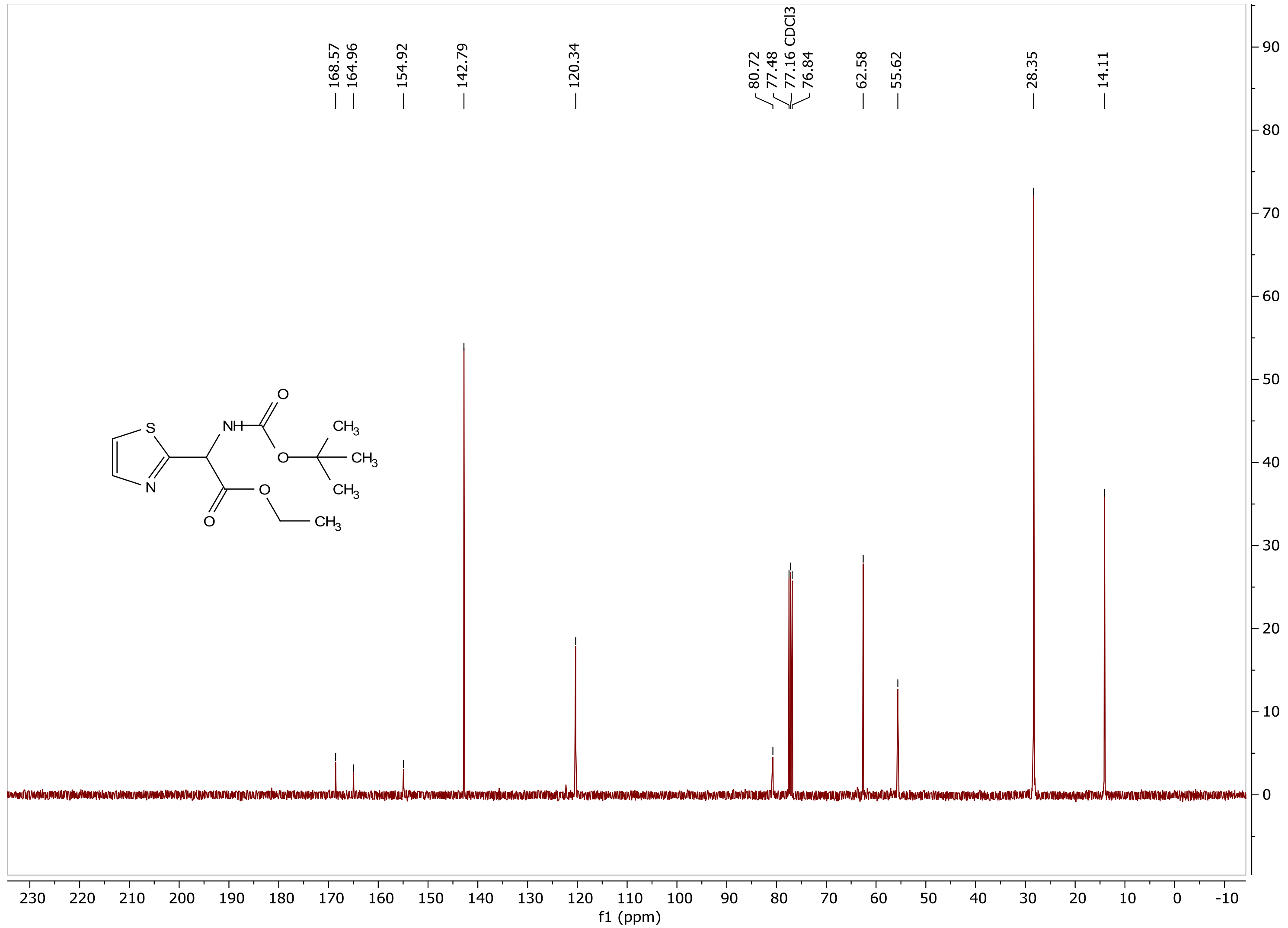

168.57
164.96
154.92
142.79
120.34
80.72
77.48
77.16 CDCl3
76.84
62.58
55.62
28.35
14.11
S
N
NH
O
O
O
O
CH3
CH3
CH3
CH3
f1 (ppm)

Ethyl 2-amino-2-(benzo[d]thiazol-2-yl)acetate hydrochloride ***(6i)*** *(DMSO-d_6, ^{1}H-NMR: 400 MHz, ^{13}C-NMR: 101 MHz)*

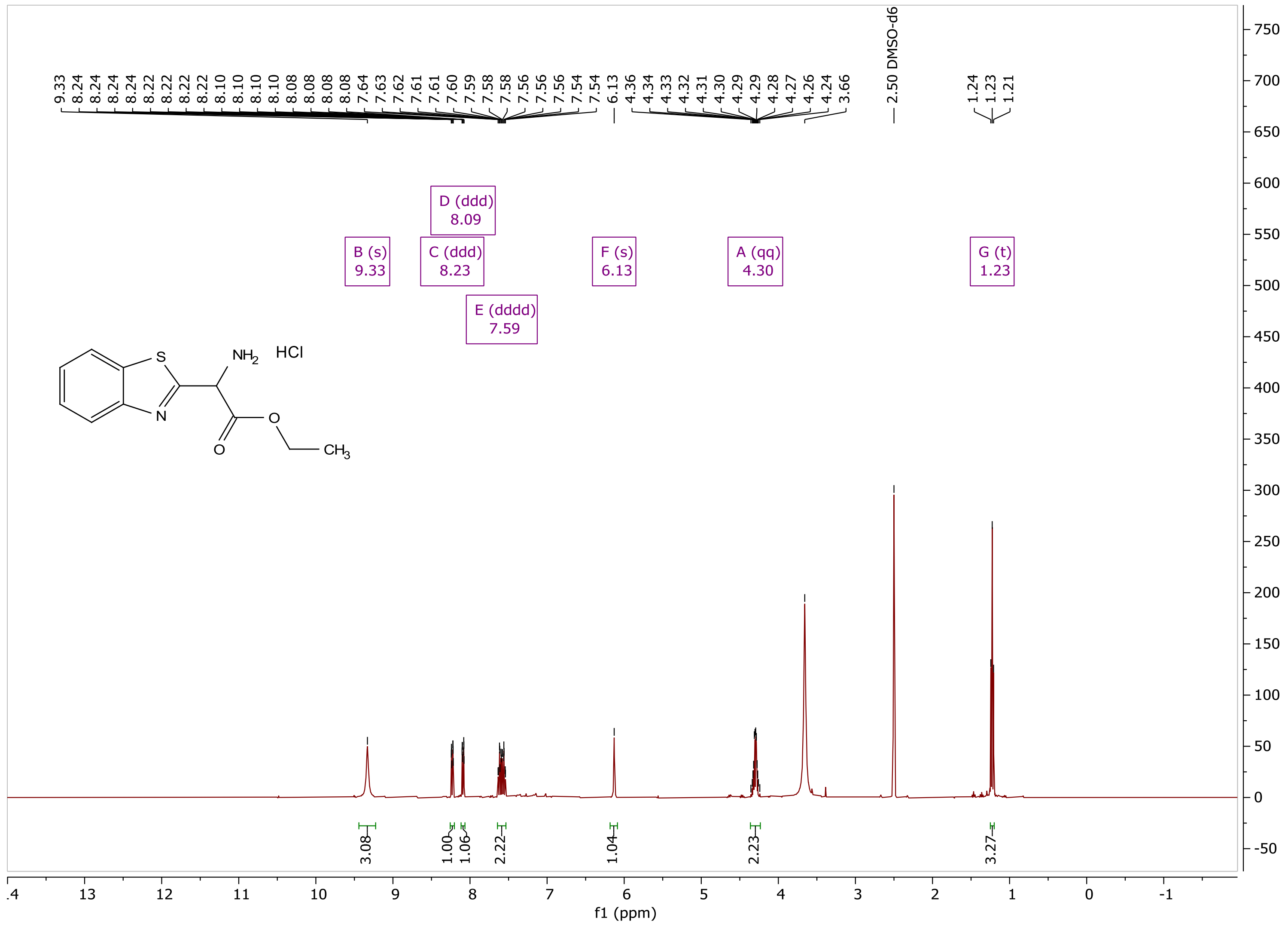

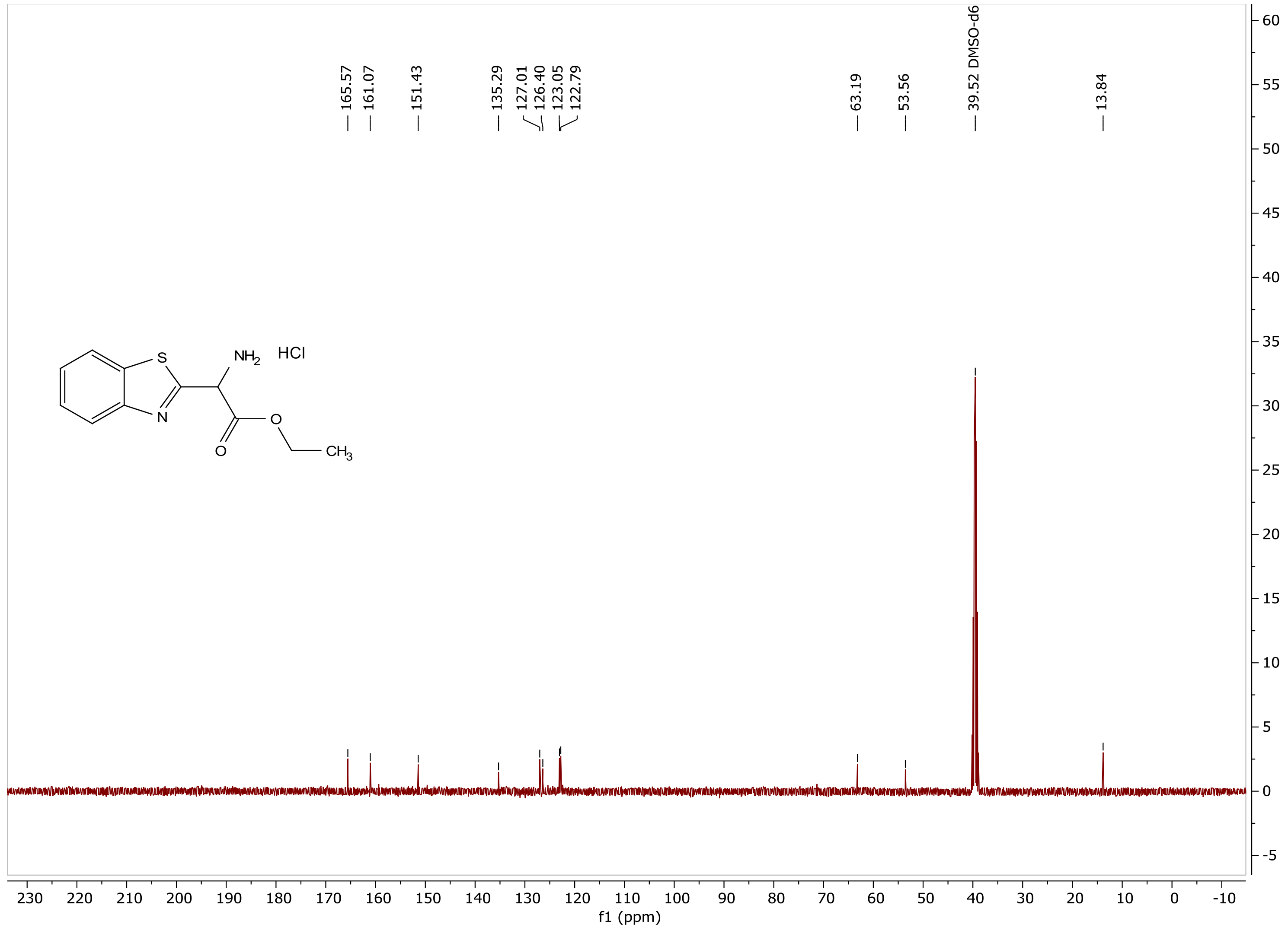

165.57
161.07
151.43
135.29
127.01
126.40
123.05
122.79
63.19
53.56
39.52 DMSO-d6
13.84
S
N
NH2
HCl
O
O
CH3
230
220
210
200
190
180
170
160
150
140
130
120
110
100
90
80
70
60
50
40
30
20
10
0
-10
f1 (ppm)
60
55
50
45
40
35
30
25
20
15
10
5
0
-5

Ethyl 2-amino-2-(furan-3-yl)acetate · hydrochloride ***(6j)*** *(CD_3OD, ^{1}H-NMR: 500 MHz, ^{13}C-NMR: 126 MHz)*

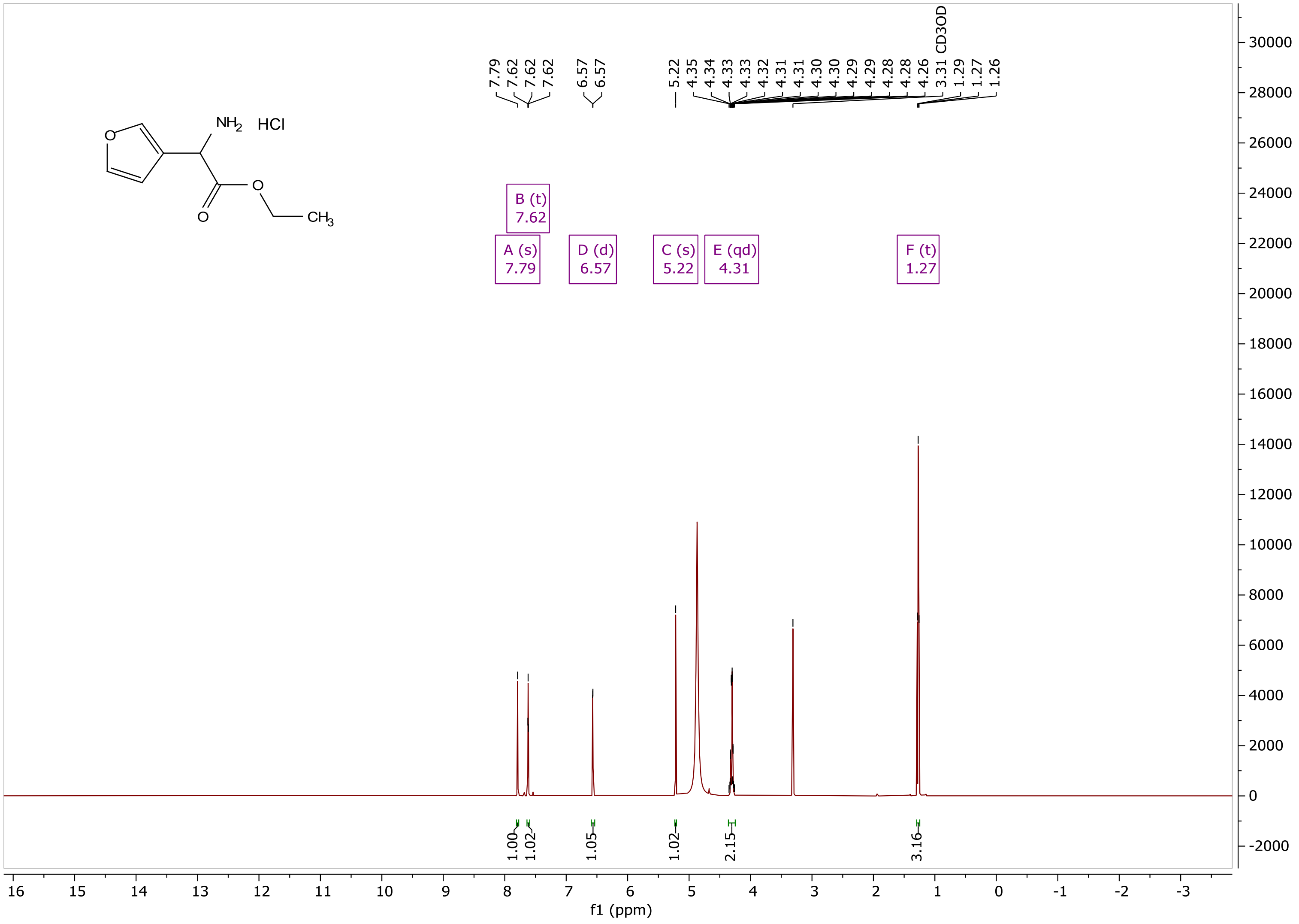

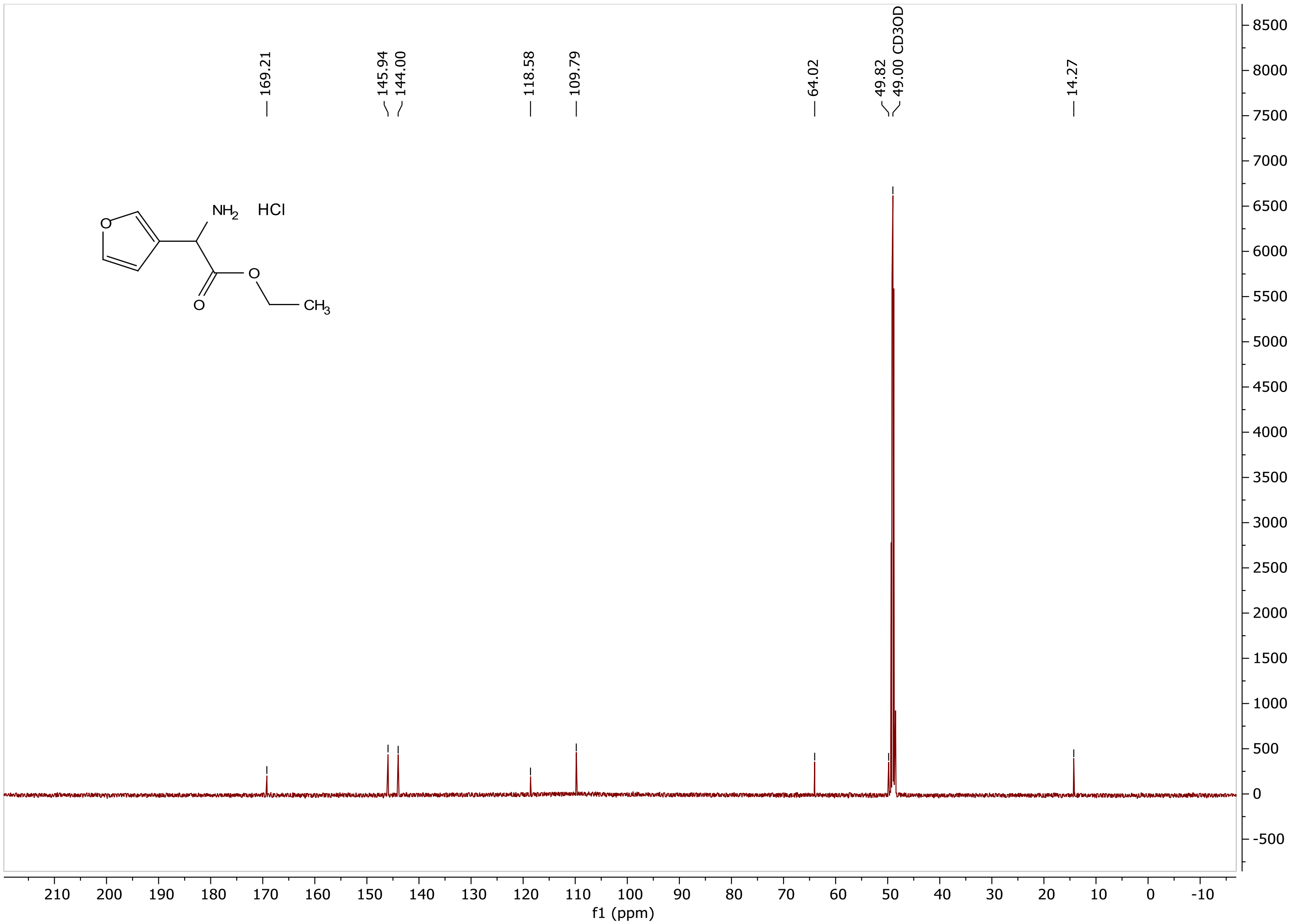
169.21
145.94
144.00
118.58
109.79
64.02
49.82
49.00 CD3OD
14.27
NH2
HCl
O
O
O
CH3
f1 (ppm)

Ethyl 2-amino-2-(1-benzyl-1H-pyrazol-3-yl)acetate hydrochloride **(6l)** *(CD_3OD, ^{1}H-NMR: 400 MHz, ^{13}C-NMR: 101 MHz)*

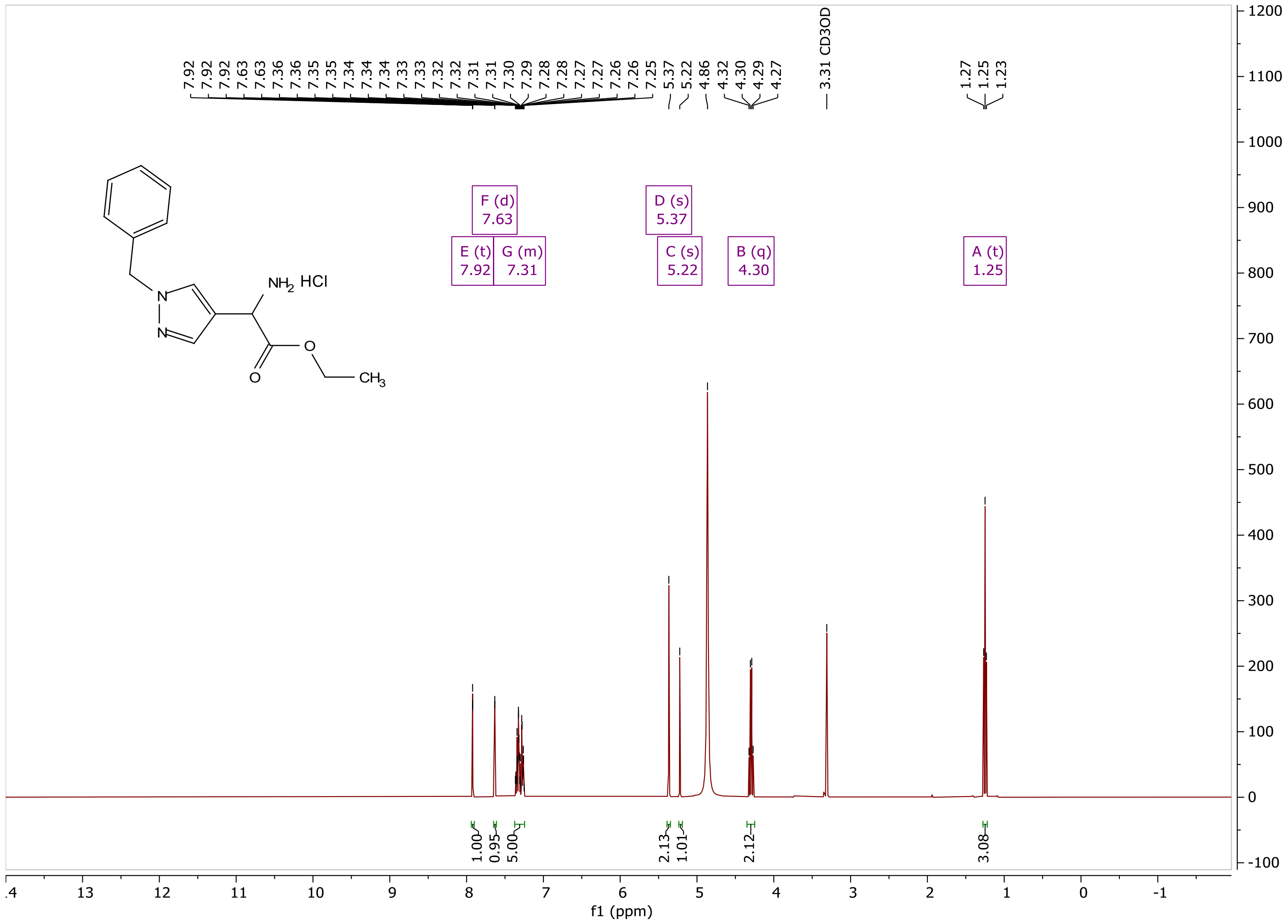

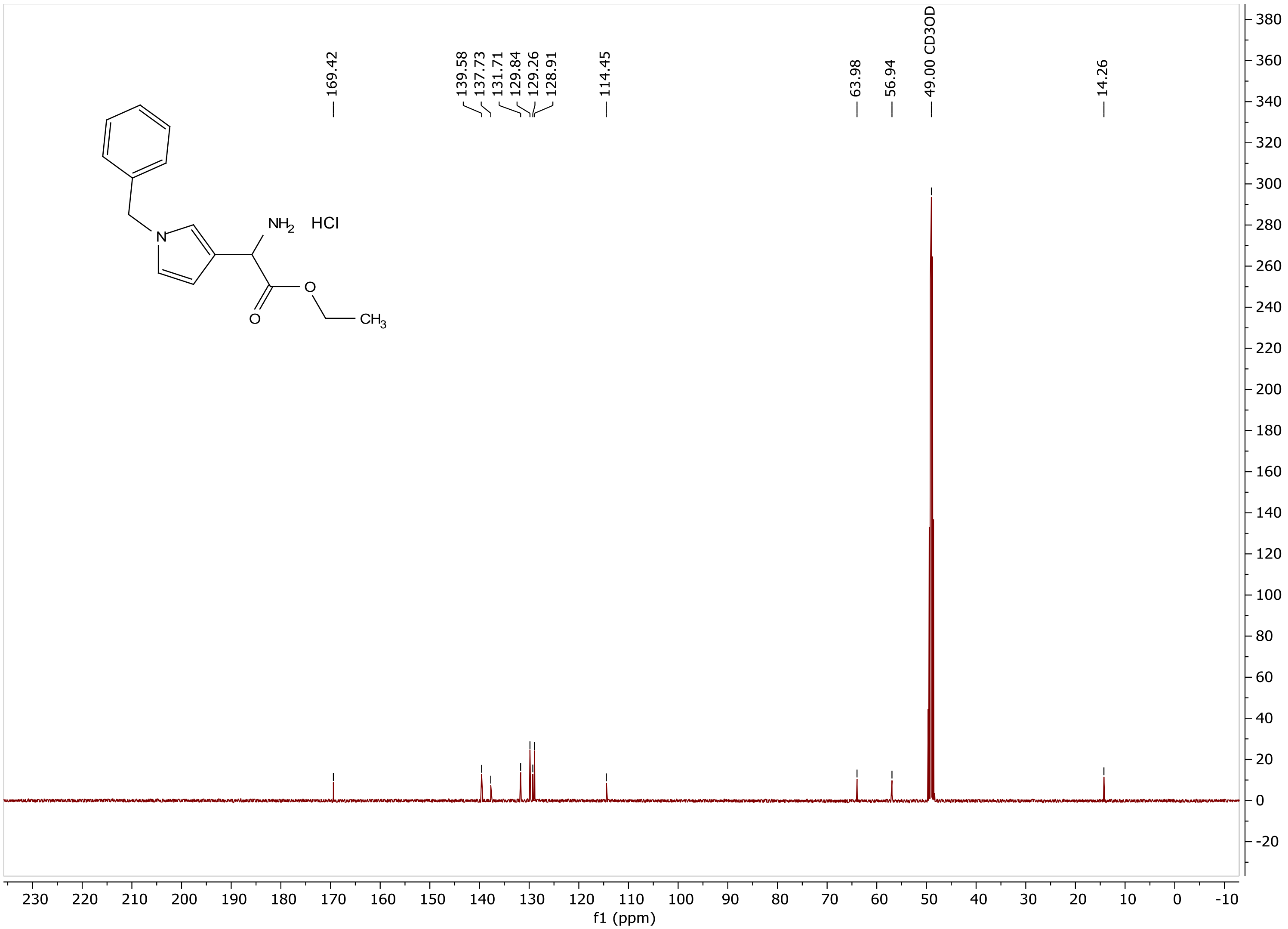
NH2
HCl
N
O
O
CH3
169.42
139.58
137.73
131.71
129.84
129.26
128.91
114.45
63.98
56.94
49.00 CD3OD
14.26
f1 (ppm)

Ethyl 2-amino-2-(1H-indazol-5-yl)acetate hydrochloride **(6r)** *(CD_3OD, ^{1}H-NMR: 400 MHz, ^{13}C-NMR: 101 MHz)*

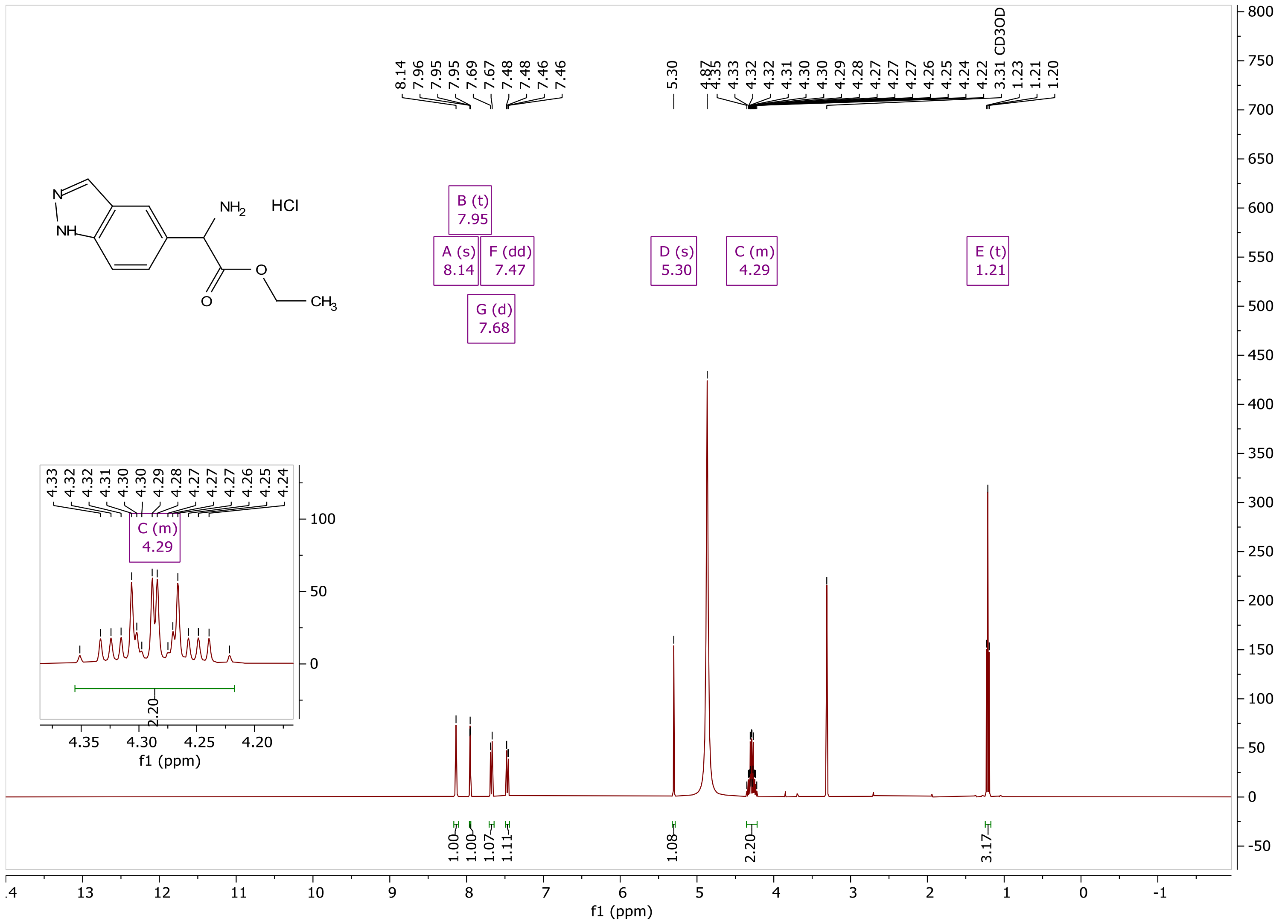

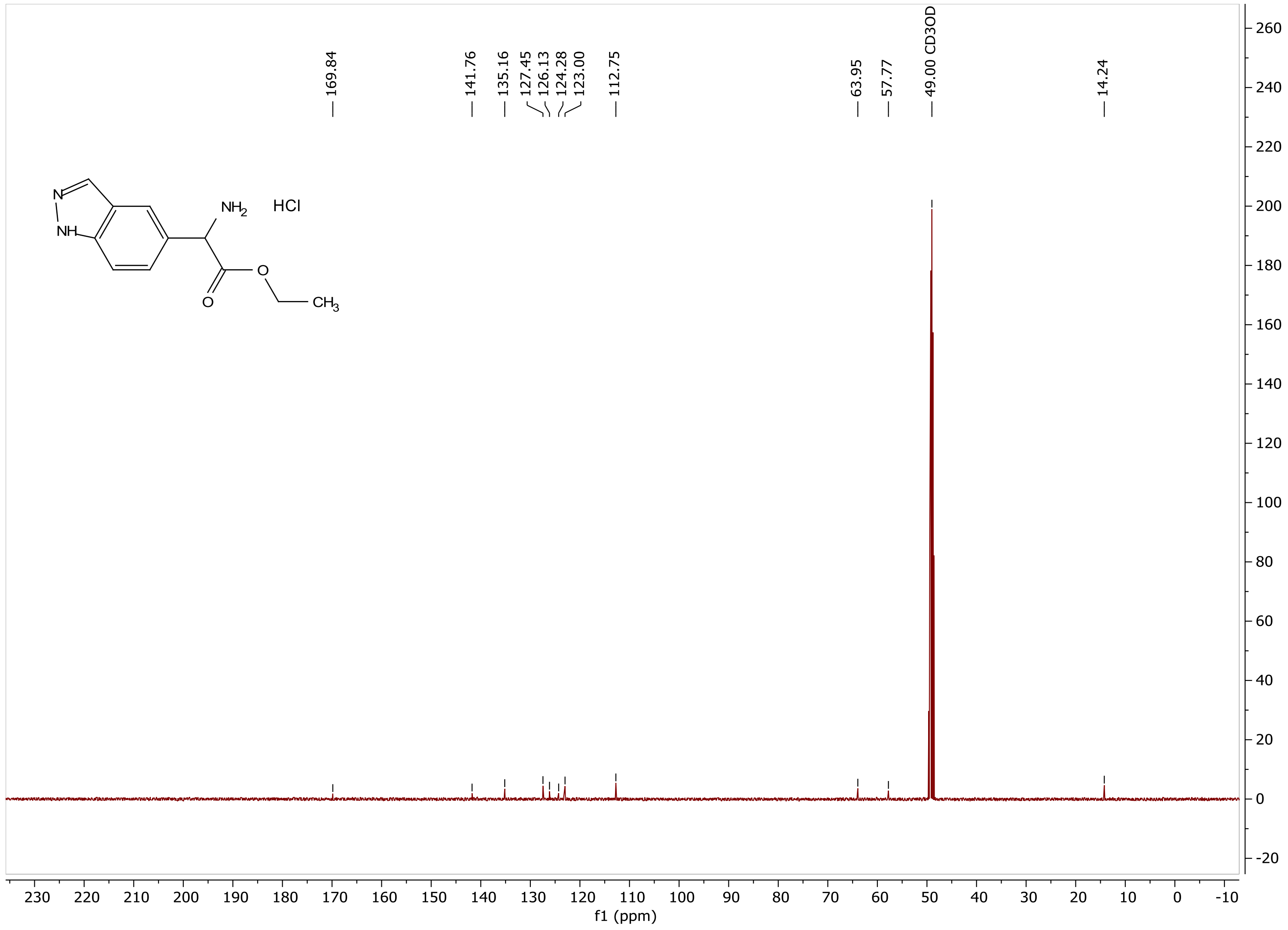
169.84
141.76
135.16
127.45
126.13
124.28
123.00
112.75
63.95
57.77
49.00 CD3OD
14.24
NH2
HCl
N
NH
O
O
CH3
f1 (ppm)

Ethyl 2-((tert-butoxycarbonyl)amino)-2-(1-methyl-1H-benzo[d]imidazol-2-yl)acetate ***(6t)*** *($CDCl_3$, ^1H-NMR: 400 MHz, ^{13}C-NMR: 101 MHz)*

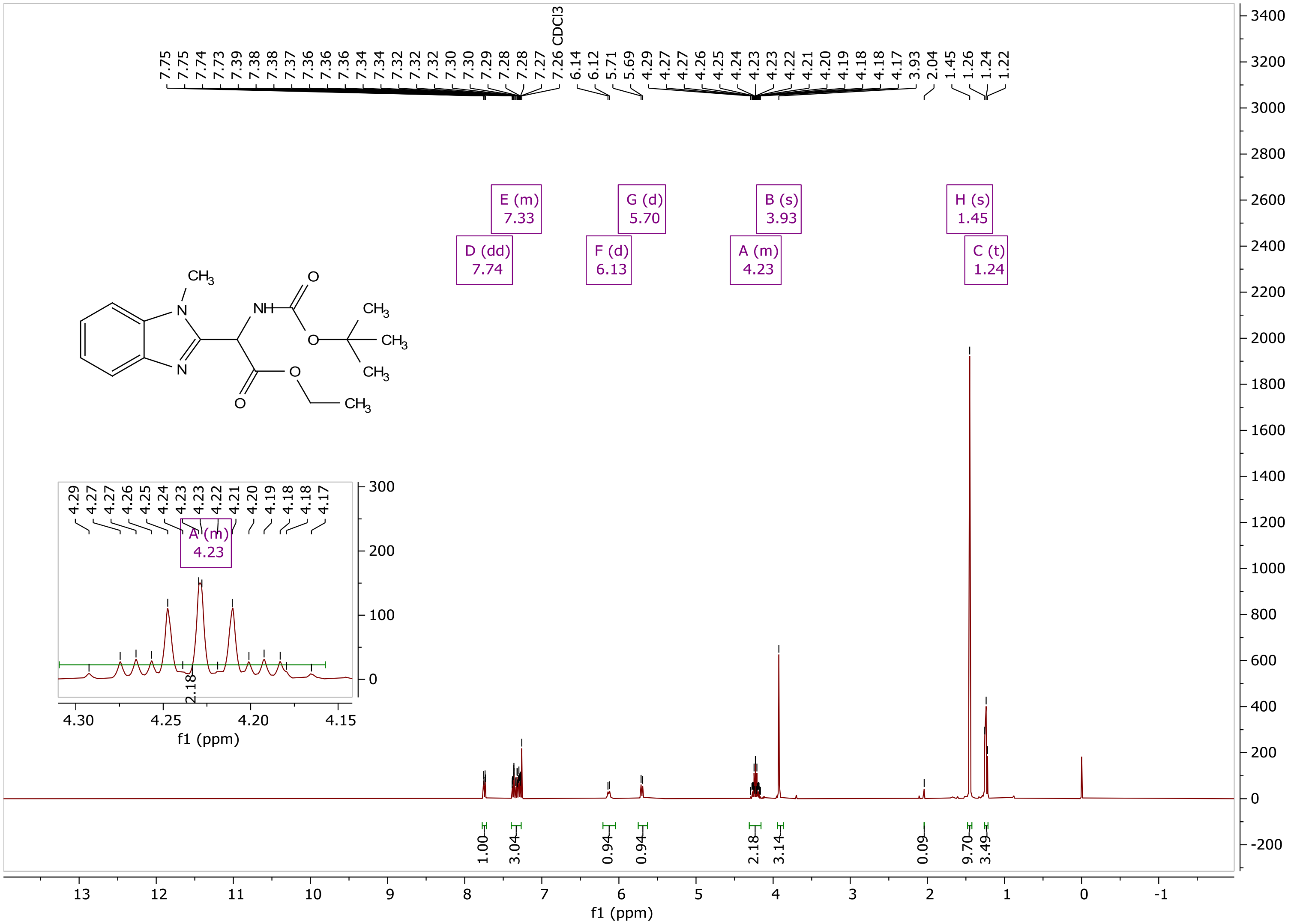

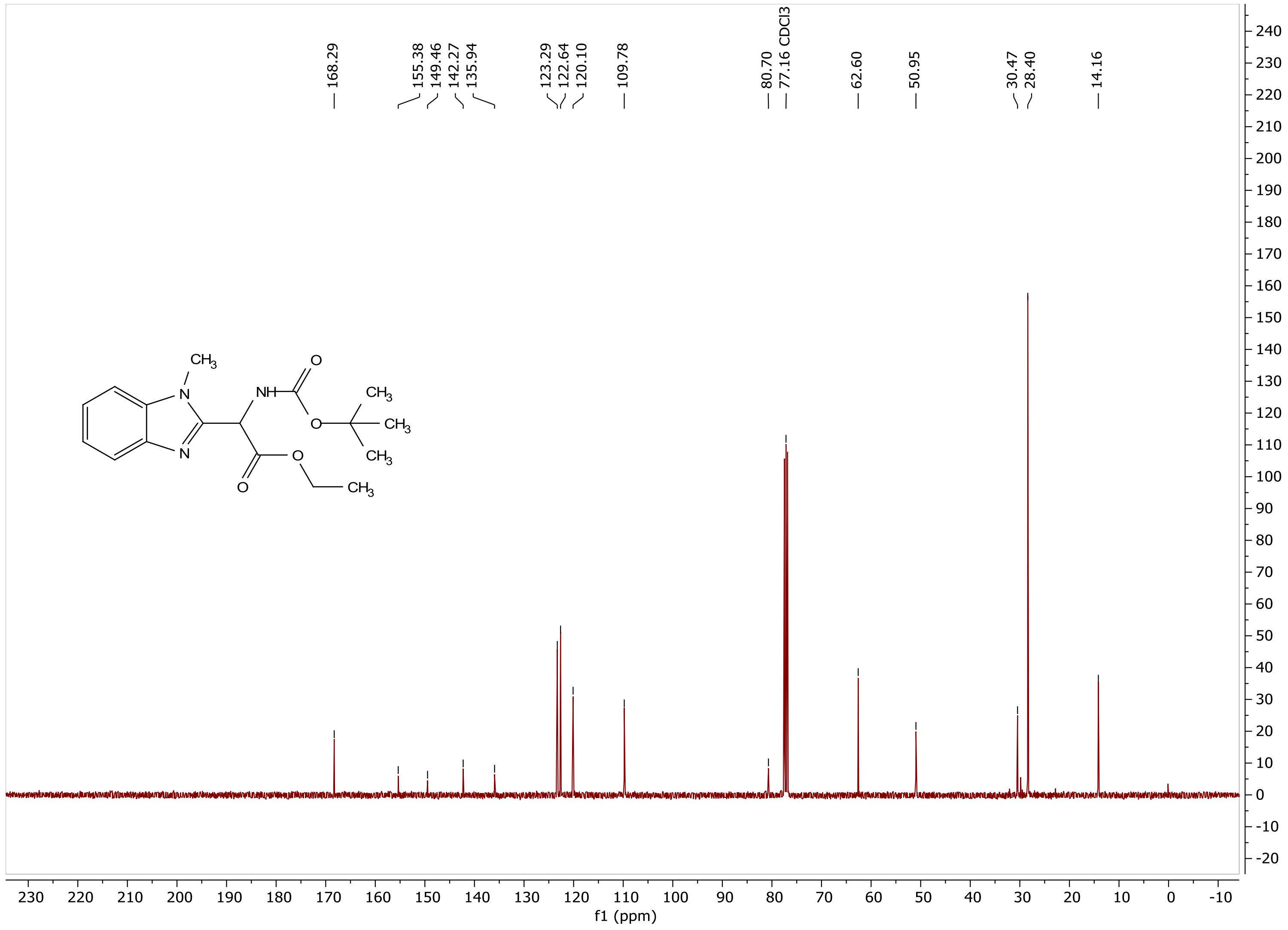

168.29
155.38
149.46
142.27
135.94
123.29
122.64
120.10
109.78
80.70
77.16 CDCl3
62.60
50.95
30.47
28.40
14.16
f1 (ppm)

References

1. Clark, M. A.; Acharya, R. A.; Arico-Muendel, C. C.; Belyanskaya, S. L.; Benjamin, D. R.; Carlson, N. R.; Centrella, P. A.; Chiu, C. H.; Creaser, S. P.; Cuozzo, J. W.; Davie, C. P.; Ding, Y.; Franklin, G. J.; Franzen, K. D.; Gefter, M. L.; Hale, S. P.; Hansen, N. J. V.; Israel, D. I.; Jiang, J.; Kavarana, M. J.; Kelley, M. S.; Kollmann, C. S.; Li, F.; Lind, K.; Mataruse, S.; Medeiros, P. F.; Messer, J. A.; Myers, P.; O'Keefe, H.; Oliff, M. C.; Rise, C. E.; Satz, A. L.; Skinner, S. R.; Svendsen, J. L.; Tang, L.; Van Vloten, K.; Wagner, R. W.; Yao, G.; Zhao, B.; Morgan, B. A. *Nat. Chem. Biol.* **2009**, *5*, 647–654.
2. Song, S.; Sun, X.; Li, X.; Yuan, Y.; Jiao, N. *Org. Lett.* **2015**, *17*, 2886–2889.
3. Liu, X.; Zhao, X.; Liang, F.; Ren, B. *Org. Biomol. Chem.* **2018**, *16*, 886–890.
4. Berton, M.; Huck, L.; Alcázar, J. *Nat. Protoc.* **2018**, *13*, 324–334.
5. Collins, I.; Wafford, K.; Dawson, G. R.; Moyes, C.; Davey, W. B.; Rowley, M.; Bromidge, F. A.; Quirk, K.; Atack, J. R.; McKernan, R. M.; Thompson, S. A.; Pike, A.; Sohal, B.; Tsou, N. N.; Ball, R. G.; Castro, J. L. *J. Med. Chem.* **2002**, *45*, 1887–1900.
6. Takaya, T.; Takasugi, H.; Masugi, T.; Yamanaka, H.; Kawabata K. 7-Acylamino-3-Vinylcephalosporanic Acid Derivatives and Processes for the Preparation Thereof. U.S. Patent 4409214, October 11, 1983.
7. Song, X.; Qin, W.; Wang, X.; Luo, G.; Ni, Q. *Org. Lett.* **2023**, *25*, 9164–9169.
8. Wenkert, E.; Guo, M.; Lavilla, R.; Porter, B.; Ramachandran, K.; Sheu, J.H. *J. Org. Chem.* **1990**, *51*, 6203-6214.
9. Wang, Y.; Huang, Y.; Qiu, R.; Tang T. Biaryl Compound, Preparation Method and Use Thereof. U.S. Patent 0002280, January 2, 2020.
10. Mylari, B. L.; Zembrowski, W. J.; Beyer, T. A.; Aldinger, C. E.; Siegel, T. W. *J. Med. Chem.* **1992**, *35,* 2155-2162.
11. Yao, J.; Feng, R.; Lin, C.; Liu, Z.; Zhang, Y. *Org. Biomol. Chem*. **2014**, 12, 5469–5476.
12. Konetzki, I.; Jakob, F.; Craan, T.; Hesslinger, C.; Ratcliffe, P.; Nardi A. Substituted Condensed Pyrimidine Compounds. International Patent WO 170020A1, October 23, 2014.
13. Wood, E.; Chapman, D. D.; Elwood, J. K.; Heseltine, D. W.; Hess, M.; Kurtz, D. W. *J. Org. Chem*. **1977**, 42, 2474–2480.
14. Mazuela, J.; Antonsson, T.; Johansson, M. J.; Knerr, L.; Marsden, S. P. *Org. Lett.* **2017**, *19*, 5541–5544.
15. Du, H. C.; Simmons, N.; Faver, J. C.; Yu, Z.; Palaniappan, M.; Riehle, K.; Matzuk, M. M. *Org. Lett.* **2019**, *21*, 2194–2199.
16. Satz, A. L.; Cai, J.; Chen, Y.; Goodnow, R.; Gruber, F.; Kowalczyk, A.; Petersen, A.; Naderi-Oboodi, G.; Orzechowski, L.; Strebel, Q. *Bioconjug. Chem.* **2015**, *26*, 1623–1632.
